# Next-step treatment for schizophrenia non-responsive to antipsychotics: a systematic review and network meta-analysis

**DOI:** 10.1016/j.eclinm.2026.103988

**Published:** 2026-05-22

**Authors:** Yuki Furukawa, Nurul Husna Salahuddin, Yaohui Wei, Elisavet Pinioti, David D. Kim, Filip Milosavlijević, Spyridon Siafis, Johannes Schneider-Thoma, Myrto Samara, Irene Bighelli, Stefan Leucht

**Affiliations:** a Technical University of Munich, TUM School of Medicine and Health, Department of Psychiatry and Psychotherapy, Munich, Germany; b German Center for Mental Health (DZPG) Partner Site Munich-Augsburg, FKZ 01EE2503B, Germany; c Department of Neuropsychiatry, University of Tokyo, Tokyo, Japan; d Shanghai Mental Health Centre, Shanghai Jiao Tong University School of Medicine, Shanghai, China; e Department of Psychiatry, University of Thessaly, Greece

**Keywords:** Treatment non-response, Treatment-resistant schizophrenia, Schizophrenia, Clozapine, Non-invasive brain stimulation, Electro-convulsive therapy, Psychotherapy

## Abstract

**Background:**

Antipsychotics are often insufficient for schizophrenia, but the optimal next-step strategies for non-response remain uncertain. Guidelines recommend some pharmacological, psychological, and non-invasive brain stimulation (NIBS) approaches, but we do not know which one works best. We summarized current evidence for schizophrenia non-responsive to antipsychotics.

**Methods:**

We searched the Cochrane Schizophrenia Group registry (up till January 13, 2025), and PubMed up till January 8, 2026 for randomized controlled trials (RCTs) of patients non-responsive to prior antipsychotic treatment, with persistent symptoms after at least one adequate 4-week antipsychotic trial. Raters needed to be masked. The primary outcome was change in overall symptoms analyzed using random-effects network meta-analyses of standardized mean differences (SMDs) with 95% confidence intervals (CIs). The protocol was pre-registered (https://osf.io/wcs5d/).

**Findings:**

Fifty-nine RCTs (5409 participants; mean age 39.8 years; 3416 men, 1441 women) were included; 5013 contributed to the primary analysis. Antipsychotic combination therapy (k = 18; n = 575; SMD −0.25, 95% CI −0.46 to −0.04; CINeMA: very low) and electroconvulsive therapy (ECT; k = 5; n = 97; SMD −0.51, −0.96 to −0.06; very low) might be more efficacious than continuing the same antipsychotic. Cognitive behavioral therapy for psychosis (CBTp) showed weak evidence of benefit over continuing the same antipsychotic (k = 5; n = 340; SMD −0.23, −0.58 to 0.11; very low). Other strategies—xanomeline–trospium augmentation, dose escalation, transcranial magnetic stimulation, and switching to clozapine or other antipsychotics—were inconclusive. Combination therapy increased adverse events (OR 1.93, 1.26–3.00). We found no evidence of subgroup difference among non-clozapine- and clozapine-non-response.

**Interpretation:**

The results were very uncertain. More head-to-head trials are needed. We found no evidence supporting dose-escalation nor switching to clozapine. Very weak evidence suggested efficacy of antipsychotic combination and ECT augmentation and they might be considered, but with substantial caution.

**Funding:**

This study was funded by the 10.13039/501100001659German Research Foundation (DFG, #468853597) and the Federal Ministry of Research, Technology, and Space (BMTR, #01EE2303B), and partly by a grant from 10.13039/501100008667SENSHIN Medical Research Foundation given to YF, DAAD fellowship to NHS and CSC scholarship to YHW.

**Research in context**

**Evidence before this study**

Up to two-thirds of patients with schizophrenia do not respond adequately to antipsychotics. In case of non-response, clinical practice guidelines recommend several pharmacotherapies (switching to non-clozapine, combining antipsychotics, and in case of treatment-resistance, switching to clozapine), cognitive behavioral therapy for psychosis, and non-invasive brain stimulation (electro-convulsive therapy [ECT] and transcranial magnetic stimulation [TMS]). However, the comparative efficacy of the different treatments is not known.

We searched PubMed on November 4 2025 with the search term (schizophrenia AND ((treatment resist∗) OR non-response) and filter “Article type: Meta-analysis”, which resulted in 148 records. We found four network meta-analyses on treatment-resistant schizophrenia, but three of them focused on either pharmacotherapies or psychotherapies, and the other focused on clozapine-resistant schizophrenia. One meta-analysis examined pharmacological interventions in schizophrenia with early non-response. No evidence was found for the comparative efficacy of pharmacological, psychological and non-invasive brain stimulation (NIBS) treatments for people with schizophrenia non-responsive to antipsychotics.

**Added value of this study**

This study summarized the currently available evidence on the comparative efficacy, acceptability, and tolerability of pharmacological, psychological and NIBS interventions for people with schizophrenia non-responsive to antipsychotics. We included 59 randomized controlled trials in 5409 participants and compared pharmacological, psychological and NIBS interventions. We found very weak evidence for combining D2R-targeting antipsychotics and ECT augmentation compared to continuation of the same antipsychotic used. There was suggestive evidence for CBTp, but other strategies (dose-escalation, switching to clozapine/non-clozapine, xanomeline-trospium augmentation, and TMS augmentation) were even more uncertain and inconclusive.

**Implications of all the available evidence**

No interventions can be recommended with moderate or high confidence in the evidence. We found very weak evidence that D2R-targeting antipsychotic combination and ECT augmentation might be considered in case of non-response to antipsychotics, but with substantial caution. CBTp might also be considered. Further research, especially head-to-head trials are warranted.

## Introduction

“What is the best way to treat people with schizophrenia that is unresponsive to treatment?” This is the top research priority identified by patients, their caregivers and clinicians through the James Lind Alliance Priority Setting Partnership.^1^ Despite the availability of dozens of antipsychotic medications, up to two-thirds of patients with schizophrenia experience insufficient symptom improvement.^2^

When first-line antipsychotic therapy fails, clinicians may consider several broad treatment strategies: pharmacological adjustments, psychological interventions, or non-invasive brain stimulation (NIBS).^3^ Our previous network meta-analysis (NMA) compared individual pharmacological agents in this population,^4^ but the granularity of intervention definitions meant that most treatments were often assessed only in a single trial or a handful of trials at best, limiting the statistical power and generalizability of findings. Furthermore, the inclusion of experimental agents not approved for schizophrenia (e.g., oxytocin, memantine) reduced clinical applicability.

To date, evidence syntheses have largely focused on single modalities—separate meta-analyses exist for pharmacological,^4^, ^5^, ^6^ psychological,^7^ or NIBS approaches^8^—but no comprehensive comparison has been made across these therapeutic domains.

In this study, we conducted the first network meta-analysis to integrate data across pharmacological, psychological, and NIBS strategies for patients with schizophrenia who do not respond adequately to antipsychotic treatment, aiming to provide a unified evidence base to inform clinical decision-making and to answer the top research priority mentioned above.

## Methods

We followed the Preferred Reporting Items for Systematic reviews and Meta-Analyses (PRISMA) guideline for network meta-analysis.^9^ The protocol was prospectively registered in the Open Science Framework (https://osf.io/wcs5d/) and can be found in the eAppendix 1. People with lived experience were not directly involved in the research process.

### Eligibility criteria and search strategies

#### Study design

We included all randomized controlled trials (RCTs) that compared at least two of the interventions listed below, with at least the rater masked. In case of cross-over trials, we used only the first phase to avoid the carry-over effects.

#### Participants

We included studies of adults with schizophrenia or related disorders (such as schizophreniform, or schizoaffective disorders) who did not respond to antipsychotics. There were no restrictions in terms of sex, ethnicity, or setting. We included all the diagnostic criteria and tested the effect of including studies without a formal diagnosis in a sensitivity analysis. Trials were accepted if 80% or more participants had schizophrenia or related disorders, but non-response to antipsychotics needed to be part of the inclusion criteria.

We defined non-response as having clinically relevant symptoms after trying at least one antipsychotic with an adequate dose for at least four weeks. This is slightly more lenient than treatment-resistant schizophrenia (TRS) according to the Treatment Resistance and Response in Psychosis consensus criteria.^10^ We later conducted post-hoc tests to examine the effect of including trials that included patients with one antipsychotic trial failure and those that included patients non-responsive to clozapine. Trials that did not report clear dose or duration of the antipsychotic were not accepted. These criteria are stricter than the criteria we used for our previous meta-analyses of antipsychotics for TRS.^4^, ^5^, ^6^^,^^11^ We aimed to minimize clinical heterogeneity among the studies and ensure the transitivity among the network, which is the prerequisite for conducting network meta-analysis. Transitivity means that the included trials are similar enough to make the indirect comparisons reasonable. For example, this requires that key effect modifiers—such as patient characteristics, and disease severity—are similarly distributed across the different comparisons.^12^ We examined the impact of including patients non-responsive to one antipsychotic trial, and patients non-responsive to clozapine in sensitivity analyses. Finally, we excluded trials focusing on patients with predominantly negative symptoms, as this group may respond differently.^13^

#### Interventions and controls

We included the following interventions that are recommended in major clinical practice guidelines (the American Psychiatric Association, US; the National Institute for Health and Care Excellence, UK; the German Association for Psychiatry, Psychotherapy and Psychosomatics, Germany; Japanese Society of Neuropsychopharmacology, Japan):^3^^,^^14^, ^15^, ^16^

1. Continuation of the antipsychotic monotherapy [reference]
2. Dose-escalation of the antipsychotic used
3. Switching to another antipsychotic (other than clozapine) monotherapy
4. Switching to clozapine monotherapy
5. Combining two D2R-targetting antipsychotics
6. Xanomeline-trospium augmentation
7. Cognitive behavioral therapy for psychosis (CBTp) augmentation
8. Non-invasive brain stimulations augmentation
  8.1 Electroconvulsive therapy (ECT) augmentation
  8.2 Transcranial magnetic stimulation (TMS) augmentation

The control groups were classified as follows: continuation of the antipsychotic monotherapy including those combined with inactive interventions, such as placebo drugs, sham stimulations, and psychological placebo; when clozapine was already used before randomization and it was continued in the trial phase, it was regarded as continuation of the antipsychotic monotherapy; where multiple arms were reported in a single trial, we included only the relevant arms; we lumped the arms, if multiple arms were categorized in the same node. In contrast to Samara et al.,^4^ we grouped all antipsychotics except clozapine and xanomeline-trospium. This decision is methodologically justified, given that most antipsychotics share a common mechanism—dopamine D2 receptor antagonism—while the precise role of other receptor profiles remains unclear. Clozapine and xanomeline-trospium were analyzed separately due to their distinct clinical and pharmacological properties.^17^^,^^18^

### Primary outcome and secondary outcomes

The primary outcome was the overall symptoms (continuous) measured by using validated rating scale at post treatment, but the minimum duration was set at three weeks. We prioritized the Positive and Negative Syndrome Scale (PANSS). If not reported, we used the following scales in this order: the Brief Psychiatric Rating Scale (BPRS), and then any other validated scales, including the Clinical Global Impression.^19^

Secondary outcomes were also examined including (i) dropout due to any reason, as a means of global assessment of acceptability (dichotomous); (ii) total number of patients with adverse effects, (dichotomous). Other psychiatric outcomes include: (iii) positive symptoms (continuous); (iv) negative symptoms (continuous); (v) depressive symptoms (continuous); (vi) quality of life (continuous); (vii) social functioning (continuous). Other specific adverse event measures included (viii) use of antiparkinsonian medication (dichotomous); (ix) weight gain (kg, continuous); (x) sedation (dichotomous); (xi) prolactin levels (ng/mL, continuous); (xii) QTc prolongation (ms, continuous); (xiii) death (dichotomous).

We prioritized the change score over endpoint score, and methods accounting for missing outcome data (e.g., mixed-models of repeated measurement [MMRM], multiple imputations) over last-observation carried forward (LOCF) and over observed cases. Missing standard deviations (SD) were imputed from test statistics, by contacting study authors, or from SDs of other included studies using a validated imputation method.^20^ We used the standardized mean difference (SMD), the difference in mean change between treatment groups divided by the pooled within-study standard deviation using Hedge's g, for continuous outcomes, but we used the mean difference (MD) for continuous outcomes when natural units make sense, such as kg for weight. We used the odds ratio (OR) for dichotomous outcomes and the number of participants randomized as the denominator for dichotomous outcomes. We assumed that those lost to follow-up had not responded to the treatment but not developed side effects.

### Search strategies

We searched the Cochrane Schizophrenia Group Register^21^ from inception until January 13, 2025, and PubMed from January 1, 2025 to January 8, 2026. eAppendix 2 presents more details. We excluded studies from mainland China, and did a sensitivity analysis excluding studies from other countries with less clinical research experience due to quality issues in many of these studies.^22^^,^^23^

### Data extraction and risk of bias assessment

Two reviewers (pairs of YF, NHS, YW, EP, JS, DDK, FM, MS and IB) independently selected trials, extracted data, evaluated the risk of bias of individual studies using the revised Cochrane Risk of Bias assessment tool for the primary outcome.^24^ We resolved any disagreement through discussion. If disagreements remained unresolved through discussion, the third senior reviewers (JS and IB) were approached to reach a consensus. We contacted authors of the included studies for further clarification, if needed.

### Data analysis

We created a network diagram to visualize the available evidence. We examined the transitivity assumption, which is the basic assumption behind NMA.^12^ It implies that all the eligible participants can be randomized to any of the interventions, and that the effect modifiers are equally distributed among the arms. We created box plots of trial and participant characteristics deemed to be potential effect modifiers (such as age, sex, baseline severity of symptoms, duration of illness, definition of treatment-resistance, treatment duration, blinding status, publication year, sample size) and visually examined whether they were similarly distributed across treatment comparisons. If transitivity does not hold, we may see inconsistencies in the network. We checked consistency using global (design-by-treatment) and local (back-calculation) tests.^25^^,^^26^

We then conducted frequentist NMA. Given the expected clinical and methodological heterogeneity of treatment effects among the studies, we used the random-effects model. Multi-arm trials were appropriately handled by accounting for the correlation between effect estimates from the same study. The variance structure was specified using a common between-study heterogeneity variance across the network, assuming a common τ^2^ for all comparisons. We visualized the NMA results using continuation of the antipsychotic monotherapy already used as the reference, and ordering interventions according to the P score.^27^ We summarized the primary and secondary outcomes using the Kilim plot.^28^ We assessed heterogeneity by comparing the estimated τ^2^ with empirical distributions.^29^

We conducted a subgroup analysis to test the effect of different grades of treatment-resistance. We performed several sensitivity analyses to confirm the robustness of the primary analysis: focusing on double-blind trials, excluding overall high risk of bias trials, excluding trials without operationalized diagnostic criteria, focusing on clozapine-resistant patients. We additionally conducted post-hoc sensitivity analyses excluding studies from countries with less research experience and excluding a baseline severity outlier. Post hoc subgroup analyses were performed to examine the effect of each combination strategies. We performed a *post hoc* component network meta-analysis to mitigate the potential effect of different control conditions.^30^

We assessed the presence of small study effects, including publication bias, by examining asymmetry in the contour-enhanced funnel plots of comparisons with more than 10 trials. We assessed the confidence in the evidence using the Confidence In Network Meta-Analysis (CINeMA) framework, considering the following 6 domains: within-study bias, reporting bias, indirectness, imprecision, heterogeneity and incoherence.^31^

We performed all analyses in R using the *meta* package (ver. 7.0.0) and the *netmeta* package (ver. 2.9.0). This study was conducted from November 2024 to January 2026.

### Role of funding source

The funders had no role in study design, data collection and analysis, decision to publish, or preparation of the manuscript.

## Results

**Fig. 1:**
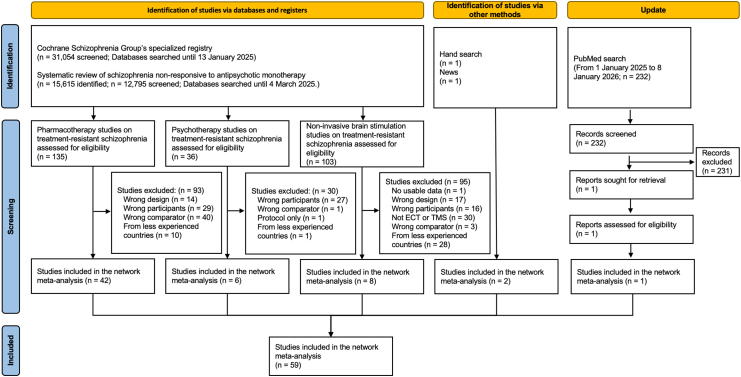
**Study selection**.

We screened 44,081 records and identified 59 trials with 5409 participants randomized (Fig. 1, eAppendix 2). We excluded some studies included in our previous meta-analyses of antipsychotics for TRS,^4^, ^5^, ^6^^,^^11^ because some compared a non-clozapine to another non-clozapine (e.g., Altamura et al., 2002),^32^ and some did not fulfill our non-response definition (e.g., Goff et al., 2013).^33^ The main reason for excluding studies for CBTp from our previous meta-analysis^7^ was not fulfilling our non-response criteria (e.g., Barrowclough et al., 2006).^34^ The main reason for excluding studies for NIBS from our ongoing meta-analysis^35^ was because they were from mainland China (eAppendix 2). The primary analysis included 55 trials with 5013 participants. Typical participants were males in their thirties to forties (1441 of 4857 of those whose sex was reported were females [29.7%], mean age 39.8 [range, 29.1–66.6]. Ethnicity data not available). Most studies used operationalized diagnostic criteria (54 of 59, 92%). The minimum number of antipsychotic trials required was 2 on average (range, 1–3). Fourteen trials out of 54 had the minimum number of antipsychotic trials of one; twelve studies included participants with two or more antipsychotic trial failures, and the only exception was the one on xanomeline-trospium augmentation, which excluded participants with two or more adequate antipsychotic trials.^36^ The minimum duration of adequate antipsychotic trials was 6 weeks on average (range, 4–50). The antipsychotic dosage was 500 mg chlorpromazine equivalent or more in about half of the trials (30 studies), while 20 studies just stated “adequate”. The base antipsychotic was clozapine in 22 studies, second generation antipsychotics were used in more than 80% of participants in 10 studies, first generation antipsychotics were used in more than 80% of the participants in 8 studies. Most studies judged treatment-resistance based on retrospective evaluation, while 5 studies involved prospective trials to confirm treatment-resistance. The overall risk of bias was low in 13 of the trials (22%), some concerns in 27 (50%) and high in 15 (28%) (eAppendix 2).

**Fig. 2:**
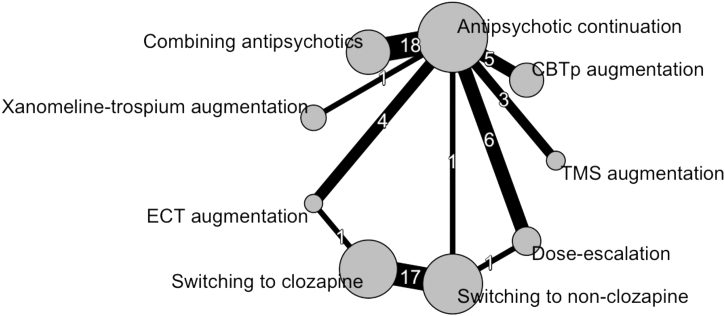
**The network diagram for the primary outcome**. The width of lines connecting treatments corresponds to the number of trials performing the corresponding comparison. This number is also given on each line. Absence of a line connecting 2 treatments means that this comparison was not performed in any study. The size of nodes corresponds to the number of participants randomized to the intervention.

The network for the primary outcome had three closed loops and four nodes were connected only to the reference node (Fig. 2).

The mean age and the proportion of females were evenly distributed among the comparisons. However, the baseline severity measured by PANSS was higher among the ECT vs switching to clozapine comparison. Blinding was mostly double-blind for pharmacotherapies, whereas it was mixed for NIBS and single-blind for CBTp, as expected. The trial duration was longest for CBTp (mean duration, 31 weeks [range, 21–39]). (eAppendix 3). We ran a post-hoc sensitivity analysis excluding the outlier comparisons in the baseline severity (ECT vs switching to clozapine).

Two comparisons (antipsychotic combination vs antipsychotic continuation, switching to clozapine vs switching to non-clozapine) had more than 10 studies. Visual inspection of the funnel plots did not suggest reporting bias (eAppendix 4).

**Fig. 3:**
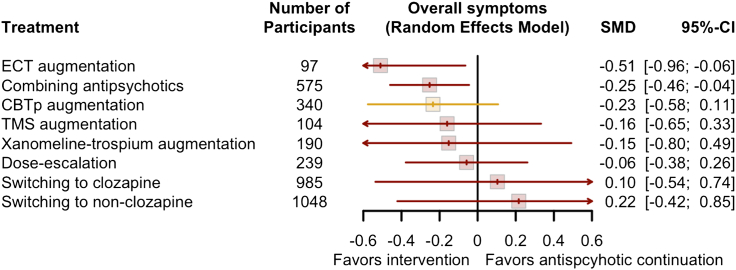
**The result of network meta-analysis**. CBTp, cognitive behavioral therapy for psychosis; CI, confidence interval; ECT, electroconvulsive therapy; k, number of arms; n, number of participants; SMD, standardized mean difference; TMS, transcranial magnetic stimulation. Yellow indicates low confidence in the evidence, and red very low according to the Confidence In Network Meta-Analysis framework.

**Fig. 4:**
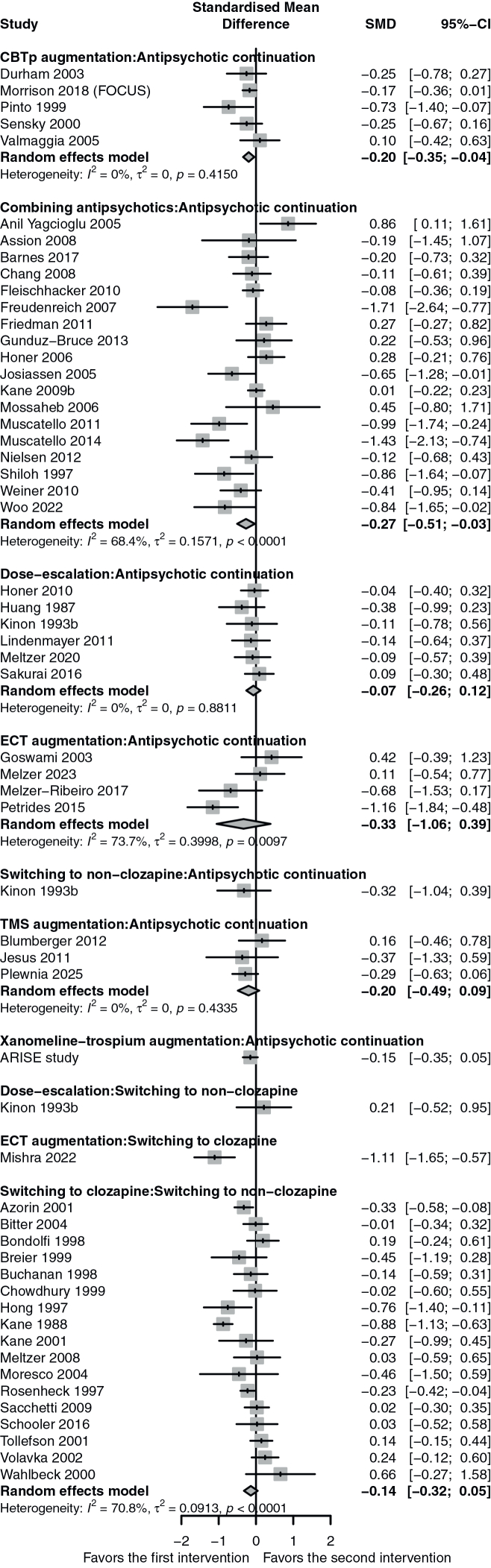
**Pairwise meta-analyses**.

**Fig. 5:**
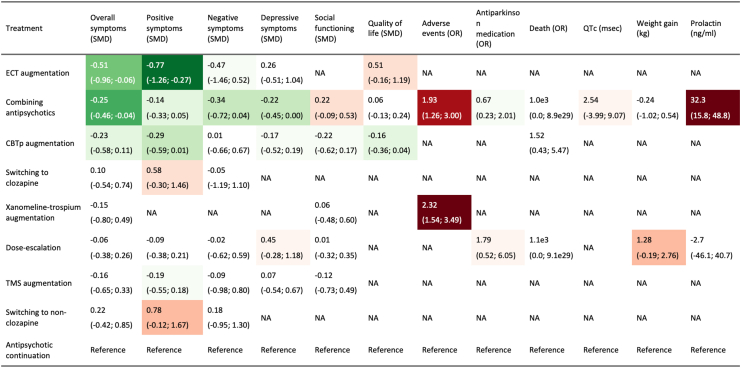
**The primary and secondary outcomes compared to the continuation of the same antipsychotic**. CBTp, cognitive behavioral therapy for psychosis; CI, confidence interval; ECT, electroconvulsive therapy; OR, odds ratio; SMD, standardized mean difference; TMS, transcranial magnetic stimulation. Darker colors correspond to smaller p-values. Green indicates better outcomes compared to the reference, whereas red indicated worse. When the network consists of several subnetworks, only those that can be compared to the reference are shown.

Fig. 3 shows the result of network meta-analysis, Fig. 4 pairwise meta-analyses, and Fig. 5 show the results all the outcomes. eAppendix 5 compared the direct and indirect evidence, and eAppendix 6 shows the league table. eAppendix 7 shows the evaluation of confidence according to CINeMA.

The evidence for all comparisons was of low to very low confidence according to CINeMA. We found that combining antipsychotics and ECT augmentation might be efficacious, albeit their very low confidence in the evidence. CBTp augmentation had wide confidence interval, but pairwise meta-analysis showed a narrower confidence interval with SMD of −0.20 (95% CI, −0.35; −0.04) due to lower heterogeneity among the direct comparison. Xanomeline-trospium augmentation, dose-escalation, TMS augmentation, switching to clozapine, and switching to non-clozapine had wide confidence intervals and the results were inconclusive.

eAppendix 3 presents the statistical assessments of the transitivity assumption. The global (design-by-treatment) test did not show evidence of inconsistency (p = 0.17). The local (back-calculation) method suggested some evidence of disagreement between direct and indirect comparisons (p = 0.06 for 4 comparisons out of 6 comparisons assessed). Net splitting showed a very wide confidence interval for the indirect estimate and the network estimate did not martially change from the direct estimate for 3 comparisons out of the 4 comparisons suggestive of disagreement (dose-escalation vs antipsychotic continuation, ECT augmentation vs antipsychotic augmentation, ECT augmentation vs switching to clozapine, switching to clozapine vs switching to non-clozapine; eAppendix 5). Multiple sensitivity analyses with one triangle loop at most did not show clear evidence of disagreement. The proportion of variation attributable to between-study heterogeneity was substantial (I^2^ = 64.3% [51.6%; 73.6%]) and the τ^2^ was within the empirically expected range (τ^2^ = 0.10; τ = 0.31).

Fig. 5 summarizes the primary and secondary outcomes. There was no evidence of differences in the all-cause dropout, suggesting good acceptability of all the interventions compared to antipsychotic continuation. Positive symptoms decreased with ECT augmentation (n = 82; SMD, −0.77 [95% CI, −1.26; −0.27]), and with lesser certainty, with CBTp augmentation (n = 308; SMD, −0.29 [95% CI, −0.59; 0.01]) and antipsychotic combination (n = 526; SMD, −0.14 [95% CI, −0.33; 0.05]) over continuation of the same antipsychotic. Combining antipsychotics might decrease negative symptoms (n = 558; SMD, −0.34 [95% CI, −0.72; 0.04]) and depressive symptoms (n = 339; SMD, −0.22 [95% CI, −0.45; 0.00]), but may result in increased adverse events (n = 190; OR, 1.93 [95% CI, 1.26; 3.00]), including prolactin increase (n = 274; MD, 32.3 ng/ml [95% CI, 15.8; 48.8]) compared to continuation of the same antipsychotic (eAppendix 7).

**Fig. 6:**
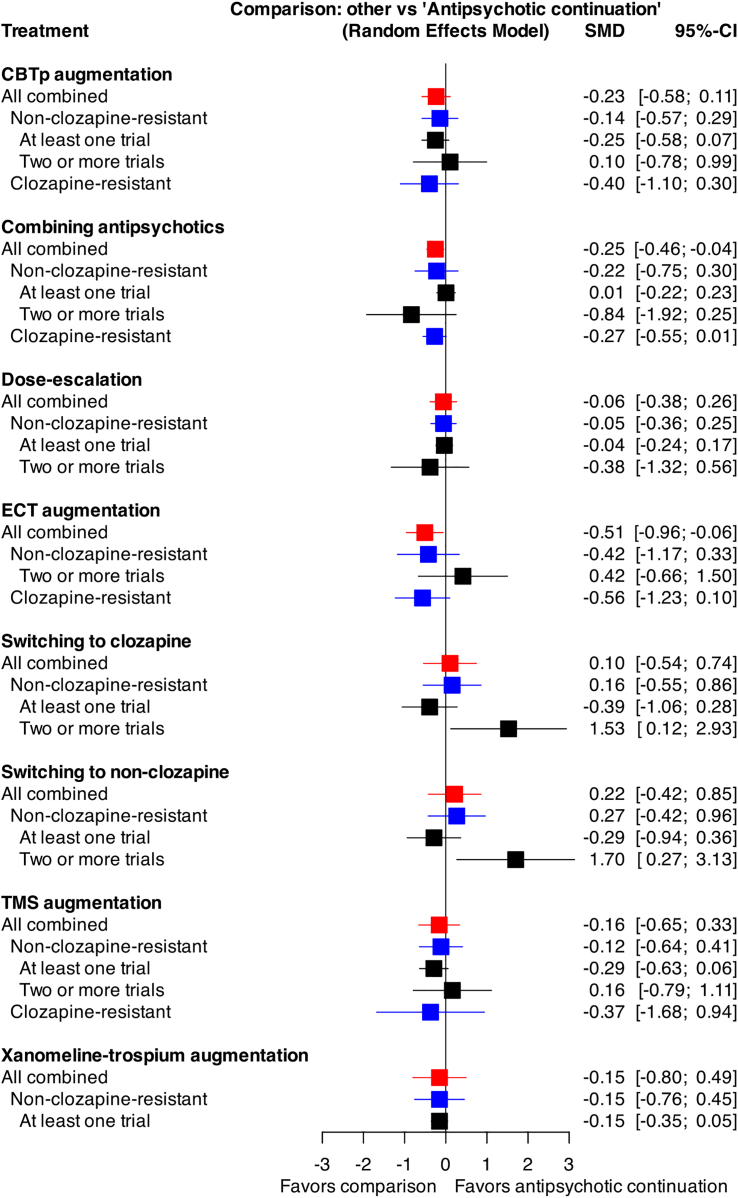
**Treatment-resistance stage-based subgroup analysis**. Red indicates results for all patients combined; Blue compares non–clozapine-resistant and clozapine resistant patients; Black compares patients with at least one antipsychotic trial versus those with two or more trials (within the non-clozapine-resistant population). CBTp, cognitive behavioral therapy for psychosis; CI, confidence interval; ECT, electroconvulsive therapy; SMD, standardized mean difference; TMS, transcranial magnetic stimulation.

Fig. 6 shows the result of subgroup analysis based on treatment-resistant staging. There was no clear evidence of modifying effect of treatment-resistant staging on the relative effects of the interventions. This is in line with our previous finding that the effects of antipsychotics in various subgroups were generally similar to those in the general population of people with schizophrenia.^13^ The large discrepancies observed in patients with one or more antipsychotic trials vs those with two or more trials are likely due to the sparse network structure, where switching to non-clozapine was compared with antipsychotic continuation only through limited indirect pathways, rather than reflecting true differences in treatment effects. eAppendix 9 summarizes the sensitivity analyses. In general, the results were robust among the different sensitivity analyses, except ECT augmentation. Combination trials showed high degree of heterogeneity (95% Prediction Interval, −1.30 to 0.76), but each combination was examined by only a few trials at most and there was no clear evidence of superiority of certain combinations (eAppendix 9).

A *post hoc*, exploratory component network meta-analysis was in line with the primary analysis (eAppendix 9).

## Discussion

To the best of our knowledge, this is the first network meta-analysis comparing different treatment modalities for non-response in schizophrenia. This study showed that combining D2R-targeting antipsychotics and ECT augmentation might be more efficacious than the continuation of antipsychotic, with very low confidence in the evidence according to CINeMA. CBTp augmentation might be potentially beneficial. We found no clear evidence of superiority of other treatment strategies (dose escalation, switching to non-clozapine, switching to clozapine, TMS augmentation, and xanomeline-trospium augmentation) over antipsychotic continuation.

Combining D2R-targeting antipsychotics was found to be more efficacious than continuing the same antipsychotic, but with more adverse events. A sensitivity analysis showed similar efficacy for non-clozapine-non-responsive schizophrenia (SMD, −0.23 [95% CI, −0.58; 0.29])) and clozapine-non-responsive schizophrenia (SMD, −0.27 [95% CI, −0.55; 0.01]). (eAppendix 8) A post hoc subgroup pairwise meta-analysis of combination trials did not find evidence of subgroup difference between clozapine-non-responsive- and non-clozapine-non-responsive schizophrenia (test for subgroup difference: p = 0.94). Most of the combinations were examined in three or less trials, and it was unclear which combinations were superior. (eAppendix 9). A previous meta-analysis showed the lack of double-blind/high-quality evidence of antipsychotic combination,^37^ but our sensitivity analyses focusing on double-blind studies and excluding high risk of bias studies aligned with the primary analysis. Large scale observational studies also found that antipsychotic combination was associated with lower risk of psychiatric rehospitalization^38^ and non-psychiatric hospitalization^39^ among people with schizophrenia. Although the combination strategy seems generally acceptable in terms of the all-cause dropout, cautions need to be taken for adverse events. In this analysis, we found the prolactin level increase, which could be due to the nature of the combination drugs examined. Most of the antipsychotics were risperidone, amisulpride and haloperidol, which are known to be associated with increased prolactin level.^40^ The two studies^41^^,^^42^ using aripiprazole did not increase the serum prolactin level. Which combinations work better needs to be further investigated.

ECT augmentation might be more efficacious than continuing the same antipsychotic, especially for positive symptoms, but the results need to be carefully interpreted. First, the NMA model assumed a common heterogeneity (τ^2^ = 0.10) among the network, which was substantially smaller than the heterogeneity observed in the pairwise meta-analysis (τ^2^ = 0.40), which may have led to an unreliably high precision. Second, it should be also noted that ECT augmentation was examined only by a small number of studies and participants (less than 100 participants in total). Meta-epidemiological studies have shown that small studies tend to show larger effect sizes than larger studies,^43^ and that the results of meta-analyses can fluctuate until a certain number of randomized subjects are included.^44^ Last but not least, the positive finding was driven largely by a trial without sham stimulation,^45^ while ECT did not show clear benefit compared to sham stimulation, which is in line with another meta-analysis of ours on NIBS for TRS with a broader TRS definition^8^ Our *post hoc* component network meta-analysis also showed similar efficacy between ECT and sham ECT (−1.33 [−1.97; −0.70] and −1.31 [−2.12; −0.51], respectively). We still need more trials before we can reach a definitive conclusion.

The direction of the result and the interpretation is similar to the effects of ECT in another network meta-analysis on clozapine-resistant schizophrenia.^46^ However, their estimated effect size is too good to be true (SMD, −4.32 [95% CI, −5.43; −3.21]), presumably because the standard deviation and the standard error were mistakenly handled.

For TMS, we found only three trials and only 104 participants were allocated to the intervention. We found more trials on TMS for schizophrenia,^8^ but many were from mainland China, and did not meet our strict treatment-resistant criteria or trial duration criteria.

CBTp has shown a promise as an effective intervention for reducing overall symptoms of individuals with TRS. The result was almost identical to our previous network meta-analysis of psychological and psychosocial interventions for treatment-resistant schizophrenia with a broader definition of treatment-resistance, which found that CBTp had the strongest evidence base.^7^ We used a broader definition of treatment-resistance for the previous study to maximize the number of eligible studies. On the other hand, we used more stringent criteria for this current study to ascertain the transitivity of the network, because we included different modalities of interventions. The 95% CI of the NMA result included null, but that of the pairwise meta-analysis result did not (SMD, −0.20 [95% CI, −0.35; −0.04]) (eAppendix 5). This is because the NMA result was influenced by the high heterogeneity among other studies, while CBTp studies themselves had low heterogeneity. Current study suggests that CBTp may also be beneficial to more strictly defined treatment-resistant patients. Baseline characteristics did not suggest any meaningful difference to the population allocated to other interventions, such as combining antipsychotics and non-invasive brain stimulation augmentation. However, CBTp was only examined against antipsychotic continuation and further trials comparing CBTp to other active interventions are needed.

Switching to clozapine failed to show superiority over continuing the same antipsychotic, switching to another non-clozapine antipsychotic, or any other treatment strategy. This is in contrast with the large-scale observational studies,^47^ but in line with our previous network meta-analysis of antipsychotic monotherapy on treatment-resistant schizophrenia^11^ and individual-participant-data meta-analysis^48^ of randomized controlled trials on treatment-resistant schizophrenia. Although around 1000 participants were allocated to each intervention, there was no clear evidence of efficacy difference among switching to clozapine and switching to non-clozapine. No trial examined switching to clozapine directly to antipsychotic continuation, while most trials examined switching to clozapine against switching to another antipsychotic. Thus, the evidence compared to antipsychotic continuation was derived only from indirect evidence. The first trial^49^ of switching to clozapine vs switching to another antipsychotic (chlorpromazine, in this case) on TRS showed a significant result (SMD, −0.88. 95% CI, −1.13; −0.63), but it was the largest effect size found among the 17 trials. The result of pairwise meta-analysis was SMD −0.14 (95% CI, −0.32; 0.05) compared to switching to another antipsychotic. (eAppendix 5) Only one trial^50^ examined switching to clozapine vs ECT augmentation. Trials that examine switching to clozapine against other candidates (i.e., ECT augmentations, combining antipsychotics and CBTp augmentation). are warranted, and the clinical practice guidelines need to acknowledge the limitation of the evidence base of clozapine for schizophrenia non-responsive to other antipsychotics.

Xanomeline-trospium is the first non-primarily-dopaminergic medication approved as monotherapy for schizophrenia.^17^ It is hoped that it leads to symptom improvement via different mechanism of action acting as muscarinic M1/M4 receptor agonist. However, the first augmentation trial with people with schizophrenia non-responsive to one antipsychotic trial^51^ did not show clear benefit in general, but a post-hoc subgroup analysis suggested benefit among those treated previously with non-risperidone antipsychotics. Further studies are needed to determine where it fits within the treatment framework.

There was no clear evidence of beneficial effect of dose-escalation. This is in line with a previous meta-analysis,^5^ and the dose–response meta-analysis, which showed no additional benefit above 5 mg risperidone equivalents.^52^^,^^53^ Given the small point estimate and the relatively narrow confidence interval, dose-escalation beyond a certain dosage may not be so promising.

Given the important findings above, our study has several limitations. First, there was substantial conceptual heterogeneity in the network. The definition for treatment non-response was more lenient than the current consensus for treatment-resistance. We therefore examined the patient characteristics and inconsistency extensively. We did not find evidence of difference among different stage of non-response. The included interventions’ modalities varied (pharmacotherapies, non-invasive brain stimulations and psychotherapy). The dosage ranges within the same pharmacological agents vary widely. For example, Honer 2006^54^ used 3 mg of risperidone in addition to clozapine, while Schooler 2016^55^ used 6–16 mg of risperidone as a stand-alone treatment. Dose–response meta-analyses suggested antipsychotic efficacy is unlikely to increase beyond 3–5 mg risperidone equivalent,^52^ but side effects may increase.^56^, ^57^, ^58^, ^59^ Therefore, the results need to be interpreted with caution, especially those related to side-effects. Switching to clozapine strategies adopted flexible dosing with greatly overlapping dose ranges. One trial had lower dose actually delivered (mean 120 mg/day), likely attributable to the advanced age of the participants (mean age 66 years).^60^ A sensitivity analysis excluding this trial did not materially change the results. Despite efforts to minimize heterogeneity in control conditions within each treatment modality—by restricting inclusion to rater-blinded studies,^61^ excluding waiting-list controls,^62^ omitting trials from mainland China,^63^ and confirming the absence of evidence for clinically meaningful differences between control conditions^7^^,^^64^ —differences in control designs across pharmacological, psychotherapeutic, and brain stimulation interventions remain a limitation. In particular, indirect comparisons between interventions of different modalities remain uncertain. Future multi-arm randomized trials directly comparing active interventions especially across modalities using harmonized control conditions are therefore needed to provide more robust and clinically informative evidence.

Second, the network was star-shaped, and there were very few head-to-head trials. Some interventions (antipsychotic combination, CBTp augmentation, TMS augmentation and xanomeline-trospium augmentation) were compared only to the antipsychotic continuation, and switching to clozapine was not directly compared to antipsychotic continuation. Many of comparisons across active interventions were therefore based solely on indirect evidence, and should be interpreted as preliminary, rather than definitive. Although we did not find any clear evidence of inconsistency in the network, it may have been masked due to high heterogeneity, or due to the nature of the starshaped network. More studies comparing promising treatment strategies to each other are warranted.

Third, some of the interventions had very few allocated participants. A meta-epidemiological study indicates that the estimated effect sizes were significantly larger in smaller trials.^43^ We therefore took into careful consideration the numbers of trials and participants when interpreting the results.

Forth, we did not involve people with lived experience of schizophrenia throughout the research process and did not analyze sex- or gender-based analyses of the primary outcome overall symptoms.

The strengths of our study are the comprehensiveness and the rigorous methodology. First, we carried out extensive literature searches across multiple databases and covered all the interventions with different modalities that are at least partially recommended by key clinical practice guidelines for non-response in general. Second, we followed the best practice of evidence synthesis. To ensure the transitivity assumption, we applied a strict non-response definition and carried out multiple examinations to test it. Our study is in sharp contrast with a network meta-analysis that suggested effectiveness of 16 augmentation agents,^65^ which excluded trials with moderate to high risk of bias. This practice is not recommended due to the potential bias during the review process.^66^ Previous network meta-analyses recommended interventions that were examined in only one or two small trials.^46^^,^^65^ We also emphasized the importance of the number of studies and participants examined for an intervention, and carefully interpreted the results.

Non-response to antipsychotics is a frequent and clinically important topic but our review found a lack of robust evidence to guide decisions. More head-to-head trials are needed before reaching a conclusion. Notably, we found no evidence to support the common practice of dose-escalation, or the guideline-recommended strategy of switching to clozapine. Our review found very weak evidence that D2R-targeting antipsychotic combination and ECT augmentation might be considered in case of non-response, though uncertainty remained. CBTp might also be considered.

## Contributors

**Yuki Furukawa:** Conceptualization, Methodology, Investigation, Formal analysis, Writing–Original Draft, Project administration.

**Nurul Husna Salahuddin:** Conceptualization, Investigation, Writing–Review & Editing.

**Yaohui Wei:** Conceptualization, Investigation, Review & Editing.

**Elisavet Pinioti:** Investigation, Review & Editing.

**David D. Kim:** Investigation, Review & Editing.

**Filip Milosavlijević:** Investigation, Review & Editing.

**Spyridon Siafis:** Conceptualization, Methodology, Review & Editing.

**Johannes Schneider-Thoma:** Conceptualization, Methodology, Review & Editing.

**Myrto Samara:** Conceptualization, Investigation, Review & Editing.

**Irene Bighelli:** Conceptualization, Methodology, Review & Editing.

**Stefan Leucht:** Conceptualization, Methodology, Writing–Review & Editing, Supervision.

YF, NHS, YW, DDK, FM and SS accessed and verified the data.

## Data sharing statement

Codes and data are available on reasonable request from the corresponding author.

## Declaration of interests

In the last 3 years MS has received honoraria for advising/consulting and/or for lectures and/or for support for attending meetings/travel from Viatris and Richter.

In the last three years SL has received honoraria for advising/consulting and/or for lectures and/or for educational material from Angelini, Apsen, Bristol-Myers-Squibb, Boehringer-Ingelheim, Gedeon-Richter, Johnson and Johnson, Karuna, Kynexis, Mitsubishi, Neurotorium, NovoNordisk, Orionpharma, Otsuka, ROVI, SunPharma, TEVA. YF received a grant from SENSHIN Medical Research Foundation, NHS from DAAD fellowship, and YHW from CSC scholarship.

All other authors declare no competing interests.
