# Supplementary material for: Next-step treatment for schizophrenia non-responsive to antipsychotics: a systematic review and network meta-analysis
Source: eClinicalMedicine. 2026 May 22;96:103988. doi: 10.1016/j.eclinm.2026.103988 (PMC13223997; doi:10.1016/j.eclinm.2026.103988)
Supplement: Supplementary Material [file mmc1.docx]

**TITLE: Next-step treatment for schizophrenia non-responsive to antipsychotics: a systematic review and network meta-analysis**

Table of Contents

[1. PROTOCOL 3](#_Toc227234469)

[Amendments 10](#_Toc227234470)

[2. SCREENING PROCESS AND RESULTS 11](#_Toc227234471)

[List of excluded trials (examples) 13](#_Toc227234472)

[List of included trials 42](#_Toc227234473)

[Study and patient characteristics 49](#_Toc227234474)

[The revised Cochrane risk of bias for the primary outcome 67](#_Toc227234475)

[3. ASSESSMENT OF TRANSITIVITY 70](#_Toc227234476)

[Box plots 70](#_Toc227234477)

[Global (design-by-treatment) test 75](#_Toc227234478)

[Local (back-calculation) test 76](#_Toc227234479)

[Comparing the tau square against empirical distributions 77](#_Toc227234480)

[4. ASSESSMENT OF PUBLICATION BIAS AND SMALL STUDY EFFECTS 78](#_Toc227234481)

[5. DIRECT COMPARISONS FOR PRIMARY OUTCOME 79](#_Toc227234482)

[6. LEAGUE TABLE FOR THE PRIMARY OUTCOME 80](#_Toc227234483)

[7. CONFIDENCE IN NETWORK META-ANALYSIS (CINeMA) 81](#_Toc227234484)

[8. SECONDARY OUTCOMES 87](#_Toc227234485)

[(i) clinically relevant response to treatment as defined by the trials (dichotomous) 87](#_Toc227234486)

[(ii) dropout due to any reason, as a means of global assessment of acceptability (dichotomous) 87](#_Toc227234487)

[(iii) total number of patients with adverse effects, as a proxy measurement of tolerability (dichotomous) 88](#_Toc227234488)

[(iv) positive symptoms (continuous) 89](#_Toc227234489)

[(v) negative symptoms (continuous) 89](#_Toc227234490)

[(vi) depressive symptoms (continuous) 91](#_Toc227234491)

[(vii) quality of life (continuous) 92](#_Toc227234492)

[(viii) social functioning (continuous) 93](#_Toc227234493)

[(ix) use of antiparkinsonian medication (dichotomous) 94](#_Toc227234494)

[(x) weight gain (kg, continuous) 95](#_Toc227234495)

[(xi) sedation (dichotomous) 96](#_Toc227234496)

[(xii) prolactin levels (ng/mL, continuous) 97](#_Toc227234497)

[(xiii) QTc prolongation (ms, continuous) 98](#_Toc227234498)

[(xiv) death (dichotomous) 99](#_Toc227234499)

[9. SENSITIVITY ANALYSES 100](#_Toc227234500)

[S1. Focusing on double-blind trials 101](#_Toc227234501)

[S2. Excluding high risk of bias trials 102](#_Toc227234502)

[S3. Excluding trials without operationalized diagnostic criteria 103](#_Toc227234503)

[S4.1. Focusing on clozapine-resistant patients 104](#_Toc227234504)

[S4.2. Focusing on TRRIP-criteria 105](#_Toc227234505)

[S6. Excluding baseline severity outlier (Mishra 2022) 107](#_Toc227234506)

[S7. Subgroup analyses of combination trials (post hoc) 108](#_Toc227234507)

[S8. Excluding Salganik 1998 (only triak on the elder population) (post hoc) 111](#_Toc227234508)

[S9. Component Network Meta-Analysis (*post hoc*) 112](#_Toc227234509)

# 1. PROTOCOL

First draft: 29 November 2024

Last updated: 8 January 2025

**Pharmacological, psychological and non-invasive brain stimulation treatments for antipsychotic resistant schizophrenia:**

**a protocol for a systematic review and network meta-analysis**

Yuki Furukawa^1,2^, Nurul Husna Salahuddin^1^, Yaohui Wei^1^, Elisavet Pinioti^3^, Spyridon Siafis^1^, Johannes Schneider-Thoma^1^, Myrto Samara^3^, Irene Bighelli^1^, Stefan Leucht^1^

1. Department of Psychiatry and Psychotherapy, School of Medicine, Technical University of Munich, Munich, Germany; 2. Department of Neuropsychiatry, University of Tokyo, Tokyo, Japan; 3. Department of Psychiatry, University of Thessaly, Greece

Contact: [yuki.furukawa@tum.de](mailto:yuki.furukawa@tum.de)

**REVIEW QUESTION**

What are the comparative efficacy, acceptability and tolerability of pharmacological, psychological, and non-invasive brain stimulation treatments for antipsychotic-resistant schizophrenia?

**BACKGROUND**

Treatment resistance to antipsychotics, the first-line treatment of schizophrenia, is notably high: approximately two thirds do not achieve symptom remission([Samara et al., 2019](#bibRef0268c23fa453c4707ad7acf0a32fb3be8)). The body of evidence concerning treatment options for individuals with an inadequate response to antipsychotic monotherapy remains a subject of ongoing debate: dose-escalation is recommended in case of insufficient response, but recent dose-response meta-analyses do not support the additional benefit of dose increase beyond 5mg risperidone equivalents([Leucht et al., 2020](#bibRef0f17a4d2505a54c21a6c61d92b61cd1a8)); clinical practice guidelines recommend clozapine for treatment-resistant patients([Association, 2020](#bibRef0226948b0bdc944b3b23c9c8213f962c2)) , but a comprehensive network meta-analysis([Dong et al., 2024](#bibRef00e33908fb2034a3b9158619bcf0b9d25)) questioned its superiority over second-generation antipsychotics, especially olanzapine; combining several antipsychotics is a common practice, but the evidence base is uncertain([Correll et al., 2017](#bibRef0acbbca277d6143458f151c3f5ebe41bc)); non-pharmacological interventions, such as non-invasive brain stimulations([Sinclair et al., 2019](#bibRef0ceab98cf533248b8b6e210c6425708b8))  and psychosocial interventions([Salahuddin et al., 2024](#bibRef00b6f7db8576345aa95e3c3e7e0229293)) are promising but need more research ([Association, 2020](#bibRef0226948b0bdc944b3b23c9c8213f962c2)).

In this study, we will examine the comparative efficacy, acceptability and tolerability of pharmacological, psychological, and non-invasive brain stimulation treatments for antipsychotic-resistant schizophrenia.

**METHODS**

We will follow the Preferred Reporting Items for Systematic reviews and Meta-Analyses (PRISMA) guideline for network meta-analysis.([Page et al., 2021](#bibRef09238b0ac567d4711a908791681c29ba1)) The protocol will be prospectively registered in the Open Science Framework.

*Data sources*

We will search the study-based trial register of schizophrenia interventions, which is based on regular searches of multiple electronic databases, including AMED, BIOSIS, CENTRAL, CINAHL, ClinicalTrials.Gov, Embase, ISRCTN, MEDLINE, ProQuest Dissertations and Theses, PubMed, WHO ICTRP and hand searches ([Shokraneh and Adams, 2020](#bibRef05d5b875b01374cbb935bf22bc6dd4314)). We will inspect relevant systematic reviews to ensure that no eligible trials are missed. Studies from countries with quality concerns, such as mainland China, will be excluded due to quality issues in many of these studies([Tong et al., 2018](#bibRef04b653806990f46e0bd741c75a78bf636)). Studies conducted in China by international companies will be accepted.

*Study design*

We will include all randomized controlled trials that compared at least two of the interventions listed below, with at least single-blind design. We will exclude cluster randomized trials to mitigate the unit-of-analysis problem. In case of cross-over trials, we will use only the first phase to avoid the carry-over effects.

*Participants*

We will include studies of patients with treatment-resistant schizophrenia or related disorders (such as schizophreniform, or schizoaffective disorders). There will be no restrictions in terms of gender, ethnicity, age, or setting. We will include all the diagnostic criteria and test the effect of including studies without a formal diagnosis in a sensitivity analysis. We will accept trials if 80% or more participants had schizophrenia or related disorders, but all the participants need to be treatment resistant as defined below.

We defined antipsychotic-resistant patients as having clinically relevant symptoms after trying at least one antipsychotic with an adequate dose (as defined by the authors, or effective dose 50%([Leucht et al., 2020](#bibRef0f17a4d2505a54c21a6c61d92b61cd1a8)) , 50% of the defined daily dose([Leucht et al., 2016](#bibRef0720e881ff1ea498a9f1b73154ced6b59)) , or the minimum effective dose([Leucht et al., 2016](#bibRef0720e881ff1ea498a9f1b73154ced6b59)) ) for at least four weeks. We will exclude patients with predominantly negative symptoms. We will examine the possible effect modification effect of different treatment resistant definitions in a subgroup analysis.

*Interventions and controls*

We will include the following interventions that are recommended in major clinical practice guidelines (APA, NICE, Germany, Japan)([Association, 2020](#bibRef0226948b0bdc944b3b23c9c8213f962c2); [Hasan et al., 2020](#bibRef0b464e0287c7f4cf2b9447ecbd72ee4a3); [Neuropsychopharmacology, 2021](#bibRef02111a77bf6204929b1aa1b6b804b4309); [(NICE), 2014](#bibRef0e1825f41cf35409789572cc3e27349c7))  :

1. Continuation of the antipsychotic monotherapy [reference]
2. Dose-escalation of the antipsychotic used
3. Switching to another antipsychotic (other than clozapine) monotherapy
4. Switching to clozapine monotherapy
5. Combining two antipsychotics
6. Cognitive behavioral therapy for psychosis (CBTp)
7. Non-invasive brain stimulations
   1. Electroconvulsive therapy (ECT)
   2. Transcranial magnetic stimulation (TMS)

Continuation of the antipsychotic monotherapy includes those with inactive interventions, such as placebo drugs, sham stimulations, and psychological placebo.

Where multiple arms are reported in a single trial, we will include only the relevant arms.

*Outcomes*

Primary outcome is the overall schizophrenia symptoms (continuous). Secondary outcomes will include: (i) clinically relevant response to treatment as defined by the trials (dichotomous); (ii) dropout due to any reason, as a means of global assessment of acceptability (dichotomous); (iii) total number of patients with adverse effects, as a proxy measurement of tolerability (dichotomous). Other psychiatric outcomes will include: (iv) positive symptoms (continuous); (v) negative symptoms (continuous); (vi) depressive symptoms (continuous); (vii) quality of life (continuous); (viii) social functioning (continuous). Other specific adverse event measures will include; (ix) use of antiparkinsonian medication (dichotomous); (x) weight gain (kg, continuous); (xi) sedation (dichotomous); (xii) prolactin levels (ng/mL, continuous); (xiii) QTc prolongation (ms, continuous); (xiv) death (dichotomous).

For schizophrenia symptoms, we will prioritize the Positive and Negative Syndrome Scale. If not reported, we will use the following scales in this order: the Brief Psychiatric Rating Scale, and then any other validated scales. We will prioritize the change score over endpoint score, and methods accounting for missing outcome data (e.g., mixed-models of repeated measurement

(MMRM), multiple imputations) over last-observation carried forward (LOCF) and over observed cases. Missing standard deviations (SD) will be derived from test statistics, by contacting study authors, or from SDs of other included studies using a validated imputation method([Furukawa et al., 2006](#bibRef04bb4b83d43314b17b3ad7f2e1cd8f140)). We will use the standardized mean difference for continuous outcomes, but we will use the mean difference for continuous outcomes using a single scale. We will use odds ratio for dichotomous outcomes and the number of participants randomized as the denominator for dichotomous outcomes. We will assume that those lost to follow-up had not responded to the treatment but not developed side effects.

We will use the outcomes measured at the end of the interventions, but the minimum duration was set at three weeks.

*Risk of bias assessment*

Two independent reviewers will evaluate the risk of bias of individual studies using the revised Cochrane Risk of Bias assessment tool ([Sterne et al., 2019](#bibRef067df603fced744d58d0c7858017ca889))

**Statistical analysis**

*Network meta-analyses*

We will create a network diagram to visualize the available evidence. Transitivity assumption is the basic assumption behind NMA([Efthimiou et al., 2016](#bibRef0aef0f44af2f54518be98a44fa95cd281)). It implies that all the eligible participants are equally likely to be randomized to any of the interventions, and that the effect modifiers are equally distributed among the arms. To assess transitivity assumption, we will create box plots of trial and participant characteristics deemed to be potential effect modifiers (such as age, sex, baseline severity of symptoms, duration of illness, definition of treatment-resistance, treatment duration, blinding status, publication year, sample size) and visually examined whether they were similarly distributed across treatment comparisons. If transitivity does not hold, we may see inconsistencies in the network. We will check consistency using global (design-by-treatment) and local (back-calculation) tests([König et al., 2013](#bibRef01b2172ff39d9488ca52178f6fb668a46); [White et al., 2012](#bibRef070a4a76c18bc47f9863080f93e233878)) .

If transitivity assumption holds, we will conduct frequentist NMA. Given the expected clinical and methodological heterogeneity of treatment effects among the studies, we will use the random-effects model. We will visualize NMA results using continuation of the antipsychotic monotherapy already used as the reference, and ordering interventions according to P score([Rücker and Schwarzer, 2015](#bibRef09b22da2dbd45466b8452de8fa103e149)) .

We will assess heterogeneity by comparing the estimated τ^2^ with empirical distributions([Turner et al., 2015](#bibRef0c4bdf03362b940f6a1f94adcda5503ab)) .

*Subgroup analysis*

- To test the effect of treatment-resistant definition

*Sensitivity analyses*

- Focusing on double-blind trials
- Excluding high risk of bias trials
- Excluding trials without operationalized diagnostic criteria
- Focusing on clozapine-resistant patients

*Small study effects and reporting bias*

We will assess the presence of small study effects, including publication bias, by examining asymmetry in the contour-enhanced funnel plots of comparisons with more than 10 trials.

*Certainty of evidence*

We will assess the certainty of evidence using CINeMA([Nikolakopoulou et al., 2020](#bibRef0d85b9e459825482b8ae5ad027464a5a7)) .

We will perform all analyses in R using the *meta* package ([Balduzzi et al., 2019](#bibRef032ada8fff33f47ef93cf0ce143c18e6e)) and the *netmeta* package([Balduzzi et al., 2023](#bibRef0952f8393b8c8497a892823a09e21ce0c)) .

**Patient and public involvement**

There was no patient or public involvement in the development of this manuscript.

**Acknowledgements**

The views expressed are those of the authors and not necessarily those of affiliated organizations.

**Declaration of interests**

Y.F. has received consultancy fee from Panasonic and lecture fee from Otsuka outside the submitted work; In the past 3 years S.L. has received honoraria as an adviser, for lectures, or for educational material from Angelini, Boehringer Ingelheim, Apsen, Eisai, Ekademia, Gedeon Richter, Janssen, Karuna, Kynexis, Lundbeck, Medichem, Medscape, Mitshubishi, NovoNordisk, Otsuka, Recordati, Rovi, and TEVA. All other authors declare no competing interests.

**Data sharing**

Codes and data for all analyses will be available in the Open Science Framework.

**REFERENCE**

Association TAP. The American Psychiatric Association Practice Guideline for the Treatment of Patients With Schizophrenia. 2020. <https://doi.org/10.1176/appi.books.9780890424841>.

Balduzzi S, Rücker G, Nikolakopoulou A, Papakonstantinou T, Salanti G, Efthimiou O, et al. netmeta: An R Package for Network Meta-Analysis Using Frequentist Methods. J Stat Softw 2023;106:1–40. <https://doi.org/10.18637/jss.v106.i02>.

Balduzzi S, Rücker G, Schwarzer G. How to perform a meta-analysis with R: a practical tutorial. Évid Based Ment Heal 2019;22:153. <https://doi.org/10.1136/ebmental-2019-300117>.

Correll CU, Rubio JM, Inczedy-Farkas G, Birnbaum ML, Kane JM, Leucht S. Efficacy of 42 Pharmacologic Cotreatment Strategies Added to Antipsychotic Monotherapy in Schizophrenia: Systematic Overview and Quality Appraisal of the Meta-analytic Evidence. JAMA Psychiatry 2017;74:675. <https://doi.org/10.1001/jamapsychiatry.2017.0624>.

Dong S, Schneider-Thoma J, Bighelli I, Siafis S, Wang D, Burschinski A, et al. A network meta-analysis of efficacy, acceptability, and tolerability of antipsychotics in treatment-resistant schizophrenia. Eur Arch Psychiatry Clin Neurosci 2024;274:917–28. <https://doi.org/10.1007/s00406-023-01654-2>.

Efthimiou O, Debray TPA, Valkenhoef G, Trelle S, Panayidou K, Moons KGM, et al. GetReal in network meta‐analysis: a review of the methodology. Res Synth Methods 2016;7:236–63. <https://doi.org/10.1002/jrsm.1195>.

Furukawa TA, Barbui C, Cipriani A, Brambilla P, Watanabe N. Imputing missing standard deviations in meta-analyses can provide accurate results. J Clin Epidemiol 2006;59:7–10. <https://doi.org/10.1016/j.jclinepi.2005.06.006>.

Hasan A, Falkai P, Lehmann I, Janssen B, Wobrock T, Zielasek J, et al. Die aktualisierte S3-Leitlinie Schizophrenie. Nervenarzt 2020;91:26–33. <https://doi.org/10.1007/s00115-019-00813-y>.

König J, Krahn U, Binder H. Visualizing the flow of evidence in network meta‐analysis and characterizing mixed treatment comparisons. Statist Med 2013;32:5414–29. <https://doi.org/10.1002/sim.6001>.

Leucht S, Crippa A, Siafis S, Patel MX, Orsini N, Davis JM. Dose-Response Meta-Analysis of Antipsychotic Drugs for Acute Schizophrenia. Am J Psychiat 2020;177:342–53. <https://doi.org/10.1176/appi.ajp.2019.19010034>.

Leucht S, Samara M, Heres S, Davis JM. Dose Equivalents for Antipsychotic Drugs: The DDD Method. Schizophr Bull 2016;42:S90–4. <https://doi.org/10.1093/schbul/sbv167>.

Neuropsychopharmacology JS of. Japanese Society of Neuropsychopharmacology: “Guideline for Pharmacological Therapy of Schizophrenia.” Neuropsychopharmacol Rep 2021;41:266–324. <https://doi.org/10.1002/npr2.12193>.

(NICE) NI for H and CE. NICE clinical guideline 178 – Psychosis and schizophrenia in adults: treatment and management 2014. <https://guidance.nice.org.uk/cg178> (accessed September 27, 2024).

Nikolakopoulou A, Higgins JPT, Papakonstantinou T, Chaimani A, Giovane CD, Egger M, et al. CINeMA: An approach for assessing confidence in the results of a network meta-analysis. Plos Med 2020;17:e1003082. <https://doi.org/10.1371/journal.pmed.1003082>.

Page MJ, McKenzie JE, Bossuyt PM, Boutron I, Hoffmann TC, Mulrow CD, et al. The PRISMA 2020 statement: an updated guideline for reporting systematic reviews. Bmj 2021;372:n71. <https://doi.org/10.1136/bmj.n71>.

Rücker G, Schwarzer G. Ranking treatments in frequentist network meta-analysis works without resampling methods. Bmc Med Res Methodol 2015;15:58. <https://doi.org/10.1186/s12874-015-0060-8>.

Salahuddin NH, Schütz A, Pitschel-Walz G, Mayer SF, Chaimani A, Siafis S, et al. Psychological and psychosocial interventions for treatment-resistant schizophrenia: a systematic review and network meta-analysis. Lancet Psychiatry 2024;11:545–53. <https://doi.org/10.1016/s2215-0366(24)00136-6>.

Samara MT, Nikolakopoulou A, Salanti G, Leucht S. How Many Patients With Schizophrenia Do Not Respond to Antipsychotic Drugs in the Short Term? An Analysis Based on Individual Patient Data From Randomized Controlled Trials. Schizophr Bull 2019;45:639–46. <https://doi.org/10.1093/schbul/sby095>.

Shokraneh F, Adams CE. Cochrane Schizophrenia Group’s Study-Based Register of Randomized Controlled Trials: Development and Content Analysis. Schizophr Bull Open 2020;1:sgaa061. <https://doi.org/10.1093/schizbullopen/sgaa061>.

Sinclair DJ, Zhao S, Qi F, Nyakyoma K, Kwong JS, Adams CE. Electroconvulsive therapy for treatment‐resistant schizophrenia. Cochrane Database Syst Rev 2019;2019:CD011847. <https://doi.org/10.1002/14651858.cd011847.pub2>.

Sterne JA, Savović J, Page MJ, Elbers RG, Blencowe NS, Boutron I, et al. RoB 2: a revised tool for assessing risk of bias in randomised trials. Bmj 2019;366:l4898. <https://doi.org/10.1136/bmj.l4898>.

Tong Z, Li F, Ogawa Y, Watanabe N, Furukawa TA. Quality of randomized controlled trials of new generation antidepressants and antipsychotics identified in the China National Knowledge Infrastructure (CNKI): a literature and telephone interview study. BMC Méd Res Methodol 2018;18:96. <https://doi.org/10.1186/s12874-018-0554-2>.

Turner RM, Jackson D, Wei Y, Thompson SG, Higgins JPT. Predictive distributions for between‐study heterogeneity and simple methods for their application in Bayesian meta‐analysis. Statist Med 2015;34:984–98. <https://doi.org/10.1002/sim.6381>.

White IR, Barrett JK, Jackson D, Higgins JPT. Consistency and inconsistency in network meta‐analysis: model estimation using multivariate meta‐regression. Res Synth Methods 2012;3:111–25. <https://doi.org/10.1002/jrsm.1045>.

## Amendments

- We decided to focus on adults to reduce methodological heterogeneity.
- We decided to use CGI as the primary outcome, when neither PANSS total score or BPRS total score was available.
  - Levine SZ, Rabinowitz J, Engel R, Etschel E, Leucht S. Extrapolation between measures of symptom severity and change: an examination of the PANSS and CGI. Schizophr Res. 2008;98(1-3):318-322. doi:10.1016/j.schres.2007.09.006
- We found a xanomeline-trospium augmentation trial (ARISE study), which was eligible according to our inclusion criteria and clinically relevant. We decided to include it and added a new node (xanomeline-trospium augmentation).
- We decided to drop the response as a secondary outcome from the main manuscript, as it is redundant (another expression of PANSS/BPRS), but kept in the appendix.
- We changed the term treatment-resistant to non-response, based on feedback of the reviewers.

# 2. SCREENING PROCESS AND RESULTS


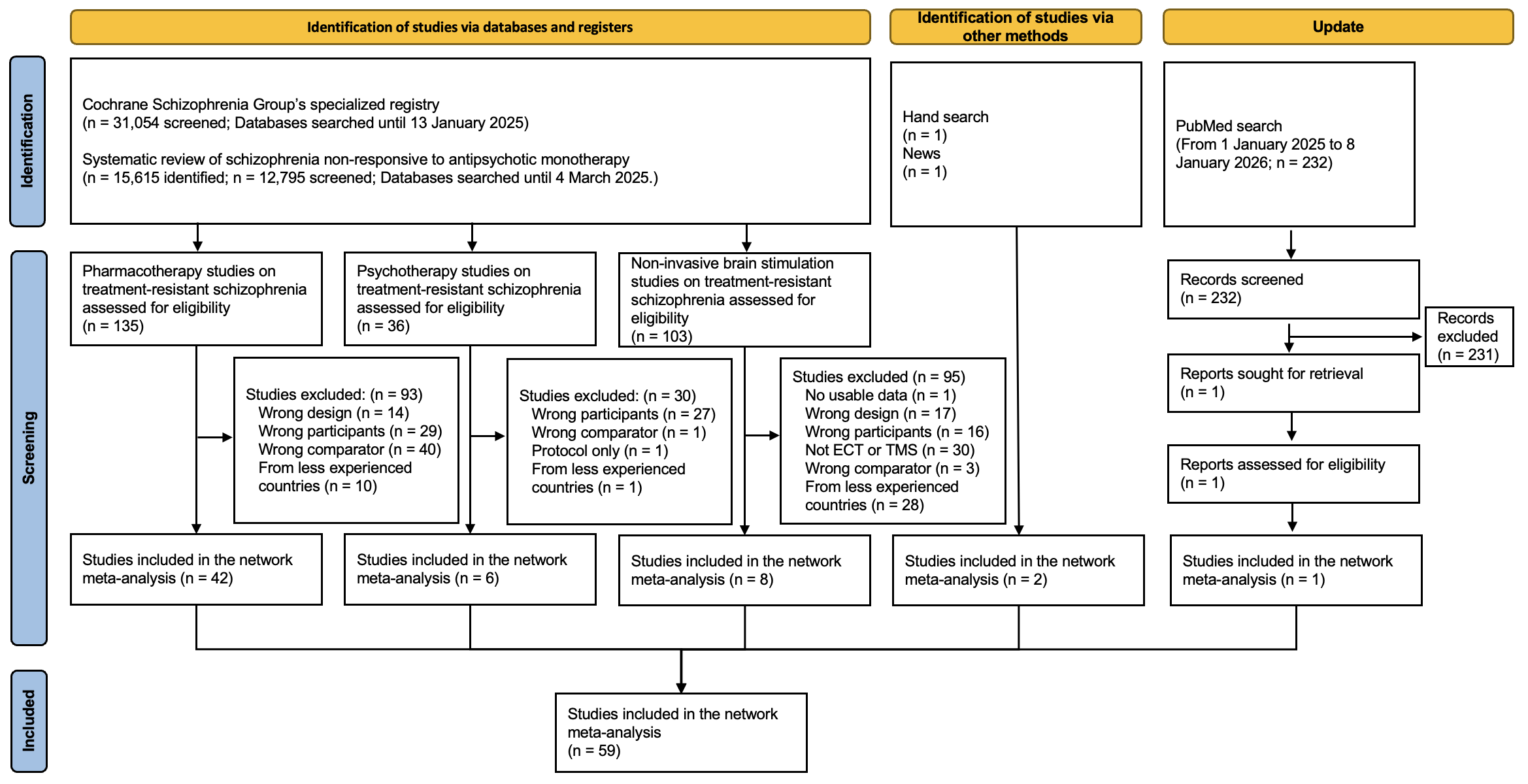


Detailed search strategies for the Cochrane Schizophrenia group’s Study-Based Register can be found in the Open Science Framework (<https://doi.org/10.17605/OSF.IO/U9R8Z>).

For pharmacotherapies, we also used references from our ongoing systematic review and meta-analysis on drug options in case of non-response in schizophrenia (<https://osf.io/4ajur>), where we searched MEDLINE, Cochrane Central Register of Controlled Trials (CENTRAL), Embase, PsycINFO, ClinicalTrials.gov, and the World Health Organization International Clinical Trials Registry Platform (ICTRP) until March 4, 2025.

**Search string used for the update search on PubMed**

("schizophrenia, treatment-resistant"[mh] OR ((Schizophrenia[mh] OR schizo*[tiab]) AND ("treatment resist*"[tiab] OR "resis*"[tiab] OR "refract*"[tiab] OR "no respon*"[tiab] OR "non-respons*"[tiab] OR "unrespons*"[tiab] OR "treatment fail*"[tiab] OR "not respon*"[tiab] OR "partially respon*"[tiab] OR "incomplete respon*"[tiab] OR "failed to respond"[tiab] OR "fail* therapy"[tiab] OR "failed to improve"[tiab] OR "difficult to treat"[tiab] OR "poorly respon*"[tiab] OR "non-remi*"[tiab] OR "partial remi*"[tiab] ))) AND (randomized controlled trial[pt] OR controlled clinical trial[pt] OR randomized[tiab] OR placebo[tiab] OR drug therapy[sh] OR randomly[tiab] OR trial[tiab] OR groups[tiab]) NOT (animals[mh] NOT humans[mh] ) AND (2025/01/01[EDAT]:3000/1/1[EDAT])

1. Shokraneh, Farhad; Adams, Clive E. Cochrane Schizophrenia Group’s Study-Based Register of Randomized Controlled Trials: Development and Content Analysis. Schizophrenia Bulletin Open 2020. https://doi.org/10.1093/schizbullopen/sgaa061.
2. Shokraneh, F., & Adams, C. E. (2024, November 25). Cochrane Schizophrenia Group’s Study-Based Register of Randomized Controlled Trials: Development and Content Analysis. https://doi.org/10.17605/OSF.IO/U9R8Z
3. Dong S, Schneider-Thoma J, Bighelli I, et al. A network meta-analysis of efficacy, acceptability, and tolerability of antipsychotics in treatment-resistant schizophrenia. *Eur Arch Psychiatry Clin Neurosci.* 2024;274(4):917-928. doi:10.1007/s00406-023-01654-2
4. Siafis S, Lorenz C, Wu H, et al. Non-invasive brain stimulation for treatment-resistant schizophrenia: protocol of a systematic review and network meta-analysis. *Syst Rev*. 2024;13(1):165. Published 2024 Jun 24. doi:10.1186/s13643-024-02585-2
5. Salahuddin NH, Schütz A, Pitschel-Walz G, et al. Psychological and psychosocial interventions for treatment-resistant schizophrenia: a systematic review and network meta-analysis. *Lancet Psychiatry*. 2024;11(7):545-553. doi:10.1016/S2215-0366(24)00136-6

## List of excluded trials (examples)

| **Category** | **Study_name** | **Reference** | **Reason_for_exclusion** |
| --- | --- | --- | --- |
| Pharmacological | Addington 1996 | Addington DE, Labelle A, Kulkarni J, Johnson G, Loebel A, Mandel FS. A comparison of ziprasidone and risperidone in the long-term treatment of schizophrenia: a 44-week, double-blind, continuation study. Can J Psychiatry. 2009;54(1):46-54. doi:10.1177/070674370905400108 | wrong C - other active comparators |
| Pharmacological | Ahlfors 1980 | Ahlfors UG, Dencker SJ, Gravem A, Remvig J. Clopenthixol decanoate and perphenazine enanthate in schizophrenic patients. A double-blind Nordic multicentre trial. Acta Psychiatr Scand Suppl. 1980;279:77-91. doi:10.1111/j.1600-0447.1980.tb07085.x | wrong P - dosage and/or duration not clearly stated |
| Pharmacological | Altamura 2002 | Altamura AC, Velonà I, Curreli R, Mundo E, Bravi D. Is olanzapine better than haloperidol in resistant schizophrenia? A double-blind study in partial responders. Int J Psychiatry Clin Pract. 2002;6(2):107-111. doi:10.1080/136515002753724117 | wrong C - other active comparators |
| Pharmacological | AstraZeneca 5077IL/0031 | A Multicenter, Double-Blind, Randomized, Comparison of Quetiapine (SEROQUEL®) and Chlorpromazine in the Treatment of Subjects with Treatment-Resistant Schizophrenia | wrong C - other active comparators |
| Pharmacological | AstraZeneca 5077IL/0054 | A Multicentre, Double-Blind, Randomised Trial to Compare the Effects of SEROQUEL and Chlorpromazine in Patients with Treatment Resistant Schizophrenia (5077IL/0054 [TRESS]) | wrong C - other active comparators |
| Pharmacological | Bjorndal 1980 | Bjørndal N, Bjerre M, Gerlach J, et al. High dosage haloperidol therapy in chronic schizophrenic patients: a double-blind study of clinical response, side effects, serum haloperidol, and serum prolactin. Psychopharmacology (Berl). 1980;67(1):17-23. doi:10.1007/BF00427590 | wrong P - dosage and/or duration not clearly stated |
| Pharmacological | Breier 1999a | Breier A, Hamilton SH. Comparative efficacy of olanzapine and haloperidol for patients with treatment-resistant schizophrenia. Biol Psychiatry. 1999;45(4):403-411. doi:10.1016/s0006-3223(98)00291-1 | wrong C - other active comparators |
| Pharmacological | Browne 1988 | Browne FW, Cooper SJ, Wilson R, King DJ. Serum haloperidol levels and clinical response in chronic, treatment-resistant schizophrenic patients. J Psychopharmacol. 1988;2(2):94-103. doi:10.1177/026988118800200204 | wrong C - vs placebo only |
| Pharmacological | Buchanan 1998_cognition | Buchanan RW, Breier A, Kirkpatrick B, Ball P, Carpenter WT Jr. Positive and negative symptom response to clozapine in schizophrenic patients with and without the deficit syndrome. Am J Psychiatry. 1998;155(6):751-760. doi:10.1176/ajp.155.6.751 | wrong design - subgroup analysis of an already included trial |
| Pharmacological | Buchanan 2005 | Buchanan RW, Ball MP, Weiner E, et al. Olanzapine treatment of residual positive and negative symptoms. Am J Psychiatry. 2005;162(1):124-129. doi:10.1176/appi.ajp.162.1.124 | wrong C - other active comparators |
| Pharmacological | Byerly 1999 | Byerly M, Weber M. Clozapine versus quetiapine for schizophrenia. Stanley Foundation Research Awards - 1999 Research Award Recipients | wrong design - protocol only |
| Pharmacological | Canuso 2010c | Canuso CM, Grinspan A, Kalali A, et al. Medication satisfaction in schizophrenia: a blinded-initiation study of paliperidone extended release in patients suboptimally responsive to risperidone. Int Clin Psychopharmacol. 2010;25(3):155-164. doi:10.1097/YIC.0b013e3283372977 | wrong design - less than 3 weeks |
| Pharmacological | Chen 2012 | Chen JJ, Chan HY, Chen CH, Gau SS, Hwu HG. Risperidone and olanzapine versus another first generation antipsychotic in patients with schizophrenia inadequately responsive to first generation antipsychotics. Pharmacopsychiatry. 2012;45(2):64-71. doi:10.1055/s-0031-1291293 | wrong C - other active comparators |
| Pharmacological | Cipriani 2013 | Cipriani A, Accordini S, Nosè M, et al. Aripiprazole versus haloperidol in combination with clozapine for treatment-resistant schizophrenia: a 12-month, randomized, naturalistic trial. J Clin Psychopharmacol. 2013;33(4):533-537. doi:10.1097/JCP.0b013e318296884f | wrong C - other active comparators |
| Pharmacological | Claus 1992 | Claus A, Bollen J, De Cuyper H, et al. Risperidone versus haloperidol in the treatment of chronic schizophrenic inpatients: a multicentre double-blind comparative study. Acta Psychiatr Scand. 1992;85(4):295-305. doi:10.1111/j.1600-0447.1992.tb01473.x | wrong C - other active comparators |
| Pharmacological | Conley 1998 | Conley RR, Tamminga CA, Bartko JJ, et al. Olanzapine compared with chlorpromazine in treatment-resistant schizophrenia. Am J Psychiatry. 1998;155(7):914-920. doi:10.1176/ajp.155.7.914 | wrong C - other active comparators |
| Pharmacological | Conley 2003 | Conley RR, Kelly DL, Richardson CM, Tamminga CA, Carpenter WT Jr. The efficacy of high-dose olanzapine versus clozapine in treatment-resistant schizophrenia: a double-blind crossover study. J Clin Psychopharmacol. 2003;23(6):668-671. doi:10.1097/01.jcp.0000096246.29231.73 | wrong C - other active comparators |
| Pharmacological | Conley 2005 | Conley RR, Kelly DL, Nelson MW, et al. Risperidone, quetiapine, and fluphenazine in the treatment of patients with therapy-refractory schizophrenia. Clin Neuropharmacol. 2005;28(4):163-168. doi:10.1097/01.wnf.0000172993.89879.0f | wrong C - other active comparators |
| Pharmacological | Emsley 2000 | Emsley RA, Raniwalla J, Bailey PJ, Jones AM. A comparison of the effects of quetiapine ('seroquel') and haloperidol in schizophrenic patients with a history of and a demonstrated, partial response to conventional antipsychotic treatment. PRIZE Study Group. Int Clin Psychopharmacol. 2000;15(3):121-131. doi:10.1097/00004850-200015030-00001 | wrong C - other active comparators |
| Pharmacological | Fan 2013 | Fan X, Borba CP, Copeland P, et al. Metabolic effects of adjunctive aripiprazole in clozapine-treated patients with schizophrenia. Acta Psychiatr Scand. 2013;127(3):217-226. doi:10.1111/acps.12009 | wrong P - dosage and/or duration not clearly stated |
| Pharmacological | Feng 2001_SGP | 冯春霞, et al. 利培酮与氯丙嗪治疗难治性精神分裂症对照研究. 山东精神医学. 2001. | From less experienced countries |
| Pharmacological | Geller 2005 | Geller V, Gorzaltsan I, Shleifer T, Belmaker RH, Bersudsky Y. Clotiapine compared with chlorpromazine in chronic schizophrenia. Schizophr Res. 2005;80(2-3):343-347. doi:10.1016/j.schres.2005.07.007 | wrong C - other active comparators |
| Pharmacological | Genc 2007 | Genç Y, Taner E, Candansayar S. Comparison of clozapine-amisulpride and clozapine-quetiapine combinations for patients with schizophrenia who are partially responsive to clozapine: a single-blind randomized study. Adv Ther. 2007;24(1):1-13. doi:10.1007/BF02849987 | wrong P - duration not adequate |
| Pharmacological | Goff 2013 | Goff DC, McEvoy JP, Citrome L, et al. High-dose oral ziprasidone versus conventional dosing in schizophrenia patients with residual symptoms: the ZEBRAS study. J Clin Psychopharmacol. 2013;33(4):485-490. doi:10.1097/JCP.0b013e3182977308 | wrong P - duration not adequate |
| Pharmacological | Gonier 1970 | Gonier T, Schiele BC, Vestre ND. A comparison of haloperidol and thioridazine HCl in chronic treatment-resistant schizophrenics. Behav Neuropsychiatry. 1970;2(3):. | wrong design - not RCT |
| Pharmacological | Hall 1968 | Hall WB, Vestre ND, Schiele BC, Zimmermann R. A controlled comparison of haloperidol and fluphenazine in chronic treatment-resistant schizophrenics. Dis Nerv Syst. 1968;29(6):405-408. | wrong C - vs placebo only |
| Pharmacological | Hamilton 1960 | HAMILTON M, SMITH AL, LAPIDUS HE, CADOGAN EP. A controlled trial of thiopropazate dihydrochloride (dartalan). chlorpromazine and occupational therapy in chronic schizophrenics. J Ment Sci. 1960;106:40-55. doi:10.1192/bjp.106.442.40 | wrong P - duration not adequate |
| Pharmacological | Hatta 2012 | Hatta K, Otachi T, Sudo Y, et al. A comparison between augmentation with olanzapine and increased risperidone dose in acute schizophrenia patients showing early non-response to risperidone. Psychiatry Res. 2012;198(2):194-201. doi:10.1016/j.psychres.2012.01.006 | wrong P - duration not adequate |
| Pharmacological | Hatta 2014a | Hatta K, Otachi T, Fujita K, et al. Antipsychotic switching versus augmentation among early non-responders to risperidone or olanzapine in acute-phase schizophrenia. Schizophr Res. 2014;158(1-3):213-222. doi:10.1016/j.schres.2014.07.015 | wrong P - duration not adequate |
| Pharmacological | Hatta 2014b | Hatta K, Otachi T, Fujita K, et al. Antipsychotic switching versus augmentation among early non-responders to risperidone or olanzapine in acute-phase schizophrenia. Schizophr Res. 2014;158(1-3):213-222. doi:10.1016/j.schres.2014.07.015 | wrong P - dosage and/or duration not clearly stated |
| Pharmacological | Heylen 1988 | Heylen S, Gelders Y, Veoden Bussche G. RISPERIDONE VERSUS HALOPERIDOL IN CHRONIC PSYCHOTIC PATIENTS: AN 8 WEEK MULTICENTER DOUBLE-BLIND COMPARATIVE TRIAL. Psychopharmacology 96 (Suppl.) p238. 1988 | wrong C - other active comparators |
| Pharmacological | Honigfeld 1984b | Honigfeld G, Patin J, Singer J. Clozapine: Antipsychotic Activity in Treatment-Resistant Schizophrenics. Advances In Therapy. 1984. | wrong P - dosage and/or duration not clearly stated |
| Pharmacological | Howard 1974 | Howard JS 3rd. Haloperidol for chronically hospitalized psychotics: a double-blind comparison with thiothixene and placebo; a follow-up open evaluation. Dis Nerv Syst. 1974;35(10):458-463. | wrong C - other active comparators |
| Pharmacological | Kahn 2018 | Kahn RS, Winter van Rossum I, Leucht S, et al. Amisulpride and olanzapine followed by open-label treatment with clozapine in first-episode schizophrenia and schizophreniform disorder (OPTiMiSE): a three-phase switching study. Lancet Psychiatry. 2018;5(10):797-807. doi:10.1016/S2215-0366(18)30252-9 | wrong C - other active comparators |
| Pharmacological | Kane 2006 | Kane JM, Khanna S, Rajadhyaksha S, Giller E. Efficacy and tolerability of ziprasidone in patients with treatment-resistant schizophrenia. Int Clin Psychopharmacol. 2006;21(1):21-28. doi:10.1097/01.yic.0000182114.65134.81 | wrong C - other active comparators |
| Pharmacological | Kane 2007a | Kane JM, Meltzer HY, Carson WH Jr, et al. Aripiprazole for treatment-resistant schizophrenia: results of a multicenter, randomized, double-blind, comparison study versus perphenazine. J Clin Psychiatry. 2007;68(2):213-223. | wrong C - other active comparators |
| Pharmacological | Kane 2010b | Kane JM, Potkin SG, Daniel DG, Buckley PF. A double-blind, randomized study comparing the efficacy and safety of sertindole and risperidone in patients with treatment-resistant schizophrenia. J Clin Psychiatry. 2011;72(2):194-204. doi:10.4088/JCP.07m03733yel | wrong C - other active comparators |
| Pharmacological | Kinon 2009 | Kinon BJ, Chen L, Ascher-Svanum H, et al. Early response to antipsychotic drug therapy as a clinical marker of subsequent response in the treatment of schizophrenia. Neuropsychopharmacology. 2010;35(2):581-590. doi:10.1038/npp.2009.164 | wrong P - duration not adequate |
| Pharmacological | Kluge 2007 | Kluge M, Schuld A, Himmerich H, et al. Clozapine and olanzapine are associated with food craving and binge eating: results from a randomized double-blind study. J Clin Psychopharmacol. 2007;27(6):662-666. doi:10.1097/jcp.0b013e31815a8872 | wrong P - dosage and/or duration not clearly stated |
| Pharmacological | Kotler 2004 | Kotler M, Strous RD, Reznik I, Shwartz S, Weizman A, Spivak B. Sulpiride augmentation of olanzapine in the management of treatment-resistant chronic schizophrenia: evidence for improvement of mood symptomatology. Int Clin Psychopharmacol. 2004;19(1):23-26. doi:10.1097/00004850-200401000-00004 | wrong design - open label |
| Pharmacological | Kreinin 2006 | Kreinin A, Novitski D, Weizman A. Amisulpride treatment of clozapine-induced hypersalivation in schizophrenia patients: a randomized, double-blind, placebo-controlled cross-over study. Int Clin Psychopharmacol. 2006;21(2):99-103. doi:10.1097/01.yic.0000188216.92408.69 | wrong design - others |
| Pharmacological | Kumra 1996 | Kumra S, Frazier JA, Jacobsen LK, et al. Childhood-onset schizophrenia. A double-blind clozapine-haloperidol comparison. Arch Gen Psychiatry. 1996;53(12):1090-1097. doi:10.1001/archpsyc.1996.01830120020005 | wrong P - dosage and/or duration not clearly stated |
| Pharmacological | Kumra 2007 | Kumra S, Kranzler H, Gerbino-Rosen G, et al. Clozapine and "high-dose" olanzapine in refractory early-onset schizophrenia: a 12-week randomized and double-blind comparison. Biol Psychiatry. 2008;63(5):524-529. doi:10.1016/j.biopsych.2007.04.043 | wrong P - dosage and/or duration not clearly stated |
| Pharmacological | Lal 2006 | Lal S, Thavundayil JX, Nair NP, et al. Levomepromazine versus chlorpromazine in treatment-resistant schizophrenia: a double-blind randomized trial. J Psychiatry Neurosci. 2006;31(4):271-279. | wrong C - other active comparators |
| Pharmacological | Lewis 2006 | Lewis SW, Barnes TR, Davies L, et al. Randomized controlled trial of effect of prescription of clozapine versus other second-generation antipsychotic drugs in resistant schizophrenia. Schizophr Bull. 2006;32(4):715-723. doi:10.1093/schbul/sbj067 | wrong P - dosage and/or duration not clearly stated |
| Pharmacological | Lin 2013 | Lin CC, Chiu HJ, Chen JY, et al. Switching from clozapine to zotepine in patients with schizophrenia: a 12-week prospective, randomized, rater blind, and parallel study. J Clin Psychopharmacol. 2013;33(2):211-214. doi:10.1097/JCP.0b013e31828700c7 | wrong C - (de-escalation) |
| Pharmacological | Lin 2013_SGP | 林昭宇, et al. 帕利哌酮与氟哌啶醇治疗精神分裂症的双盲对照研究. Journal of Psychiatry. 2013. | From less experienced countries |
| Pharmacological | Marjerrison 1964 | MARJERRISON G, IRVINE D, STEWART CN, WILLIAMS R, MATHEU H, DEMAY M. WITHDRAWAL OF LONG-TERM PHENOTHIAZINES FROM CHRONICALLY HOSPITALIZED PSYCHIATRIC PATIENTS. Can Psychiatr Assoc J. 1964;9:290-298. doi:10.1177/070674376400900404 | wrong C - other active comparators |
| Pharmacological | Mayabhate 2014 | Mayabhate, M. M., Badar, V. A., Waradkar, P., & Somani, A. (2017). Cognitive and psychomotor effects of adjunctive aripiprazole or paliperidone in patients of schizophrenia receiving olanzapine: a double blind placebo controlled clinical study. International Journal of Basic & Clinical Pharmacology, 3(1), 130–138. Retrieved from https://www.ijbcp.com/index.php/ijbcp/article/view/967 | wrong P - dosage and/or duration not clearly stated |
| Pharmacological | McCreadie 1977 | McCreadie RG, MacDonald IM. High dosage haloperidol in chronic schizophrenia. Br J Psychiatry. 1977;131:310-316. doi:10.1192/bjp.131.3.310 | wrong P - dosage and/or duration not clearly stated |
| Pharmacological | McEvoy 1991 | McEvoy JP, Hogarty GE, Steingard S. Optimal dose of neuroleptic in acute schizophrenia. A controlled study of the neuroleptic threshold and higher haloperidol dose. Arch Gen Psychiatry. 1991;48(8):739-745. doi:10.1001/archpsyc.1991.01810320063009 | wrong P - duration not adequate |
| Pharmacological | McEvoy 2006 | McEvoy JP, Lieberman JA, Stroup TS, et al. Effectiveness of clozapine versus olanzapine, quetiapine, and risperidone in patients with chronic schizophrenia who did not respond to prior atypical antipsychotic treatment. Am J Psychiatry. 2006;163(4):600-610. doi:10.1176/ajp.2006.163.4.600 | wrong P - dosage and/or duration not clearly stated |
| Pharmacological | McGorry 2011 | McGorry PD, Cocks J, Power P, Burnett P, Harrigan S, Lambert T. Very low-dose risperidone in first-episode psychosis: a safe and effective way to initiate treatment. Schizophr Res Treatment. 2011;2011:631690. doi:10.1155/2011/631690 | wrong design - open label |
| Pharmacological | McGurk 2005 | McGurk SR, Carter C, Goldman R, et al. The effects of clozapine and risperidone on spatial working memory in schizophrenia. Am J Psychiatry. 2005;162(5):1013-1016. doi:10.1176/appi.ajp.162.5.1013 | wrong design - subgroup analysis of an already included trial |
| Pharmacological | Meltzer 2014 | Meltzer HY, Lindenmayer JP, Kwentus J, Share DB, Johnson R, Jayathilake K. A six month randomized controlled trial of long acting injectable risperidone 50 and 100mg in treatment resistant schizophrenia. Schizophr Res. 2014;154(1-3):14-22. doi:10.1016/j.schres.2014.02.015 | wrong C - other active comparators |
| Pharmacological | Mercer 1997 | Mercer G, Finlayson A, Johnstone EC, Murray C, Owens DG. A study of enhanced management in patients with treatment-resistant schizophrenia. J Psychopharmacol. 1997;11(4):349-356. doi:10.1177/026988119701100411 | wrong C - other active comparators |
| Pharmacological | Mergl 1999 | Mergl RP, et al. Digitised hand movement analysis of EPMS. 11th World Congress of Psychiatry. 1999. | wrong design - subgroup analysis of an already included trial |
| Pharmacological | Meyer-Lindenberg 1997 | Meyer-Lindenberg A, Gruppe H, Bauer U, Lis S, Krieger S, Gallhofer B. Improvement of cognitive function in schizophrenic patients receiving clozapine or zotepine: results from a double-blind study. Pharmacopsychiatry. 1997;30(2):35-42. doi:10.1055/s-2007-979481 | wrong P - duration not adequate |
| Pharmacological | NCT02717195 | Effect of Lu AF35700 in Patients With Treatment-resistant Schizophrenia (DayBreak) | wrong C - other active comparators |
| Pharmacological | Naber 2005 | Naber D, Riedel M, Klimke A, et al. Randomized double blind comparison of olanzapine vs. clozapine on subjective well-being and clinical outcome in patients with schizophrenia. Acta Psychiatr Scand. 2005;111(2):106-115. doi:10.1111/j.1600-0447.2004.00486.x | wrong P - dosage and/or duration not clearly stated |
| Pharmacological | Naber 2005_cognition | Naber D, Riedel M, Klimke A, et al. Randomized double blind comparison of olanzapine vs. clozapine on subjective well-being and clinical outcome in patients with schizophrenia. Acta Psychiatr Scand. 2005;111(2):106-115. doi:10.1111/j.1600-0447.2004.00486.x | wrong design - subgroup analysis of an already included trial |
| Pharmacological | Ning 2004_SGP | 宁布, et al. 利培酮与氯氮平治疗难治性精神分裂症临床对照研究. Chinese Journal of Clinical practical Medicine. 2004. | From less experienced countries |
| Pharmacological | O Reilly 1957 | O'REILLY PO, WOJCICKI HM, HRYCHUK W, KEOGH RP. Perphenazine (trilafon) treatment of psychoses. Can Med Assoc J. 1957;77(10):952-955. | wrong P - dosage and/or duration not clearly stated |
| Pharmacological | Oliemeulen 2000 | Oliemeulen EAP, et al. B.29. IS OLANZAPINE A SUBSTITUTE FOR CLOZAPINE? THE EFFECTS ON PSYCHOMOTOR PERFORMANCE. | wrong P - dosage and/or duration not clearly stated |
| Pharmacological | Peet 1981 | Peet M, Bethell MS, Coates A, et al. Propranolol in schizophrenia. I. Comparison of propranolol, chlorpromazine and placebo. Br J Psychiatry. 1981;139:105-111. doi:10.1192/bjp.139.2.105 | wrong C - other active comparators |
| Pharmacological | Potkin 1993 | Potkin SG, Bera R, Gulasekaram B, et al. Plasma clozapine concentrations predict clinical response in treatment-resistant schizophrenia. J Clin Psychiatry. 1994;55 Suppl B:133-136. | wrong P - dosage and/or duration not clearly stated |
| Pharmacological | Potkin 2003b | Potkin SG, Basile VS, Jin Y, et al. D1 receptor alleles predict PET metabolic correlates of clinical response to clozapine. Mol Psychiatry. 2003;8(1):109-113. doi:10.1038/sj.mp.4001191 | wrong C - other active comparators |
| Pharmacological | Repo-Tiihonen 2012 | Repo-Tiihonen E, Hallikainen T, Kivistö P, Tiihonen J. Antipsychotic Polypharmacy in Clozapine Resistant Schizophrenia: A Randomized Controlled Trial of Tapering Antipsychotic Co-treatment. Ment Illn. 2012;4(1):e1. Published 2012 Jan 30. doi:10.4081/mi.2012.e1 | wrong C - other active comparators |
| Pharmacological | Schiele 1961, 06602 | SCHIELE BC, VESTRE ND, STEIN KE. A comparison of thioridazine, trifluoperazine, chlorpromazine, and placebo: a double-blind controlled study on the treatment of chronic, hospitalized, schizophrenic patients. J Clin Exp Psychopathol Q Rev Psychiatry Neurol. 1961;22:151-162. | wrong C - other active comparators |
| Pharmacological | Schlosberg 1978 | Schlosberg A, et al. A comparative controlled study of two long-acting phenothiazines: pipotiazine palmitate and fluphenazine decanoate. Current Therapeutic Research. 1978. | wrong C - other active comparators |
| Pharmacological | See 1999 | See RE, Fido AA, Maurice M, Ibrahim MM, Salama GM. Risperidone-induced increase of plasma norepinephrine is not correlated with symptom improvement in chronic schizophrenia. Biol Psychiatry. 1999;45(12):1653-1656. doi:10.1016/s0006-3223(98)00199-1 | wrong C - other active comparators |
| Pharmacological | Shalev 1993 | Shalev A, Hermesh H, Rothberg J, Munitz H. Poor neuroleptic response in acutely exacerbated schizophrenic patients. Acta Psychiatr Scand. 1993;87(2):86-91. doi:10.1111/j.1600-0447.1993.tb03335.x | wrong C - other active comparators |
| Pharmacological | Shaw 2006 | Shaw P, Sporn A, Gogtay N, et al. Childhood-onset schizophrenia: A double-blind, randomized clozapine-olanzapine comparison. Arch Gen Psychiatry. 2006;63(7):721-730. doi:10.1001/archpsyc.63.7.721 | wrong P - children |
| Pharmacological | Shoja-Shafti 2009 | Shoja-Shafti S. Augmentation of olanzapine by fluphenazine decanoate in poorly responsive schizophrenia. Clinical schizophrenia & related psychosis. 2009. | wrong design - high risk of bias during randomization |
| Pharmacological | Shoja-Shafti 2017 | Saeed Shoja Shafti (2017) Augmentation of aripiprazole by flupenthixol decanoate in poorly responsive schizophrenia: a randomized clinical study, Psychiatry and Clinical Psychopharmacology, 27:3, 235-242, DOI: 10.1080/24750573.2017.1342753 | wrong design - high risk of bias during randomization |
| Pharmacological | Simpson 1999 | Simpson GM, Josiassen RC, Stanilla JK, et al. Double-blind study of clozapine dose response in chronic schizophrenia. Am J Psychiatry. 1999;156(11):1744-1750. doi:10.1176/ajp.156.11.1744 | wrong C - other active comparators |
| Pharmacological | Sirota 2006 | Sirota P, Pannet I, Koren A, Tchernichovsky E. Quetiapine versus olanzapine for the treatment of negative symptoms in patients with schizophrenia. Hum Psychopharmacol. 2006;21(4):227-234. doi:10.1002/hup.763 | wrong P - predominently negative |
| Pharmacological | Smith 2001 | Smith RC, Infante M, Singh A, Khandat A. The effects of olanzapine on neurocognitive functioning in medication-refractory schizophrenia. Int J Neuropsychopharmacol. 2001;4(3):239-250. doi:10.1017/s146114570100253x | wrong C - other active comparators |
| Pharmacological | Sulejmanpasic-Arslanagic 2019a | Sulejmanpasic-Arslanagic G, Bise S. Clozapine augmented with amisulpride in treatment-reisistant schizophrenia. 2019 | wrong P - dosage and/or duration not clearly stated |
| Pharmacological | Suzuki 2007 | Suzuki T, Uchida H, Watanabe K, et al. How effective is it to sequentially switch among Olanzapine, Quetiapine and Risperidone?--A randomized, open-label study of algorithm-based antipsychotic treatment to patients with symptomatic schizophrenia in the real-world clinical setting. Psychopharmacology (Berl). 2007;195(2):285-295. doi:10.1007/s00213-007-0872-2 | wrong design - open label |
| Pharmacological | Tablot 1964a | TALBOT DR. ARE TRANQUILIZER COMBINATIONS MORE EFFECTIVE THAN A SINGLE TRANQUILIZER?. Am J Psychiatry. 1964;121:597-600. doi:10.1176/ajp.121.6.597 | wrong P - dosage and/or duration not clearly stated |
| Pharmacological | Tablot 1964b | TALBOT DR. ARE TRANQUILIZER COMBINATIONS MORE EFFECTIVE THAN A SINGLE TRANQUILIZER?. Am J Psychiatry. 1964;121:597-600. doi:10.1176/ajp.121.6.597 | wrong P - dosage and/or duration not clearly stated |
| Pharmacological | Teja 1975 | Teja JS, Grey WH, Clums JM, Warren C. Tranquilizers or anti-depressants for chronic schizophrenics: a long term study. Aust N Z J Psychiatry. 1975;9(4):241-247. doi:10.3109/00048677509159857 | wrong design - not RCT |
| Pharmacological | Toru 1972 | Toru M, Shimazono Y, Miyasaka M, Kokubo T, Mori Y, Nasu T. A double-blind comparison of sulpiride with chlorpromazine in chronic schizophrenia. J Clin Pharmacol New Drugs. 1972;12(5):221-229. doi:10.1002/j.1552-4604.1972.tb00166.x | wrong C - other active comparators |
| Pharmacological | Wang 2004_SGP | 王立, et al. 奥氮平与氯丙嗪治疗难治性精神分裂症的对照研究. Health Psychology Journal. 2004. | wrong design - not RCT |
| Pharmacological | Wang 2007_SGP | 王艳君. 奥氮平与氯氮平治疗难治性精神分裂症的对照研究. Practical Pharmacy And Clinical Remedies. 2007. | From less experienced countries |
| Pharmacological | Wang 2022 | Wang D, Wei N, Hu F, et al. Paliperidone Extended Release Versus Olanzapine in Treatment-Resistant Schizophrenia: A Randomized, Double-Blind, Multicenter Study. J Clin Psychopharmacol. 2022;42(4):383-390. doi:10.1097/JCP.0000000000001573 | wrong C - other active comparators |
| Pharmacological | Wirshing 1999 | Wirshing DA, Marshall BD Jr, Green MF, Mintz J, Marder SR, Wirshing WC. Risperidone in treatment-refractory schizophrenia. Am J Psychiatry. 1999;156(9):1374-1379. doi:10.1176/ajp.156.9.1374 | wrong C - other active comparators |
| Pharmacological | Yan 2008b_SGP | 王玲, et al. 齐拉西酮片与利培酮治疗精神分裂症对照研究. Journal of Medical Forum. 2008. | From less experienced countries |
| Pharmacological | Zhang 1999_SGP | 张向阳, et al. 精神分裂症神经内分泌、免疫、自由基代谢与治疗药物的关系. Chin J Psychiatry. 1999. | From less experienced countries |
| Pharmacological | Zhang 2009f_SGP | 张帆, et al. 齐拉西酮与氯氮平治疗女性难治性精神分裂症对照研究. Journal of Psychiatry. 2009. | From less experienced countries |
| Pharmacological | Zhu 2022 | Zhu MH, Liu ZJ, Hu QY, et al. Amisulpride augmentation therapy improves cognitive performance and psychopathology in clozapine-resistant treatment-refractory schizophrenia: a 12-week randomized, double-blind, placebo-controlled trial. Mil Med Res. 2022;9(1):59. Published 2022 Oct 18. doi:10.1186/s40779-022-00420-0 | From less experienced countries |
| Pharmacological | Zink 2009 | Zink M, Kuwilsky A, Krumm B, Dressing H. Efficacy and tolerability of ziprasidone versus risperidone as augmentation in patients partially responsive to clozapine: a randomised controlled clinical trial. J Psychopharmacol. 2009;23(3):305-314. doi:10.1177/0269881108089593 | wrong C - other active comparators |
| Psychological | Ali beigi 2012 | Ali beigi N, et al. A Randomized Controlled Trial of Group Cognitive- Behavior Therapy, Cognitive-Remediation Therapy and Combined Cognitive Therapy on improving function and reducing symptoms for Patients with Schizophrenia. European Journal of Experimental Biology. 2012. | wrong P - dosage and/or duration not clearly stated |
| Psychological | Barrowclough 2006 | Barrowclough C, Haddock G, Lobban F, et al. Group cognitive-behavioural therapy for schizophrenia. Randomised controlled trial. Br J Psychiatry. 2006;189:527-532. doi:10.1192/bjp.bp.106.021386 | wrong P - dosage and/or duration not clearly stated |
| Psychological | ChiCTR-IPR-17011541 | Cognitive behavioral therapy for worry in people with persecutory delusion: a randomized controlled trial in Chinese population. | wrong design - protocol only |
| Psychological | DRKS00012523 | MOtivation and SociAl Interactive Competence in patients with negative symptoms: Innovative cognitive behavioral therapy and neurobiological correlates (MOSAIC) | wrong P - predominently negative |
| Psychological | Dellazizzo 2021 | Dellazizzo L, Potvin S, Phraxayavong K, Dumais A. One-year randomized trial comparing virtual reality-assisted therapy to cognitive-behavioral therapy for patients with treatment-resistant schizophrenia. NPJ Schizophr. 2021;7(1):9. Published 2021 Feb 12. doi:10.1038/s41537-021-00139-2 | wrong C - other active comparators |
| Psychological | Foster 2010 | Foster C, Startup H, Potts L, Freeman D. A randomised controlled trial of a worry intervention for individuals with persistent persecutory delusions. J Behav Ther Exp Psychiatry. 2010;41(1):45-51. doi:10.1016/j.jbtep.2009.09.001 | wrong P - duration not adequate |
| Psychological | Freeman 2014 | Freeman D, Pugh K, Dunn G, et al. An early Phase II randomised controlled trial testing the effect on persecutory delusions of using CBT to reduce negative cognitions about the self: the potential benefits of enhancing self confidence. Schizophr Res. 2014;160(1-3):186-192. doi:10.1016/j.schres.2014.10.038 | wrong P - dosage not adequate |
| Psychological | Freeman 2015 (WIT) | Freeman D, Dunn G, Startup H, Kingdon D. An explanatory randomised controlled trial testing the effects of targeting worry in patients with persistent persecutory delusions: the Worry Intervention Trial (WIT). Southampton (UK): NIHR Journals Library; March 2015. | wrong P - dosage not adequate |
| Psychological | Freeman 2015b | Freeman D, Waite F, Startup H, et al. Efficacy of cognitive behavioural therapy for sleep improvement in patients with persistent delusions and hallucinations (BEST): a prospective, assessor-blind, randomised controlled pilot trial. Lancet Psychiatry. 2015;2(11):975-983. doi:10.1016/S2215-0366(15)00314-4 | wrong P - dosage and/or duration not clearly stated |
| Psychological | Garety 2021b | Garety P, Ward T, Emsley R, et al. Effects of SlowMo, a Blended Digital Therapy Targeting Reasoning, on Paranoia Among People With Psychosis: A Randomized Clinical Trial. JAMA Psychiatry. 2021;78(7):714-725. doi:10.1001/jamapsychiatry.2021.0326 | wrong P - dosage and/or duration not clearly stated |
| Psychological | Haddock 1998 | Haddock G, Slade PD, Bentall RP, Reid D, Faragher EB. A comparison of the long-term effectiveness of distraction and focusing in the treatment of auditory hallucinations. Br J Med Psychol. 1998;71(3):339-349. doi:10.1111/j.2044-8341.1998.tb00996.x | wrong P - dosage and/or duration not clearly stated |
| Psychological | Haddock 2009 | Haddock G, Barrowclough C, Shaw JJ, Dunn G, Novaco RW, Tarrier N. Cognitive-behavioural therapy v. social activity therapy for people with psychosis and a history of violence: randomised controlled trial. Br J Psychiatry. 2009;194(2):152-157. doi:10.1192/bjp.bp.107.039859 | wrong P - dosage and/or duration not clearly stated |
| Psychological | Hayward 2009 | Hayward P, et al. Promoting Therapeutic Alliance in Clozapine Users: An Exploratory Randomized Controlled Trial. Clinical Schizophrenia & Related Psychoses. 2009. | wrong P - dosage and/or duration not clearly stated |
| Psychological | Isham 2018 | Isham L, Grafahrend H, Nickless A, et al. Group-Based Worry Intervention for Persecutory Delusions: an Initial Feasibility Study. Behav Cogn Psychother. 2018;46(5):619-625. doi:10.1017/S1352465818000383 | wrong P - dosage and/or duration not clearly stated |
| Psychological | Krakvik 2013 | Kråkvik B, Gråwe RW, Hagen R, Stiles TC. Cognitive behaviour therapy for psychotic symptoms: a randomized controlled effectiveness trial. Behav Cogn Psychother. 2013;41(5):511-524. doi:10.1017/S1352465813000258 | wrong P - dosage and/or duration not clearly stated |
| Psychological | Kuipers 1997 | Kuipers E, Garety P, Fowler D, et al. London-East Anglia randomised controlled trial of cognitive-behavioural therapy for psychosis. I: effects of the treatment phase. Br J Psychiatry. 1997;171:319-327. doi:10.1192/bjp.171.4.319 | wrong P - dosage not adequate |
| Psychological | Lee 2013 PT TR | Lee DE, et al. The Effect of Cognitive Behavioral Therapy in Drug-Resistant Patients with Schizophrenia. J Korean Neuropsychiatr Assoc. 2013. | wrong P - dosage and/or duration not clearly stated |
| Psychological | Liang 2022 | Liang N, Li X, Guo X, et al. Visual P300 as a neurophysiological correlate of symptomatic improvement by a virtual reality-based computer AT system in patients with auditory verbal hallucinations: A Pilot study. J Psychiatr Res. 2022;151:261-271. doi:10.1016/j.jpsychires.2022.04.027 | From less experienced countries |
| Psychological | NCT00688259 | Cognitive-Behavioral Therapy in Veterans With Schizophrenia | wrong P - dosage and/or duration not clearly stated |
| Psychological | NCT02787135 | Efficacy and Mechanisms of Change of an Emotion-oriented Version of Cognitive-behavioral Therapy for Psychosis (CBTd-E) | wrong P - dosage and/or duration not clearly stated |
| Psychological | Penn 2009 | Penn DL, Meyer PS, Evans E, Wirth RJ, Cai K, Burchinal M. A randomized controlled trial of group cognitive-behavioral therapy vs. enhanced supportive therapy for auditory hallucinations. Schizophr Res. 2009;109(1-3):52-59. doi:10.1016/j.schres.2008.12.009 | wrong P - dosage and/or duration not clearly stated |
| Psychological | Rector 2003 | Rector NA, Seeman MV, Segal ZV. Cognitive therapy for schizophrenia: a preliminary randomized controlled trial. Schizophr Res. 2003;63(1-2):1-11. doi:10.1016/s0920-9964(02)00308-0 | wrong P - dosage not adequate |
| Psychological | Shawyer 2012 | Shawyer F, Farhall J, Mackinnon A, et al. A randomised controlled trial of acceptance-based cognitive behavioural therapy for command hallucinations in psychotic disorders. Behav Res Ther. 2012;50(2):110-121. doi:10.1016/j.brat.2011.11.007 | wrong P - dosage and/or duration not clearly stated |
| Psychological | Tarrier 1998 | Tarrier N, Yusupoff L, Kinney C, et al. Randomised controlled trial of intensive cognitive behaviour therapy for patients with chronic schizophrenia. BMJ. 1998;317(7154):303-307. doi:10.1136/bmj.317.7154.303 | wrong P - dosage and/or duration not clearly stated |
| Psychological | Turkington 2002 | Turkington D, Kingdon D, Turner T; Insight into Schizophrenia Research Group. Effectiveness of a brief cognitive-behavioural therapy intervention in the treatment of schizophrenia. Br J Psychiatry. 2002;180:523-527. doi:10.1192/bjp.180.6.523 | wrong P - dosage and/or duration not clearly stated |
| Psychological | Velligan 2015 | Velligan DI, Tai S, Roberts DL, et al. A randomized controlled trial comparing cognitive behavior therapy, cognitive adaptation training, their combination and treatment as usual in chronic schizophrenia. Schizophr Bull. 2015;41(3):597-603. doi:10.1093/schbul/sbu127 | wrong P - dosage and/or duration not clearly stated |
| Psychological | Wahass 1997 | Wahass S, Kent G. The Modification of Psychological Interventions for Persistent Auditory Hallucinations to an Islamic Culture. Behavioural and Cognitive Psychotherapy. 1997;25(4):351-364. doi:10.1017/S1352465800018750 | wrong P - dosage and/or duration not clearly stated |
| Psychological | Wittorf 2010_full study | Wittorf A, Jakobi UE, Bannert KK, et al. Does the cognitive dispute of psychotic symptoms do harm to the therapeutic alliance?. J Nerv Ment Dis. 2010;198(7):478-485. doi:10.1097/NMD.0b013e3181e4f526 | wrong P - dosage and/or duration not clearly stated |
| Psychological | Wong 2019 | Wong AWS, Ting KT, Chen EYH. Group cognitive behavioural therapy for Chinese patients with psychotic disorder: A feasibility controlled study. Asian J Psychiatr. 2019;39:157-164. doi:10.1016/j.ajp.2018.12.015 | wrong P - dosage and/or duration not clearly stated |
| Psychological | Wykes 2005 | Wykes T, Hayward P, Thomas N, et al. What are the effects of group cognitive behaviour therapy for voices? A randomised control trial. Schizophr Res. 2005;77(2-3):201-210. doi:10.1016/j.schres.2005.03.013 | wrong P - dosage and/or duration not clearly stated |
| NIBS | An 2011 | 安翠霞, et al. 不同部位低频重复经颅磁刺激治疗顽固性幻听疗效和安全性的双盲对照研究. Chin J Psychiatry. 2011. | From less experienced countries |
| NIBS | Argawal 1985 | Agarwal A, Winny GC. Role of ect phenothiazine combination in schizophrenia. Indian J Psychiatry. 1985;27(3):233-236. | wrong design - less than 3 weeks |
| NIBS | Aubonnet 2020 | Aubonnet R, Banea OC, Sirica R, et al. P300 Analysis Using High-Density EEG to Decipher Neural Response to rTMS in Patients With Schizophrenia and Auditory Verbal Hallucinations. Front Neurosci. 2020;14:575538. Published 2020 Nov 20. doi:10.3389/fnins.2020.575538 | wrong P - dosage and/or duration not clearly stated |
| NIBS | Bais 2014 | Bais L, Vercammen A, Stewart R, et al. Short and long term effects of left and bilateral repetitive transcranial magnetic stimulation in schizophrenia patients with auditory verbal hallucinations: a randomized controlled trial. PLoS One. 2014;9(10):e108828. Published 2014 Oct 20. doi:10.1371/journal.pone.0108828 | wrong design - less than 3 weeks |
| NIBS | Bose 2018 | Bose A, Shivakumar V, Agarwal SM, et al. Efficacy of fronto-temporal transcranial direct current stimulation for refractory auditory verbal hallucinations in schizophrenia: A randomized, double-blind, sham-controlled study. Schizophr Res. 2018;195:475-480. doi:10.1016/j.schres.2017.08.047 | not ECT or TMS |
| NIBS | Brunelin 2006 | Brunelin J, Poulet E, Bediou B, et al. Low frequency repetitive transcranial magnetic stimulation improves source monitoring deficit in hallucinating patients with schizophrenia. Schizophr Res. 2006;81(1):41-45. doi:10.1016/j.schres.2005.10.009 | wrong P - dosage and/or duration not clearly stated |
| NIBS | Brunelin 2012 | Brunelin J, Mondino M, Gassab L, et al. Examining transcranial direct-current stimulation (tDCS) as a treatment for hallucinations in schizophrenia [published correction appears in Am J Psychiatry. 2012 Dec 1;169(12):1321]. Am J Psychiatry. 2012;169(7):719-724. doi:10.1176/appi.ajp.2012.11071091 | not ECT or TMS |
| NIBS | Brunelin 2021 | Brunelin J, Mondino M, Haesebaert J, et al. Examining transcranial random noise stimulation as an add-on treatment for persistent symptoms in schizophrenia (STIM'Zo): a study protocol for a multicentre, double-blind, randomized sham-controlled clinical trial. Trials. 2021;22(1):964. Published 2021 Dec 28. doi:10.1186/s13063-021-05928-9 | wrong design - protocol only |
| NIBS | Chang 2018 | Chang CC, Tzeng NS, Chao CY, Yeh CB, Chang HA. The Effects of Add-on Fronto-Temporal Transcranial Direct Current Stimulation (tDCS) on Auditory Verbal Hallucinations, Other Psychopathological Symptoms, and Insight in Schizophrenia: A Randomized, Double-Blind, Sham-Controlled Trial. Int J Neuropsychopharmacol. 2018;21(11):979-987. doi:10.1093/ijnp/pyy074 | not ECT or TMS |
| NIBS | Chanpattana 2000 | Chanpattana W, Chakrabhand ML, Buppanharun W, Sackeim HA. Effects of stimulus intensity on the efficacy of bilateral ECT in schizophrenia: a preliminary study. Biol Psychiatry. 2000;48(3):222-228. doi:10.1016/s0006-3223(00)00830-1 | not ECT or TMS |
| NIBS | Charkhe 2022 | Charkhe P, et al. EFFICACY OF ADJUNCTIVE EXTENDED ROBOTIZED NEURONAVIGATED CONTINUOUS THETA BURST STIMULATION OVER THE LEFT SUPERIOR TEMPORAL CORTEX ON AUDITORY HALLUCINATIONS IN SCHIZOPHRENIA: A RANDOMIZED SHAM CONTROLLED STUDY. Dissertation. | not ECT or TMS |
| NIBS | Chauhan 2021 | Chauhan P, Garg S, Tikka SK, Khattri S. Efficacy of Intensive Cerebellar Intermittent Theta Burst Stimulation (iCiTBS) in Treatment-Resistant Schizophrenia: a Randomized Placebo-Controlled Study. Cerebellum. 2021;20(1):116-123. doi:10.1007/s12311-020-01193-9 | wrong design - less than 3 weeks |
| NIBS | Chen 2024 | 陈基娜, et al. 不同rTMS模式对精神分裂症幻听患者的应用效果研究. 2024. | From less experienced countries |
| NIBS | Cheng 2013 | Cheng J, Li H, Shi WZ. Clinical efficacy of clozapine combined with repetitive transcranial magnetic stimulation in the treatment of refractory schizophrenia. Chinese Journal of Rehabilitation Medicine. 2013 | From less experienced countries |
| NIBS | ChiCTR2100047636 | Transcranial Direct-Current stimulation to improve auditory hallucination in schizophrenia and explore the electrophysiological mechanism : A Randomized controlled study | not ECT or TMS |
| NIBS | ChiCTR2100052759 | cTBS treatment for auditory hallucinations in schizophrenia: a randomized controlled trial. | Chinese study |
| NIBS | Chibbaro 2005 | Chibbaro G, Daniele M, Alagona G, et al. Repetitive transcranial magnetic stimulation in schizophrenic patients reporting auditory hallucinations. Neurosci Lett. 2005;383(1-2):54-57. doi:10.1016/j.neulet.2005.03.052 | not ECT or TMS |
| NIBS | Dollfus 2018 | Dollfus S, Jaafari N, Guillin O, et al. High-Frequency Neuronavigated rTMS in Auditory Verbal Hallucinations: A Pilot Double-Blind Controlled Study in Patients With Schizophrenia. Schizophr Bull. 2018;44(3):505-514. doi:10.1093/schbul/sbx127 | wrong P - dosage and/or duration not clearly stated |
| NIBS | Donde 2023 | Dondé C, Fivel L, Haesebaert F, Poulet E, Mondino M, Brunelin J. Mechanistic account of the left auditory cortex for tone-matching in schizophrenia: A pilot transcranial random noise stimulation (tRNS) sham-controlled study. Asian J Psychiatr. 2024;92:103879. doi:10.1016/j.ajp.2023.103879 | not ECT or TMS |
| NIBS | Fitzgerald 2005 | Fitzgerald PB, Benitez J, Daskalakis JZ, et al. A double-blind sham-controlled trial of repetitive transcranial magnetic stimulation in the treatment of refractory auditory hallucinations. J Clin Psychopharmacol. 2005;25(4):358-362. doi:10.1097/01.jcp.0000168487.22140.7f | wrong design - less than 3 weeks |
| NIBS | Fitzgerald 2014a | Fitzgerald PB, McQueen S, Daskalakis ZJ, Hoy KE. A negative pilot study of daily bimodal transcranial direct current stimulation in schizophrenia. Brain Stimul. 2014;7(6):813-816. doi:10.1016/j.brs.2014.08.002 | not ECT or TMS |
| NIBS | Fitzgerald 2014b | Fitzgerald PB, McQueen S, Daskalakis ZJ, Hoy KE. A negative pilot study of daily bimodal transcranial direct current stimulation in schizophrenia. Brain Stimul. 2014;7(6):813-816. doi:10.1016/j.brs.2014.08.002 | not ECT or TMS |
| NIBS | Fröhlich 2015 | Fröhlich F, Burrello TN, Mellin JM, et al. Exploratory study of once-daily transcranial direct current stimulation (tDCS) as a treatment for auditory hallucinations in schizophrenia. Eur Psychiatry. 2016;33:54-60. doi:10.1016/j.eurpsy.2015.11.005 | not ECT or TMS |
| NIBS | Gao 2009 | 高志勤, et al. 低频重复经颅磁刺激治疗精神分裂症慢性幻听的疗效及随访研究. 2009. | From less experienced countries |
| NIBS | Gornerova 2023 | Gornerova N, Brunovsky M, Klirova M, et al. The effect of low-frequency rTMS on auditory hallucinations, EEG source localization and functional connectivity in schizophrenia. Neurosci Lett. 2023;794:136977. doi:10.1016/j.neulet.2022.136977 | wrong design - less than 3 weeks |
| NIBS | Hoffman 2000 | Hoffman RE, Boutros NN, Hu S, Berman RM, Krystal JH, Charney DS. Transcranial magnetic stimulation and auditory hallucinations in schizophrenia. Lancet. 2000;355(9209):1073-1075. doi:10.1016/S0140-6736(00)02043-2 | wrong design - subgroup analysis of an already included trial |
| NIBS | Hoffman 2005 | Hoffman RE, Gueorguieva R, Hawkins KA, et al. Temporoparietal transcranial magnetic stimulation for auditory hallucinations: safety, efficacy and moderators in a fifty patient sample. Biol Psychiatry. 2005;58(2):97-104. doi:10.1016/j.biopsych.2005.03.041 | wrong P - dosage and/or duration not clearly stated |
| NIBS | Hoffman 2013 | Hoffman RE, Wu K, Pittman B, et al. Transcranial magnetic stimulation of Wernicke's and Right homologous sites to curtail "voices": a randomized trial. Biol Psychiatry. 2013;73(10):1008-1014. doi:10.1016/j.biopsych.2013.01.016 | not ECT or TMS |
| NIBS | Holi 2004 | Holi MM, Eronen M, Toivonen K, Toivonen P, Marttunen M, Naukkarinen H. Left prefrontal repetitive transcranial magnetic stimulation in schizophrenia. Schizophr Bull. 2004;30(2):429-434. doi:10.1093/oxfordjournals.schbul.a007089 | wrong P - dosage and/or duration not clearly stated |
| NIBS | Hu 2019 | 胡浩浩, et al. 重复经颅磁刺激治疗精神分裂症顽固性幻听的临床效果. 中国民康医学. 2019. | From less experienced countries |
| NIBS | Huang 2013 | 黄继伟, 周刚柱. 重复经颅磁刺激治疗精神分裂症顽固性幻听30例疗效分析. Journal of Chinese Practical Diagnosis and Therapy. 2013. | From less experienced countries |
| NIBS | Ithal 2020 | Ithal D, Arumugham SS, Kumar CN, Venkatapura RJ, Thirthalli J, Gangadhar BN. Comparison of cognitive adverse effects and efficacy of 2 pulse widths (0.5 ms and 1.5 ms) of brief pulse bilateral electroconvulsive therapy in patients with schizophrenia - A randomized single blind controlled trial. Schizophr Res. 2020;216:520-522. doi:10.1016/j.schres.2019.11.062 | wrong P - dosage and/or duration not clearly stated |
| NIBS | Jandl 2006 | Jandl M, Steyer J, Weber M, et al. Treating auditory hallucinations by transcranial magnetic stimulation: a randomized controlled cross-over trial. Neuropsychobiology. 2006;53(2):63-69. doi:10.1159/000091721 | wrong design - less than 3 weeks |
| NIBS | Jiang 2017 | 姜洪亮. 低频重复经颅磁刺激治疗精神分裂症顽固性幻听的随机双盲对照研究. 2017. | From less experienced countries |
| NIBS | Kantrowitz 2019 | Kantrowitz JT, Sehatpour P, Avissar M, et al. Significant improvement in treatment resistant auditory verbal hallucinations after 5 days of double-blind, randomized, sham controlled, fronto-temporal, transcranial direct current stimulation (tDCS): A replication/extension study. Brain Stimul. 2019;12(4):981-991. doi:10.1016/j.brs.2019.03.003 | not ECT or TMS |
| NIBS | Kim 2014 | Kim EJ, Yeo S, Hwang I, et al. Bilateral Repetitive Transcranial Magnetic Stimulation for Auditory Hallucinations in Patients with Schizophrenia: A Randomized Controlled, Cross-over Study. Clin Psychopharmacol Neurosci. 2014;12(3):222-228. doi:10.9758/cpn.2014.12.3.222 | wrong design - less than 3 weeks |
| NIBS | Kimura 2016 | Kimura H, Kanahara N, Takase M, Yoshida T, Watanabe H, Iyo M. A randomized, sham-controlled study of high frequency rTMS for auditory hallucination in schizophrenia. Psychiatry Res. 2016;241:190-194. doi:10.1016/j.psychres.2016.04.119 | wrong design - less than 3 weeks |
| NIBS | Kindler 2013a | Kindler J, Homan P, Flury R, Strik W, Dierks T, Hubl D. Theta burst transcranial magnetic stimulation for the treatment of auditory verbal hallucinations: results of a randomized controlled study. Psychiatry Res. 2013;209(1):114-117. doi:10.1016/j.psychres.2013.03.029 | wrong C - other active comparators |
| NIBS | Kindler 2013b | Kindler J, Homan P, Jann K, et al. Reduced neuronal activity in language-related regions after transcranial magnetic stimulation therapy for auditory verbal hallucinations. Biol Psychiatry. 2013;73(6):518-524. doi:10.1016/j.biopsych.2012.06.019 | no usable data |
| NIBS | Kisku 2021 | Kisku RR. EFFICACY OF EXTENDED REPETITIVE TRANSCRANIAL MAGNETIC STIMULATION IN PERSISTENT AUDITORY HALLUCINATIONS IN SCHIZOPHRENIA AS MEASURED BY CEREBRAL OXYGEN GRADIENT: A RANDOMIZED SHAM CONTROLLED STUDY USING FUNCTIONAL NEAR INFRARED SPECTROSCOPY. Dissertation. 2021. | not ECT or TMS |
| NIBS | Klein 1999 | Klein E, Kolsky Y, Puyerovsky M, Koren D, Chistyakov A, Feinsod M. Right prefrontal slow repetitive transcranial magnetic stimulation in schizophrenia: a double-blind sham-controlled pilot study. Biol Psychiatry. 1999;46(10):1451-1454. doi:10.1016/s0006-3223(99)00182-1 | wrong P - duration not adequate |
| NIBS | Klirova 2013 | Klirova M, Horacek J, Novak T, et al. Individualized rTMS neuronavigated according to regional brain metabolism ((18)FGD PET) has better treatment effects on auditory hallucinations than standard positioning of rTMS: a double-blind, sham-controlled study. Eur Arch Psychiatry Clin Neurosci. 2013;263(6):475-484. doi:10.1007/s00406-012-0368-x | wrong design - less than 3 weeks |
| NIBS | Koops 2016 | Koops S, van Dellen E, Schutte MJ, Nieuwdorp W, Neggers SF, Sommer IE. Theta Burst Transcranial Magnetic Stimulation for Auditory Verbal Hallucinations: Negative Findings From a Double-Blind-Randomized Trial. Schizophr Bull. 2016;42(1):250-257. doi:10.1093/schbul/sbv100 | wrong P - duration not adequate |
| NIBS | Koops 2018 | Koops S, Blom JD, Bouachmir O, Slot MI, Neggers B, Sommer IE. Treating auditory hallucinations with transcranial direct current stimulation in a double-blind, randomized trial. Schizophr Res. 2018;201:329-336. doi:10.1016/j.schres.2018.06.010 | not ECT or TMS |
| NIBS | Laurin 2020 | Laurin A, et al. Effects of the transcranial magnetic stimulation on the sense of agency and body ownership impairments in schizophrenia with first-rank symptoms. European Neuropsychopharmacology. 2020. | wrong P - dosage and/or duration not clearly stated |
| NIBS | Lee 2005 | Lee SH, Kim W, Chung YC, et al. A double blind study showing that two weeks of daily repetitive TMS over the left or right temporoparietal cortex reduces symptoms in patients with schizophrenia who are having treatment-refractory auditory hallucinations. Neurosci Lett. 2005;376(3):177-181. doi:10.1016/j.neulet.2004.11.048 | wrong design - less than 3 weeks |
| NIBS | Li 2013 | 李梅英. 重复经颅磁刺激治疗精神分裂症顽固性幻听对照研究. Chinese Journal of Practical Nervous Diseases. 2013. | From less experienced countries |
| NIBS | Li 2015 | 李艳红, et al. 低频重复经颅磁刺激治疗精神分裂症顽固性幻听疗效观察. J Clin Psychosom Dis. 2015. | From less experienced countries |
| NIBS | Li 2019 | Li MZ, Chen LC, Rong H, et al. Low-charge electrotherapy for patients with schizophrenia: A double-blind, randomised controlled pilot clinical trial. Psychiatry Res. 2019;272:676-681. doi:10.1016/j.psychres.2018.12.143 | From less experienced countries |
| NIBS | Lindenmayer 2019 | Lindenmayer JP, Kulsa MKC, Sultana T, et al. Transcranial direct-current stimulation in ultra-treatment-resistant schizophrenia. Brain Stimul. 2019;12(1):54-61. doi:10.1016/j.brs.2018.10.002 | not ECT or TMS |
| NIBS | Liu 2017 | 刘合懿, et al. 氯氮平联合MECT 治疗难治性精神分裂症的疗效分析. 2017. | From less experienced countries |
| NIBS | Loo 2010 | Loo CK, Sainsbury K, Mitchell P, Hadzi-Pavlovic D, Sachdev PS. A sham-controlled trial of left and right temporal rTMS for the treatment of auditory hallucinations. Psychol Med. 2010;40(4):541-546. doi:10.1017/S0033291709990900 | not ECT or TMS |
| NIBS | Lu 2014 | 路亚洲, et al. 20Hz重复经颅磁刺激治疗改善难治性精神分裂症注意和执行功能的损害. China Journal of Health Psychology. 2014. | From less experienced countries |
| NIBS | Maity 2021 | Maity M. TMS-EEG and Serum BDNF During Augmentation of Clozapine with tDCS, in Clozapine Resistant Schizophrenia: A Double Blinded Sham Controlled Study. Disseration. 2021. | not ECT or TMS |
| NIBS | Marquardt 2022 | Marquardt L, Craven AR, Hugdahl K, et al. Pilot-RCT Finds No Evidence for Modulation of Neuronal Networks of Auditory Hallucinations by Transcranial Direct Current Stimulation. Brain Sci. 2022;12(10):1382. Published 2022 Oct 12. doi:10.3390/brainsci12101382 | not ECT or TMS |
| NIBS | McIntosh 2004 | McIntosh AM, Semple D, Tasker K, et al. Transcranial magnetic stimulation for auditory hallucinations in schizophrenia. Psychiatry Res. 2004;127(1-2):9-17. doi:10.1016/j.psychres.2004.03.005 | wrong P - dosage and/or duration not clearly stated |
| NIBS | Mellin 2018 | Mellin JM, Alagapan S, Lustenberger C, et al. Randomized trial of transcranial alternating current stimulation for treatment of auditory hallucinations in schizophrenia. Eur Psychiatry. 2018;51:25-33. doi:10.1016/j.eurpsy.2018.01.004 | not ECT or TMS |
| NIBS | Mondino 2016 | Mondino M, Jardri R, Suaud-Chagny MF, Saoud M, Poulet E, Brunelin J. Effects of Fronto-Temporal Transcranial Direct Current Stimulation on Auditory Verbal Hallucinations and Resting-State Functional Connectivity of the Left Temporo-Parietal Junction in Patients With Schizophrenia. Schizophr Bull. 2016;42(2):318-326. doi:10.1093/schbul/sbv114 | wrong design - subgroup analysis of an already included trial |
| NIBS | Moulier 2021 | Moulier V, Krir MW, Dalmont M; SURECT Group, Guillin O, Rothärmel M. A prospective multicenter assessor-blinded randomized controlled study to compare the efficacy of short versus long protocols of electroconvulsive therapy as an augmentation strategy to clozapine in patients with ultra-resistant schizophrenia (SURECT study). Trials. 2021;22(1):284. Published 2021 Apr 15. doi:10.1186/s13063-021-05227-3 | not ECT or TMS |
| NIBS | NCT05282329 | Efficacy of tACS for Treatment of Auditory Hallucinations in Refractory Schizophrenia | not ECT or TMS |
| NIBS | Nelson 2015 | Nelson BG, et al. Transcranial Current Stimulation for the Treatment of Medication Refractory Auditory Hallucinations. International Congress on Schizophrenia Research. 2015. | not ECT or TMS |
| NIBS | Paillere-Martinot 2017 | Paillère-Martinot ML, Galinowski A, Plaze M, et al. Active and placebo transcranial magnetic stimulation effects on external and internal auditory hallucinations of schizophrenia. Acta Psychiatr Scand. 2017;135(3):228-238. doi:10.1111/acps.12680 | wrong P - dosage and/or duration not clearly stated |
| NIBS | Parlikar 2023 | Parlikar R, et al. Neurobiological and clinical effects of High-Definition tDCS on persistent auditory hallucinations in schizophrenia: A randomized controlled trial medRxiv 2023.05.10.23289796; doi: https://doi.org/10.1101/2023.05.10.23289796 | not ECT or TMS |
| NIBS | Plewnia 2018 | Plewnia C, Brendel B, Schwippel T, et al. Treatment of auditory hallucinations with bilateral theta burst stimulation (cTBS): protocol of a randomized, double-blind, placebo-controlled, multicenter trial. Eur Arch Psychiatry Clin Neurosci. 2018;268(7):663-673. doi:10.1007/s00406-017-0861-3 | wrong design - protocol only |
| NIBS | Potapov 2022 | Potapov I, Maslenikov N, Tsukarzi E, Mosolov S. Randomized comparative study of 1-Hz transcranial magnetic stimulation (TMS), continuous theta-burst stimulation (cTBS) and sham-TMS for treatment-refractory auditory hallucinations (AH) in schizophrenia. European Psychiatry. 2022;65(S1):S737-S737. doi:10.1192/j.eurpsy.2022.1903 | wrong P - dosage and/or duration not clearly stated |
| NIBS | Poulet 2005 | Poulet E, Brunelin J, Bediou B, et al. Slow transcranial magnetic stimulation can rapidly reduce resistant auditory hallucinations in schizophrenia. Biol Psychiatry. 2005;57(2):188-191. doi:10.1016/j.biopsych.2004.10.007 | not ECT or TMS |
| NIBS | Pu 2013 | 蒲绮霞, et al. 低频重复经颅磁刺激治疗顽固性幻听的疗效及对脑源性神经营养因子的影响. Guangdong Medical Journal. 2013. | From less experienced countries |
| NIBS | Purohith 2020 | Continuous theta-burst stimulation(cTBS). Conference abstract. | wrong P |
| NIBS | Quan 2012 | 权文香, et al. 低频重复经颅磁刺激治疗精神分裂症患者的顽 固性幻听. Chinese Mental Health Journal. 2012. | From less experienced countries |
| NIBS | Ren 2010 | 任艳萍, et al. 低频重复经颅磁刺激治疗精神分裂症顽 固性幻听的随机双盲对照研究. 2010. | From less experienced countries |
| NIBS | Rosa 2007 | Rosa MO, Gattaz WF, Rosa MA, et al. Effects of repetitive transcranial magnetic stimulation on auditory hallucinations refractory to clozapine. J Clin Psychiatry. 2007;68(10):1528-1532. doi:10.4088/jcp.v68n1009 | wrong design - less than 3 weeks |
| NIBS | Rosenberg 2012 | Rosenberg O, Gersner R, Klein LD, Kotler M, Zangen A, Dannon P. Deep transcranial magnetic stimulation add-on for the treatment of auditory hallucinations: a double-blind study. Ann Gen Psychiatry. 2012;11:13. Published 2012 May 6. doi:10.1186/1744-859X-11-13 | wrong P - dosage and/or duration not clearly stated |
| NIBS | Schonfeldt-Lecuona 2004 | Schönfeldt-Lecuona C, Grön G, Walter H, et al. Stereotaxic rTMS for the treatment of auditory hallucinations in schizophrenia. Neuroreport. 2004;15(10):1669-1673. doi:10.1097/01.wnr.0000126504.89983.ec | not ECT or TMS |
| NIBS | Slotema 2012 | Slotema CW, Blom JD, de Weijer AD, Hoek HW, Sommer IE. Priming does not enhance the efficacy of 1 Hertz repetitive transcranial magnetic stimulation for the treatment of auditory verbal hallucinations: results of a randomized controlled study. Brain Stimul. 2012;5(4):554-559. doi:10.1016/j.brs.2011.10.005 | wrong C - other active comparators |
| NIBS | Sun 2018 | 孙丛丛, et al. 重复经颅磁刺激治疗难治性精神分裂症的增效作用研究. Journal of Capital Medical University. 2018. | From less experienced countries |
| NIBS | Suonmaa 2021 | Suonmaa E. Transcranial Direct Current Stimulation (tDCS) for severely ill schizophrenia patients. Brain Stimulation. 2021. | not ECT or TMS |
| NIBS | Taylor 1980 | Taylor P, Fleminger JJ. ECT for schizophrenia. Lancet. 1980;1(8183):1380-1382. doi:10.1016/s0140-6736(80)92653-7 | wrong P - dosage and/or duration not clearly stated |
| NIBS | Thomas 2019 | Thomas F, Bouaziz N, Gallea C, et al. Structural and functional brain biomarkers of clinical response to rTMS of medication-resistant auditory hallucinations in schizophrenia patients: study protocol for a randomized sham-controlled double-blind clinical trial. Trials. 2019;20(1):229. Published 2019 Apr 23. doi:10.1186/s13063-019-3311-x | wrong P - dosage and/or duration not clearly stated |
| NIBS | Tuppurainen 2020 | Tuppurainen H, et al. P.414 Neuronav igated and indiv idualized alpha frequency transcranial magnetic stimulation in treatmentrefractory schizophrenia. European Neuropsychopharmacology. 2020. https://doi.org/10.1016/j.euroneuro.2020.09.307 | wrong P - dosage and/or duration not clearly stated |
| NIBS | Tyagi 2022 | Tyagi P, Dhyani M, Khattri S, Tejan V, Tikka SK, Garg S. "Efficacy of intensive bilateral Temporo-Parietal Continuous theta-burst Stimulation for Auditory VErbal hallucinations (TPC-SAVE) in schizophrenia: A randomized sham-controlled trial"☆. Asian J Psychiatr. 2022;74:103176. doi:10.1016/j.ajp.2022.103176 | wrong design - less than 3 weeks |
| NIBS | Vercammen 2010 | Vercammen A, Knegtering H, Liemburg EJ, den Boer JA, Aleman A. Functional connectivity of the temporo-parietal region in schizophrenia: effects of rTMS treatment of auditory hallucinations. J Psychiatr Res. 2010;44(11):725-731. doi:10.1016/j.jpsychires.2009.12.011 | wrong design - subgroup analysis of an already included trial |
| NIBS | Wang 2016 | 王雪, et al. 低频重复经颅磁刺激对难治性精神分裂症的增效作用. 2016. | From less experienced countries |
| NIBS | Wang 2021 | 王璐, et al. The Clinical Efficacy of precise rTMS and its Neural Mechanism in Treatment-Refractory Schizophrenia. Dissertation. 2021. | From less experienced countries |
| NIBS | Weijer 2014 | de Weijer AD, Sommer IE, Lotte Meijering A, et al. High frequency rTMS; a more effective treatment for auditory verbal hallucinations?. Psychiatry Res. 2014;224(3):204-210. doi:10.1016/j.pscychresns.2014.10.007 | wrong C - other active comparators |
| NIBS | Wen-Xiang 2012 | 权文香, et al. 低频重复经颅磁刺激治疗精神分裂症患者的顽 固性幻听. Chinese Mental Health Journal. 2012. | From less experienced countries |
| NIBS | Xie 2023 | Xie Y, Guan M, He Y, et al. The Static and dynamic functional connectivity characteristics of the left temporoparietal junction region in schizophrenia patients with auditory verbal hallucinations during low-frequency rTMS treatment. Front Psychiatry. 2023;14:1071769. Published 2023 Jan 25. doi:10.3389/fpsyt.2023.1071769 | From less experienced countries |
| NIBS | Xu 2011 | 徐亚秋, et al. 低频重复经颅磁刺激治疗精神分裂症顽固性幻听的临床研究. 2011. | From less experienced countries |
| NIBS | Xu 2020 | 许珮玮, et al. 超难治性精神分裂症的MST 与MECT 平行对照探索性研究. 2020. Dissertation. | From less experienced countries |
| NIBS | Yang 2022 | 杨菊, et al. 利培酮联合低频重复经颅磁刺激治疗精神分裂症顽固性幻听的效果及对事件相关电位的影响. 2022. | From less experienced countries |
| NIBS | Yue 2013 | 岳莉莉, et al. 低频重复经颅磁刺激对精神分裂症患者顽固性幻听的疗效分析. Clin J Med Office. 2013. | From less experienced countries |
| NIBS | Zhang 2010_NIBS | 张轶杰, et al. 无抽搐电痉挛治疗合并氯氮平治疗难治性精神分裂症及其对记忆力的影响. Chinese Mental Health Journal. 2010. | From less experienced countries |
| NIBS | Zhang 2014 | 张翠红, et al. 低频重复经颅磁刺激对精神分裂症顽固性幻听的随机双盲对照研究. Medical Journal of Chinese People's Health. 2014. | From less experienced countries |
| NIBS | Zhang 2016 | 张少霞, et al. 抗精神病药物联合低频重复经颅磁刺激治疗对精神分裂症顽固性幻听的临床对照研究. China &Foreign Medical Treatment. 2016. | From less experienced countries |
| NIBS | Zhang 2022 | Zhang M, Force RB, Walker C, Ahn S, Jarskog LF, Frohlich F. Alpha transcranial alternating current stimulation reduces depressive symptoms in people with schizophrenia and auditory hallucinations: a double-blind, randomized pilot clinical trial. Schizophrenia (Heidelb). 2022;8(1):114. Published 2022 Dec 24. doi:10.1038/s41537-022-00321-0 | not ECT or TMS |
| NIBS | Zhu 2013 | 朱琳, 刘少华. 低频重复经颅磁刺激治疗精神分裂症难治性幻听的疗效. Journal of Gannan Medical University. 2013. | From less experienced countries |

## List of included trials

| **Study Name** | **Reference** |
| --- | --- |
| Anil Yagcioglu 2005 | Anil Yağcioğlu AE, Kivircik Akdede BB, Turgut TI, et al. A double-blind controlled study of adjunctive treatment with risperidone in schizophrenic patients partially responsive to clozapine: efficacy and safety. J Clin Psychiatry. 2005;66(1):63-72. doi:10.4088/jcp.v66n0109 |
| ARISE study | https://news.bms.com/news/details/2025/Bristol-Myers-Squibb-Announces-Topline-Results-from-Phase-3-ARISE-Trial-Evaluating-Cobenfy-xanomeline-and-trospium-chloride-as-an-Adjunctive-Treatment-to-Atypical-Antipsychotics-in-Adults-with-Schizophrenia/default.aspx　(Accessed on 25. April. 2025) |
| Assion 2008 | Assion HJ, Reinbold H, Lemanski S, Basilowski M, Juckel G. Amisulpride augmentation in patients with schizophrenia partially responsive or unresponsive to clozapine. A randomized, double-blind, placebo-controlled trial. Pharmacopsychiatry. 2008;41(1):24-28. doi:10.1055/s-2007-993209 |
| Azorin 2001 | Azorin JM, Spiegel R, Remington G, et al. A double-blind comparative study of clozapine and risperidone in the management of severe chronic schizophrenia. Am J Psychiatry. 2001;158(8):1305-1313. doi:10.1176/appi.ajp.158.8.1305 |
| Barnes 2017 | Barnes TR, Leeson VC, Paton C, et al. Amisulpride augmentation in clozapine-unresponsive schizophrenia (AMICUS): a double-blind, placebo-controlled, randomised trial of clinical effectiveness and cost-effectiveness. Health Technol Assess. 2017;21(49):1-56. doi:10.3310/hta21490 |
| Bitter 2004 | Bitter I, Dossenbach MR, Brook S, et al. Olanzapine versus clozapine in treatment-resistant or treatment-intolerant schizophrenia. Prog Neuropsychopharmacol Biol Psychiatry. 2004;28(1):173-180. doi:10.1016/j.pnpbp.2003.09.033 |
| Blumberger 2012 | Blumberger DM, Christensen BK, Zipursky RB, et al. MRI-targeted repetitive transcranial magnetic stimulation of Heschl's gyrus for refractory auditory hallucinations. Brain Stimul. 2012;5(4):577-585. doi:10.1016/j.brs.2011.12.002 |
| Bondolfi 1998 | Bondolfi G, Dufour H, Patris M, et al. Risperidone versus clozapine in treatment-resistant chronic schizophrenia: a randomized double-blind study. The Risperidone Study Group. Am J Psychiatry. 1998;155(4):499-504. doi:10.1176/ajp.155.4.499 |
| Breier 1999 | Breier AF, Malhotra AK, Su TP, et al. Clozapine and risperidone in chronic schizophrenia: effects on symptoms, parkinsonian side effects, and neuroendocrine response. Am J Psychiatry. 1999;156(2):294-298. doi:10.1176/ajp.156.2.294 |
| Buchanan 1998 | Buchanan RW, Breier A, Kirkpatrick B, Ball P, Carpenter WT Jr. Positive and negative symptom response to clozapine in schizophrenic patients with and without the deficit syndrome. Am J Psychiatry. 1998;155(6):751-760. doi:10.1176/ajp.155.6.751 |
| Cather 2005 | Cather C, Penn D, Otto MW, Yovel I, Mueser KT, Goff DC. A pilot study of functional Cognitive Behavioral Therapy (fCBT) for schizophrenia. Schizophr Res. 2005;74(2-3):201-209. doi:10.1016/j.schres.2004.05.002 |
| Chang 2008 | Chang JS, Ahn YM, Park HJ, et al. Aripiprazole augmentation in clozapine-treated patients with refractory schizophrenia: an 8-week, randomized, double-blind, placebo-controlled trial. J Clin Psychiatry. 2008;69(5):720-731. doi:10.4088/jcp.v69n0505 |
| Chowdhury 1999 | Chowdhury AN, Mukherjee A, Ghosh K, Chowdhury S, Das Sen K. Horizon of a new hope: recovery of schizophrenia in India. International medical journal, 1999, 6(3), 181‐185 |
| Durham 2003 | Durham RC, Guthrie M, Morton RV, et al. Tayside-Fife clinical trial of cognitive-behavioural therapy for medication-resistant psychotic symptoms. Results to 3-month follow-up. Br J Psychiatry. 2003;182:303-311. doi:10.1192/bjp.182.4.303 |
| Fleischhacker 2010 | Fleischhacker WW, Heikkinen ME, Olié JP, et al. Effects of adjunctive treatment with aripiprazole on body weight and clinical efficacy in schizophrenia patients treated with clozapine: a randomized, double-blind, placebo-controlled trial. Int J Neuropsychopharmacol. 2010;13(8):1115-1125. doi:10.1017/S1461145710000490 |
| Freudenreich 2007 | Freudenreich O, Henderson DC, Walsh JP, Culhane MA, Goff DC. Risperidone augmentation for schizophrenia partially responsive to clozapine: a double-blind, placebo-controlled trial. Schizophr Res. 2007;92(1-3):90-94. doi:10.1016/j.schres.2006.12.030 |
| Friedman 2011 | Friedman JI, Lindenmayer JP, Alcantara F, et al. Pimozide augmentation of clozapine inpatients with schizophrenia and schizoaffective disorder unresponsive to clozapine monotherapy [published correction appears in Neuropsychopharmacology. 2011 May;36(6):1317. Kaushik, Saurabh [added]] [published correction appears in Neuropsychopharmacology. 2011 Sep;36(10):2150. Novakovick, Vladan [corrected to Novakovic, Vladan]]. Neuropsychopharmacology. 2011;36(6):1289-1295. doi:10.1038/npp.2011.14 |
| Goswami 2003 | Goswami U, Kumar U, Singh B. Efficacy of Electroconvulsive Therapy in Treatment Resistant Schizophreinia : A double-blind study. Indian J Psychiatry. 2003;45(1):26-29. |
| Gunduz-Bruce 2013 | Gunduz-Bruce H, Oliver S, Gueorguieva R, et al. Efficacy of pimozide augmentation for clozapine partial responders with schizophrenia. Schizophr Res. 2013;143(2-3):344-347. doi:10.1016/j.schres.2012.11.008 |
| Honer 2006 | Honer WG, Thornton AE, Chen EY, et al. Clozapine alone versus clozapine and risperidone with refractory schizophrenia. N Engl J Med. 2006;354(5):472-482. doi:10.1056/NEJMoa053222 |
| Honer 2010 | Honer WG, MacEwan GW, Gendron A, et al. A randomized, double-blind, placebo-controlled study of the safety and tolerability of high-dose quetiapine in patients with persistent symptoms of schizophrenia or schizoaffective disorder. J Clin Psychiatry. 2012;73(1):13-20. doi:10.4088/JCP.10m06194 |
| Hong 1997 | Hong CJ, Chen JY, Chiu HJ, Sim CB. A double-blind comparative study of clozapine versus chlorpromazine on Chinese patients with treatment-refractory schizophrenia. Int Clin Psychopharmacol. 1997;12(3):123-130. doi:10.1097/00004850-199705000-00001 |
| Huang 1987 | Huang CC, Gerhardstein RP, Kim DY, Hollister L. Treatment-resistant schizophrenia: controlled study of moderate- and high-dose thiothixene. Int Clin Psychopharmacol. 1987;2(1):69-75. doi:10.1097/00004850-198701000-00006 |
| Jesus 2011 | de Jesus DR, Gil A, Barbosa L, et al. A pilot double-blind sham-controlled trial of repetitive transcranial magnetic stimulation for patients with refractory schizophrenia treated with clozapine. Psychiatry Res. 2011;188(2):203-207. doi:10.1016/j.psychres.2010.11.022 |
| Josiassen 2005 | Josiassen RC, Joseph A, Kohegyi E, et al. Clozapine augmented with risperidone in the treatment of schizophrenia: a randomized, double-blind, placebo-controlled trial. Am J Psychiatry. 2005;162(1):130-136. doi:10.1176/appi.ajp.162.1.130 |
| Kane 1988 | Kane J, Honigfeld G, Singer J, Meltzer H. Clozapine for the treatment-resistant schizophrenic. A double-blind comparison with chlorpromazine. Arch Gen Psychiatry. 1988;45(9):789-796. doi:10.1001/archpsyc.1988.01800330013001 |
| Kane 2001 | Kane JM, Marder SR, Schooler NR, et al. Clozapine and haloperidol in moderately refractory schizophrenia: a 6-month randomized and double-blind comparison. Arch Gen Psychiatry. 2001;58(10):965-972. doi:10.1001/archpsyc.58.10.965 |
| Kane 2009 | Kane JM, Correll CU, Goff DC, et al. A multicenter, randomized, double-blind, placebo-controlled, 16-week study of adjunctive aripiprazole for schizophrenia or schizoaffective disorder inadequately treated with quetiapine or risperidone monotherapy. J Clin Psychiatry. 2009;70(10):1348-1357. doi:10.4088/JCP.09m05154yel |
| Kinon 1993 | Kinon BJ, Kane JM, Johns C, et al. Treatment of neuroleptic-resistant schizophrenic relapse. Psychopharmacol Bull. 1993;29(2):309-314. |
| Lindenmayer 2011 | Lindenmayer JP, Citrome L, Khan A, Kaushik S, Kaushik S. A randomized, double-blind, parallel-group, fixed-dose, clinical trial of quetiapine at 600 versus 1200 mg/d for patients with treatment-resistant schizophrenia or schizoaffective disorder. J Clin Psychopharmacol. 2011;31(2):160-168. doi:10.1097/JCP.0b013e31820f4fe0 |
| Meltzer 2008 | Meltzer HY, Bobo WV, Roy A, et al. A randomized, double-blind comparison of clozapine and high-dose olanzapine in treatment-resistant patients with schizophrenia. J Clin Psychiatry. 2008;69(2):274-285. doi:10.4088/jcp.v69n0214 |
| Meltzer 2020 | Meltzer HY, Share DB, Jayathilake K, Salomon RM, Lee MA. Lurasidone Improves Psychopathology and Cognition in Treatment-Resistant Schizophrenia. J Clin Psychopharmacol. 2020;40(3):240-249. doi:10.1097/JCP.0000000000001205 |
| Melzer 2023 | Melzer-Ribeiro DL, Napolitano IC, Leite SA, et al. Randomized, double-blind, sham-controlled trial to evaluate the efficacy and tolerability of electroconvulsive therapy in patients with clozapine-resistant schizophrenia. Schizophr Res. 2024;268:252-260. doi:10.1016/j.schres.2023.11.009 |
| Melzer-Ribeiro 2017 | Melzer-Ribeiro DL, Rigonatti SP, Kayo M, et al. / Arch Clin Psychiatry. 2017;44(2):45-50 |
| Mishra 2022 | Mishra BR, Agrawal K, Biswas T, Mohapatra D, Nath S, Maiti R. Comparison of Acute Followed by Maintenance ECT vs Clozapine on Psychopathology and Regional Cerebral Blood Flow in Treatment-Resistant Schizophrenia: A Randomized Controlled Trial. Schizophr Bull. 2022;48(4):814-825. doi:10.1093/schbul/sbac027 |
| Moresco 2004 | Moresco RM, Cavallaro R, Messa C, et al. Cerebral D2 and 5-HT2 receptor occupancy in Schizophrenic patients treated with olanzapine or clozapine. J Psychopharmacol. 2004;18(3):355-365. doi:10.1177/026988110401800306 |
| Morrison 2018 (FOCUS) | Morrison AP, Pyle M, Gumley A, et al. Cognitive behavioural therapy in clozapine-resistant schizophrenia (FOCUS): an assessor-blinded, randomised controlled trial. Lancet Psychiatry. 2018;5(8):633-643. doi:10.1016/S2215-0366(18)30184-6 |
| Mossaheb 2006 | Mossaheb N, Sacher J, Wiesegger G, et al. P.3.c.037 Haloperidol in combination with clozapine in treatment-refractory patients with schizophrenia. European Neuropsychopharmacology. 2006. https://doi.org/10.1016/S0924-977X(06)70524-7 |
| Muscatello 2011 | Muscatello MR, Bruno A, Pandolfo G, et al. Effect of aripiprazole augmentation of clozapine in schizophrenia: a double-blind, placebo-controlled study. Schizophr Res. 2011;127(1-3):93-99. doi:10.1016/j.schres.2010.12.011 |
| Muscatello 2014 | Muscatello MR, Pandolfo G, Micò U, et al. Augmentation of clozapine with ziprasidone in refractory schizophrenia: a double-blind, placebo-controlled study. J Clin Psychopharmacol. 2014;34(1):129-133. doi:10.1097/JCP.0000000000000042 |
| Nielsen 2012 | Nielsen RE, Levander S, Thode D, Nielsen J. Effects of sertindole on cognition in clozapine-treated schizophrenia patients. Acta Psychiatr Scand. 2012;126(1):31-39. doi:10.1111/j.1600-0447.2012.01840.x |
| Petrides 2015 | Petrides G, Malur C, Braga RJ, et al. Electroconvulsive therapy augmentation in clozapine-resistant schizophrenia: a prospective, randomized study. Am J Psychiatry. 2015;172(1):52-58. doi:10.1176/appi.ajp.2014.13060787 |
| Pinto 1999 | Pinto A, La Pia S, Mennella R, Giorgio D, DeSimone L. Cognitive-behavioral therapy and clozapine for clients with treatment-refractory schizophrenia. Psychiatr Serv. 1999;50(7):901-904. doi:10.1176/ps.50.7.901 |
| Plewnia 2014 | Plewnia C, Zwissler B, Wasserka B, Fallgatter AJ, Klingberg S. Treatment of auditory hallucinations with bilateral theta burst stimulation: a randomized controlled pilot trial. Brain Stimul. 2014;7(2):340-341. doi:10.1016/j.brs.2014.01.001 |
| Plewnia 2025 | Plewnia C, Brendel B, Schwippel T, et al. Theta burst stimulation of temporo-parietal cortex regions for the treatment of persistent auditory hallucinations: a multicentre, randomised, sham-controlled, triple-blind phase 3 trial in Germany. Lancet Psychiatry. 2025;12(9):638-649. doi:10.1016/S2215-0366(25)00202-0 |
| Rosenheck 1997 | Rosenheck R, Cramer J, Xu W, et al. A comparison of clozapine and haloperidol in hospitalized patients with refractory schizophrenia. Department of Veterans Affairs Cooperative Study Group on Clozapine in Refractory Schizophrenia. N Engl J Med. 1997;337(12):809-815. doi:10.1056/NEJM199709183371202 |
| Sacchetti 2009 | Sacchetti E, Galluzzo A, Valsecchi P, et al. Ziprasidone vs clozapine in schizophrenia patients refractory to multiple antipsychotic treatments: the MOZART study [published correction appears in Schizophr Res. 2010 Aug;121(1-3)281. multiple investigator names added]. Schizophr Res. 2009;113(1):112-121. doi:10.1016/j.schres.2009.05.002 |
| Sakurai 2016 | Sakurai H, Suzuki T, Bies RR, et al. Increasing Versus Maintaining the Dose of Olanzapine or Risperidone in Schizophrenia Patients Who Did Not Respond to a Modest Dosage: A Double-Blind Randomized Controlled Trial. J Clin Psychiatry. 2016;77(10):1381-1390. doi:10.4088/JCP.15m10490 |
| Salganik 1998 | Salganik I, Modai I, Bercovici BR, Kutzuk D, Weizman A. Clozapine vs haloperidol therapy in elderly chronic schizophrenic inpatients - Preliminary results. A double-blind, cross-over randomized study. International journal of geriatric psychopharmacology, 1998, 1(4), 185‐187 |
| Schooler 2016 | Schooler NR, Marder SR, Chengappa KN, et al. Clozapine and risperidone in moderately refractory schizophrenia: a 6-month randomized double-blind comparison. J Clin Psychiatry. 2016;77(5):628-634. doi:10.4088/JCP.13m08351 |
| Sensky 2000 | Sensky T, Turkington D, Kingdon D, et al. A randomized controlled trial of cognitive-behavioral therapy for persistent symptoms in schizophrenia resistant to medication. Arch Gen Psychiatry. 2000;57(2):165-172. doi:10.1001/archpsyc.57.2.165 |
| Shiloh 1997 | Shiloh R, Zemishlany Z, Aizenberg D, et al. Sulpiride augmentation in people with schizophrenia partially responsive to clozapine. A double-blind, placebo-controlled study. Br J Psychiatry. 1997;171:569-573. doi:10.1192/bjp.171.6.569 |
| Slotema 2011 | Slotema CW, Blom JD, de Weijer AD, et al. Can low-frequency repetitive transcranial magnetic stimulation really relieve medication-resistant auditory verbal hallucinations? Negative results from a large randomized controlled trial. Biol Psychiatry. 2011;69(5):450-456. doi:10.1016/j.biopsych.2010.09.051 |
| Tollefson 2001 | Tollefson GD, Birkett MA, Kiesler GM, Wood AJ; Lilly Resistant Schizophrenia Study Group. Double-blind comparison of olanzapine versus clozapine in schizophrenic patients clinically eligible for treatment with clozapine. Biol Psychiatry. 2001;49(1):52-63. doi:10.1016/s0006-3223(00)01026-x |
| Valmaggia 2005 | Valmaggia LR, van der Gaag M, Tarrier N, Pijnenborg M, Slooff CJ. Cognitive-behavioural therapy for refractory psychotic symptoms of schizophrenia resistant to atypical antipsychotic medication. Randomised controlled trial. Br J Psychiatry. 2005;186:324-330. doi:10.1192/bjp.186.4.324 |
| Volavka 2002 | Volavka J, Czobor P, Sheitman B, et al. Clozapine, olanzapine, risperidone, and haloperidol in the treatment of patients with chronic schizophrenia and schizoaffective disorder [published correction appears in Am J Psychiatry 2002 Dec;159(12):2132]. Am J Psychiatry. 2002;159(2):255-262. doi:10.1176/appi.ajp.159.2.255 |
| Wahlbeck 2000 | Wahlbeck K, Cheine M, Tuisku K, Ahokas A, Joffe G, Rimón R. Risperidone versus clozapine in treatment-resistant schizophrenia: a randomized pilot study. Prog Neuropsychopharmacol Biol Psychiatry. 2000;24(6):911-922. doi:10.1016/s0278-5846(00)00118-4 |
| Weiner 2010 | Weiner E, Conley RR, Ball MP, et al. Adjunctive risperidone for partially responsive people with schizophrenia treated with clozapine. Neuropsychopharmacology. 2010;35(11):2274-2283. doi:10.1038/npp.2010.101 |
| Woo 2022 | Woo YS, Park SY, Yoon BH, Choi WS, Wang SM, Bahk WM. Amisulpride Augmentation in Schizophrenia Patients with Poor Response to Olanzapine: A 4-week, Randomized, Rater-Blind, Controlled, Pilot Study. Clin Psychopharmacol Neurosci. 2022;20(3):567-572. doi:10.9758/cpn.2022.20.3.567 |

## Study and patient characteristics

| Study name | Treatment | Previous antipsychotic | New intervention | Dose (mg) | N | N of females | Mean age (year) | Diagnostic criteria | Min no of AP trials | Required treatment duration (week) | Dosage definition |
| --- | --- | --- | --- | --- | --- | --- | --- | --- | --- | --- | --- |
| ARISE study | Antipsychotic continuation | SGA > 80% | AP + Placebo |  | 196 | 71 | 41.7 | DSM-5 | 1 | 6 | dosed appropriately (within the label) |
|  | Xanomeline-trospium augmentation |  | AP + Xanomeline-trospium |  | 190 | 70 | 41.5 |  |  |  |  |
| Anil Yagcioglu 2005 | Antipsychotic continuation | Clozapine  (mean dose 414mg) | Clozapine + Placebo |  | 14 | 3 | 31.2 | DSM-IV | 3 | 24 | CP 500mg or more |
|  | Combining antipsychotics | (mean dose 516mg) | Clozapine + Risperidone | 2-6 | 16 | 7 | 35.3 |  |  |  |  |
| Assion 2008 | Antipsychotic continuation | Clozapine  (mean plasma level 285ng/mL) | Clozapine + Placebo | 0 | 3 | 0 | 46.3 | DSM-IV | 3 | 12 | CP 500mg or more |
|  | Combining antipsychotics | (mean plasma level 256ng/mL) | Clozapine + Amisulpride | 400 | 7 | 1 | 43.0 |  |  |  |  |
|  | Combining antipsychotics | (mean plasma level 268ng/mL) | Clozapine + Amisulpride | 600 | 6 | 3 | 41.5 |  |  |  |  |
| Azorin 2001 | Switching to clozapine | n.i. | Clozapine | 200-900 | 138 | 32 | 37.8 | DSM-IV | 2 | 6 | CP 500mg or more |
|  | Switching to non-clozapine |  | Risperidone | 2-15 | 135 | 46 | 39.5 |  |  |  |  |
| Barnes 2017 | Antipsychotic continuation | Clozapine  (400mg or more) | Clozapine + Placebo |  | 33 | 10 | 39.5 |  | 3 | 12 | CP 500mg or more |
|  | Combining antipsychotics |  | Clozapine + Amisulpride | 400-800 | 35 | 11 | 39.5 |  |  |  |  |
| Bitter 2004 | Switching to clozapine | n.i. | Clozapine | 100-500 | 72 | 29 | 37.4 | DSM-IV | 1 | 4 | CP 300-500mg |
|  | Switching to non-clozapine |  | Olanzapine | 5-25 | 75 | 30 | 37.6 |  |  |  |  |
| Blumberger 2012 | Antipsychotic continuation | SGA > 80% | AP + sham rTMS |  | 17 |  | 40.8 | DSM-IV | 2 | 6 | "adequate" |
|  | TMS augmentation |  | AP + rTMS |  | 17 |  | 36.6 |  |  |  |  |
|  | TMS augmentation |  | AP + rTMS |  | 17 |  | 43.8 |  |  |  |  |
| Bondolfi 1998 | Switching to clozapine | n.i. | Clozapine | 150-400 | 43 | 11 | 36.2 | DSM-III-R | 2 | 4 | "adequate" |
|  | Switching to non-clozapine |  | Risperidone | 3-10 | 43 | 14 | 38.3 |  |  |  |  |
| Breier 1999 | Switching to clozapine | FGA > 80% | Clozapine | 200-600 | 14 | 6 | 37.7 | DSM-IV | 2 | 6 | "adequate" |
|  | Switching to non-clozapine |  | Risperidone | 2-9 | 15 | 4 | 32.4 |  |  |  |  |
| Buchanan 1998 | Switching to clozapine | FGA > 80% | Clozapine | 200-600 | 38 | 13 | 41.0 | DSM-III-R | 3 | 6 | CP 500mg or more |
|  | Switching to non-clozapine |  | Haloperidol | 10-30 | 37 | 10 | 40.1 |  |  |  |  |
| Cather 2005 | Antipsychotic continuation | SGA > 80% | AP + Psychoeducation |  | 14 | 6 | 40.4 | DSM-IV | 1 | 24 | everybody was taking antipsychotics and the mean dose was adequate |
|  | CBTp augmentation |  | AP + Cognitive behavioral therapy |  | 16 | 7 | 40.4 |  |  |  |  |
| Chang 2008 | Antipsychotic continuation | Clozapine  (mean dose 291mg) | Clozapine + Placebo |  | 32 | 6 | 31.7 | DSM-IV | 3 | 50 | CP 500mg or more |
|  | Combining antipsychotics | (mean dose 304mg) | Clozapine + Aripiprazole | 5-30 | 29 | 7 | 33.2 |  |  |  |  |
| Chowdhury 1999 | Switching to clozapine | FGA > 80% | Clozapine | 200-500 | 30 | 8 | 30.3 | ICD-10 | 1 | 6 | CP 500mg or more |
|  | Switching to non-clozapine |  | Risperidone | 4-8 | 30 | 7 | 32.4 |  |  |  |  |
| Durham 2003 | Antipsychotic continuation | n.i. | AP + Supportive therapy |  | 23 | 8 | 37.0 | ICD-10 and DSM-IV | 1 | 24 | everybody was taking antipsychotics and the mean dose was adequate |
|  | Antipsychotic continuation |  | AP + treatment as usual |  | 21 | 6 | 36.0 |  |  |  |  |
|  | CBTp augmentation |  | AP +  Cognitive behavioral therapy |  | 22 | 7 | 36.0 |  |  |  |  |
| Fleischhacker 2010 | Antipsychotic continuation | Clozapine  (mean dose 363mg) | Clozapine + Placebo |  | 99 | 33 | 40.5 | DSM-IV-TR | 3 | 12 | CP 300-500mg |
|  | Combining antipsychotics | (mean dose 384mg) | Clozapine + Aripiprazole | 5-15 | 108 | 40 | 37.6 |  |  |  |  |
| Freudenreich 2007 | Antipsychotic continuation | Clozapine  (mean dose 456mg) | Clozapine + Placebo |  | 13 | 2 | 42.3 | DSM-IV | 3 | 24 | "adequate" |
|  | Combining antipsychotics |  | Clozapine + Risperidone | 4 | 11 | 1 | 42.3 |  |  |  |  |
| Friedman 2011 | Antipsychotic continuation | Clozapine  (mean dose 478mg) | Clozapine + Placebo |  | 28 | 6 | 44.9 | DSM-IV | 3 | 4 | "adequate" |
|  | Combining antipsychotics | (mean dose 519mg) | Clozapine + Pimozide | 2-8 | 25 | 6 | 44.9 |  |  |  |  |
| Goswami 2003 | Antipsychotic continuation | n.i. | AP + sham ECT |  | 10 | 3 | 29.1 | DSM-IV | 2 | 6 | CP 500mg or more |
|  | ECT augmentation |  | AP + ECT |  | 15 | 6 | 29.8 |  |  |  |  |
| Gunduz-Bruce 2013 | Antipsychotic continuation | Clozapine  (mean plasma level 471 ng/mL) | Clozapine + Placebo |  | 14 | 4 | 42.9 | DSM-IV | 3 | 4 | "adequate" |
|  | Combining antipsychotics | (mean plasma level 642 ng/mL) | Clozapine + Pimozide | 1-4 | 14 | 4 | 42.9 |  |  |  |  |
| Honer 2006 | Antipsychotic continuation | Clozapine  (mean dose 487mg) | Clozapine + Placebo |  | 34 | 9 | 34.9 | DSM-IV | 3 | 12 | CP 500mg or more |
|  | Combining antipsychotics | (mean dose 494mg) | Clozapine + Risperidone | 1-3 | 34 | 9 | 39.4 |  |  |  |  |
| Honer 2010 | Antipsychotic continuation | Quetiapine | Quetiapine | 800 | 43 | 11 | 37.9 | DSM-IV | 1 | 4 | CP 500mg or more |
|  | Dose-escalation |  | Quetiapine | 1,200 | 88 | 30 | 40.6 |  |  |  |  |
| Hong 1997 | Switching to clozapine | n.i. | Clozapine | 100-900 | 21 | 14 | 39.7 | DSM-IV | 2 | 24 | CP 500mg or more |
|  | Switching to non-clozapine |  | Chlorpromazine | 200-1,800 | 19 | 12 | 37.1 |  |  |  |  |
| Huang 1987 | Antipsychotic continuation | Thiothixene | Moderate dose | 60 | 21 |  |  | DSM-III | 2 | 24 | CP 500mg or more |
|  | Dose-escalation |  | High dose | 400 | 21 |  |  |  |  |  |  |
| Jesus 2011 | Antipsychotic continuation | Clozapine  (median dose 650mg) | AP + sham rTMS |  | 9 | 2 | 36.5 | OPCRIT | 3 | 16 | CP 500mg or more |
|  | TMS augmentation | (median dose 700mg) | AP + rTMS |  | 8 | 3 | 46.0 |  |  |  |  |
| Josiassen 2005 | Antipsychotic continuation | Clozapine  (mean dose 403mg) | Clozapine + Placebo |  | 20 | 4 | 39.9 | DSM-IV | 3 | 12 | CP 500mg or more |
|  | Combining antipsychotics | (mean dose 529mg) | Clozapine + Risperidone | 1-6 | 20 | 1 | 40.8 |  |  |  |  |
| Kane 1988 | Switching to clozapine | FGA > 80% | Clozapine | Up to 900 | 126 | 29 | 36.2 | DSM-III | 2 | 6 | CP 500mg or more |
|  | Switching to non-clozapine |  | Chlorpromazine | Up to 1,800 | 142 | 29 | 35.6 |  |  |  |  |
| Kane 2001 | Switching to clozapine | n.i. | Clozapine | 200-800 | 37 | 11 | 41.0 | DSM-III-R | 2 | 6 | CP 500mg or more |
|  | Switching to non-clozapine |  | Haloperidol | 4-16 | 34 | 10 | 40.0 |  |  |  |  |
| Kane 2009b | Antipsychotic continuation | SGA > 80%  (Risperidone or quetiapine) | AP + Placebo |  | 155 | 56 | 44.4 | DSM-IV-TR | 1 | 4 | CP 300-500mg |
|  | Combining antipsychotics |  | AP + Aripiprazole | 2-15 | 168 | 69 | 44.1 |  |  |  |  |
| Kinon 1993b | Antipsychotic continuation | Fluphenazine 20mg | Fluphenazine | 20 | 18 |  | 29.4 | DSM-III-R | 1 | 4 | CP 500mg or more |
|  | Dose-escalation |  | Fluphenazine | 80 | 16 |  |  |  |  |  |  |
|  | Switching to non-clozapine |  | Haloperidol | 20 | 13 |  |  |  |  |  |  |
| Lindenmayer 2011 | Antipsychotic continuation | Quetiapine | Quetiapine | 600 | 31 | 4 | 41.0 | DSM-IV-TR | 1 | 4 | CP 500mg or more |
|  | Dose-escalation |  | Quetiapine | 1,200 | 29 | 1 | 39.3 |  |  |  |  |
| Meltzer 2008 | Switching to clozapine | n.i. | Clozapine | 300-900 | 21 | 6 | 37.2 | DSM-IV | 2 | 6 | "adequate" |
|  | Switching to non-clozapine |  | Olanzapine | 25-45 | 19 | 7 | 36.4 |  |  |  |  |
| Meltzer 2020 | Antipsychotic continuation | Lurasidone | Lurasidone | 80 | 34 | 20 | 47.0 | DSM-IV-TR | 2 | 6 | CP 500mg or more |
|  | Dose-escalation |  | Lurasidone | 240 | 33 | 18 | 45.2 |  |  |  |  |
| Melzer 2023 | Antipsychotic continuation | Clozapine  (mean dose 663mg) | AP + sham ECT |  | 19 | 4 | 36.6 | DSM-5 | 3 | 12 | "adequate" |
|  | ECT augmentation | (mean dose 633mg) | AP + ECT |  | 21 | 5 | 38.1 |  |  |  |  |
| Melzer-Ribeiro 2017 | Antipsychotic continuation | Clozapine  (mean dose 533mg) | AP + sham ECT |  | 10 | 3 | 37.6 | DSM-IV-TR | 3 | 8 | "adequate" |
|  | ECT augmentation | (mean dose 505mg) | AP + ECT |  | 13 | 4 | 36.6 |  |  |  |  |
| Mishra 2022 | ECT augmentation | n.i. | AP + mECT |  | 30 | 12 | 34.5 |  | 2 | 6 | CP 500mg or more |
|  | Switching to clozapine |  | Clozapine | 250-400 | 30 | 13 | 35.8 |  |  |  |  |
| Moresco 2004 | Switching to clozapine | n.i. | Clozapine | 300-400 | 12 | 3 | 38.3 | DSM-IV | 2 | 6 | CP 500mg or more |
|  | Switching to non-clozapine |  | Olanzapine | 15-20 | 11 | 1 | 34.1 |  |  |  |  |
| Morrison 2018 (FOCUS) | Antipsychotic continuation | Clozapine  (mean dose 400mg) | Clozapine + TAU |  | 245 | 72 | 42.8 | ICD-10 | 3 | 12 | CP 500mg or more |
|  | CBTp augmentation |  | Clozapine + Cognitive behavioral therapy |  | 242 | 66 | 42.2 |  |  |  |  |
| Mossaheb 2006 | Antipsychotic continuation | Clozapine  (mean dose 500mg) | Clozapine + Placebo |  | 5 |  | 32.5 |  | 3 | 6 | "adequate" |
|  | Combining antipsychotics | (mean dose 450mg) | Clozapine + Haloperidol | 4 | 5 |  | 32.5 |  |  |  |  |
| Muscatello 2011 | Antipsychotic continuation | Clozapine  (mean dose 341mg) | Clozapine + Placebo |  | 20 | 6 | 35.3 | DSM-IV | 3 | 50 | CP 500mg or more |
|  | Combining antipsychotics | (mean dose 311mg) | Clozapine + Aripiprazole | 10-15 | 20 | 10 | 39.4 |  |  |  |  |
| Muscatello 2014 | Antipsychotic continuation | Clozapine  (mean dose 463mg) | Clozapine + Placebo |  | 20 | 12 | 33.5 | DSM-IV | 3 | 50 | CP 500mg or more |
|  | Combining antipsychotics | (mean dose 428mg) | Clozapine + Ziprasidone | 80 | 20 | 15 | 36.5 |  |  |  |  |
| Nielsen 2012 | Antipsychotic continuation | Clozapine  (mean dose 435mg) | Clozapine + Placebo | 0 | 25 | 10 | 42.7 | ICD-10 | 3 | 24 | CP 300-500mg |
|  | Combining antipsychotics | (mean dose 394mg) | Clozapine + Sertindole | 16 | 25 | 10 | 41.8 |  |  |  |  |
| Petrides 2015 | Antipsychotic continuation | Clozapine  (mean plasma level 829ng/mL) | Clozapine + TAU |  | 19 | 6 | 42.8 | DSM-IV | 3 | 12 | "adequate" |
|  | ECT augmentation | (mean plasma level 855ng/mL) | Clozapine + ECT |  | 20 | 5 | 35.7 |  |  |  |  |
| Pinto 1999 | Antipsychotic continuation | Clozapine  (mean dose 547mg) | Clozapine + Supportive therapy |  | 21 | 7 | 35.8 | DSM-IV | 2 | 6 | CP 500mg or more |
|  | CBTp augmentation | (mean dose 553mg) | Clozapine + Cognitive behavioral therapy + social skills training |  | 20 | 6 | 33.9 |  |  |  |  |
| Plewnia 2014 | Antipsychotic continuation | n.i. | AP + sham cTBS |  | 8 |  | 48.3 | DSM-IV | 1 | 6 | "adequate" |
|  | TMS augmentation |  | AP + cTBS |  | 8 |  | 45.6 |  |  |  |  |
| Plewnia 2025 | Antipsychotic continuation | n.i. | AP + sham cTBS |  | 68 |  | 36.2 | DSM-5 | 1 | 6 | "adequate" |
|  | TMS augmentation |  | AP + cTBS |  | 70 |  | 36.9 |  |  |  |  |
| Rosenheck 1997 | Switching to clozapine | n.i. | Clozapine | 100-900 | 205 | 3 | 43.2 | DSM-III-R | 2 | 4 | "adequate" |
|  | Switching to non-clozapine |  | Haloperidol | 5-30 | 218 | 7 | 43.9 |  |  |  |  |
| Sacchetti 2009 | Switching to clozapine | n.i. | Clozapine | 250-600 | 74 | 24 | 38.3 | DSM-IV | 2 | 6 | "adequate" |
|  | Switching to non-clozapine |  | Ziprasidone | 80-160 | 73 | 21 | 41.6 |  |  |  |  |
| Sakurai 2016 | Antipsychotic continuation | Risperidone 3mg or Olanzapine 10mg | Continuation |  | 51 | 33 | 50.3 | ICD-10 | 1 | 4 | CP 300-500mg |
|  | Dose-escalation |  | Increment | Double | 52 | 32 | 51.1 |  |  |  |  |
| Salganik 1998 | Switching to clozapine | FGA > 80% | Clozapine | Mean dose 120 |  |  | 66.6 | DSM-III-R | 2 | 6 | CP 500mg or more |
|  | Switching to non-clozapine |  | Haloperidol | Mean dose18 |  |  | 66.6 |  |  |  |  |
| Schooler 2016 | Switching to clozapine | n.i. | Clozapine | 500-800 | 53 | 12 | 42.0 | DSM-IV | 1 | 6 | "adequate" |
|  | Switching to non-clozapine |  | Risperidone | 6-16 | 54 | 11 | 42.0 |  |  |  |  |
| Sensky 2000 | Antipsychotic continuation | n.i. | AP + Befriending |  | 44 | 22 | 40.0 | ICD-10 research and DSM-IV | 2 | 24 | CP 300-500mg |
|  | CBTp augmentation |  | AP + Cognitive behavioral therapy |  | 46 | 15 | 39.0 |  |  |  |  |
| Shiloh 1997 | Antipsychotic continuation | Clozapine  (mean dose 446mg) | Clozapine + Placebo |  | 12 | 4 | 38.9 | DSM-IV | 3 | 12 | "adequate" |
|  | Combining antipsychotics | (mean dose 403mg) | Clozapine + Sulpiride | 600 | 16 | 5 | 38.9 |  |  |  |  |
| Slotema 2011 | Antipsychotic continuation | n.i. | AP + sham rTMS |  | 20 | 10 | 41.0 |  | 2 | 6 | "adequate" |
|  | TMS augmentation |  | AP + rTMS |  | 22 | 6 | 38.0 |  |  |  |  |
|  | TMS augmentation |  | AP + rTMS |  | 20 | 10 | 36.0 |  |  |  |  |
| Tollefson 2001 | Switching to clozapine | n.i. | Clozapine | 200-600 | 90 | 36 | 38.6 | DSM-IV | 2 | 6 | CP 500mg or more |
|  | Switching to non-clozapine |  | Olanzapine | 15-25 | 90 | 29 | 38.6 |  |  |  |  |
| Valmaggia 2005 | Antipsychotic continuation | SGA > 80% | AP + Supportive counselling |  | 26 | 9 | 35.5 | DSM-IV | 2 | 6 | "adequate" |
|  | CBTp augmentation |  | AP + Cognitive behavioral therapy |  | 36 | 8 | 35.4 |  |  |  |  |
| Volavka 2002 | Switching to clozapine | FGA > 80% | Clozapine | 200-800 | 40 | 4 | 42.6 | DSM-IV | 1 | 6 | CP 500mg or more |
|  | Switching to non-clozapine |  | Haloperidol | 10-30 | 37 | 8 | 37.3 |  |  |  |  |
|  | Switching to non-clozapine |  | Olanzapine | 10-40 | 39 | 5 | 41.0 |  |  |  |  |
|  | Switching to non-clozapine |  | Risperidone | 4-16 | 41 | 6 | 42.9 |  |  |  |  |
| Wahlbeck 2000 | Switching to clozapine | n.i. | Clozapine | 400-600 | 11 | 2 | 35.7 | DSM-IV | 2 | 6 | CP 500mg or more |
|  | Switching to non-clozapine |  | Risperidone | 6-10 | 9 | 7 | 36.8 |  |  |  |  |
| Weiner 2010 | Antipsychotic continuation | Clozapine  (mean plasma level 491ng/mL) | Clozapine + Placebo |  | 36 | 10 | 44.1 | DSM-IV | 3 | 24 | "adequate" |
|  | Combining antipsychotics | (mean plasma level 680ng/mL) | Clozapine + Risperidone | 4 | 33 | 11 | 48.3 |  |  |  |  |
| Woo 2022 | Antipsychotic continuation | Olanzapine | Olanzapine |  | 12 | 6 | 47.7 | DSM-5 | 2 | 4 | CP 500mg or more |
|  | Combining antipsychotics |  | Olanzapine + Amisulpride | 800 | 13 | 7 | 47.7 |  |  |  |  |

AP = antipsychotic; CBTp = cognitive behavioral therapy for psychosis; cTBS = continuous Theta Burst Stimulation; CP = chlorpromazine; FGA = first generation antipsychotic; DSM = the Diagnostic and Statistical Manual of Mental Disorders; mECT = modified Electroonvulsive Therapy; rTMS = repetitive Transcranial Magnetic Stimulation; SGA = second generation antipsychotic;

## The revised Cochrane risk of bias for the primary outcome

| **studlab** | **D1** | **D2** | **D3** | **D4** | **D5** | **Overall** |
| --- | --- | --- | --- | --- | --- | --- |
| ARISE study | Low | Low | Low | Low | Low | Low |
| Anil Yagcioglu 2005 | Some concerns | Low | Low | Low | Low | Some concerns |
| Assion 2008 | Some concerns | Some concerns | Low | Some concerns | Low | Some concerns |
| Azorin 2001 | Some concerns | Some concerns | Low | Some concerns | Low | Some concerns |
| Barnes 2017 | Some concerns | Low | Low | Low | Low | Some concerns |
| Bitter 2004 | Low | Low | Low | Low | Low | Low |
| Blumberger 2012 | Some concerns | Low | Low | Low | Low | Some concerns |
| Bondolfi 1998 | Some concerns | Low | Low | Low | Low | Some concerns |
| Breier 1999 | Some concerns | Low | Some concerns | Low | Low | Some concerns |
| Buchanan 1998 | Some concerns | Low | Low | Low | Low | Some concerns |
| Chang 2008 | Low | Low | Low | Low | Low | Low |
| Chowdhury 1999 | Some concerns | Some concerns | Low | Some concerns | Low | Some concerns |
| Durham 2003 | Low | Low | Low | Low | Some concerns | Some concerns |
| Fleischhacker 2010 | Some concerns | Some concerns | Low | Some concerns | Low | Some concerns |
| Freudenreich 2007 | Some concerns | Low | Low | Low | Low | Some concerns |
| Friedman 2011 | Some concerns | Low | Low | Low | Low | Some concerns |
| Goswami 2003 | Low | Low | High | Low | High | High |
| Gunduz-Bruce 2013 | Some concerns | Low | Low | Low | Low | Some concerns |
| Honer 2006 | Some concerns | Low | Low | Low | Low | Some concerns |
| Honer 2010 | Low | Some concerns | Low | Some concerns | Low | Some concerns |
| Hong 1997 | Some concerns | Low | Low | Low | Low | Some concerns |
| Huang 1987 | Some concerns | Some concerns | Some concerns | Some concerns | High | High |
| Jesus 2011 | Low | Low | Some concerns | Low | Some concerns | Some concerns |
| Josiassen 2005 | Some concerns | Low | Low | Low | Low | Some concerns |
| Kane 1988 | Some concerns | Low | Low | Low | Low | Some concerns |
| Kane 2001 | Low | Low | High | Low | Low | High |
| Kane 2009b | Some concerns | Low | Low | Some concerns | Low | Some concerns |
| Kinon 1993b | Some concerns | Some concerns | High | Some concerns | Low | High |
| Lindenmayer 2011 | Some concerns | Low | Low | Low | High | High |
| Meltzer 2008 | Low | Low | Low | Low | Low | Low |
| Meltzer 2020 | Some concerns | Low | Low | Low | Low | Some concerns |
| Melzer 2023 | Low | Low | Low | Low | Low | Low |
| Melzer-Ribeiro 2017 | Some concerns | High | Low | Low | Low | High |
| Mishra 2022 | Some concerns | Low | Low | Low | Low | Some concerns |
| Moresco 2004 | Some concerns | Some concerns | High | Some concerns | Low | High |
| Morrison 2018 (FOCUS) | Low | Low | Low | Low | Low | Low |
| Mossaheb 2006 | Some concerns | Some concerns | Some concerns | Some concerns | Some concerns | High |
| Muscatello 2011 | Some concerns | Low | High | Low | Some concerns | High |
| Muscatello 2014 | Low | Low | Low | Low | Low | Low |
| Nielsen 2012 | Low | Low | Low | Low | Low | Low |
| Petrides 2015 | Some concerns | Some concerns | Low | Low | High | High |
| Pinto 1999 | Some concerns | Some concerns | Low | Some concerns | Some concerns | High |
| Plewnia 2025 | Low | Low | Low | Low | Low | Low |
| Rosenheck 1997 | Some concerns | Low | Low | Low | High | High |
| Sacchetti 2009 | Low | Low | Low | Low | Low | Low |
| Sakurai 2016 | Low | Low | Low | Low | Low | Low |
| Schooler 2016 | Some concerns | Low | High | Low | Some concerns | High |
| Sensky 2000 | Low | Low | Low | Low | Some concerns | Some concerns |
| Shiloh 1997 | Some concerns | Low | Low | Low | Some concerns | Some concerns |
| Tollefson 2001 | Low | Low | Low | Low | Low | Low |
| Valmaggia 2005 | Low | Low | Low | Low | Some concerns | Some concerns |
| Volavka 2002 | Low | Low | Low | Low | Low | Low |
| Wahlbeck 2000 | Low | High | Low | Low | Low | High |
| Weiner 2010 | Some concerns | Some concerns | Low | Some concerns | Low | Some concerns |
| Woo 2022 | Some concerns | High | Low | Low | Low | High |
| D1 = bias due to the randomization process; D2 = deviation from intended intervention; D3 = missing outcome data; D4 = measurement of outcomes; D5 = selection of the reported result | | | | | | |

# 3. ASSESSMENT OF TRANSITIVITY

## Box plots

# Mean age


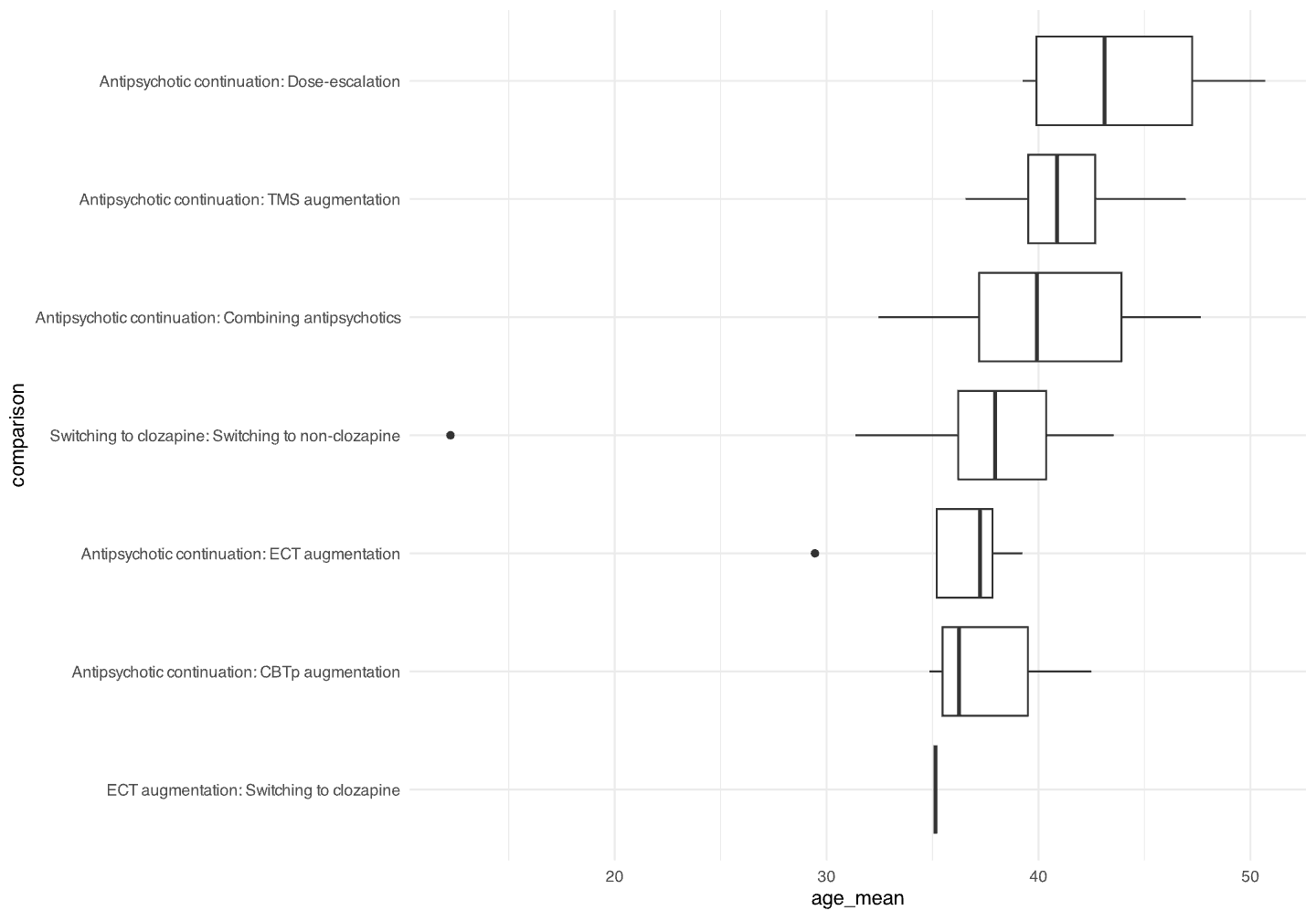


# Proportion of females


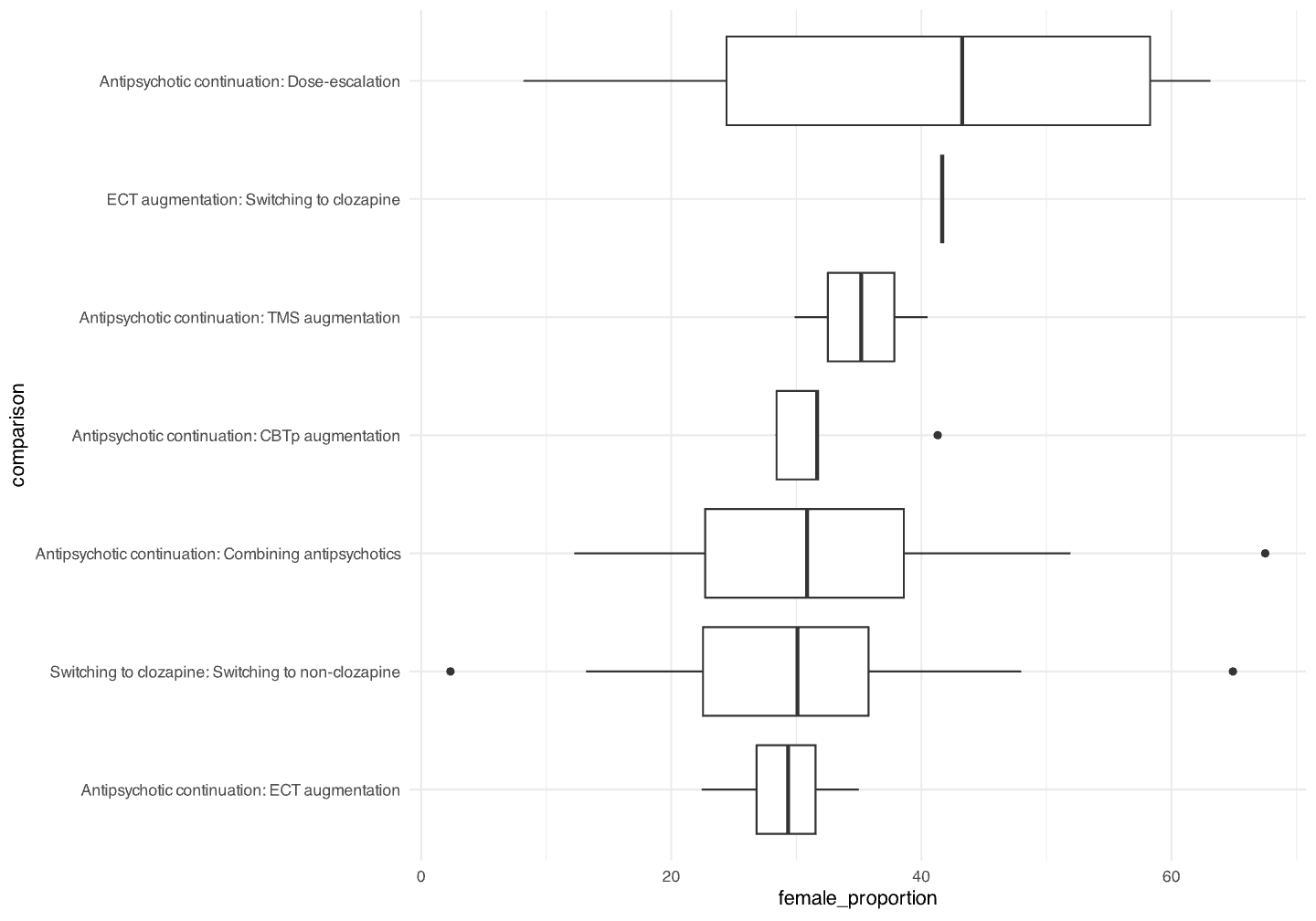
# Baseline severity


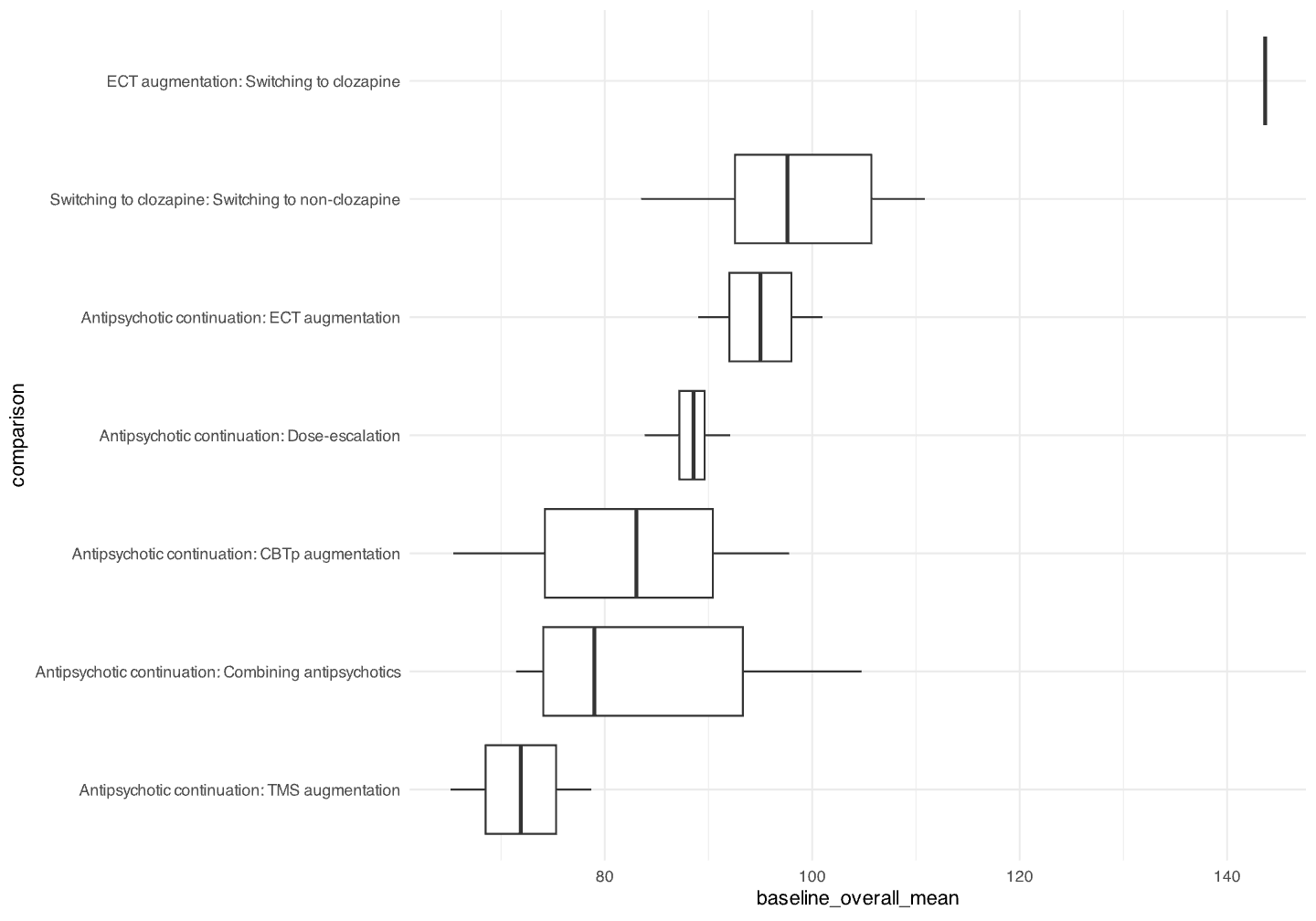


# Duration of illness


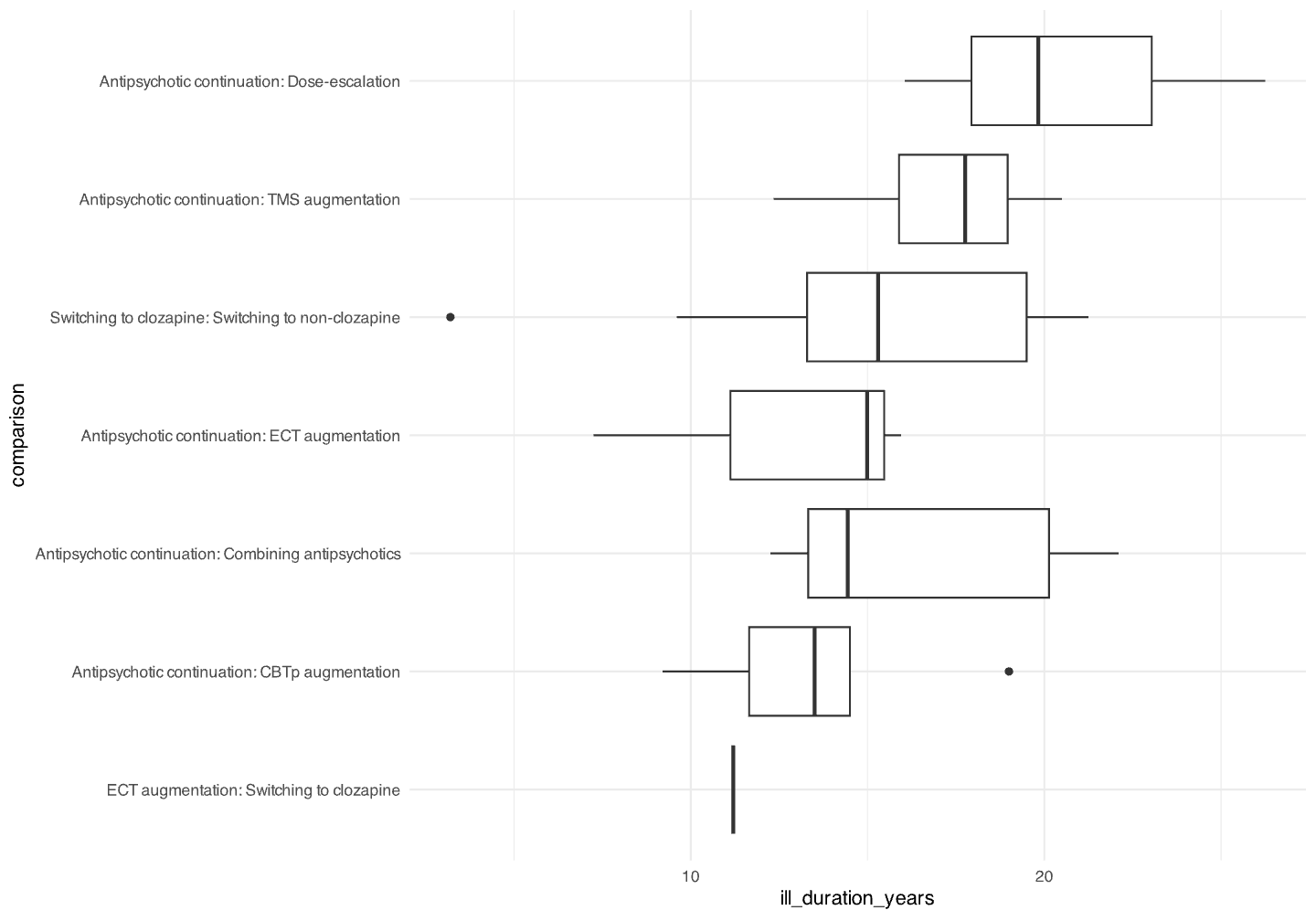
# Number of participants


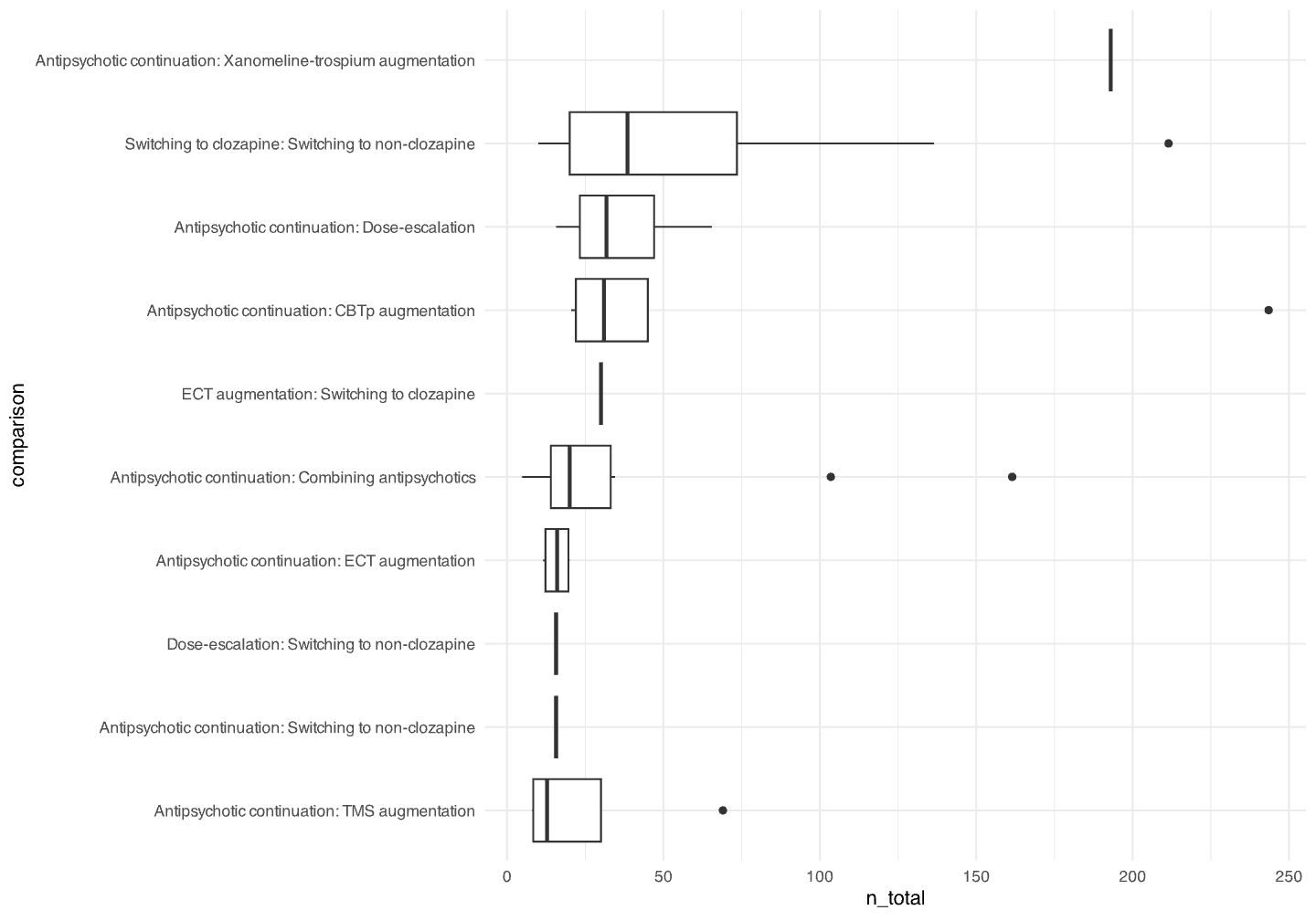


# Publication year


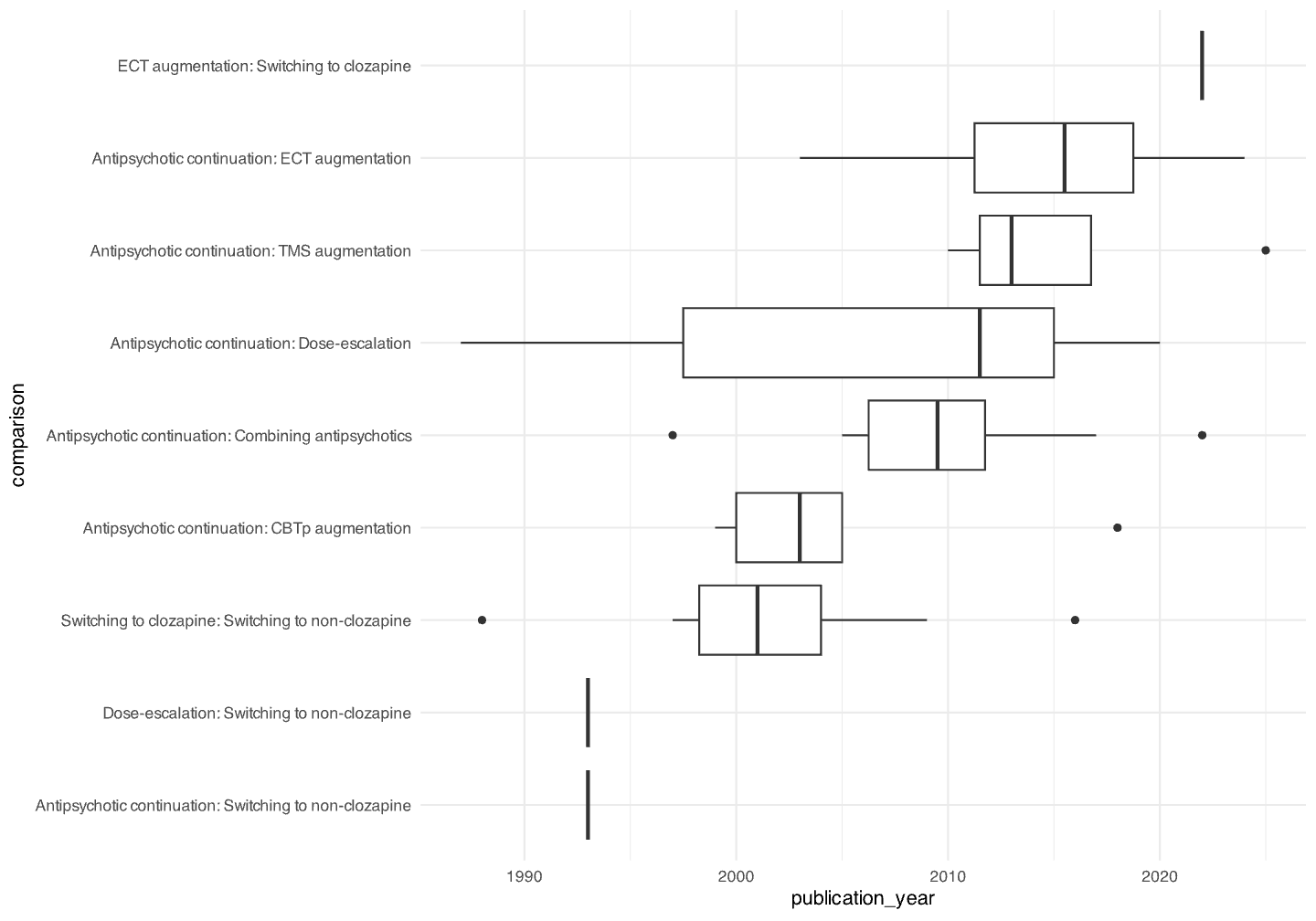
# Blinding type


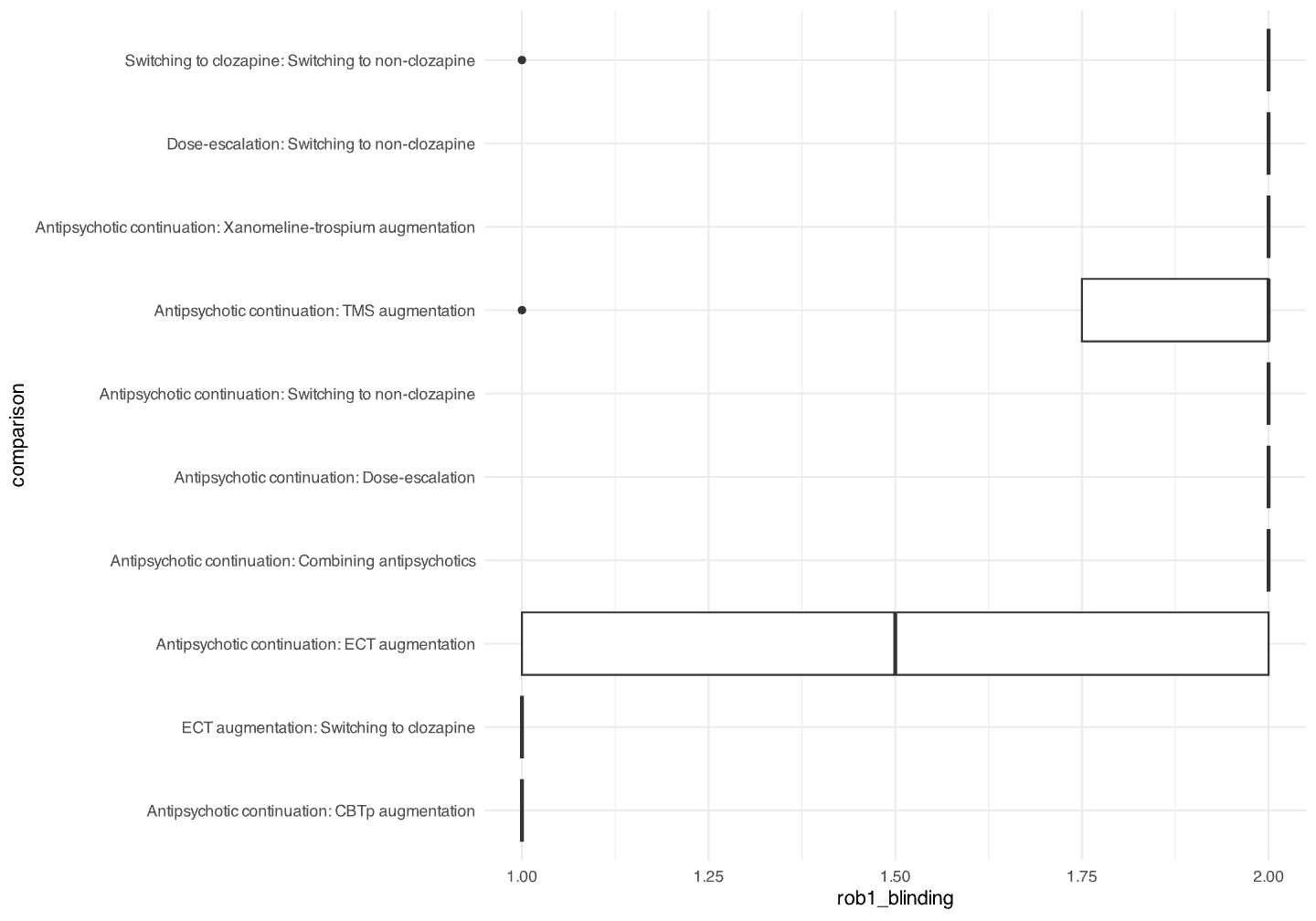


# Trial duration (weeks)


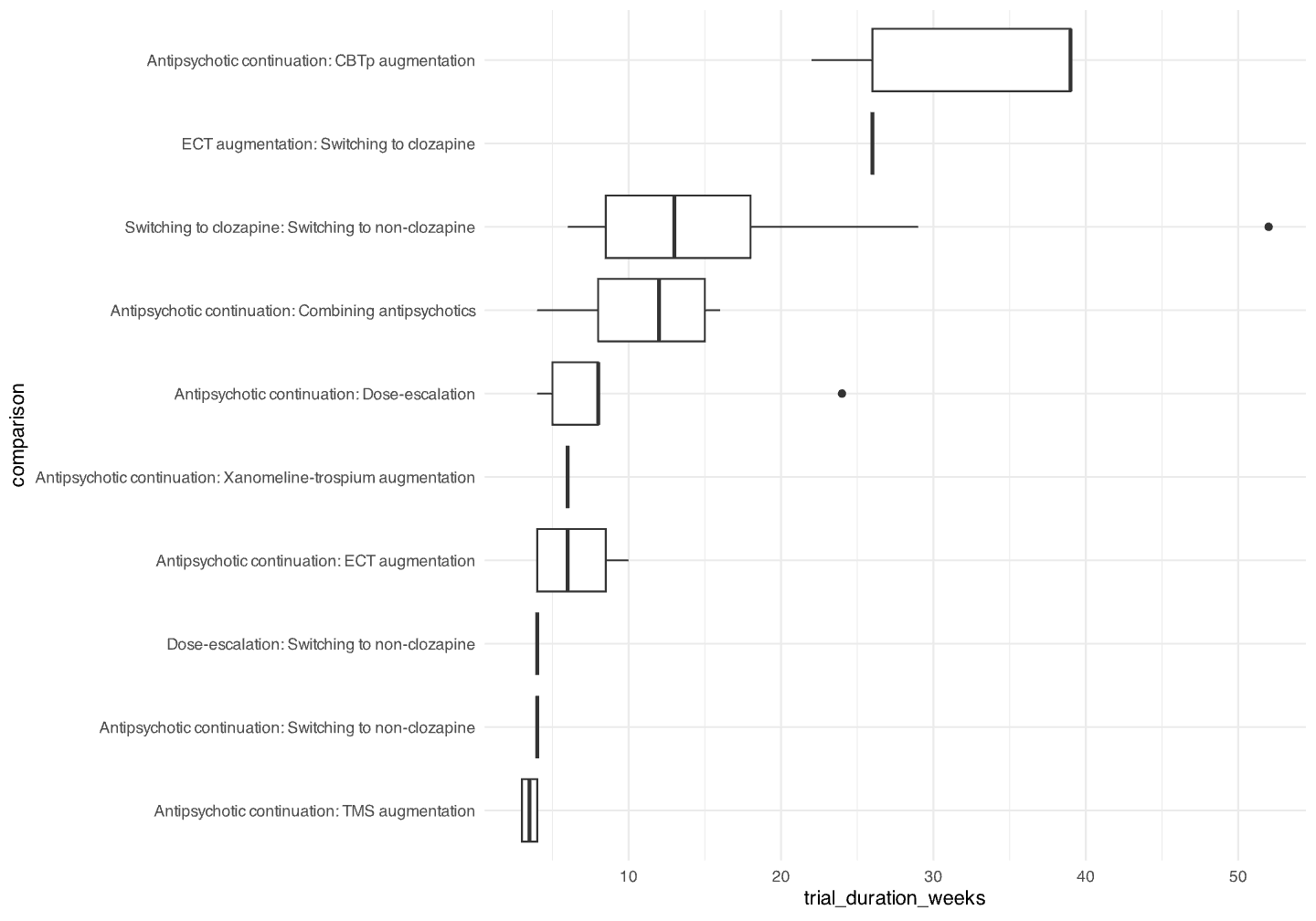
# Minimal antipsychotic trial (the number of antipsychotics)


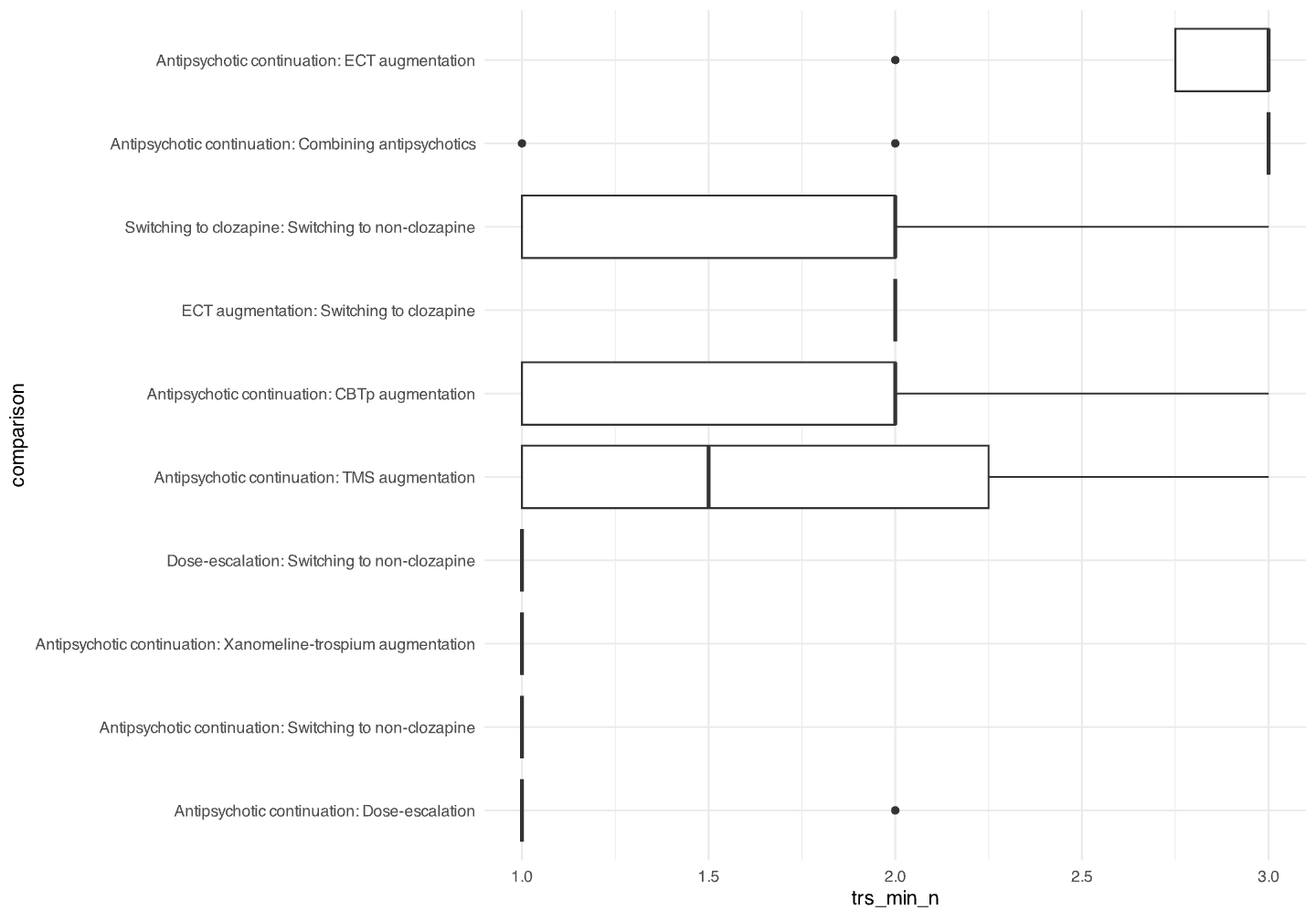


## Global (design-by-treatment) test

Q statistics to assess homogeneity / consistency

Q df p-value

Total 134.43 48 < 0.0001

Within designs 127.30 46 < 0.0001

Between designs 7.13 2 0.0283

Design-specific decomposition of within-designs Q statistic

Design Q df p-value

Switching to clozapine:Switching to non-clozapine 54.77 16 < 0.0001

Antipsychotic continuation:Combining antipsychotics 53.76 17 < 0.0001

Antipsychotic continuation:ECT augmentation 11.42 3 0.0097

Antipsychotic continuation:CBTp augmentation 3.93 4 0.4150

Antipsychotic continuation:TMS augmentation 1.67 2 0.4335

Antipsychotic continuation:Dose-escalation 1.75 4 0.7824

Between-designs Q statistic after detaching of single designs

(influential designs have p-value markedly different from 0.0283)

Detached design Q df p-value

Antipsychotic continuation:ECT augmentation 0.02 1 0.9010

ECT augmentation:Switching to clozapine 0.02 1 0.9010

Switching to clozapine:Switching to non-clozapine 0.02 1 0.9010

Antipsychotic continuation:Dose-escalation 6.61 1 0.0101

Antipsychotic continuation:Dose-escalation:Switching to non-clozapine 0.00 0 --

Q statistic to assess consistency under the assumption of

a full design-by-treatment interaction random effects model

Q df p-value tau.within tau2.within

Between designs 3.55 2 0.1693 0.3062 0.0938

## Local (back-calculation) test

Separate indirect from direct evidence (SIDE) using back-calculation method

Random effects model:

comparison k prop nma direct indir. Diff z p-value

Dose-escalation:Antipsychotic continuation 6 0.99 -0.0580 -0.0952 2.4286 -2.5239 -1.87 0.0619

ECT augmentation:Antipsychotic continuation 4 0.86 -0.5102 -0.3391 -1.5641 1.2250 1.87 0.0619

Switching to non-clozapine:Antipsychotic continuation 1 0.46 0.2157 -0.3228 0.6698 -0.9926 -1.52 0.1283

Dose-escalation:Switching to non-clozapine 1 0.49 -0.2737 0.2122 -0.7495 0.9618 1.40 0.1606

ECT augmentation:Switching to clozapine 1 0.59 -0.6138 -1.1102 0.1148 -1.2250 -1.87 0.0619

Switching to clozapine:Switching to non-clozapine 17 0.98 -0.1121 -0.1386 1.0864 -1.2250 -1.87 0.0619

Legend:

comparison - Treatment comparison

k - Number of studies providing direct evidence

prop - Direct evidence proportion

nma - Estimated treatment effect (SMD) in network meta-analysis

direct - Estimated treatment effect (SMD) derived from direct evidence

indir. - Estimated treatment effect (SMD) derived from indirect evidence

Diff - Difference between direct and indirect treatment estimates

z - z-value of test for disagreement (direct versus indirect)

p-value - p-value of test for disagreement (direct versus indirect)

## Comparing the tau square against empirical distributions

> # Comparing τ2 against empirical distributions-----

> # non-pharma vs pharma in mental health indication

> d1=rnorm(1000000, mean = -1.09, sd= 1.27)

> quantile(exp(d1), c(0.025,0.5, .975))

2.5% 50% 97.5%

0.02789602 0.33653669 4.05721127

> tau2 <- 0.0975

> mean(d1>log(tau2))

[1] 0.835175

- Turner RM, Jackson D, Wei Y, Thompson SG, Higgins JP. Predictive distributions for between-study heterogeneity and simple methods for their application in Bayesian meta-analysis. Stat Med. 2015;34(6):984-998. doi:10.1002/sim.63

# 4. ASSESSMENT OF PUBLICATION BIAS AND SMALL STUDY EFFECTS


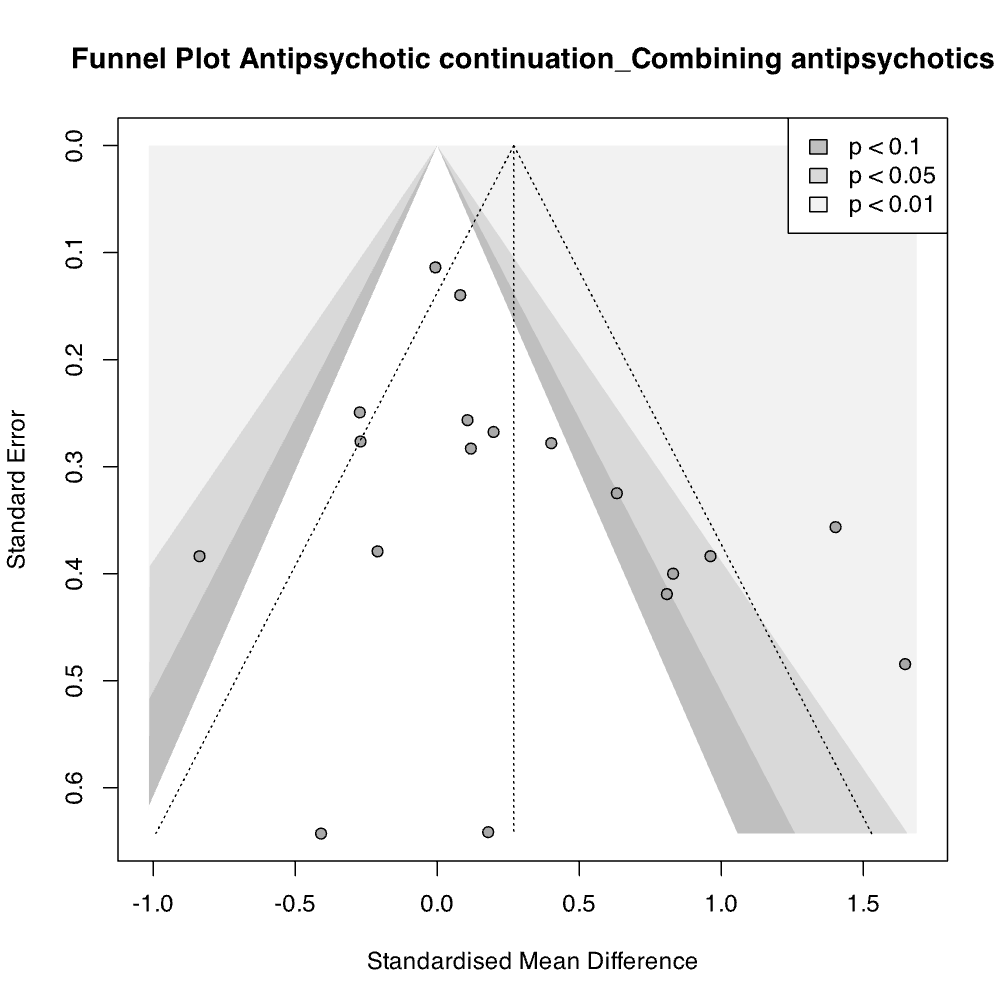

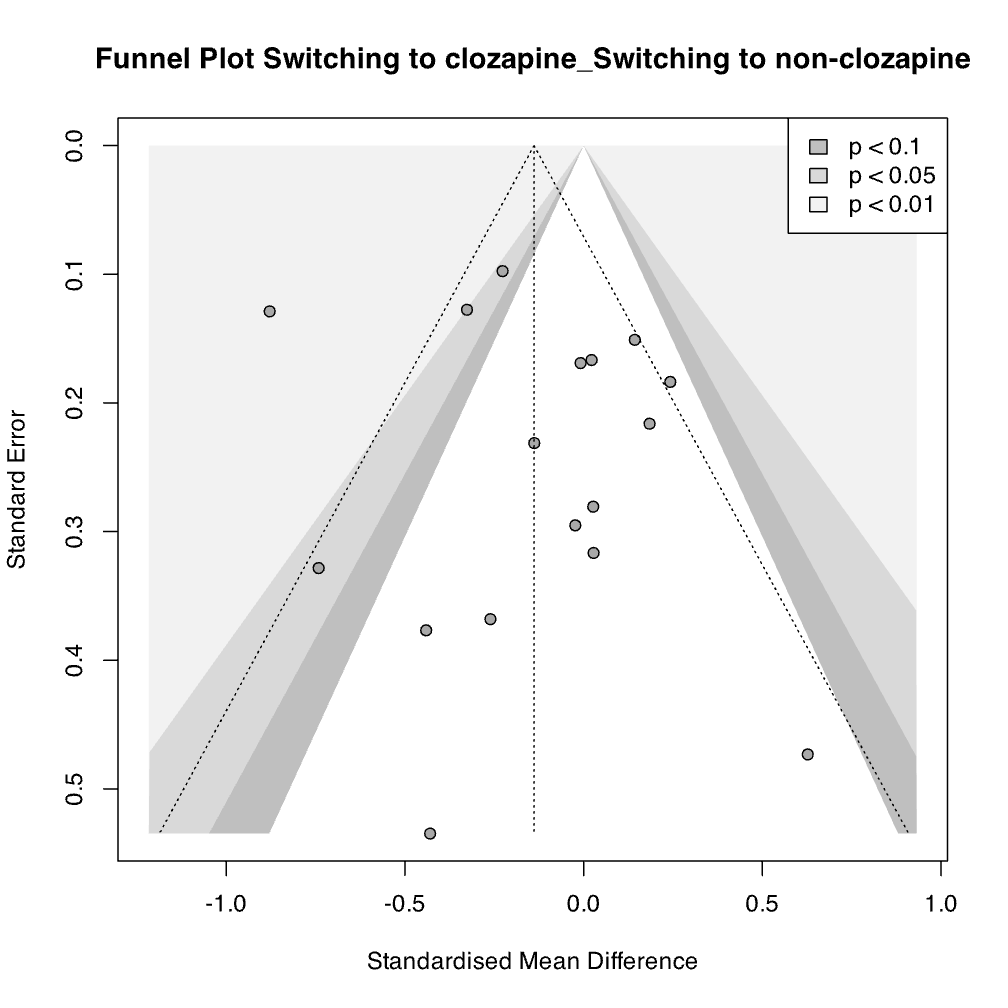


# 5. DIRECT COMPARISONS FOR PRIMARY OUTCOME

Direct estimate and indirect estimates of the network meta-analysis


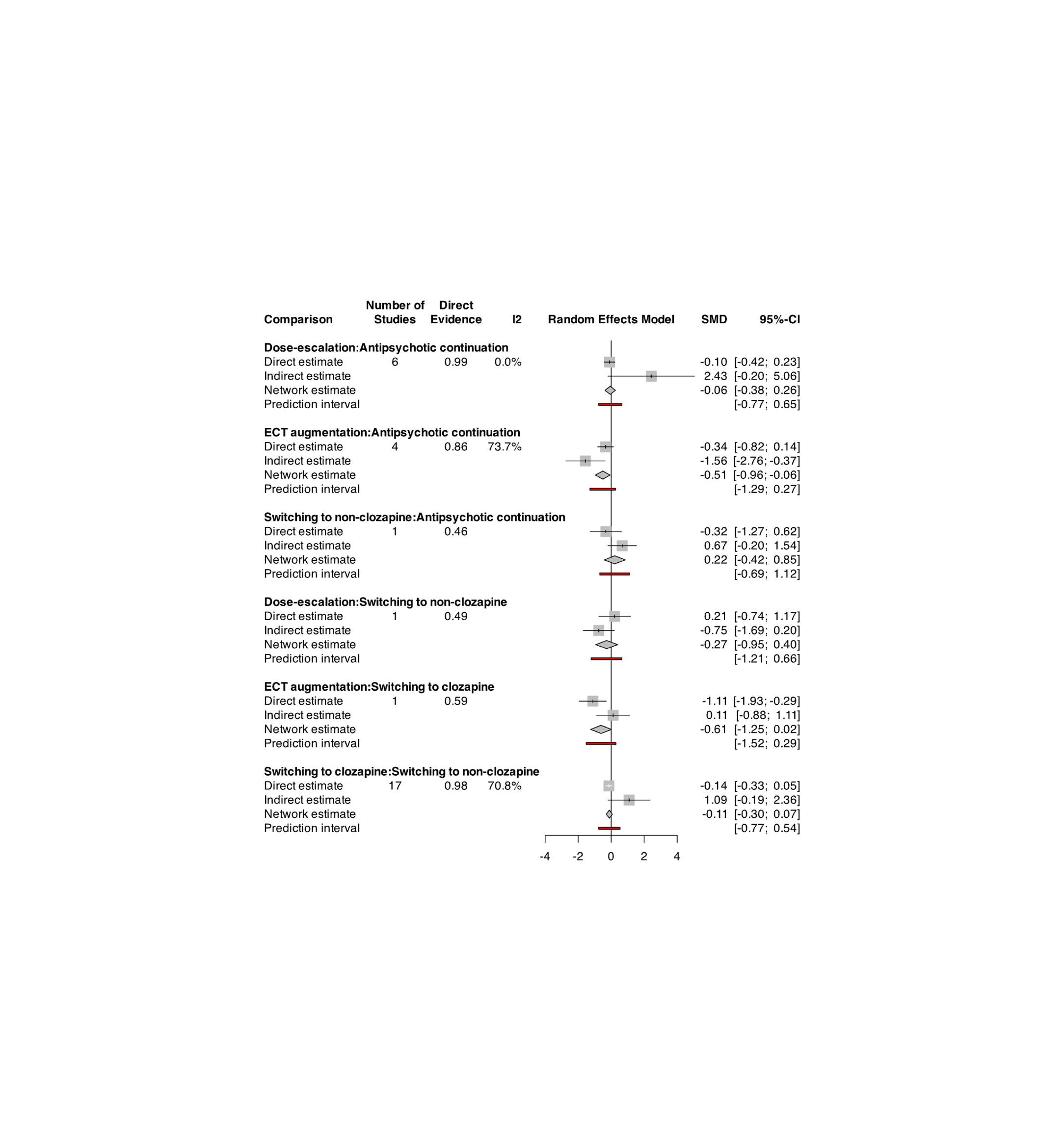


# 6. LEAGUE TABLE FOR THE PRIMARY OUTCOME

CINeMA = Confidence In Network Meta-Analysis. Standardized mean difference (95% confidence interval) of schizophrenia symptoms of the upper-left-defined treatment against down-right-defined treatment. The down-left cells correspond to the network meta-analysis result, while the upper-right cells correspond to the direct pairwise meta-analyses results. Blue indicated moderate confidence in the evidence, yellow low, and red very low according to CINeMA.

# 7. CONFIDENCE IN NETWORK META-ANALYSIS (CINeMA)

We used the Majority RoB for the within-study bias domain. We set the clinically important size of effect as SMD = 0. We downgraded the confidence rating by two levels for each domain with major concerns, and by one level for each domain with some concerns. Given the sparse network connectivity, we considered the confidence in the comparisons based solely on indirect evidence to be very low.

| **Comparison** | **N of studies** | **Within-study bias** | **Reporting bias** | **Indirectness** | **Imprecision** | **Heterogeneity** | **Incoherence** | **Confidence rating** | **Reason(s) for downgrading** |
| --- | --- | --- | --- | --- | --- | --- | --- | --- | --- |
| Antipsychotic continuation:CBTp augmentation | 5 | Some concerns | Low risk | No concerns | Major concerns | No concerns | No concerns | Very low | ["Within-study bias","Imprecision"] |
| Antipsychotic continuation:Combining antipsychotics | 18 | Some concerns | Low risk | No concerns | No concerns | Major concerns | No concerns | Very low | ["Within-study bias","Heterogeneity"] |
| Antipsychotic continuation:Dose-escalation | 6 | Major concerns | Low risk | No concerns | Major concerns | No concerns | No concerns | Very low | ["Within-study bias","Imprecision"] |
| Antipsychotic continuation:ECT augmentation | 4 | Major concerns | Low risk | No concerns | No concerns | Major concerns | Major concerns | Very low | ["Within-study bias","Heterogeneity","Incoherence"] |
| Antipsychotic continuation:Switching to non-clozapine | 1 | Major concerns | Low risk | No concerns | Major concerns | No concerns | No concerns | Very low | ["Within-study bias","Imprecision"] |
| Antipsychotic continuation:TMS augmentation | 3 | Some concerns | Low risk | No concerns | Major concerns | No concerns | No concerns | Very low | ["Within-study bias","Imprecision"] |
| Antipsychotic continuation:Xanomeline-trospium augmentation | 1 | No concerns | Low risk | No concerns | Major concerns | No concerns | No concerns | Low | ["Imprecision"] |
| Dose-escalation:Switching to non-clozapine | 1 | Major concerns | Low risk | No concerns | Major concerns | No concerns | No concerns | Very low | ["Within-study bias","Imprecision"] |
| ECT augmentation:Switching to clozapine | 1 | Some concerns | Low risk | No concerns | Major concerns | No concerns | Major concerns | Very low | ["Within-study bias","Imprecision","Incoherence"] |
| Switching to clozapine:Switching to non-clozapine | 17 | Some concerns | Low risk | No concerns | Major concerns | No concerns | No concerns | Very low | ["Within-study bias","Imprecision"] |
| Antipsychotic continuation:Switching to clozapine | 0 | Major concerns | Low risk | No concerns | Major concerns | No concerns | No concerns | Very low | [] |
| CBTp augmentation:Combining antipsychotics | 0 | Some concerns | Low risk | No concerns | Major concerns | No concerns | No concerns | Very low | [] |
| CBTp augmentation:Dose-escalation | 0 | Some concerns | Low risk | No concerns | Major concerns | No concerns | No concerns | Very low | [] |
| CBTp augmentation:ECT augmentation | 0 | Major concerns | Low risk | No concerns | Major concerns | No concerns | No concerns | Very low | [] |
| CBTp augmentation:Switching to clozapine | 0 | Some concerns | Low risk | No concerns | Major concerns | No concerns | No concerns | Very low | [] |
| CBTp augmentation:Switching to non-clozapine | 0 | Major concerns | Low risk | No concerns | Major concerns | No concerns | No concerns | Very low | [] |
| CBTp augmentation:TMS augmentation | 0 | Some concerns | Low risk | No concerns | Major concerns | No concerns | No concerns | Very low | [] |
| CBTp augmentation:Xanomeline-trospium augmentation | 0 | No concerns | Low risk | No concerns | Major concerns | No concerns | No concerns | Very low | [] |
| Combining antipsychotics:Dose-escalation | 0 | Some concerns | Low risk | No concerns | Major concerns | No concerns | No concerns | Very low | [] |
| Combining antipsychotics:ECT augmentation | 0 | Major concerns | Low risk | No concerns | Major concerns | No concerns | No concerns | Very low | [] |
| Combining antipsychotics:Switching to clozapine | 0 | Some concerns | Low risk | No concerns | Major concerns | No concerns | No concerns | Very low | [] |
| Combining antipsychotics:Switching to non-clozapine | 0 | Major concerns | Low risk | No concerns | Major concerns | No concerns | No concerns | Very low | [] |
| Combining antipsychotics:TMS augmentation | 0 | Some concerns | Low risk | No concerns | Major concerns | No concerns | No concerns | Very low | [] |
| Combining antipsychotics:Xanomeline-trospium augmentation | 0 | No concerns | Low risk | No concerns | Major concerns | No concerns | No concerns | Very low | [] |
| Dose-escalation:ECT augmentation | 0 | Major concerns | Low risk | No concerns | Major concerns | No concerns | No concerns | Very low | [] |
| Dose-escalation:Switching to clozapine | 0 | Major concerns | Low risk | No concerns | Major concerns | No concerns | No concerns | Very low | [] |
| Dose-escalation:TMS augmentation | 0 | Some concerns | Low risk | No concerns | Major concerns | No concerns | No concerns | Very low | [] |
| Dose-escalation:Xanomeline-trospium augmentation | 0 | No concerns | Low risk | No concerns | Major concerns | No concerns | No concerns | Very low | [] |
| ECT augmentation:Switching to non-clozapine | 0 | Major concerns | Low risk | No concerns | No concerns | Major concerns | No concerns | Very low | [] |
| ECT augmentation:TMS augmentation | 0 | Some concerns | Low risk | No concerns | Major concerns | No concerns | No concerns | Very low | [] |
| ECT augmentation:Xanomeline-trospium augmentation | 0 | No concerns | Low risk | No concerns | Major concerns | No concerns | No concerns | Very low | [] |
| Switching to clozapine:TMS augmentation | 0 | Some concerns | Low risk | No concerns | Major concerns | No concerns | No concerns | Very low | [] |
| Switching to clozapine:Xanomeline-trospium augmentation | 0 | No concerns | Low risk | No concerns | Major concerns | No concerns | No concerns | Very low | [] |
| Switching to non-clozapine:TMS augmentation | 0 | Some concerns | Low risk | No concerns | Major concerns | No concerns | No concerns | Very low | [] |
| Switching to non-clozapine:Xanomeline-trospium augmentation | 0 | No concerns | Low risk | No concerns | Major concerns | No concerns | No concerns | Very low | [] |
| TMS augmentation:Xanomeline-trospium augmentation | 0 | No concerns | Low risk | No concerns | Major concerns | No concerns | No concerns | Very low | [] |

# 8. SECONDARY OUTCOMES

## (i) clinically relevant response to treatment as defined by the trials (dichotomous)


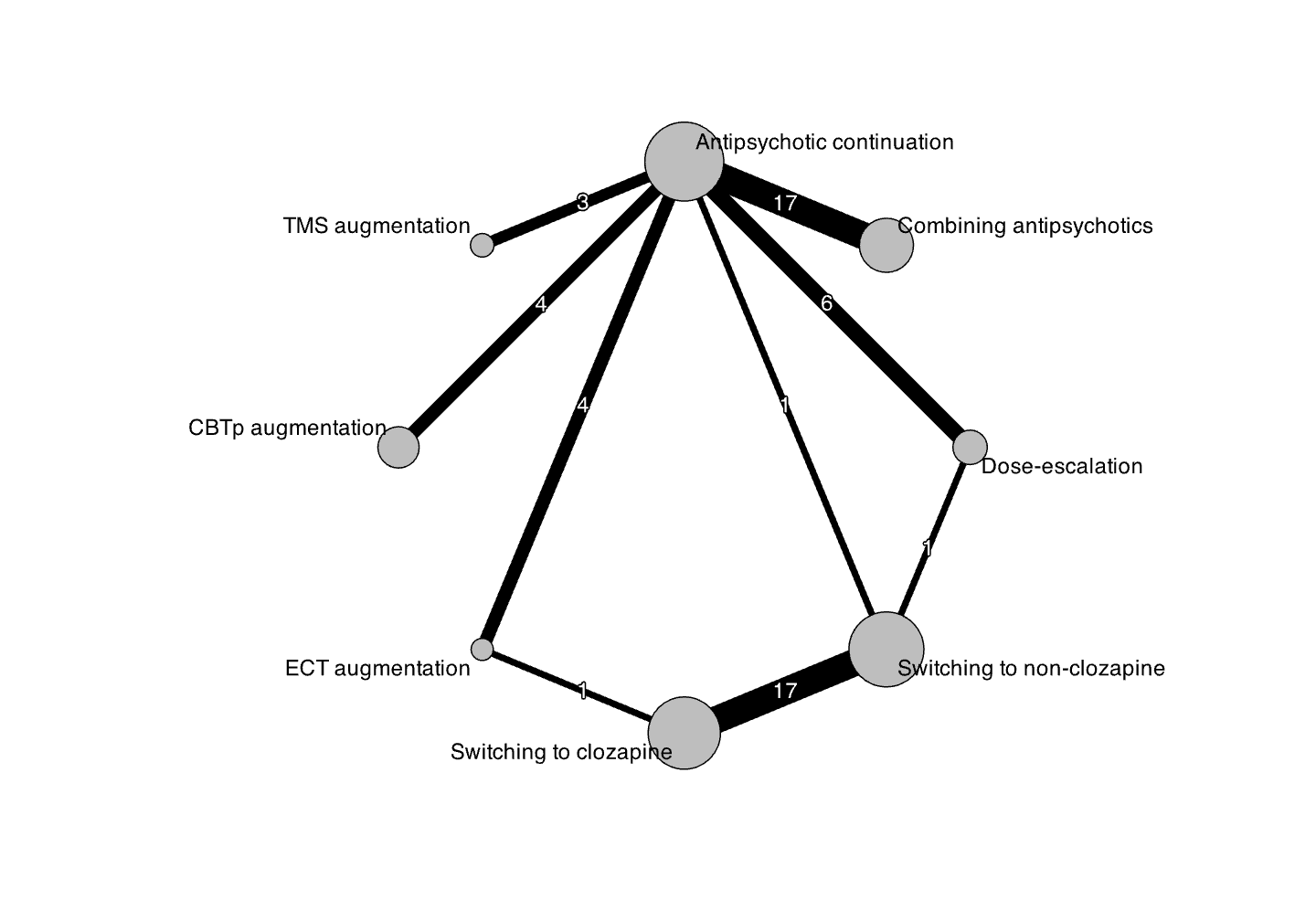


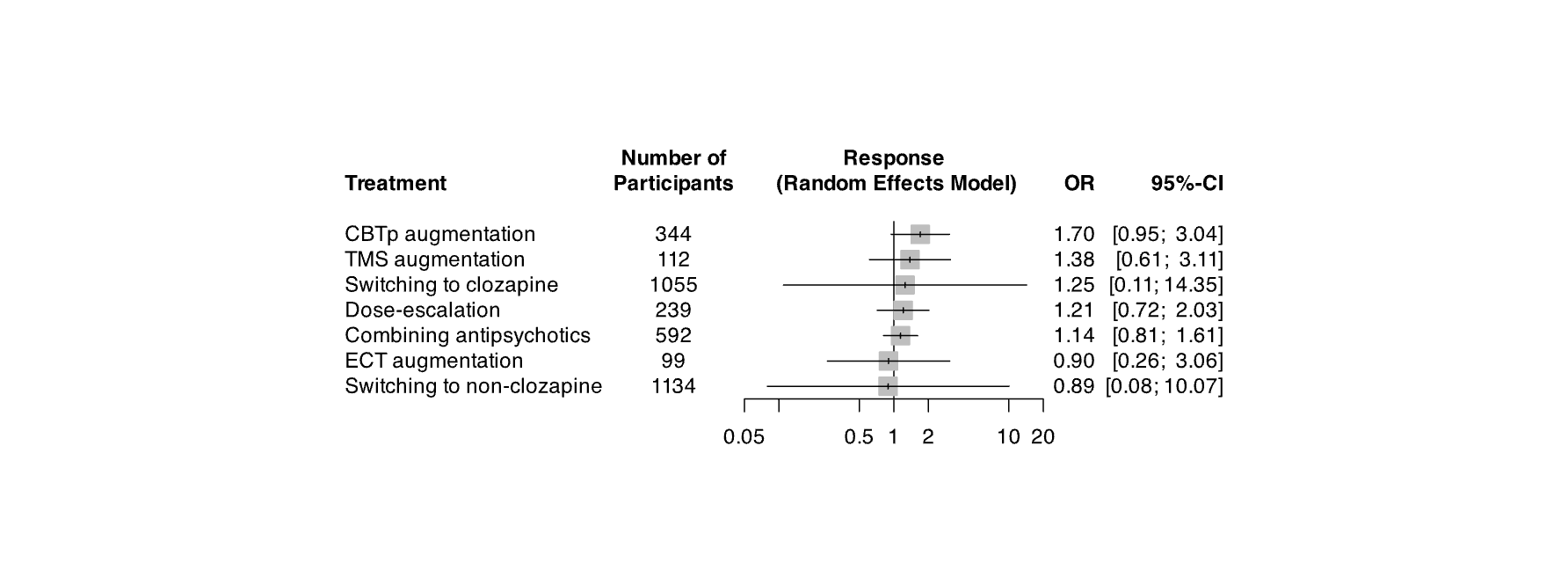


## (ii) dropout due to any reason, as a means of global assessment of acceptability (dichotomous)


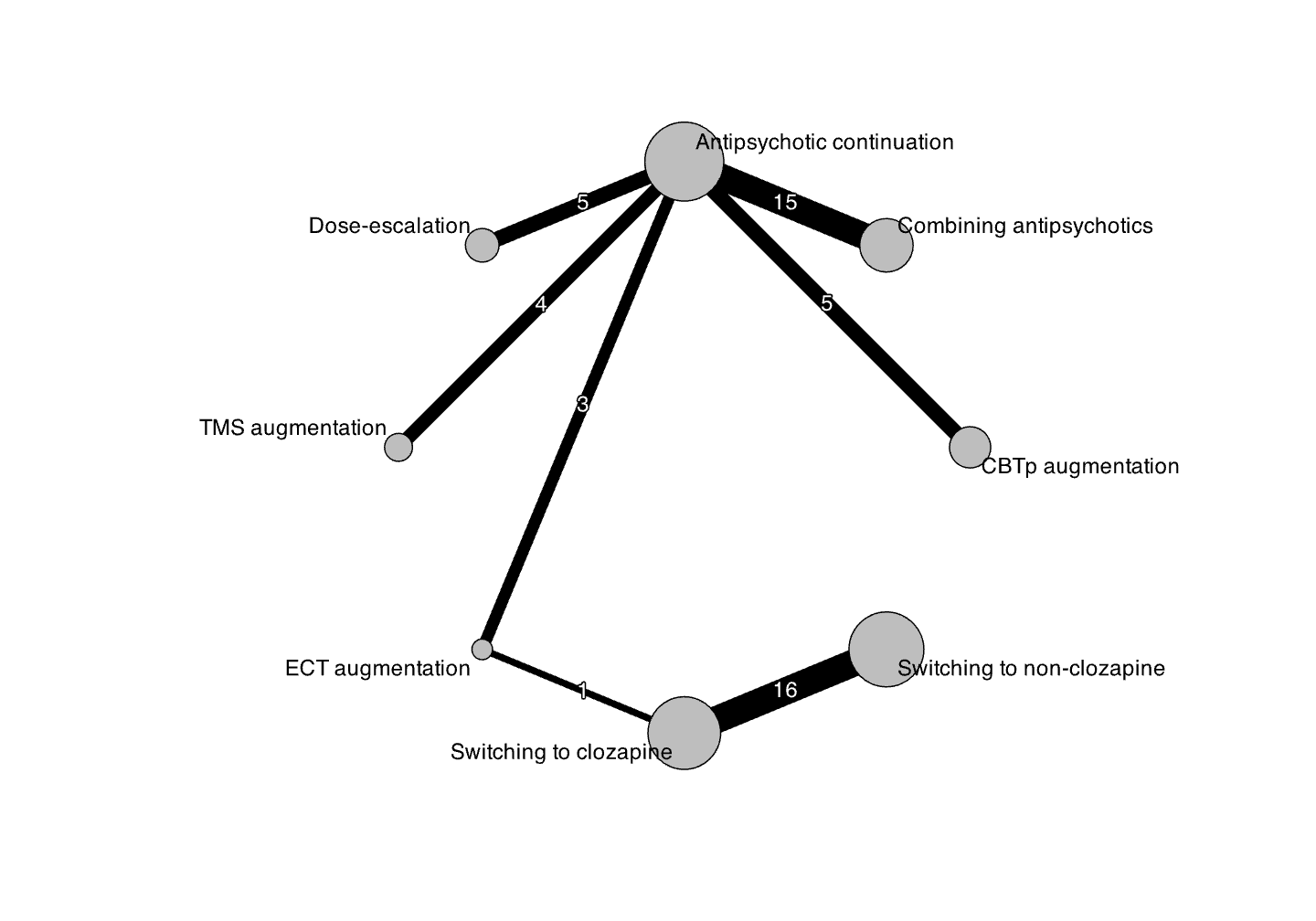


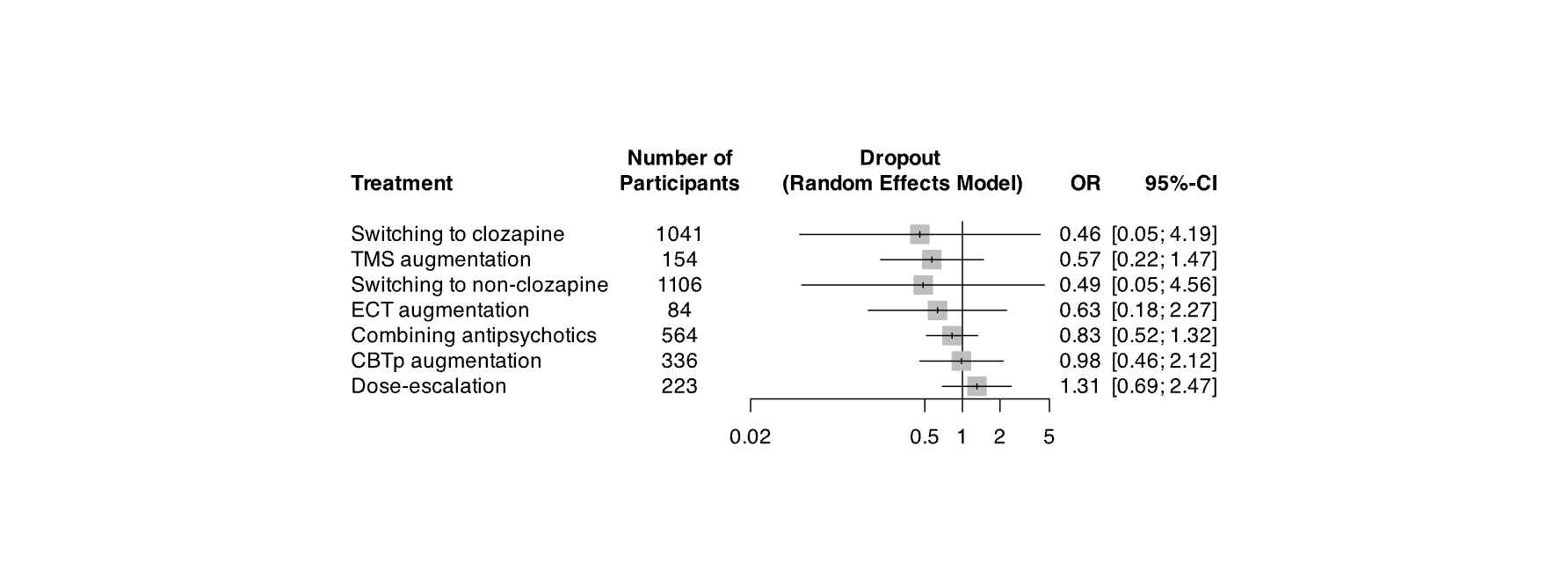


## (iii) total number of patients with adverse effects, as a proxy measurement of tolerability (dichotomous)


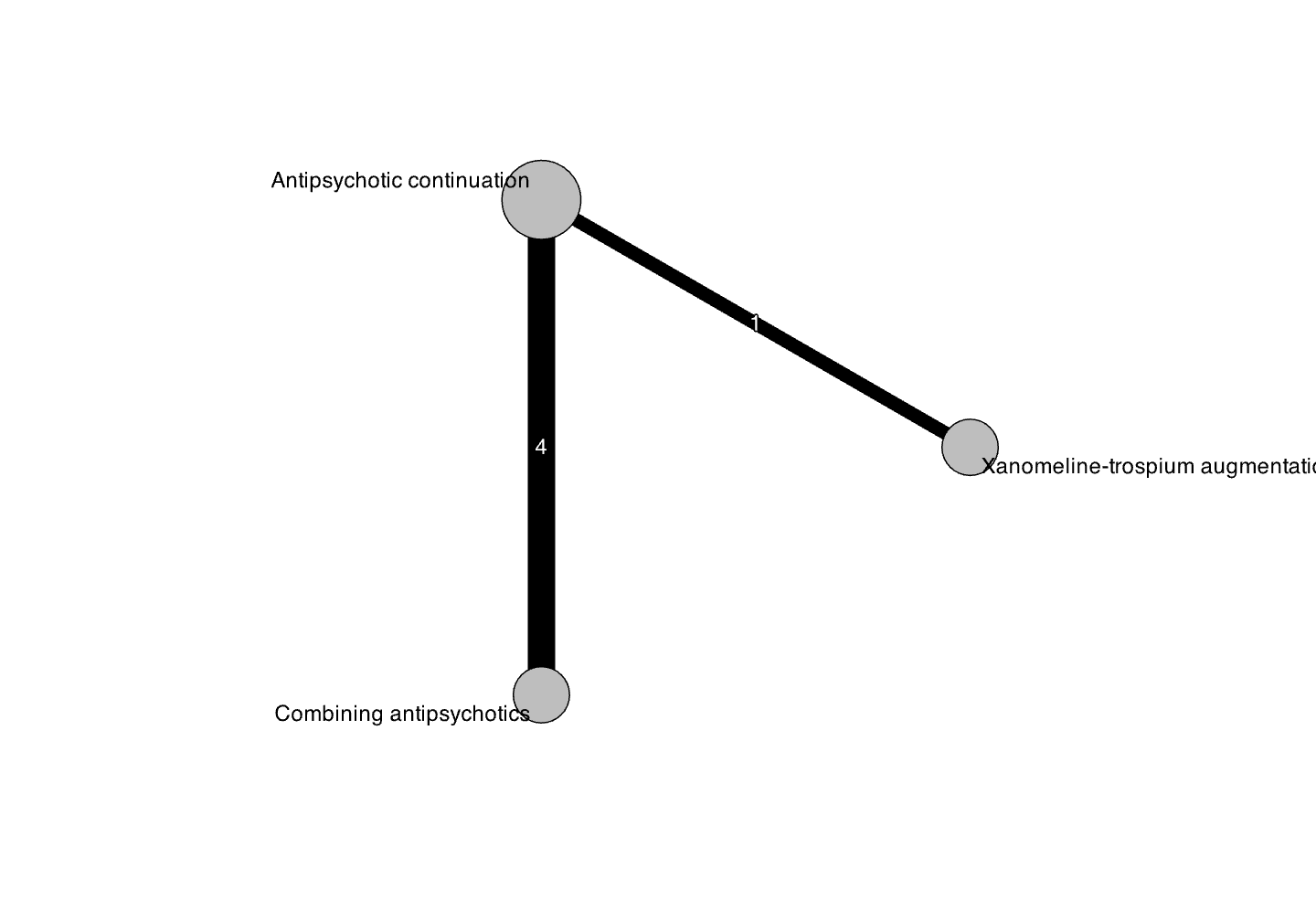

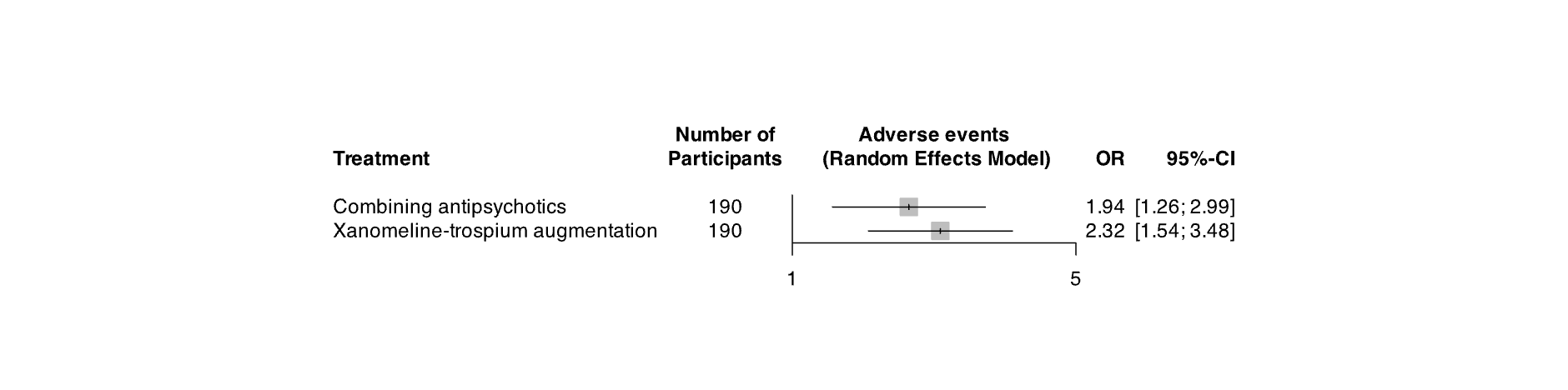


## (iv) positive symptoms (continuous)


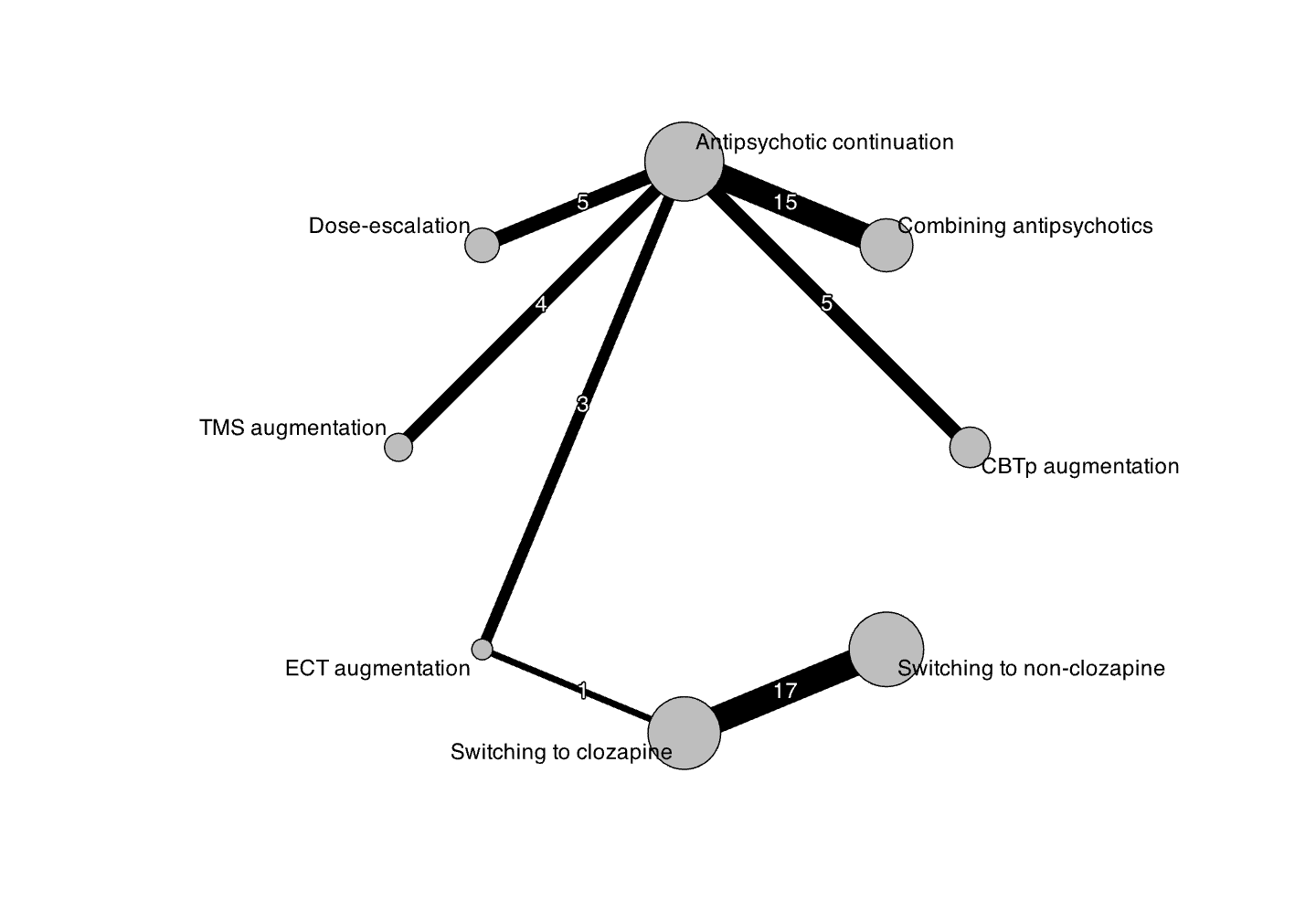

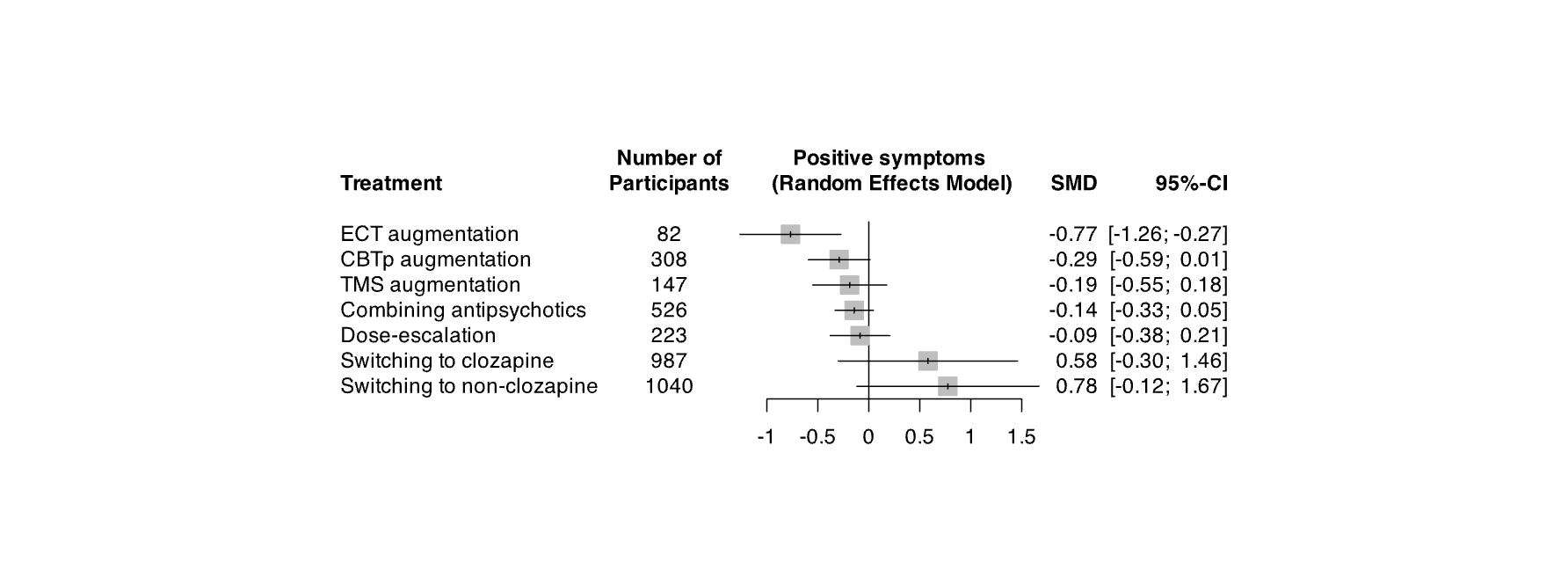


## (v) negative symptoms (continuous)


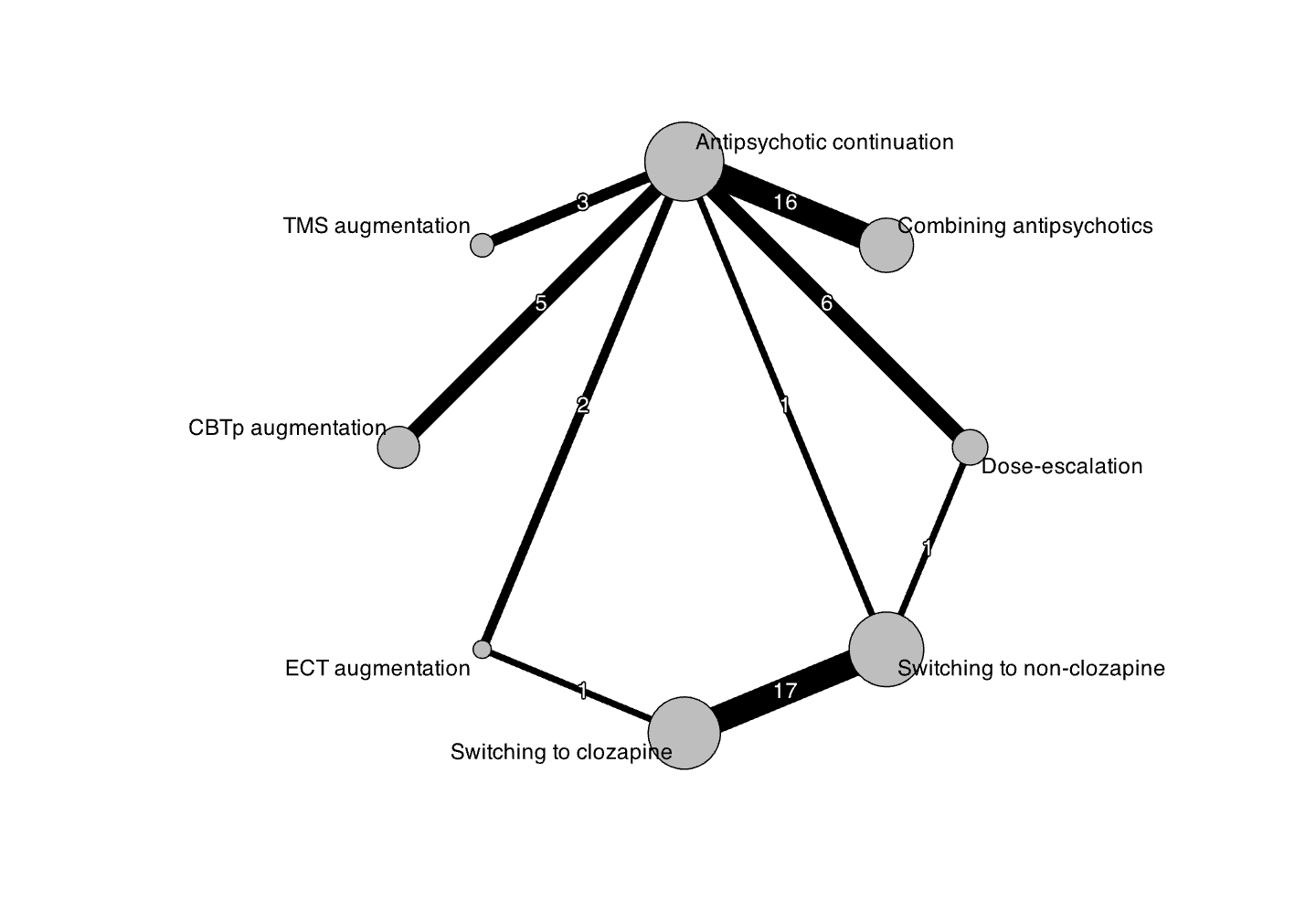


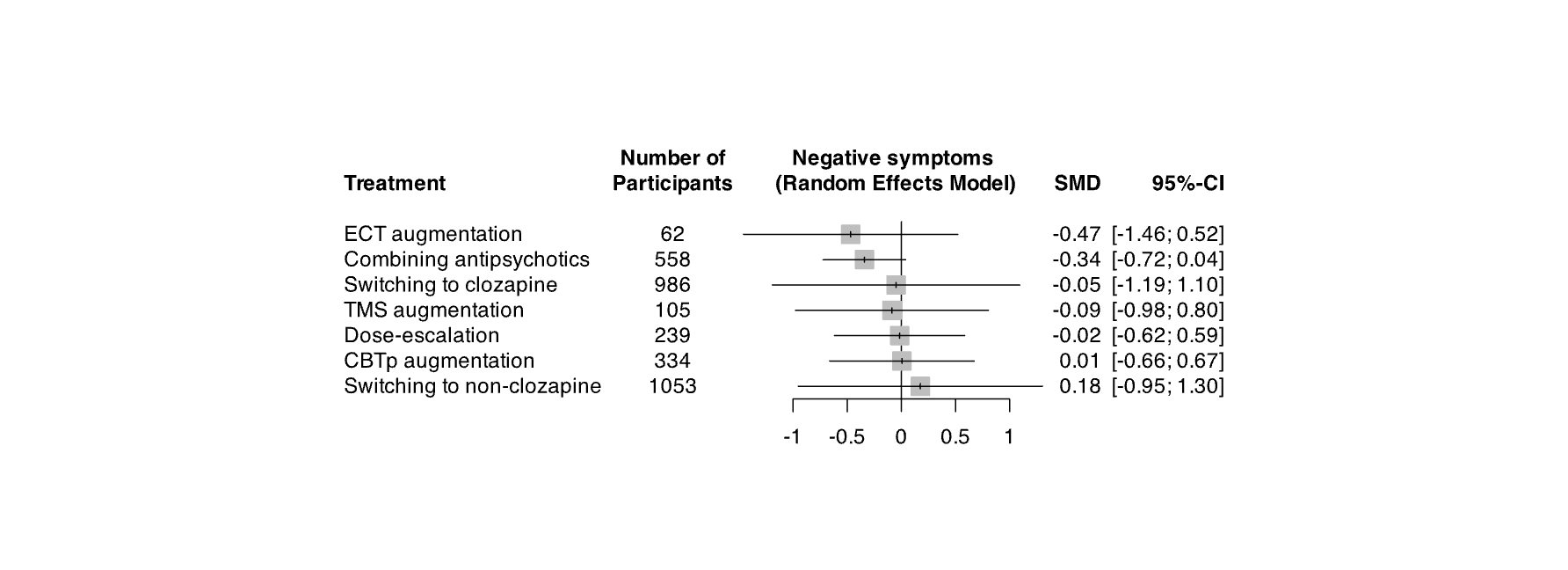


## (vi) depressive symptoms (continuous)

Subnetwork 1 (switching to non-clozapine as a reference)


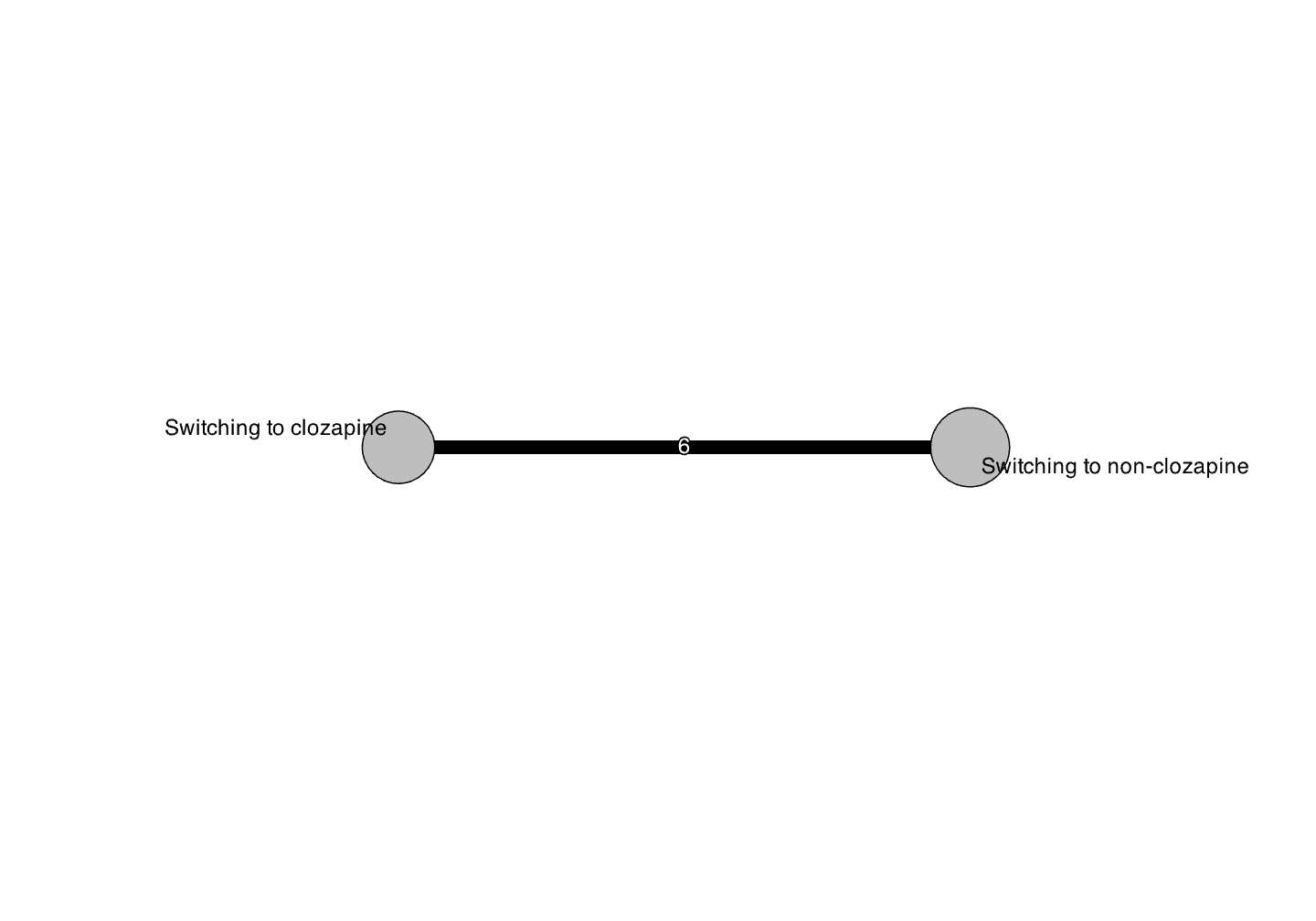

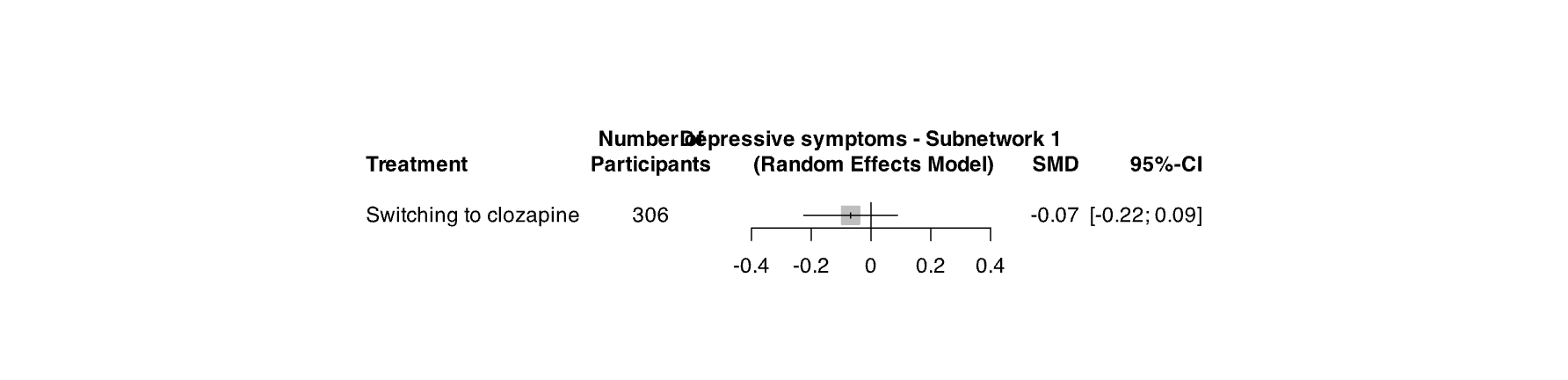


Subnetwork 2 (antipsychotic continuation as a reference)


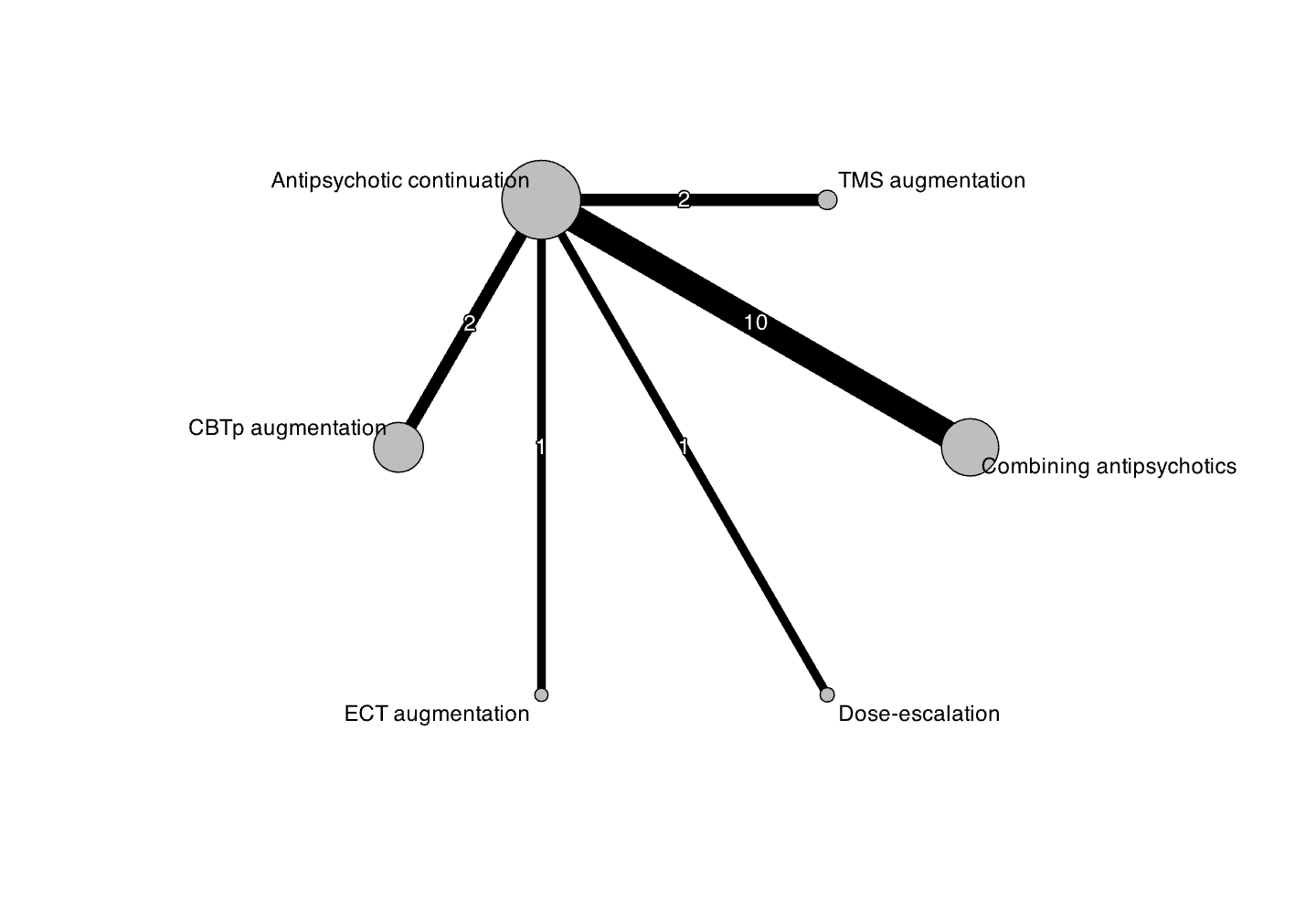


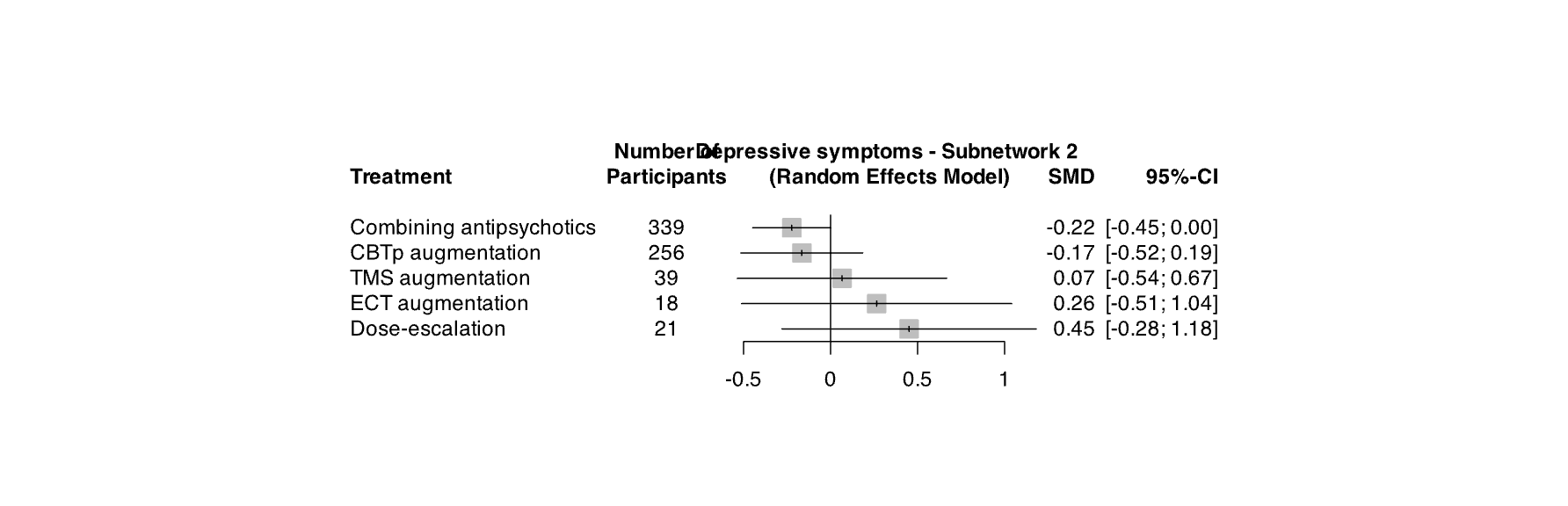


## (vii) quality of life (continuous)

Subnetwork 1 (switching to non-clozapine as a reference)


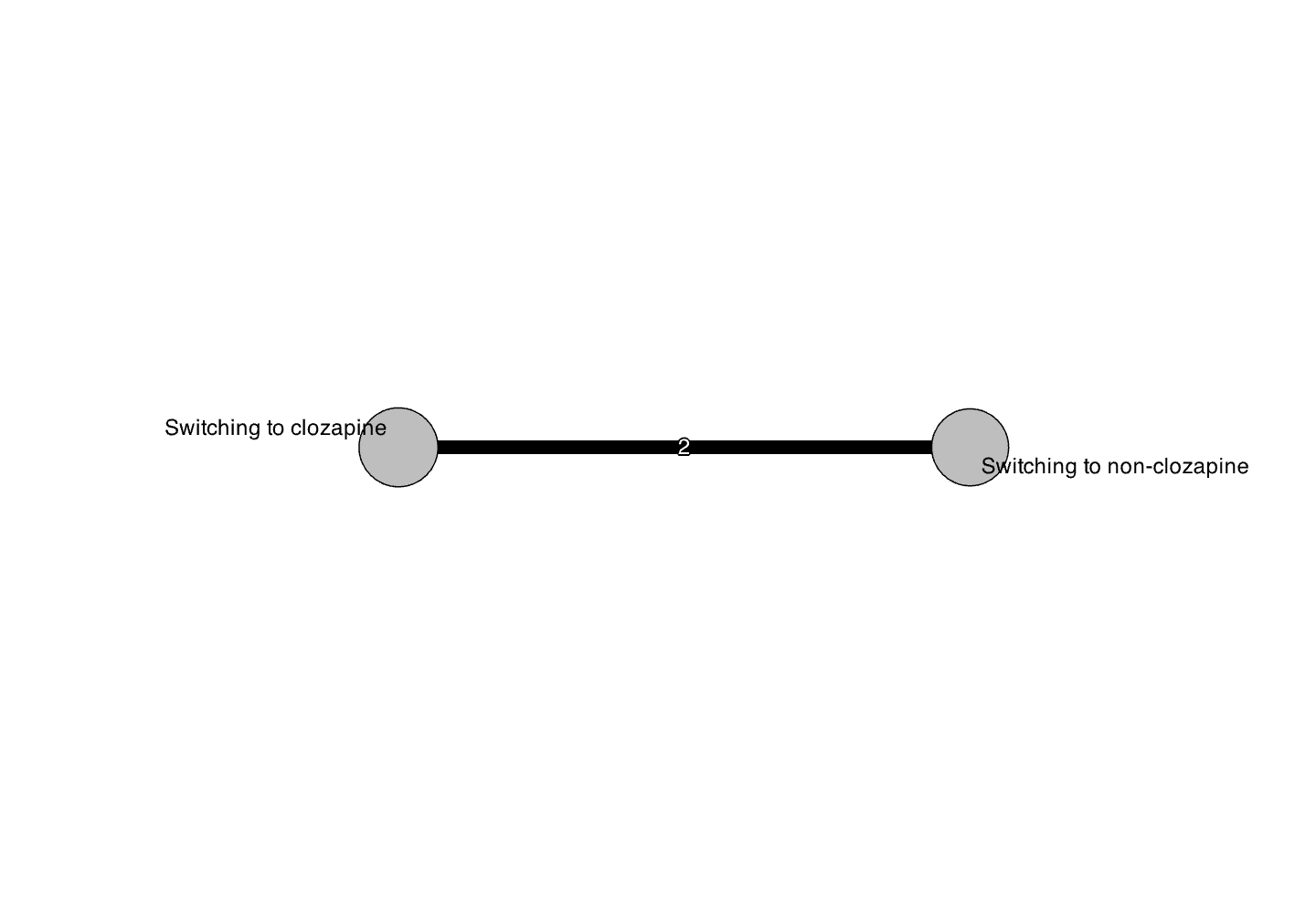


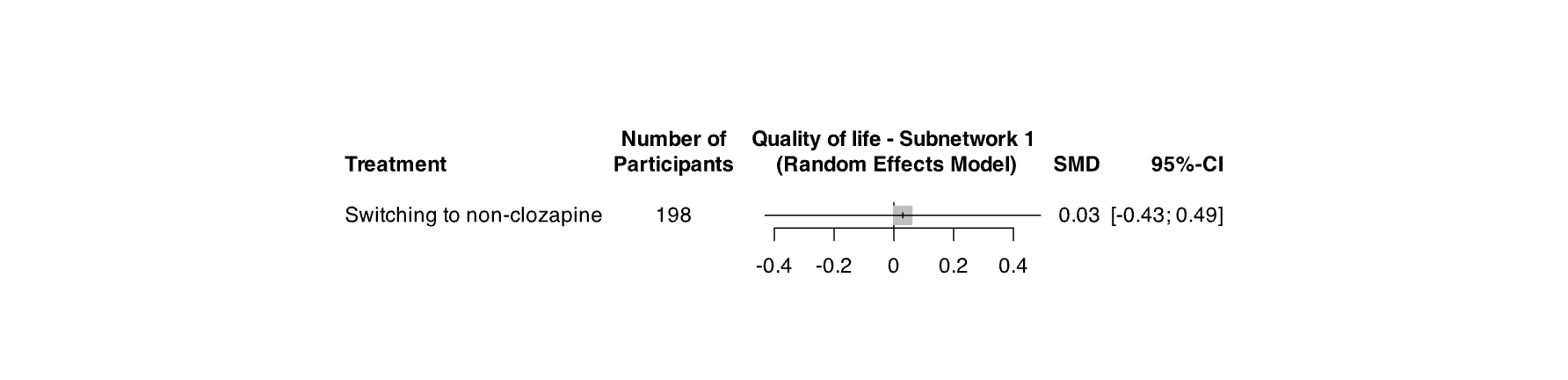


Subnetwork 2 (antipsychotic continuation as a reference)


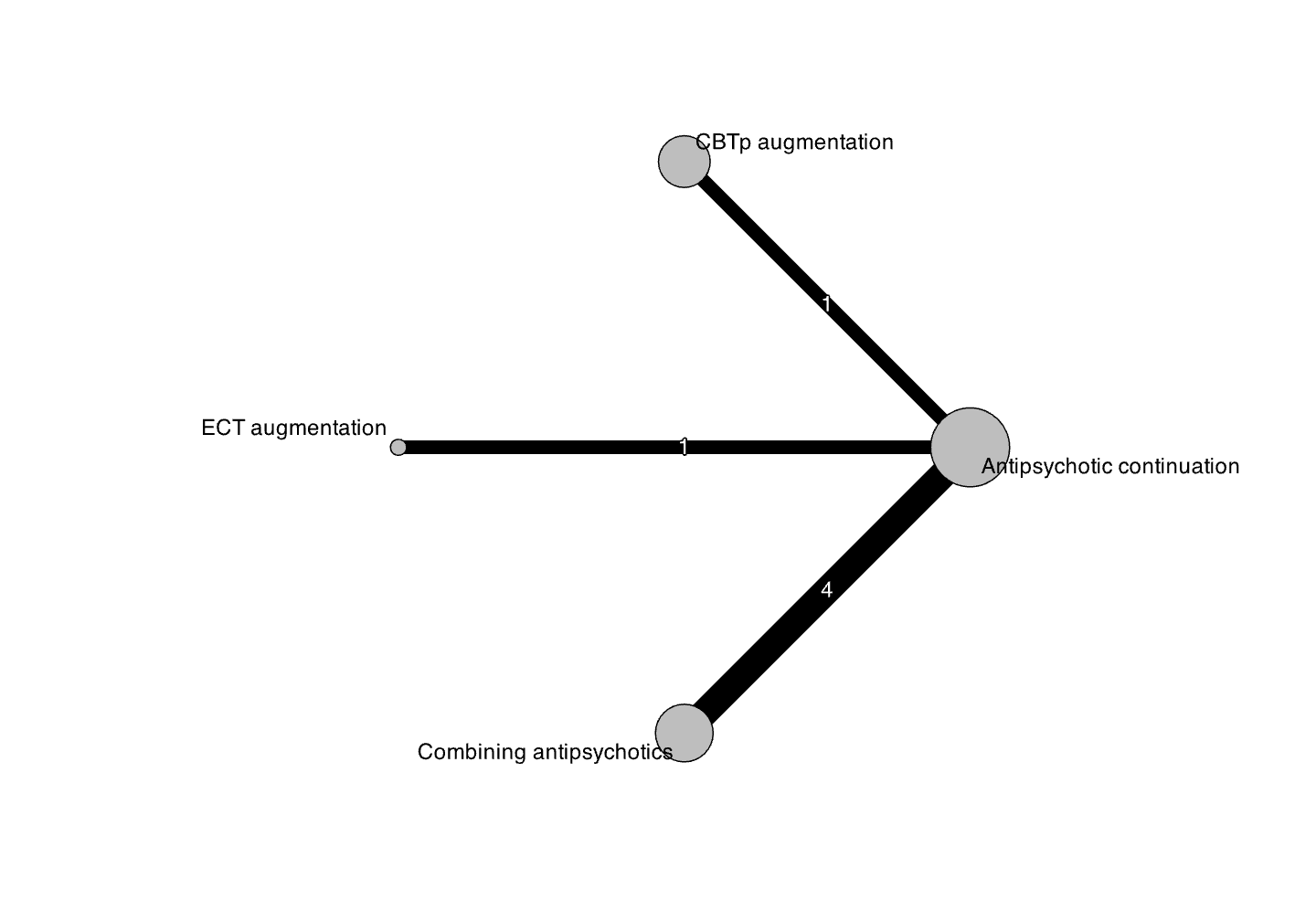


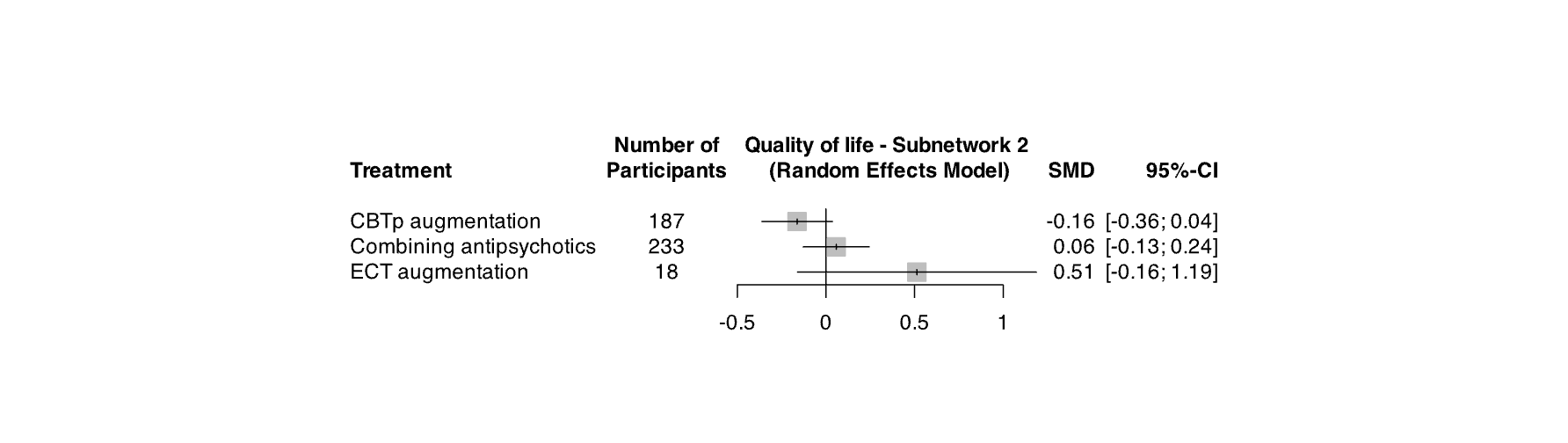


## (viii) social functioning (continuous)

Subnetwork 1 (antipsychotic continuation as a reference)


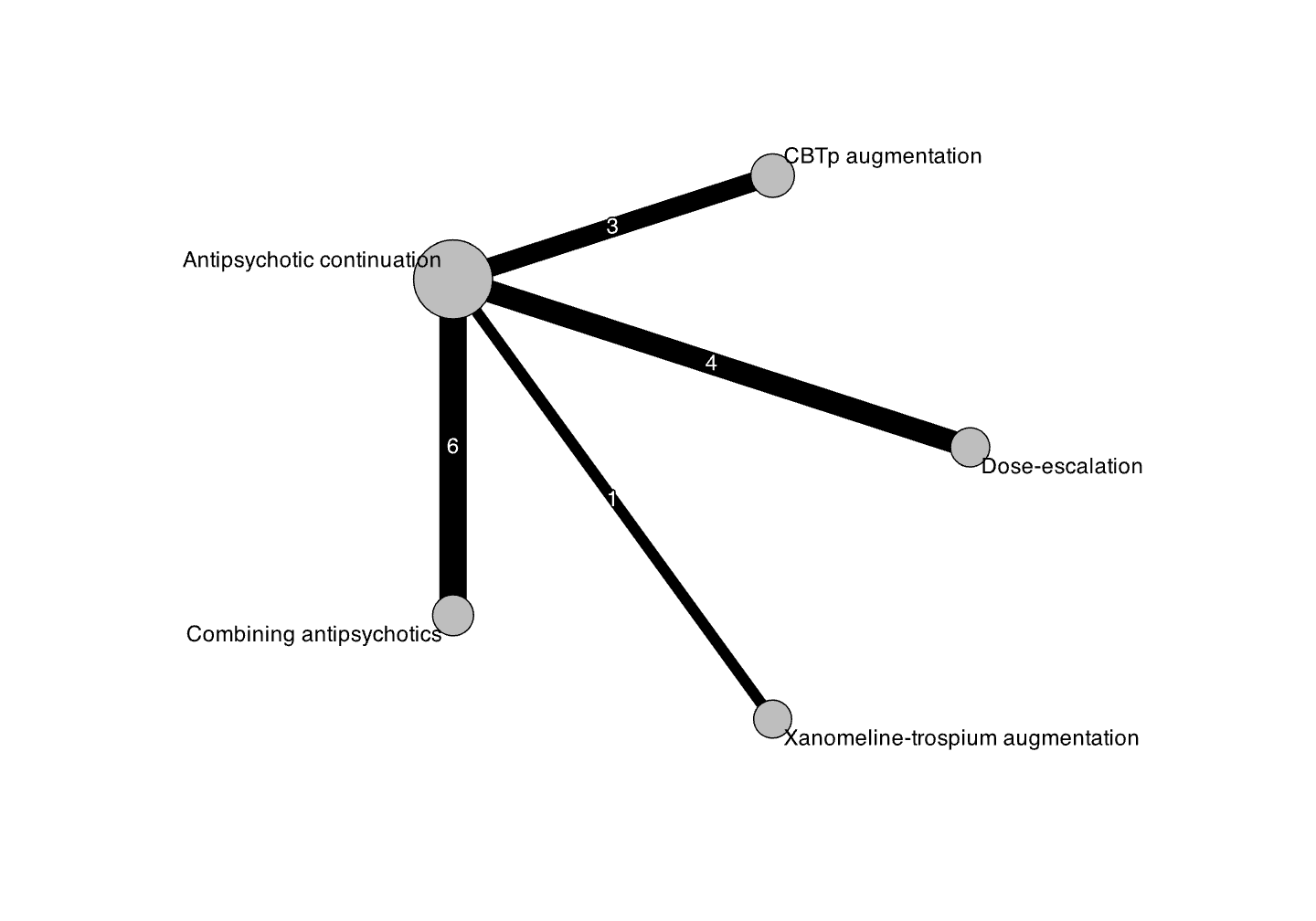


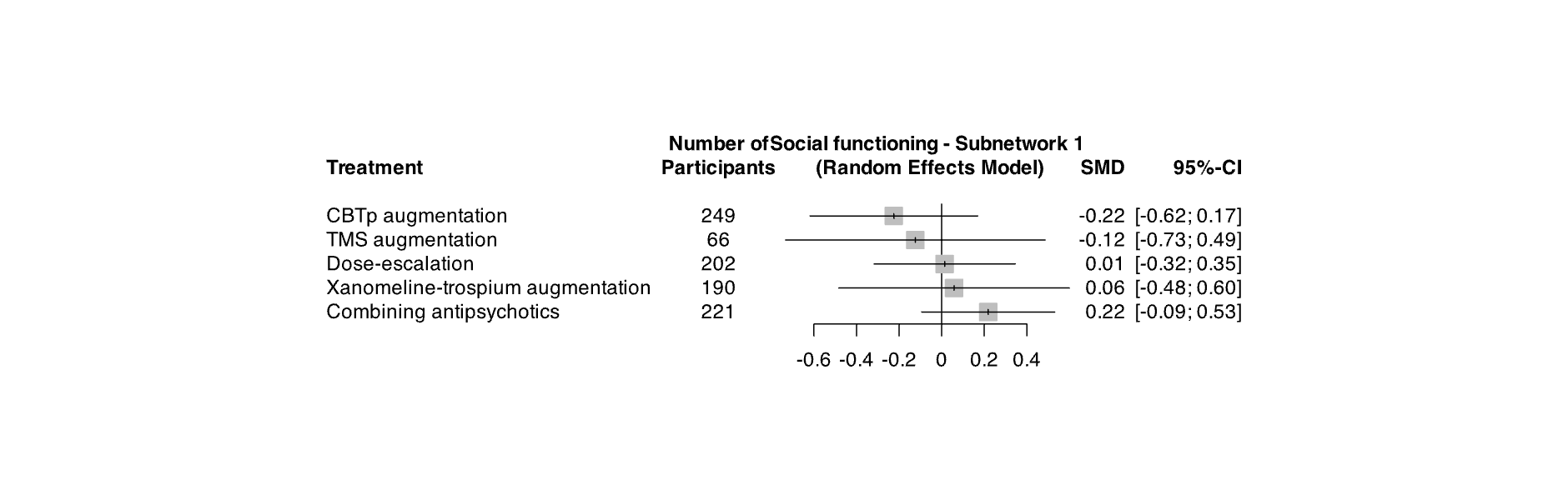


Subnetwork 2 (switching to clozapine as a reference)


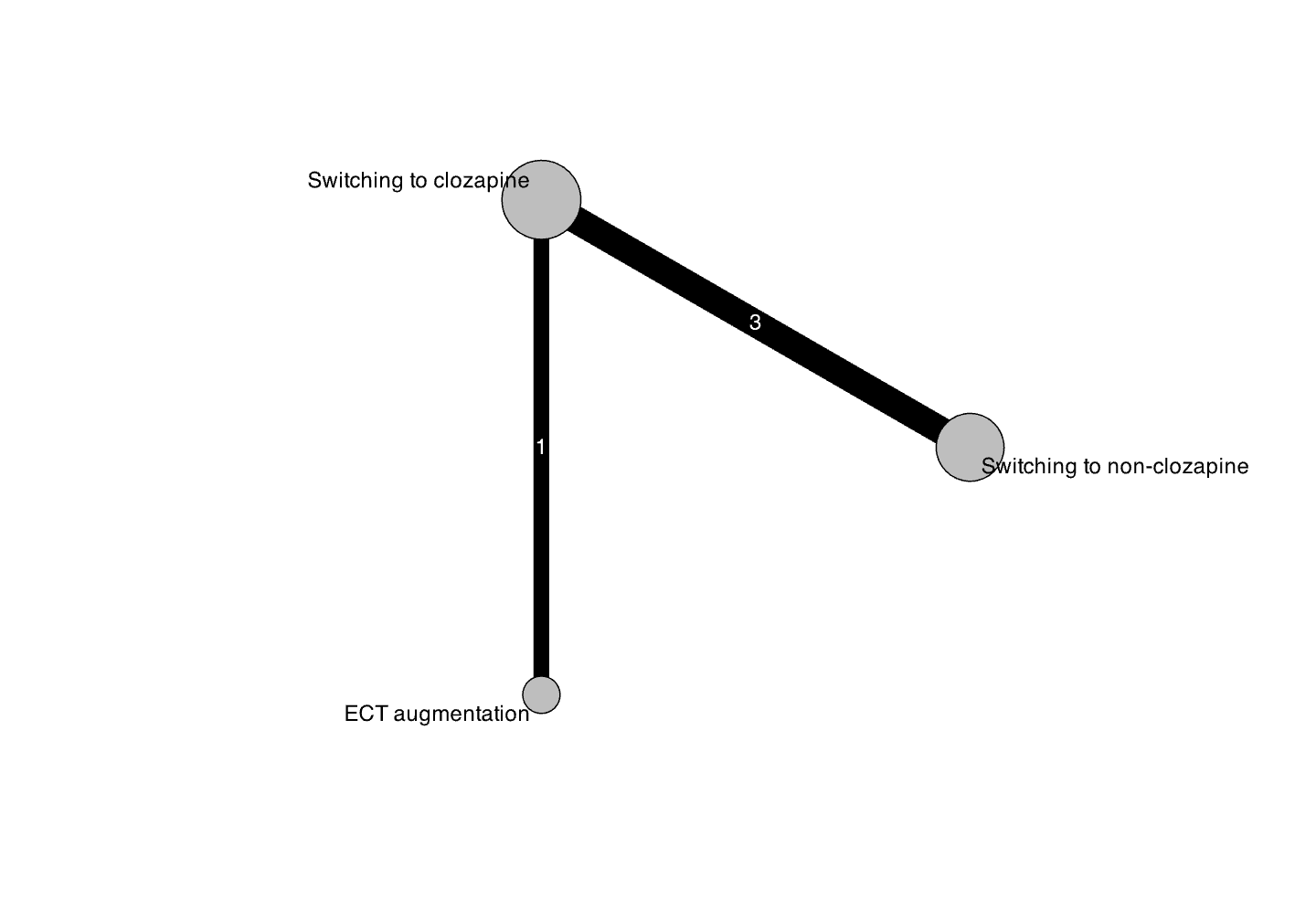


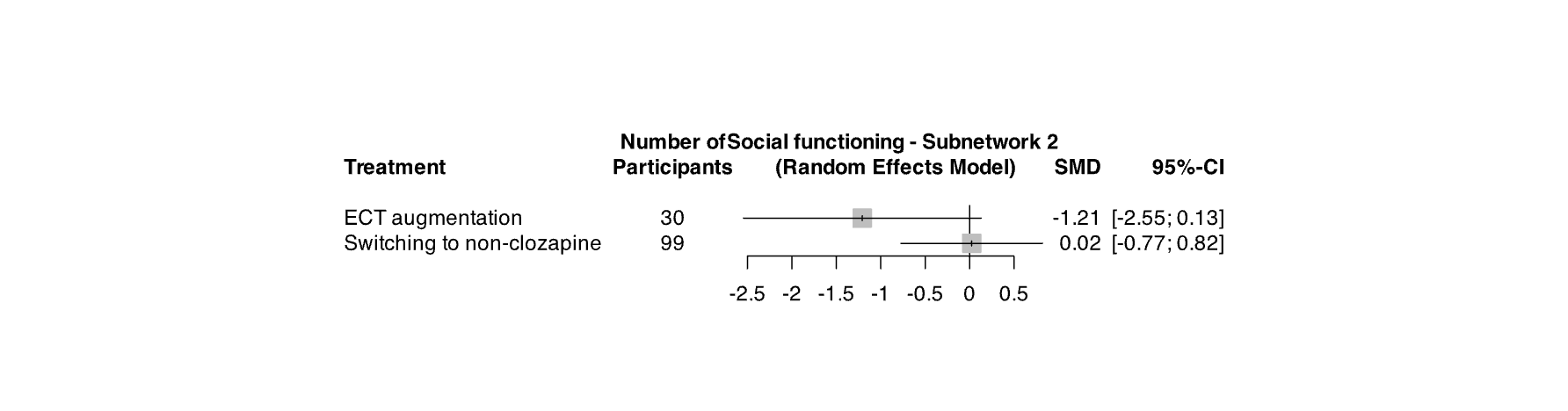


## (ix) use of antiparkinsonian medication (dichotomous)

Subnetwork 1 (switching to clozapine as a reference)


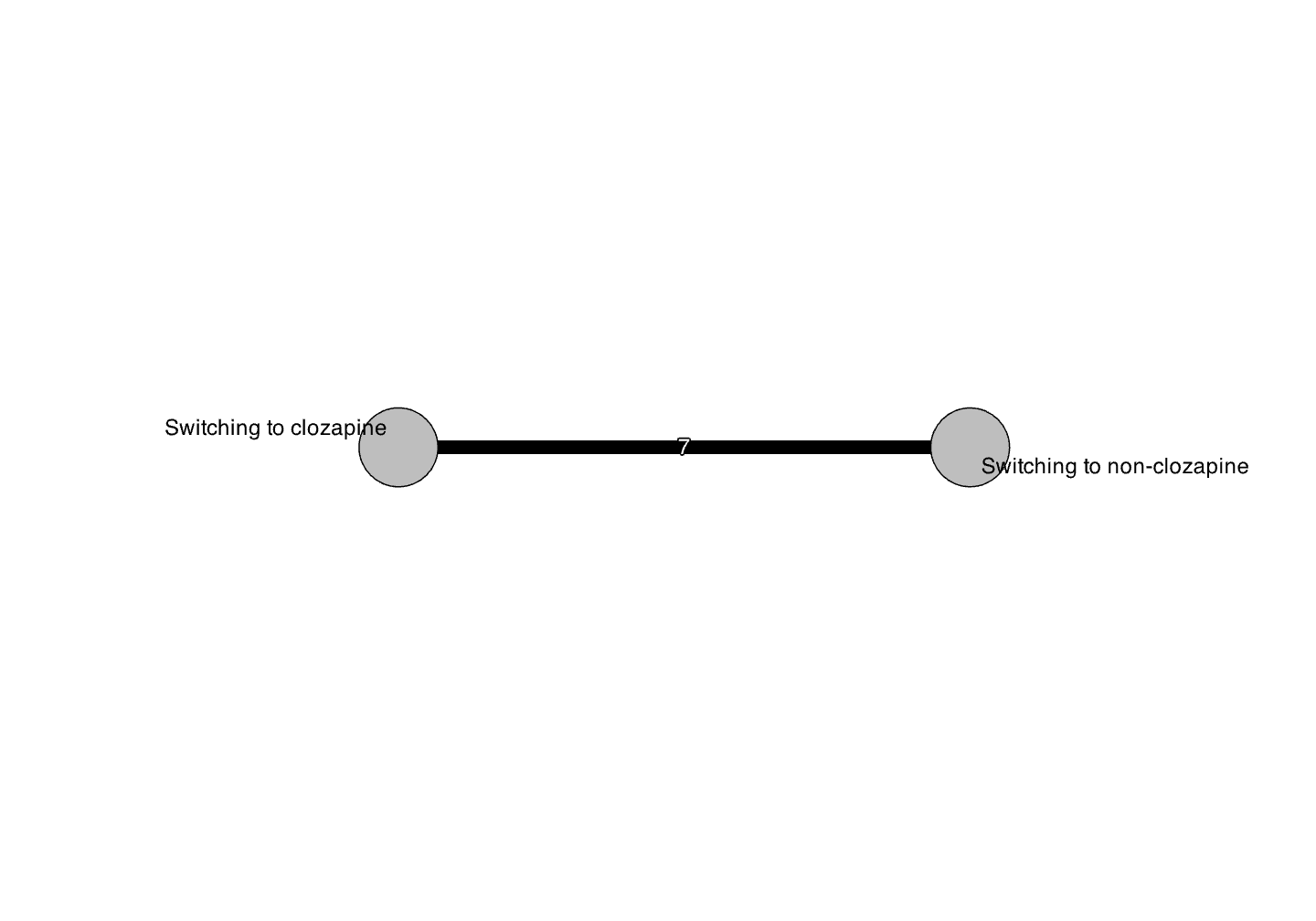


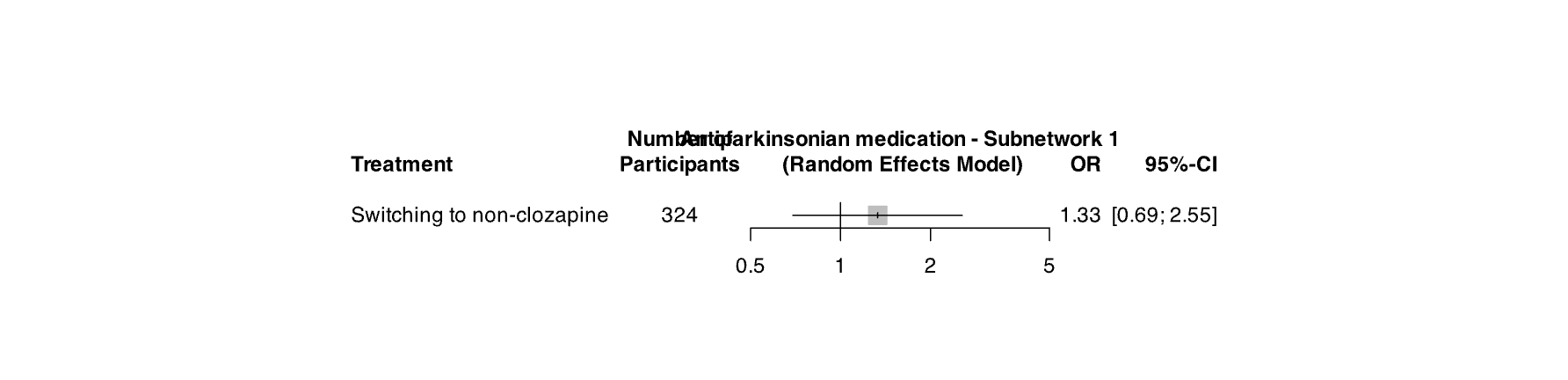


Subnetwork 2 (antipsychotic continuation as a reference)


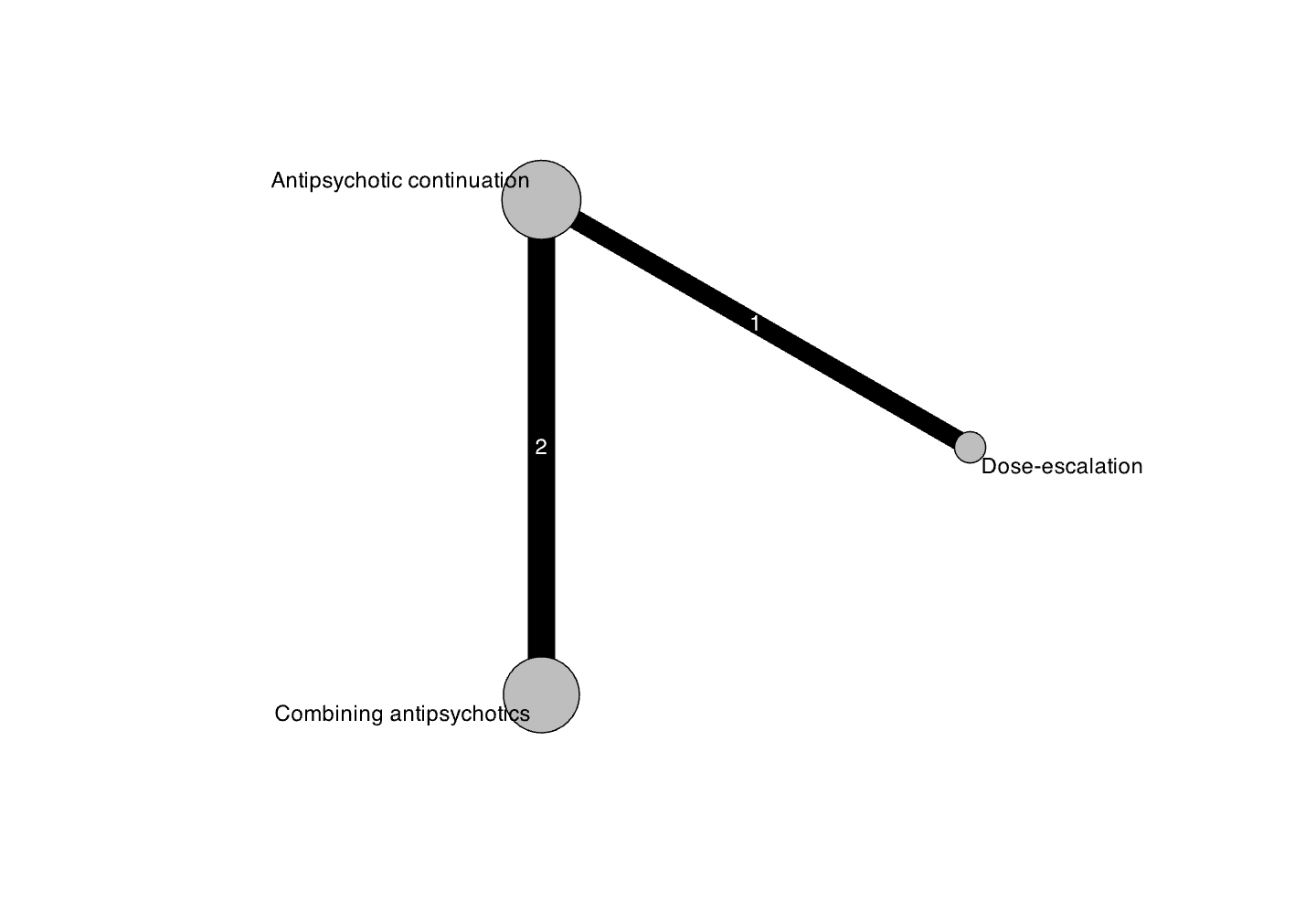

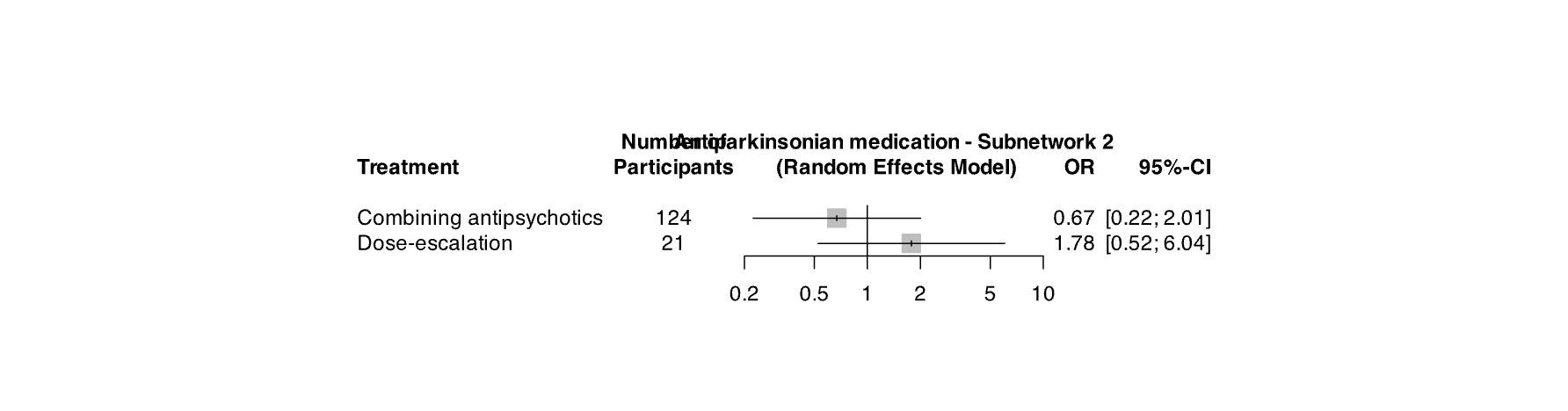


## (x) weight gain (kg, continuous)

Subnetwork 1 (switching to non-clozapine as a reference)


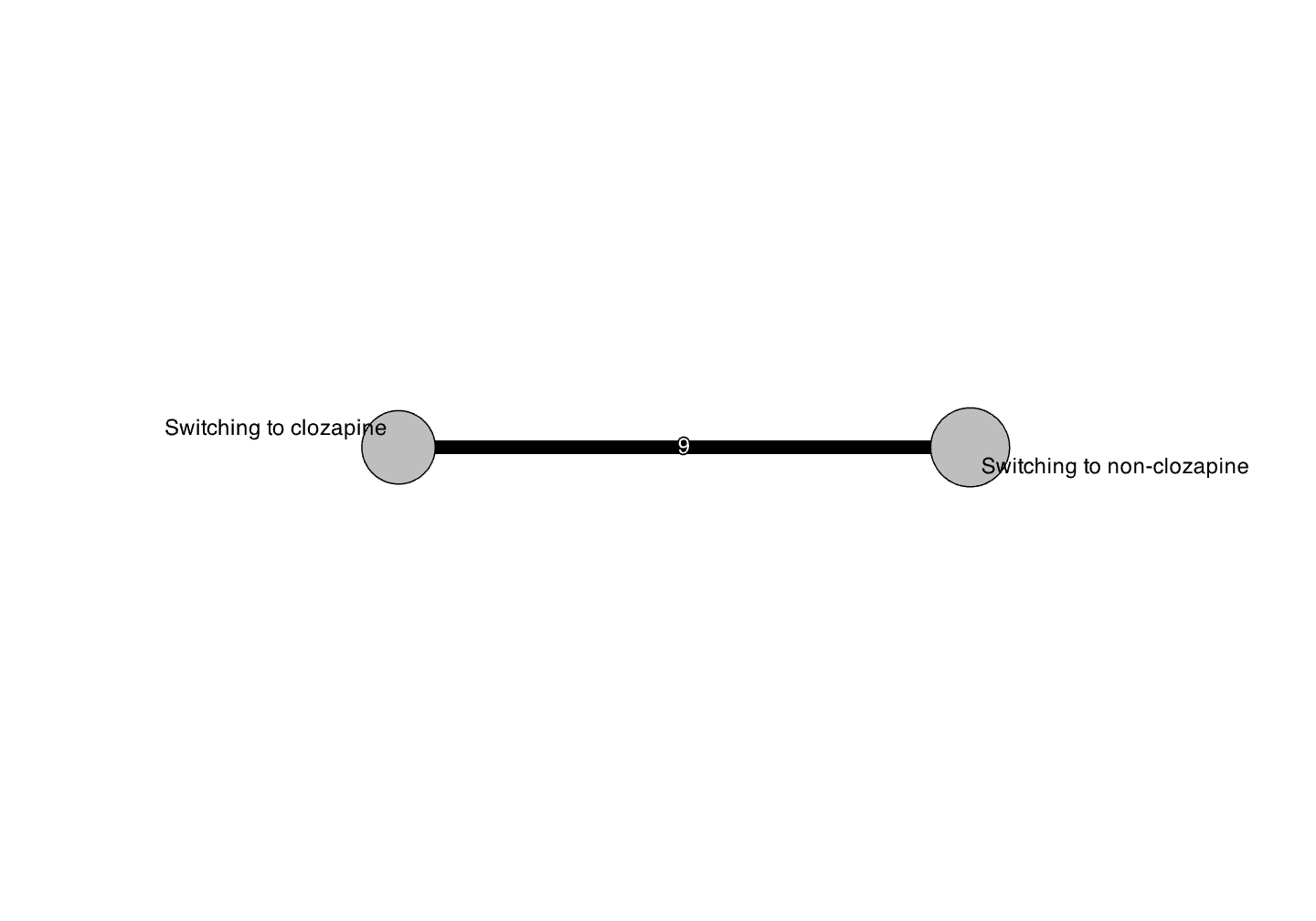

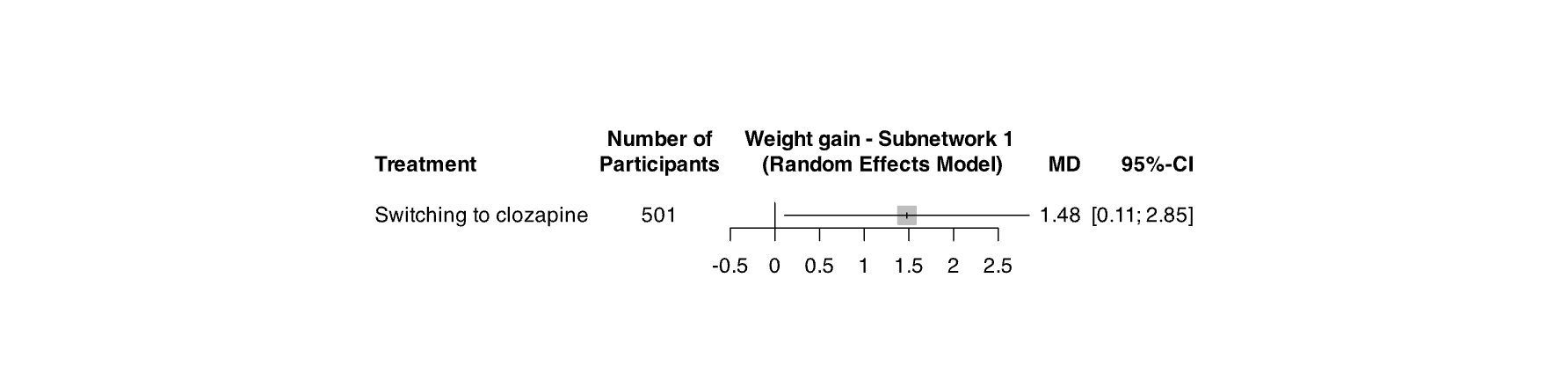


Subnetwork 2 (antipsychotic continuation as a reference)


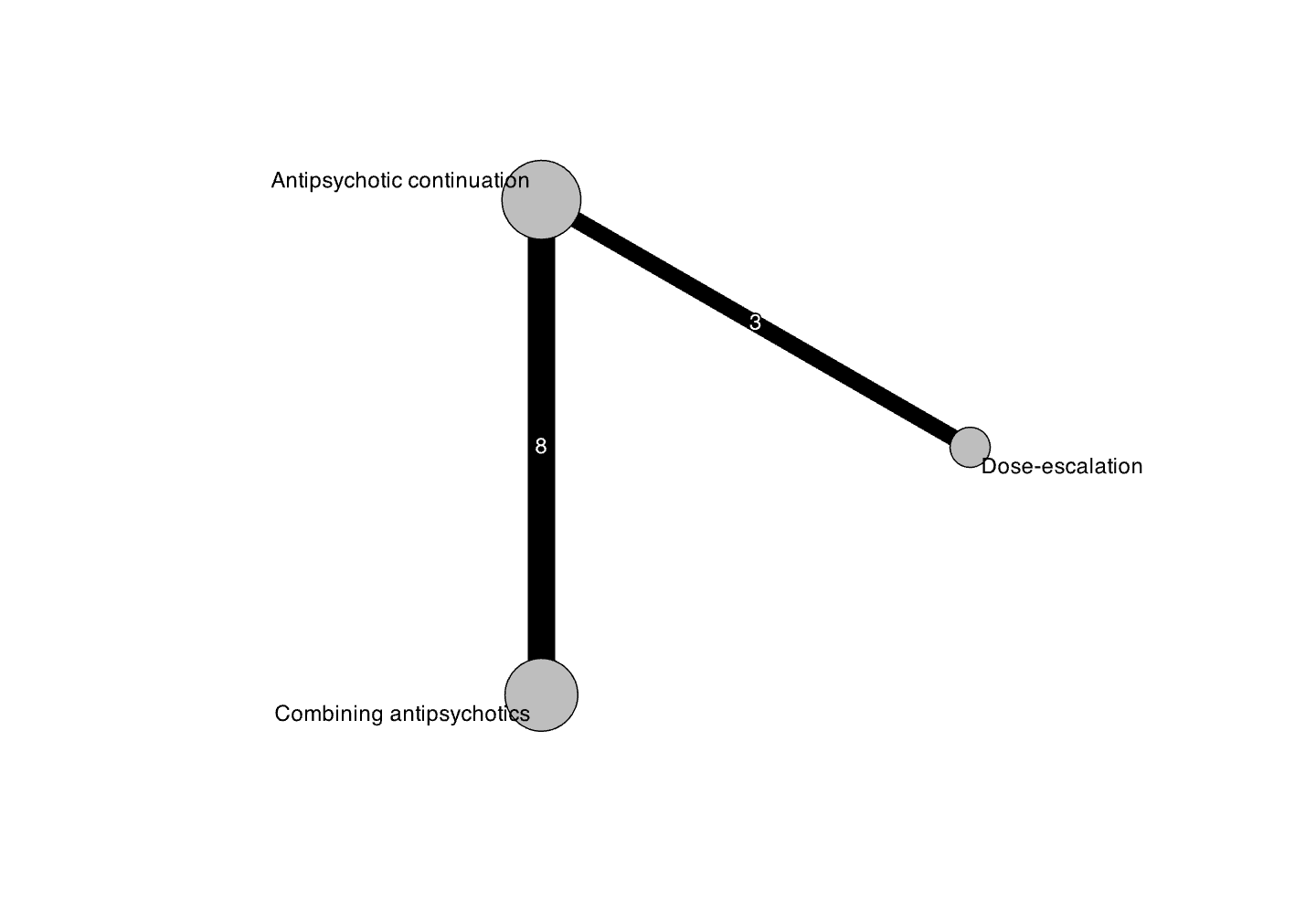

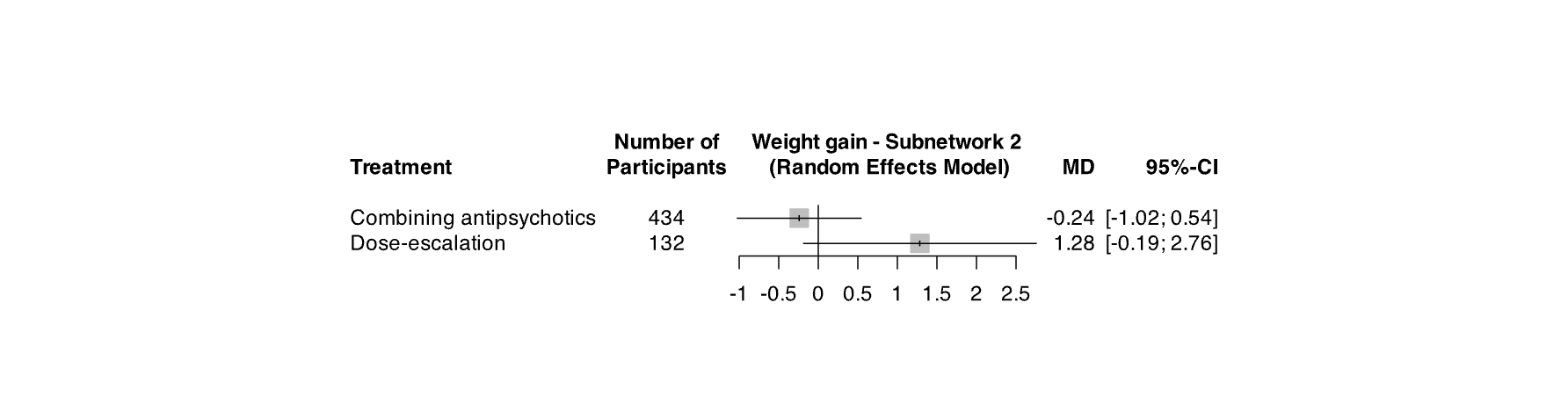


## (xi) sedation (dichotomous)

Subnetwork 1 (switching to non-clozapine as a reference)


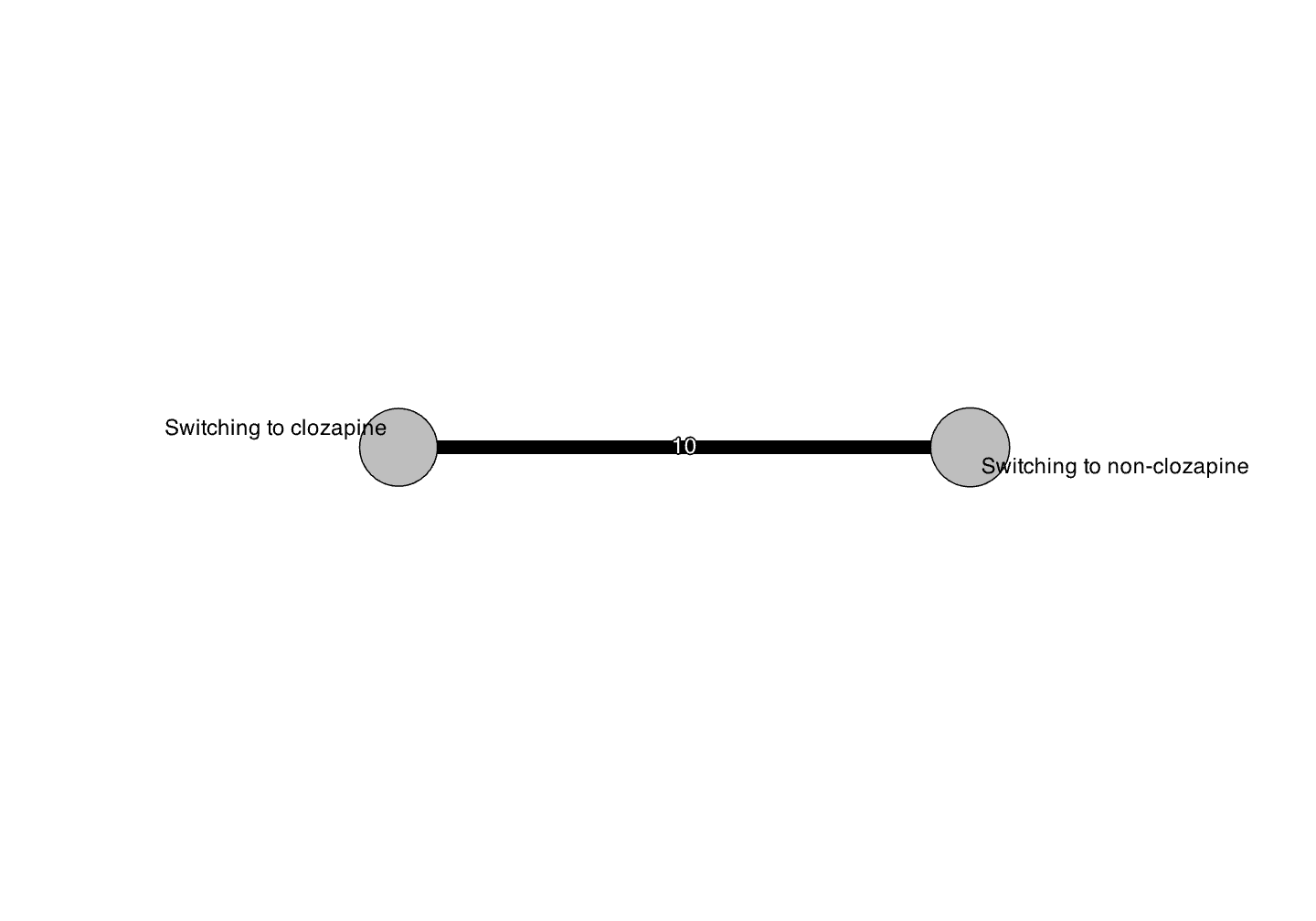

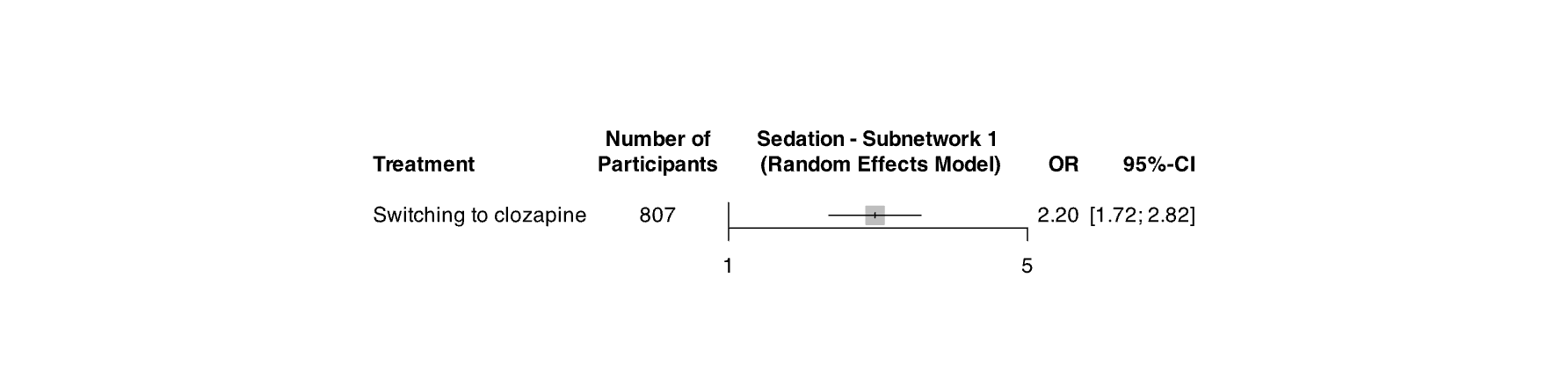


Subnetwork 2 (antipsychotic continuation as a reference)


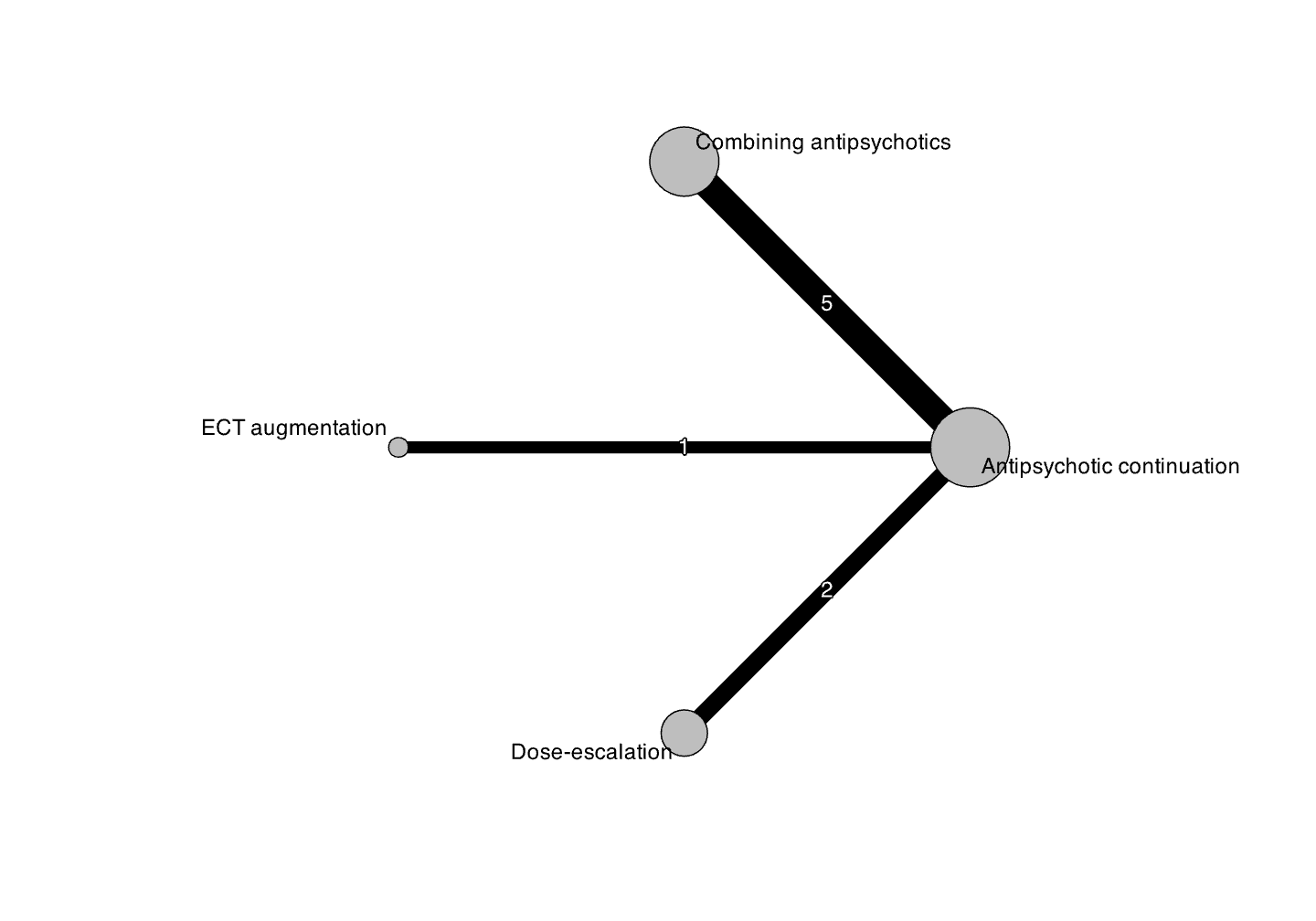


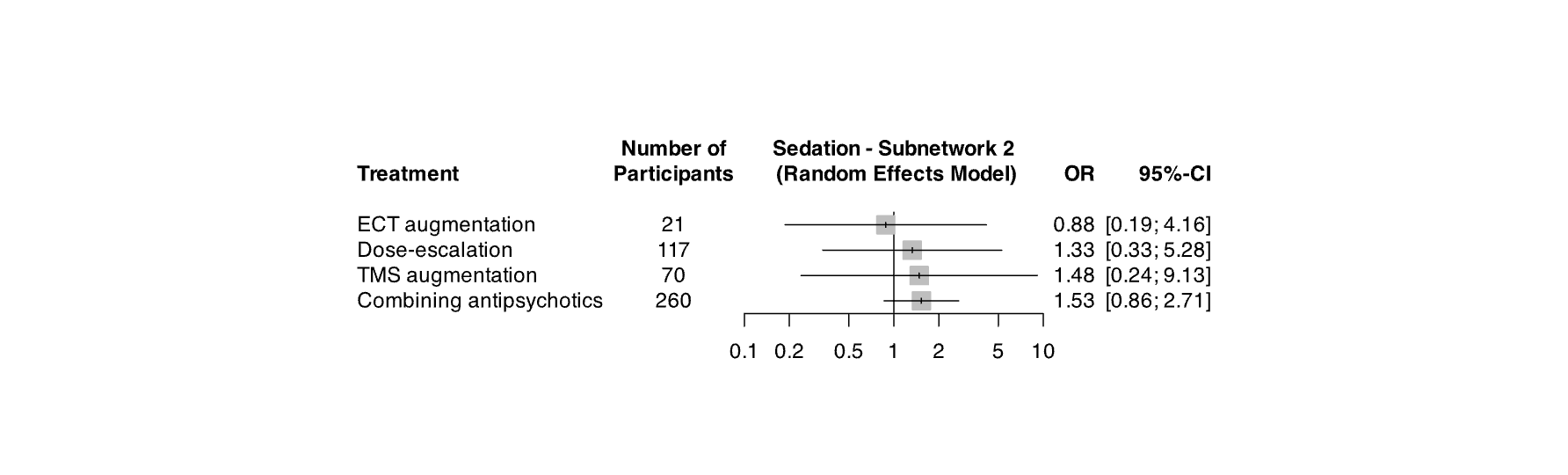


## (xii) prolactin levels (ng/mL, continuous)

Subnetwork 1 (switching to clozapine as a reference)


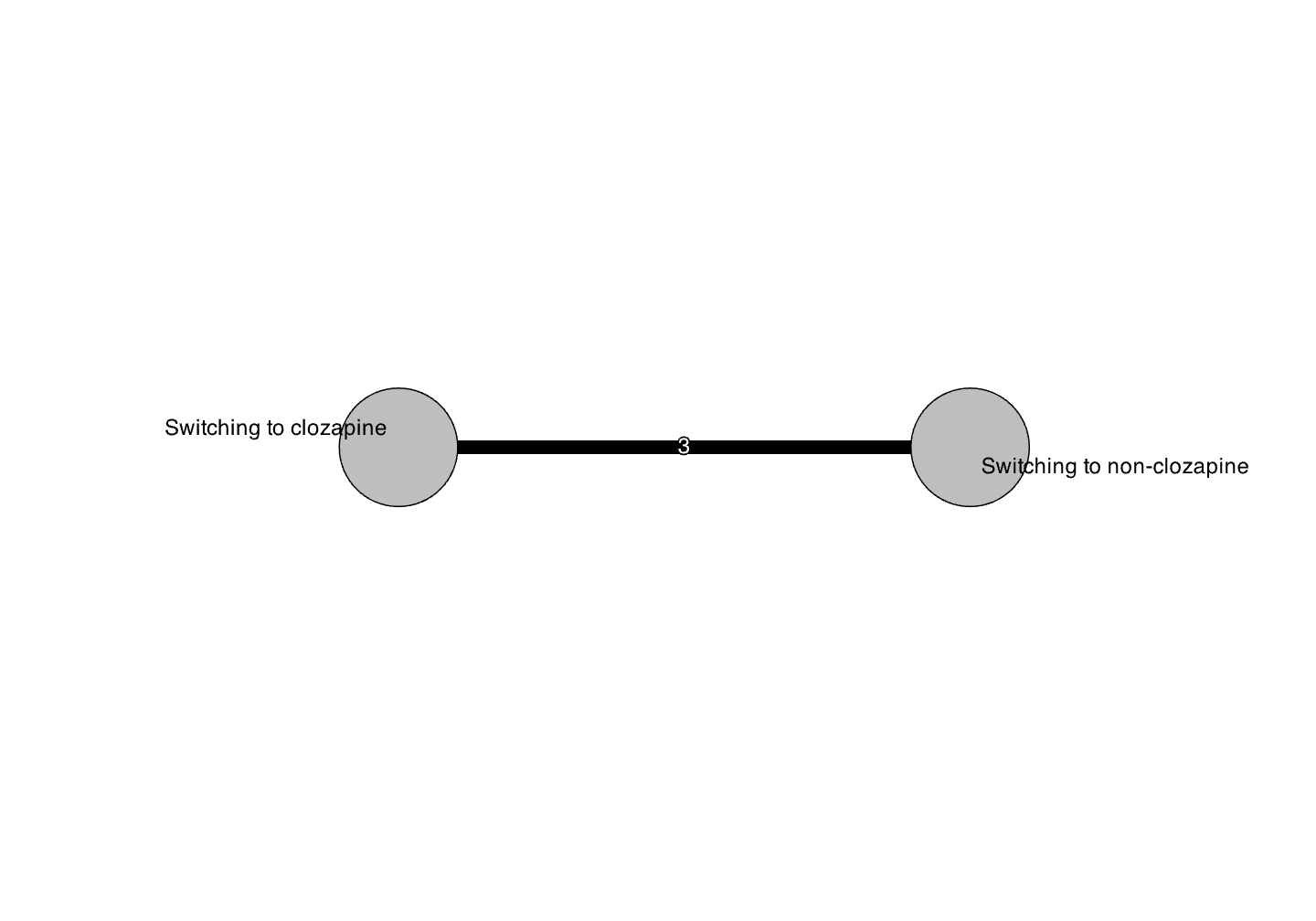

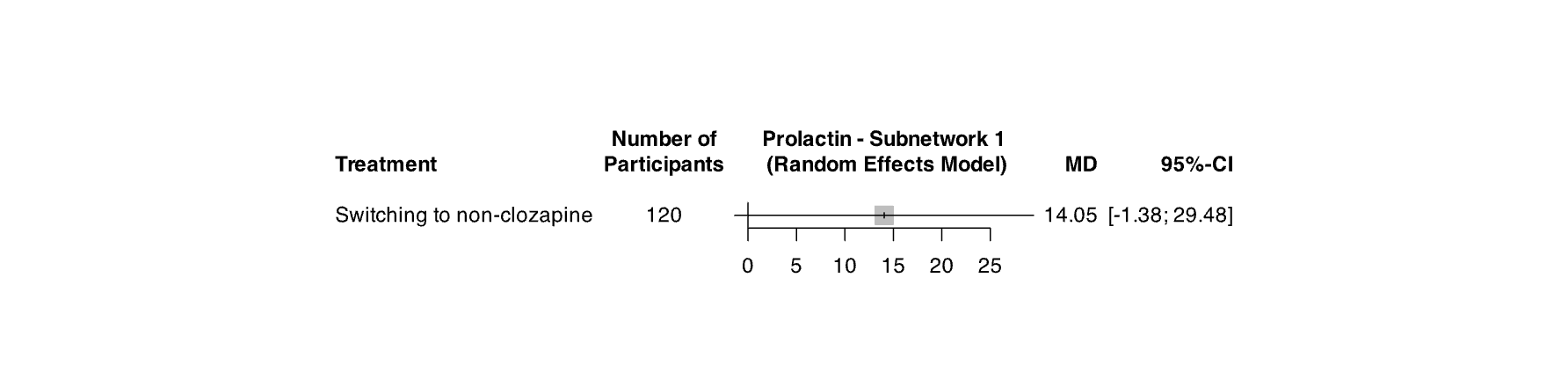


Subnetwork 2 (antipsychotic continuation as a reference)


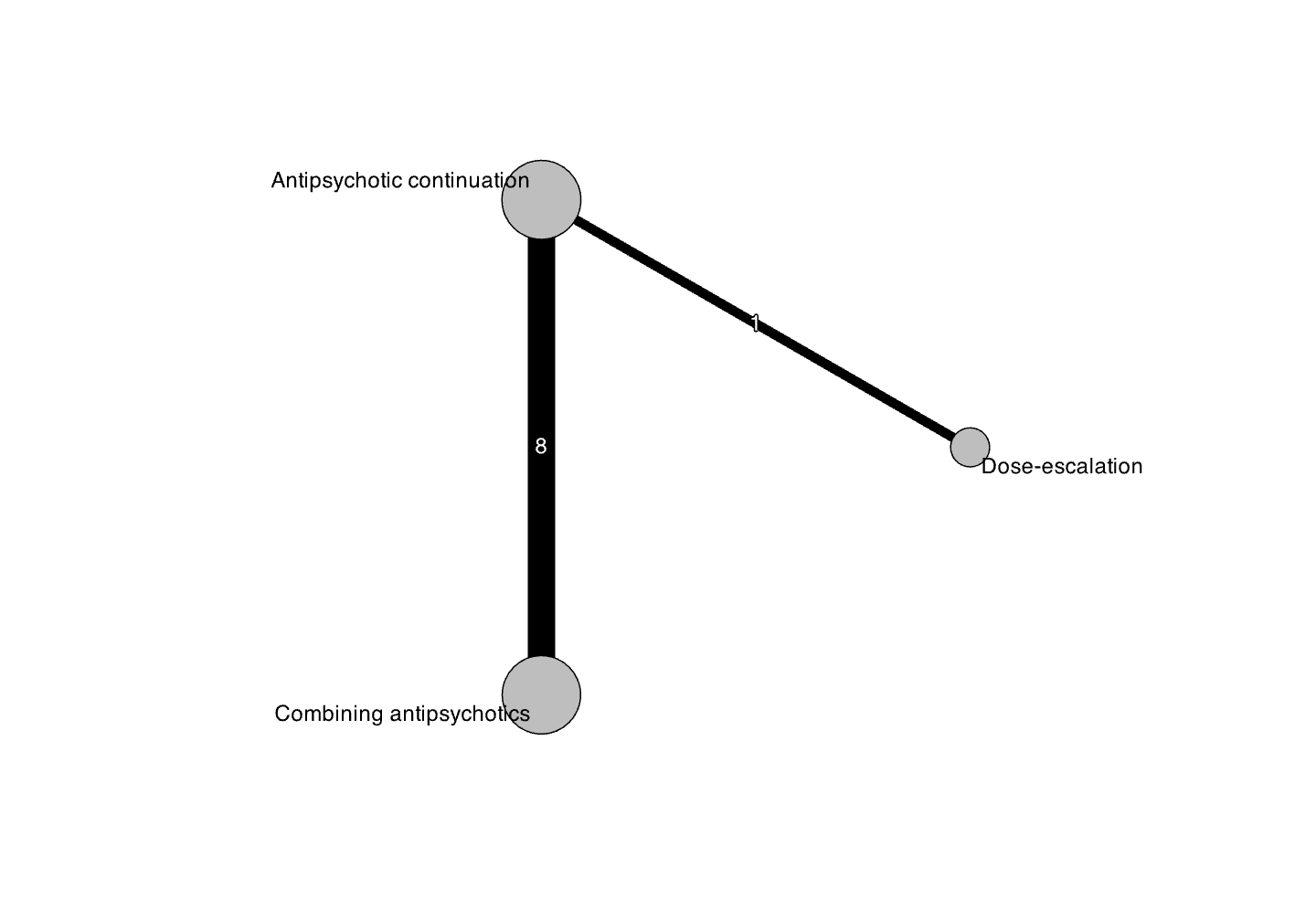

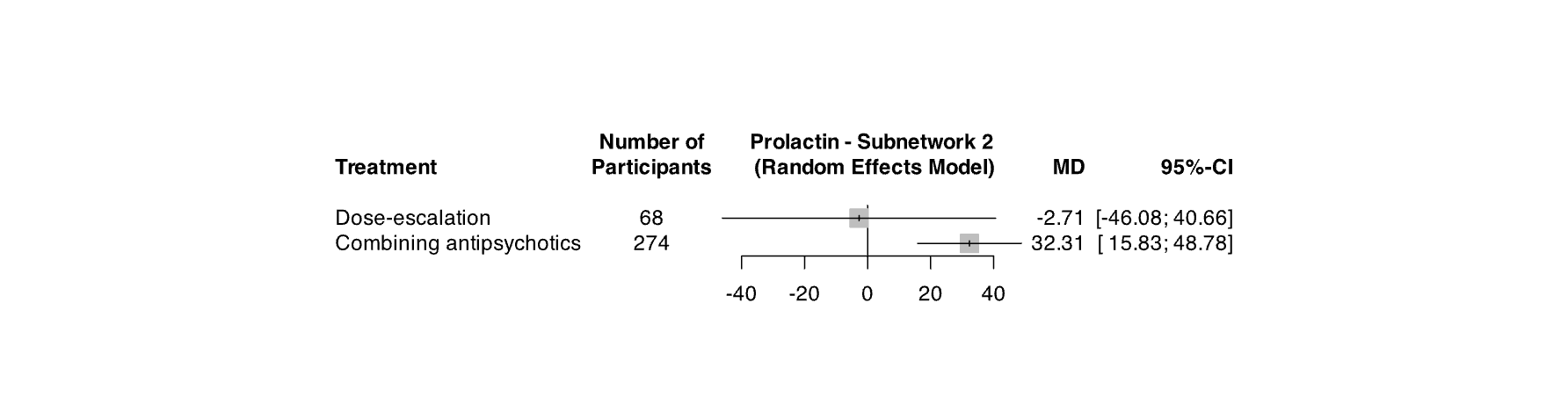


## (xiii) QTc prolongation (ms, continuous)

Subnetwork 1 (antipsychotic continuation as a reference)


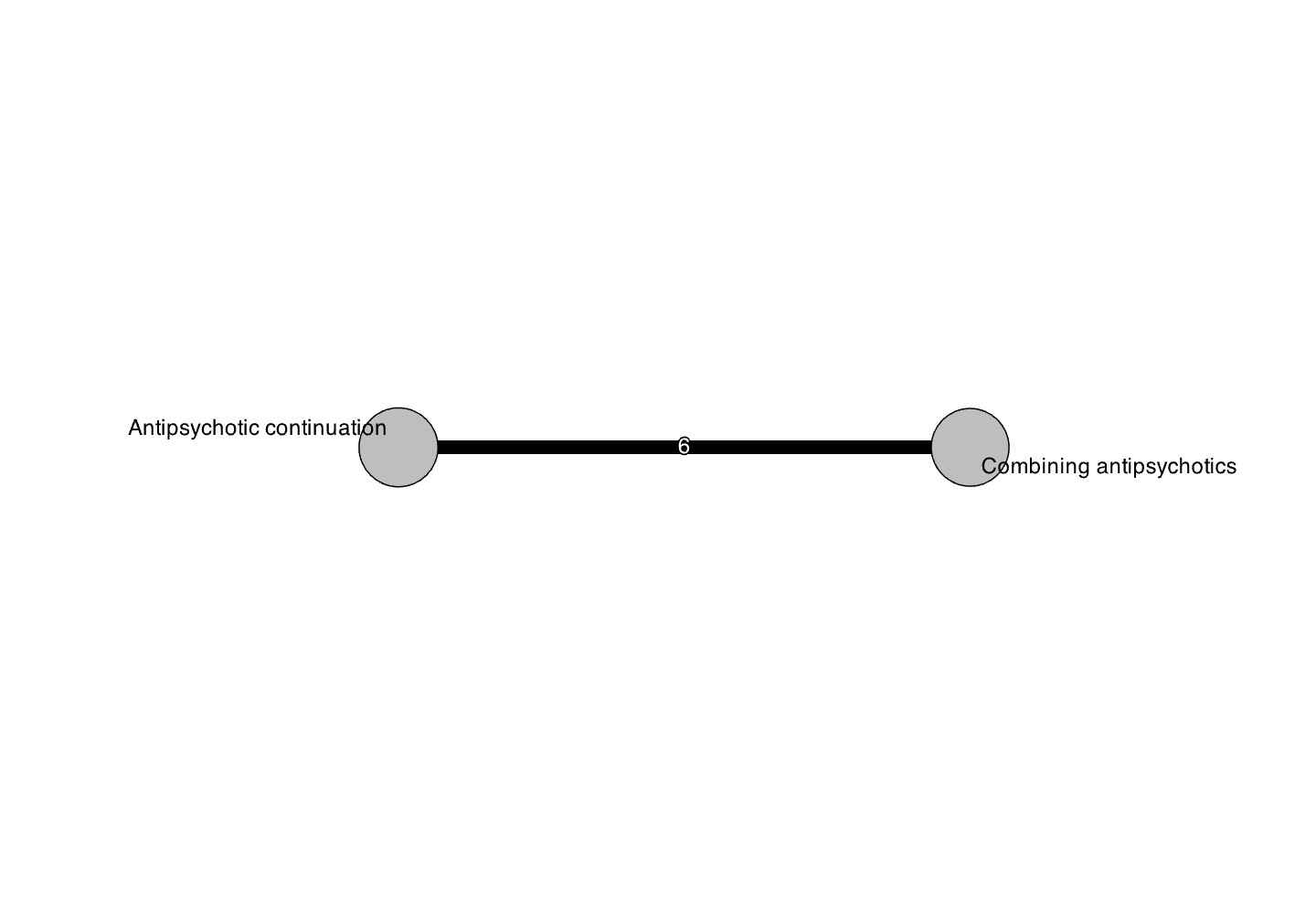

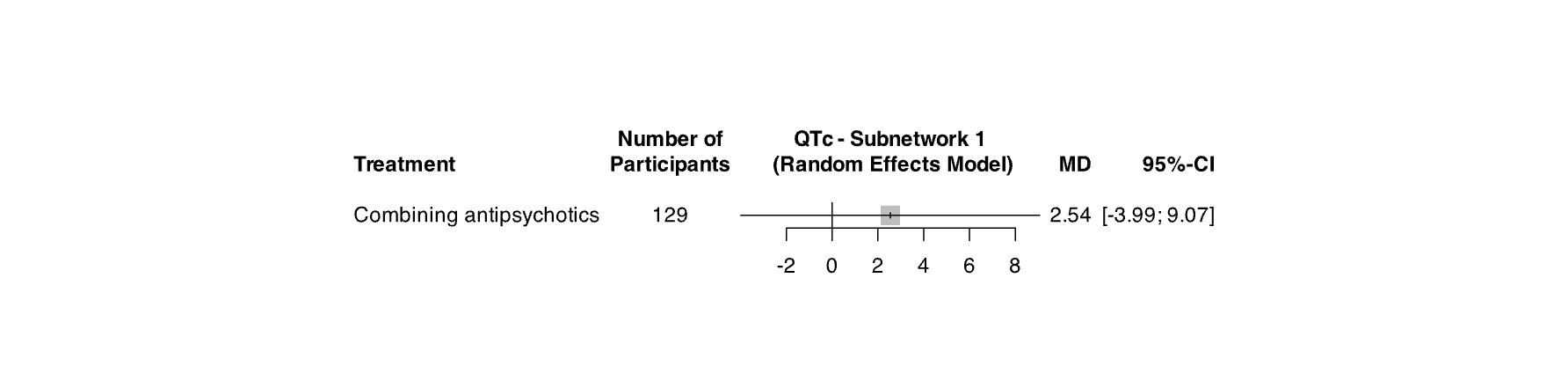


Subnetwork 2 (switching to clozapine as a reference)


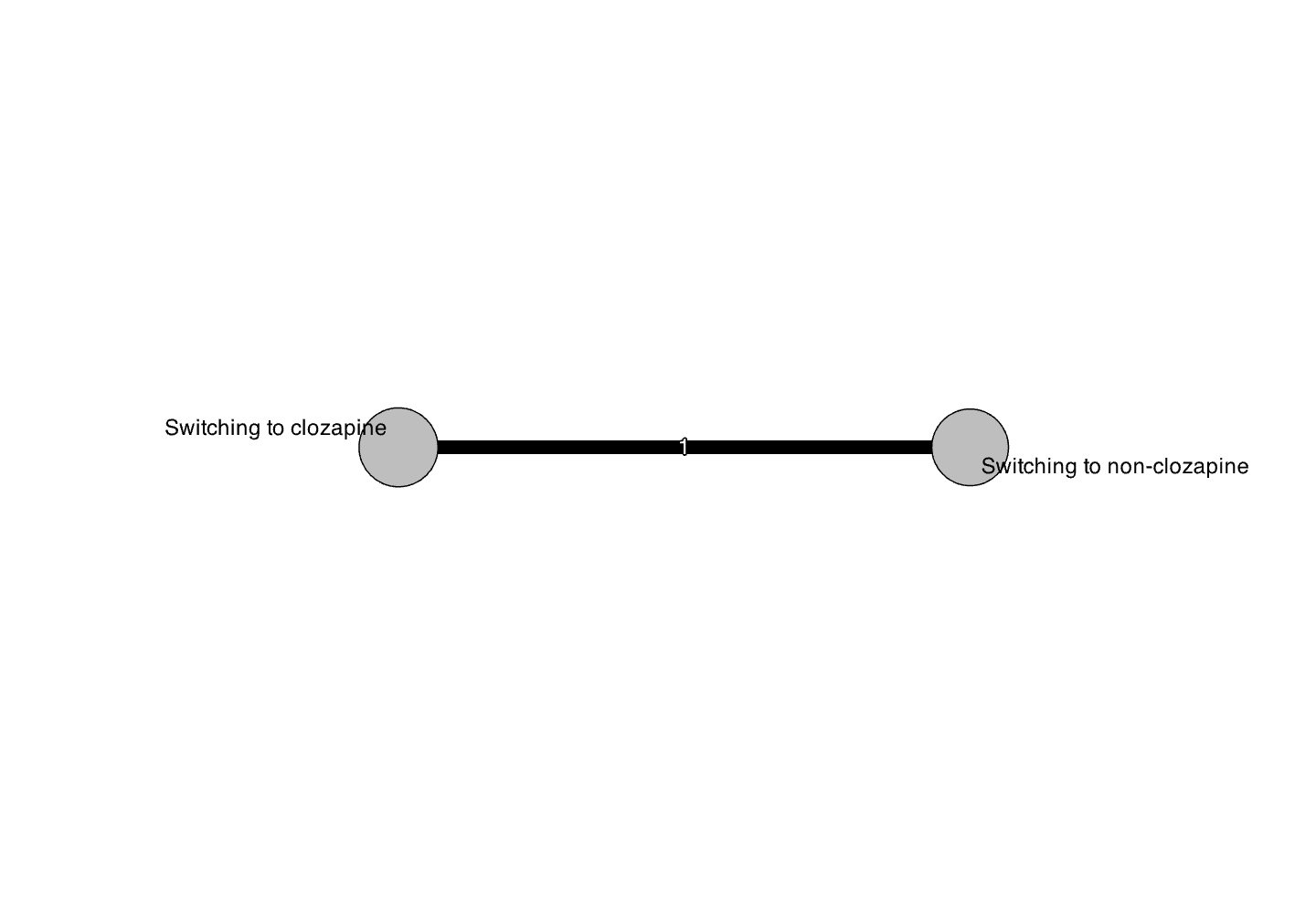

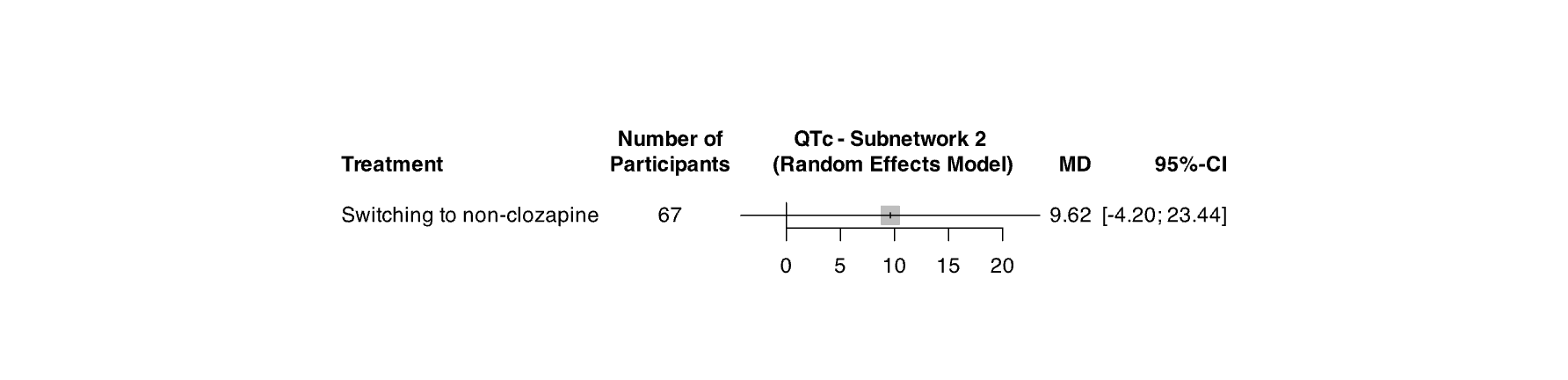


## (xiv) death (dichotomous)

Subnetwork 1 (switching to clozapine as a reference)


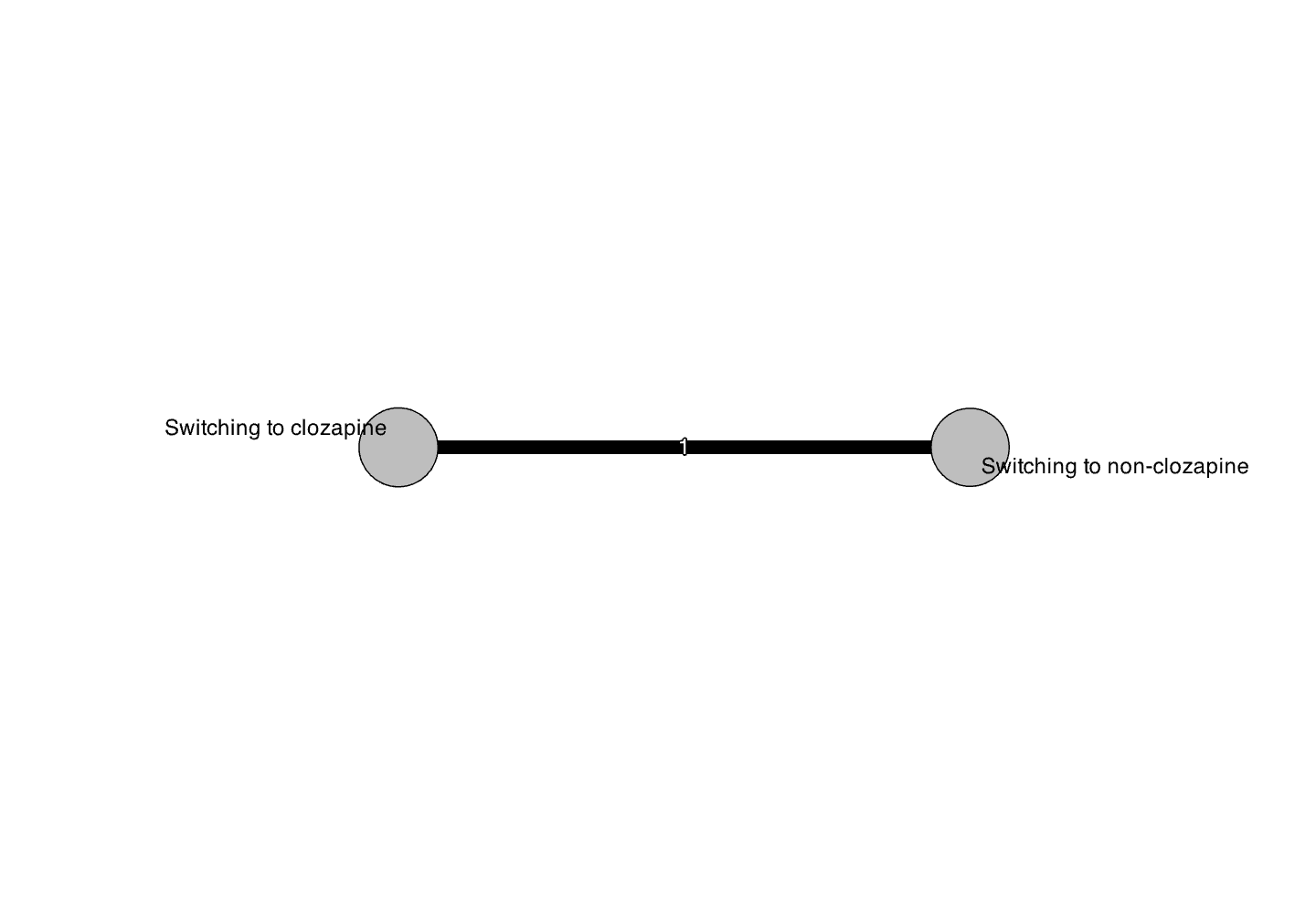


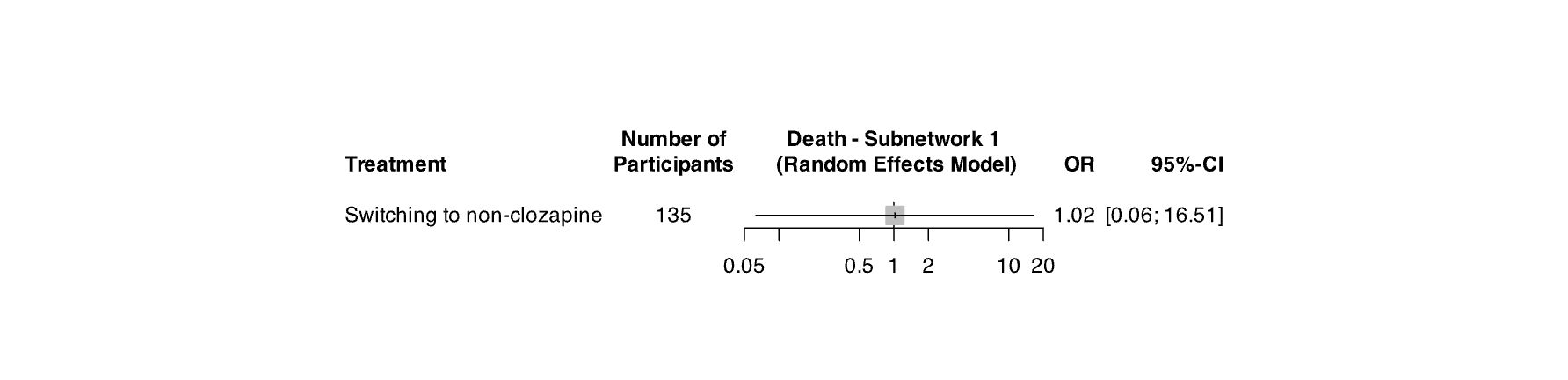


Subnetwork 2 (antipsychotic continuation as a reference)


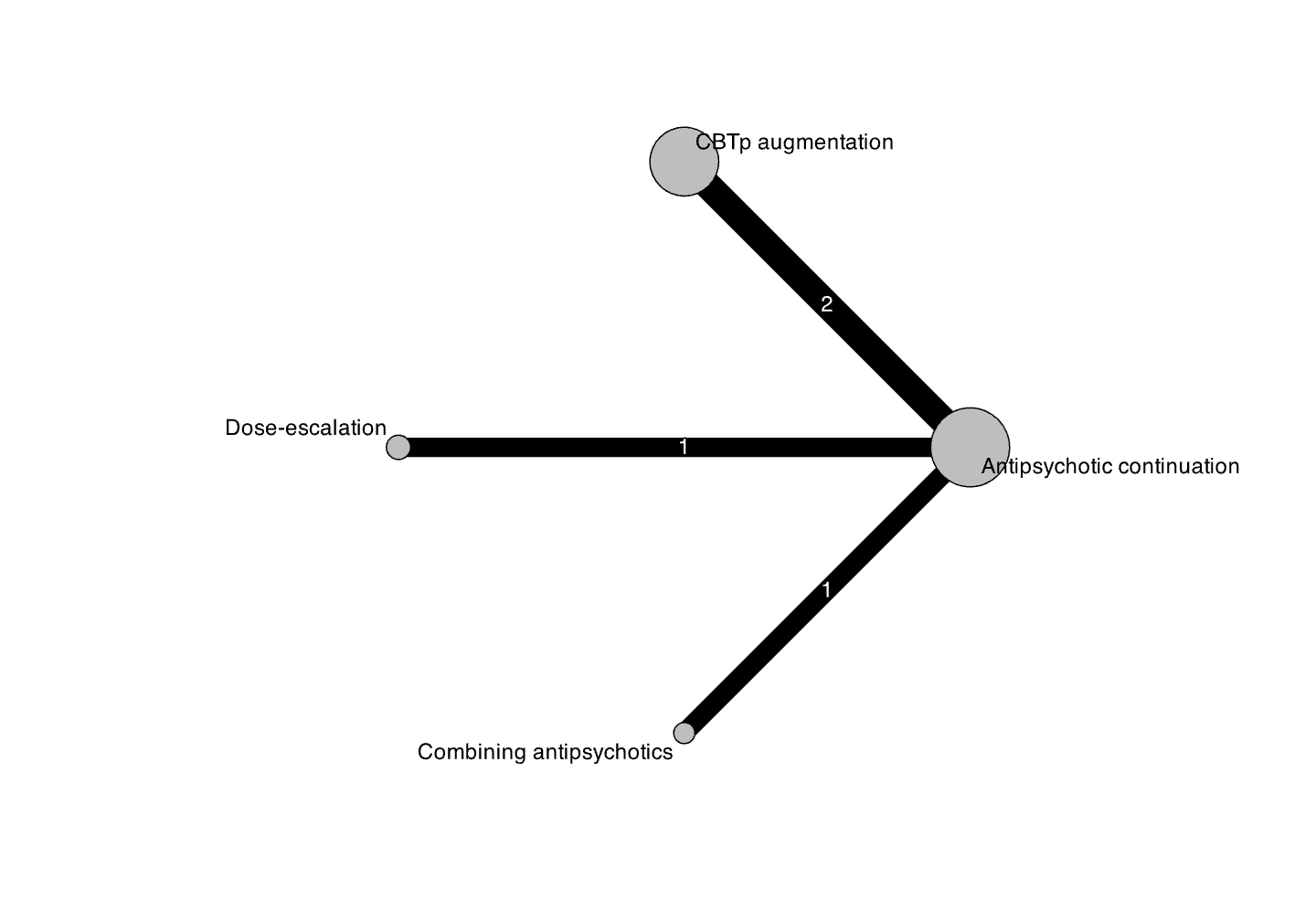

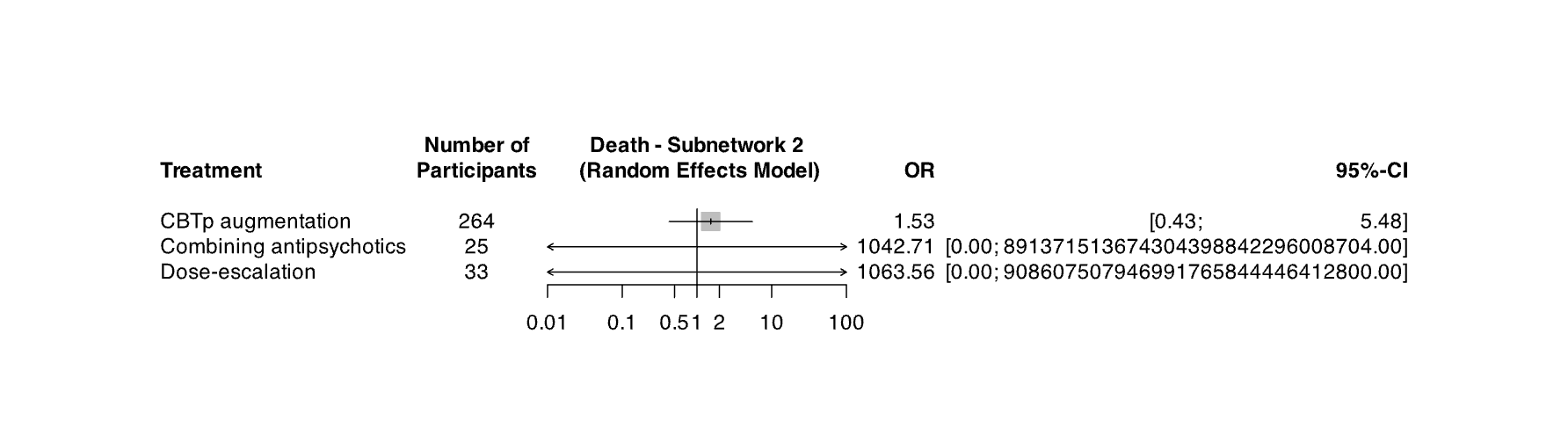


# 9. SENSITIVITY ANALYSES

Summary


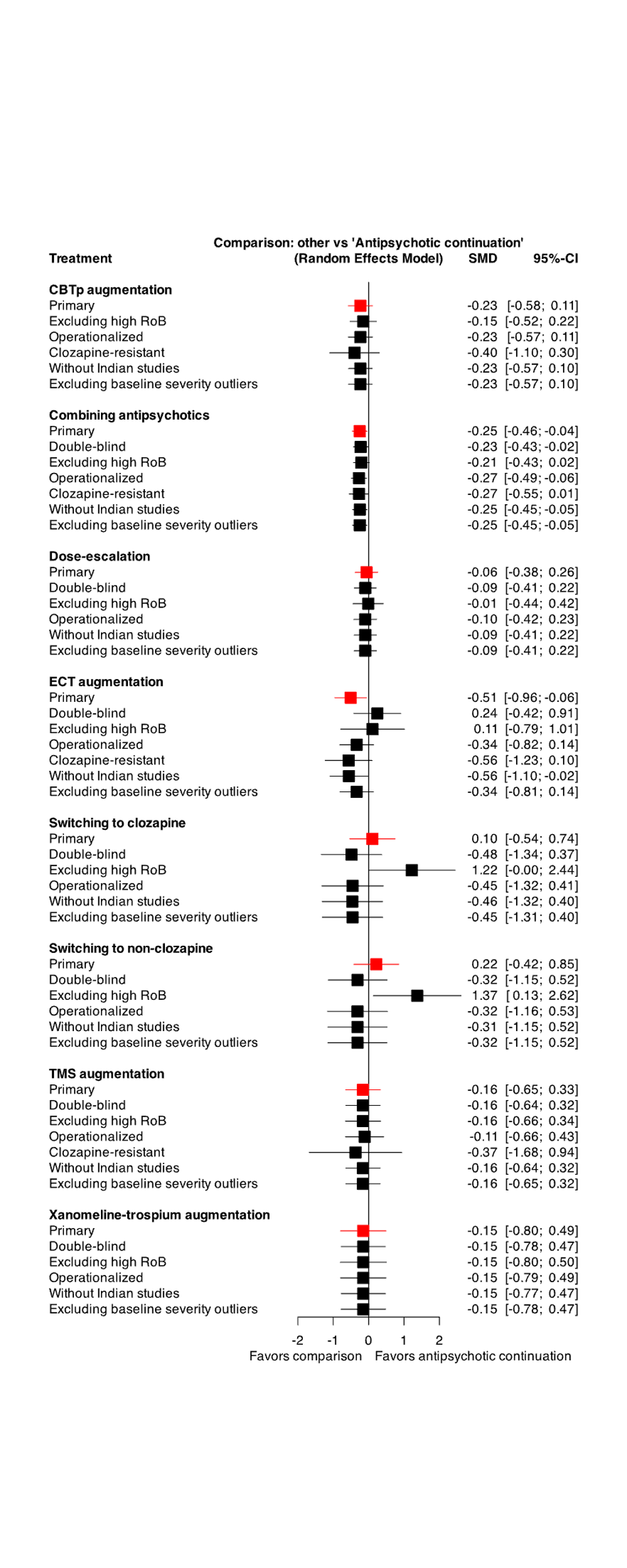


## S1. Focusing on double-blind trials


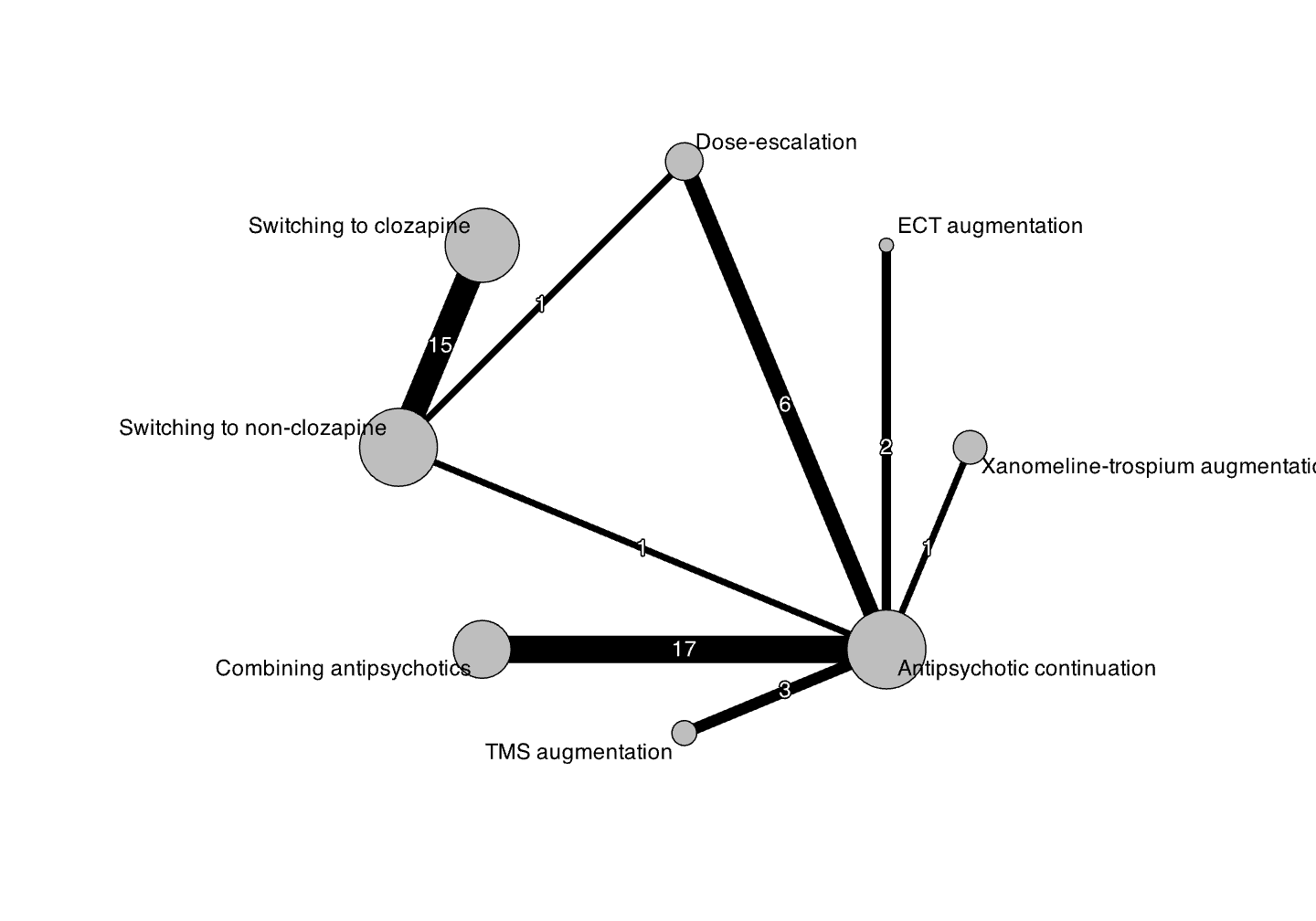


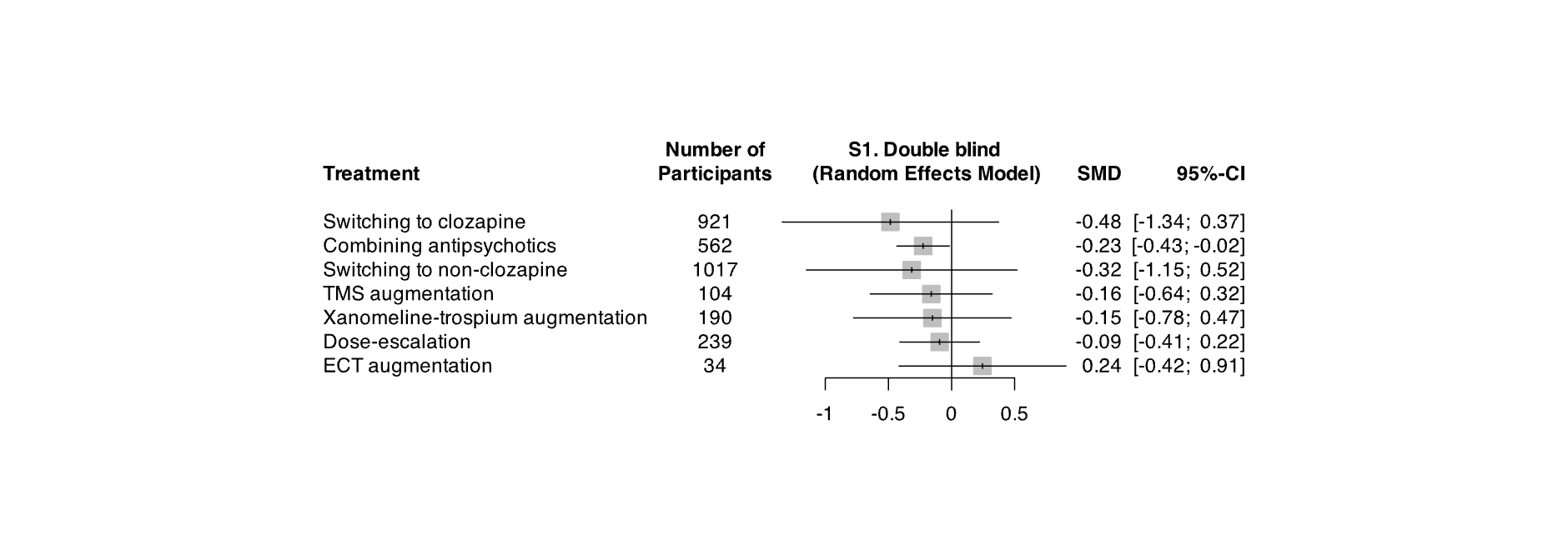


## S2. Excluding high risk of bias trials

A sensitivity analysis suggested negative effects of switching to clozapine and switching to non-clozapine compared to antipsychotic continuation, but this reflects a sparse network of the analysis: neither strategies were directly connected to antipsychotic continuation.
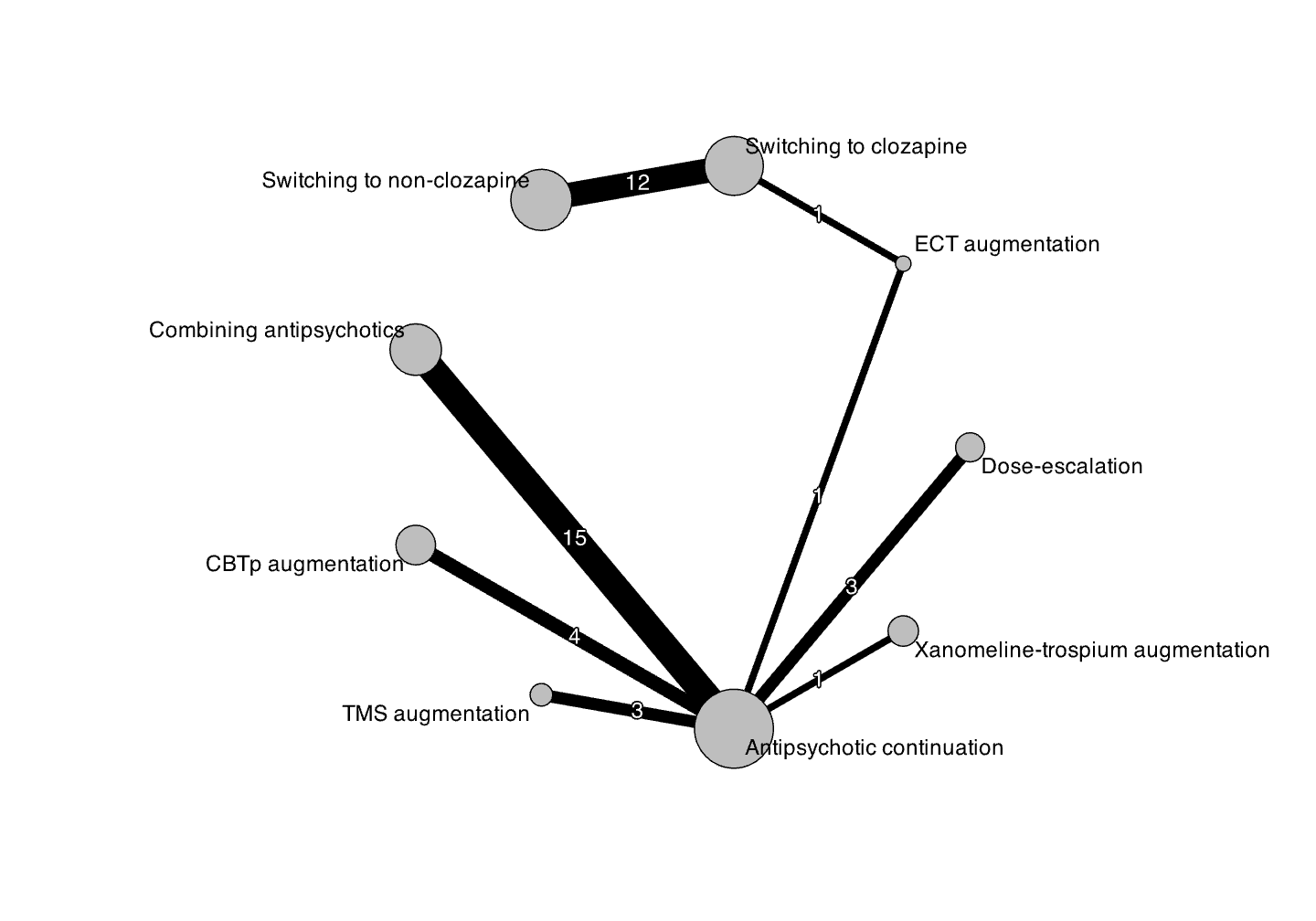

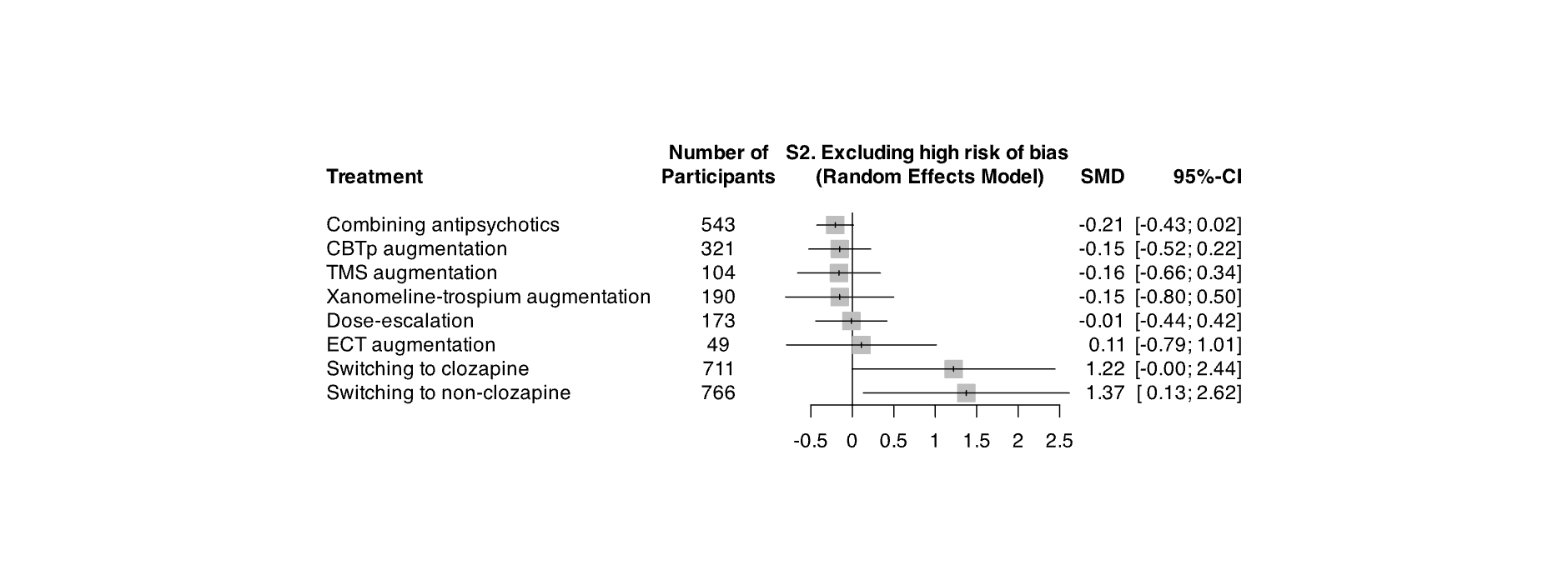


## S3. Excluding trials without operationalized diagnostic criteria


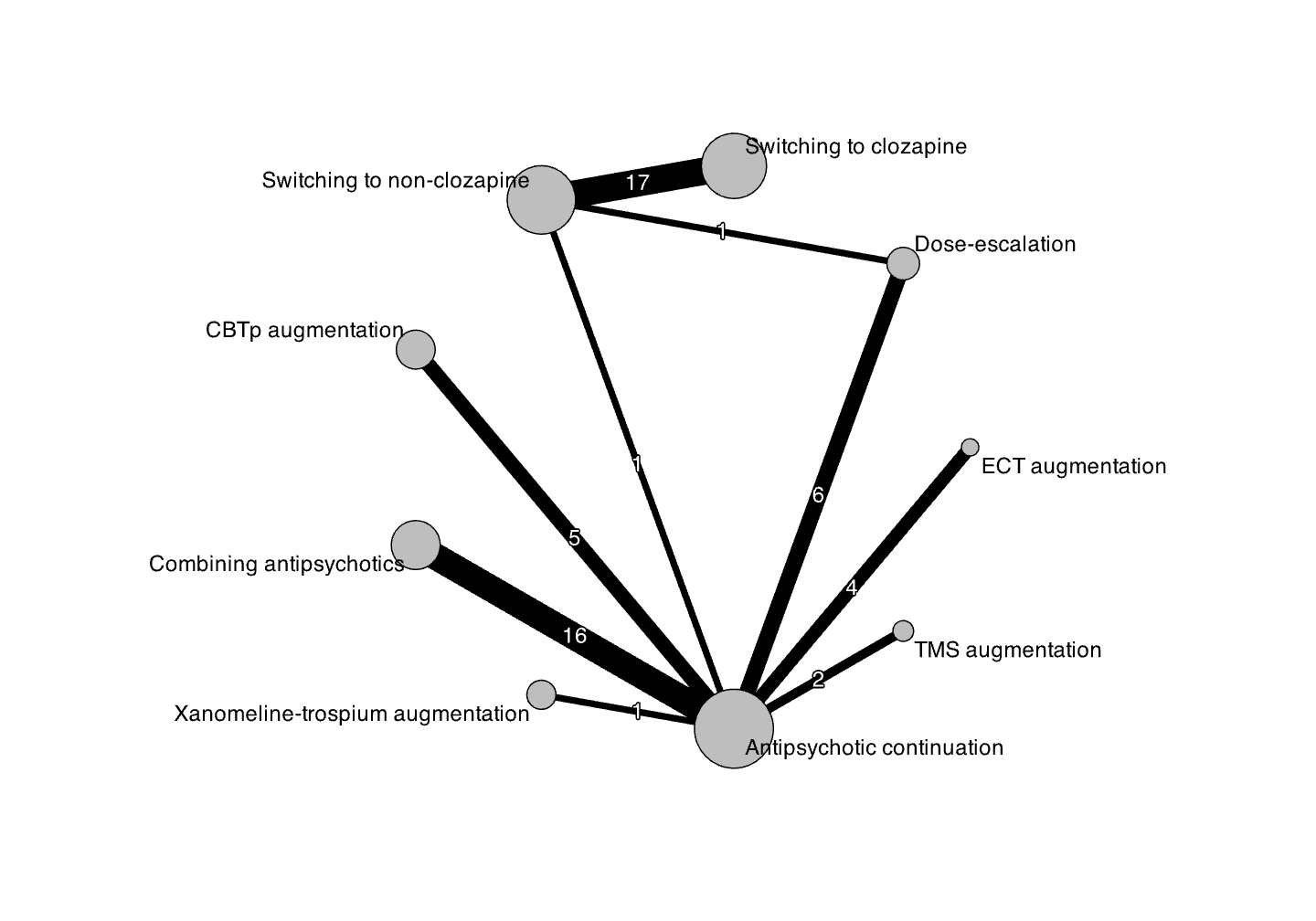

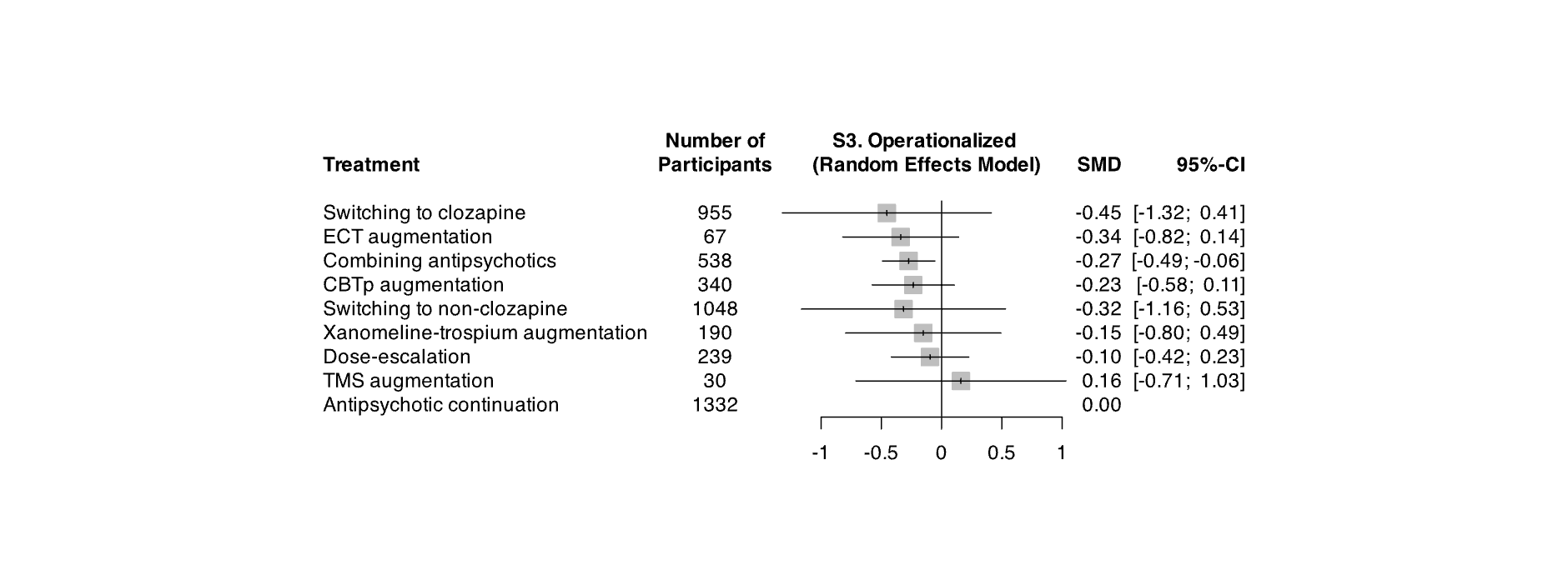


## S4.1. Focusing on clozapine-resistant patients


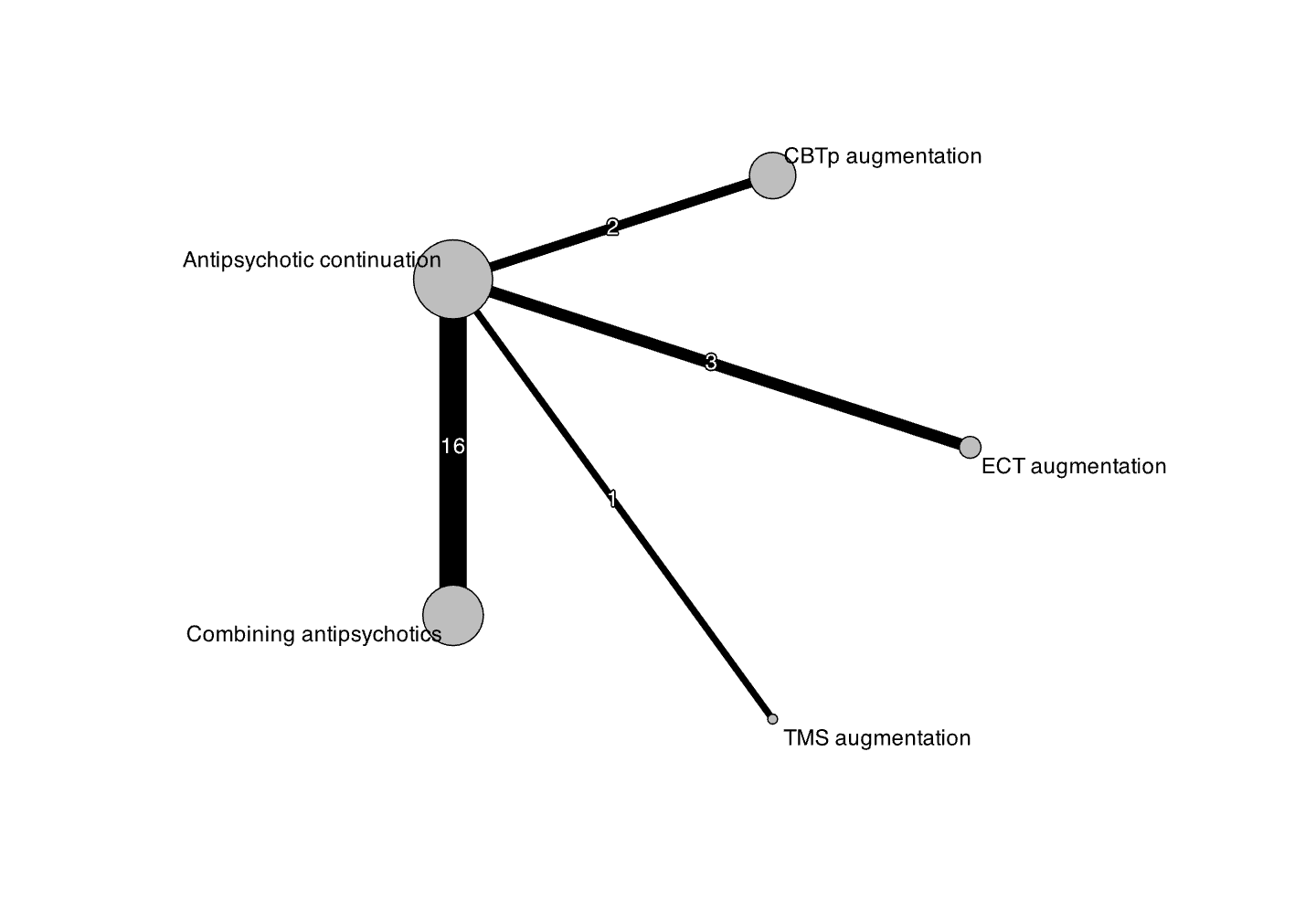

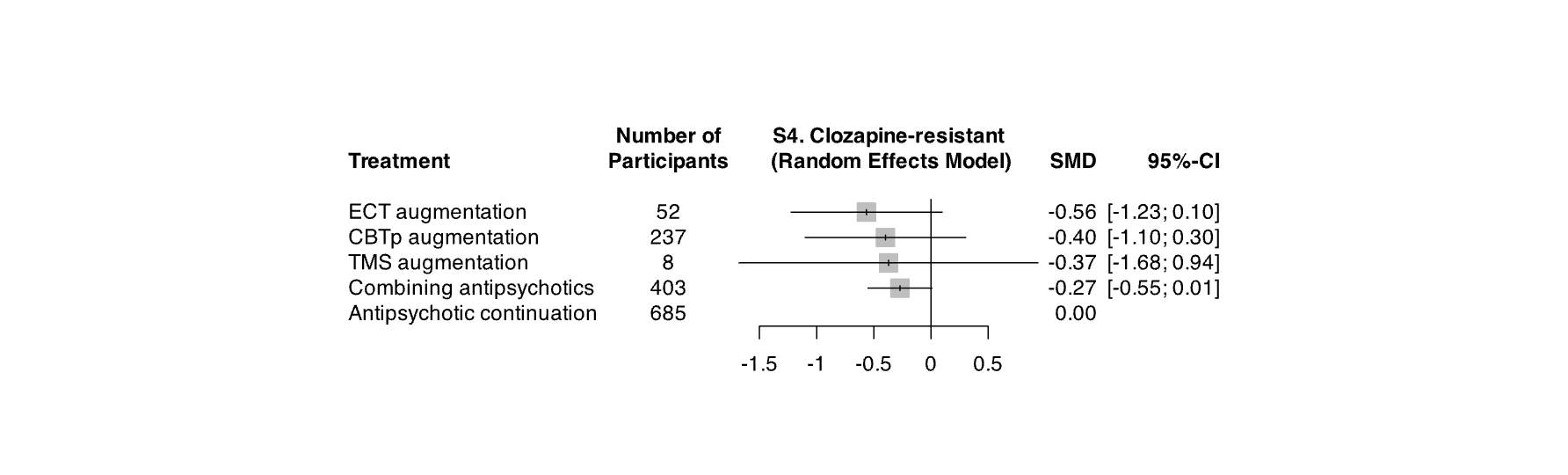


## S4.2. Focusing on TRRIP-criteria


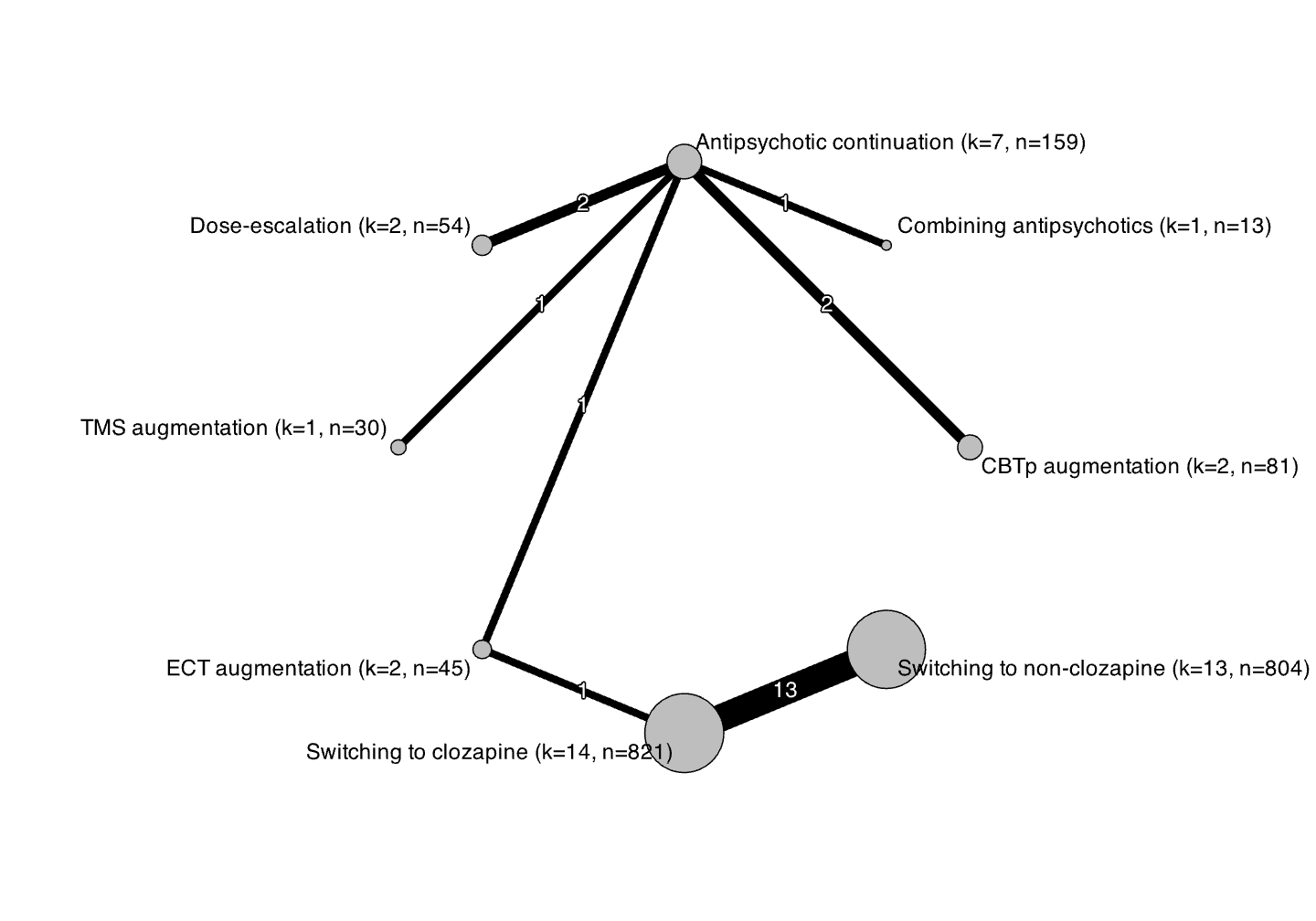


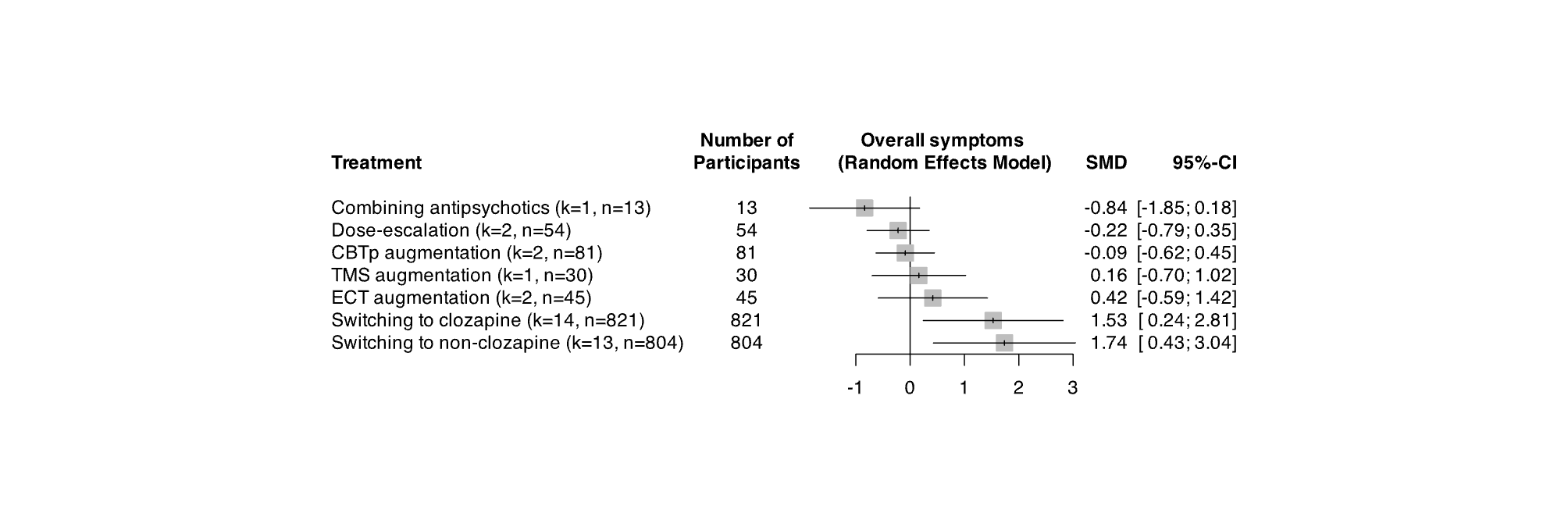
S5. Excluding studies from countries with less experience


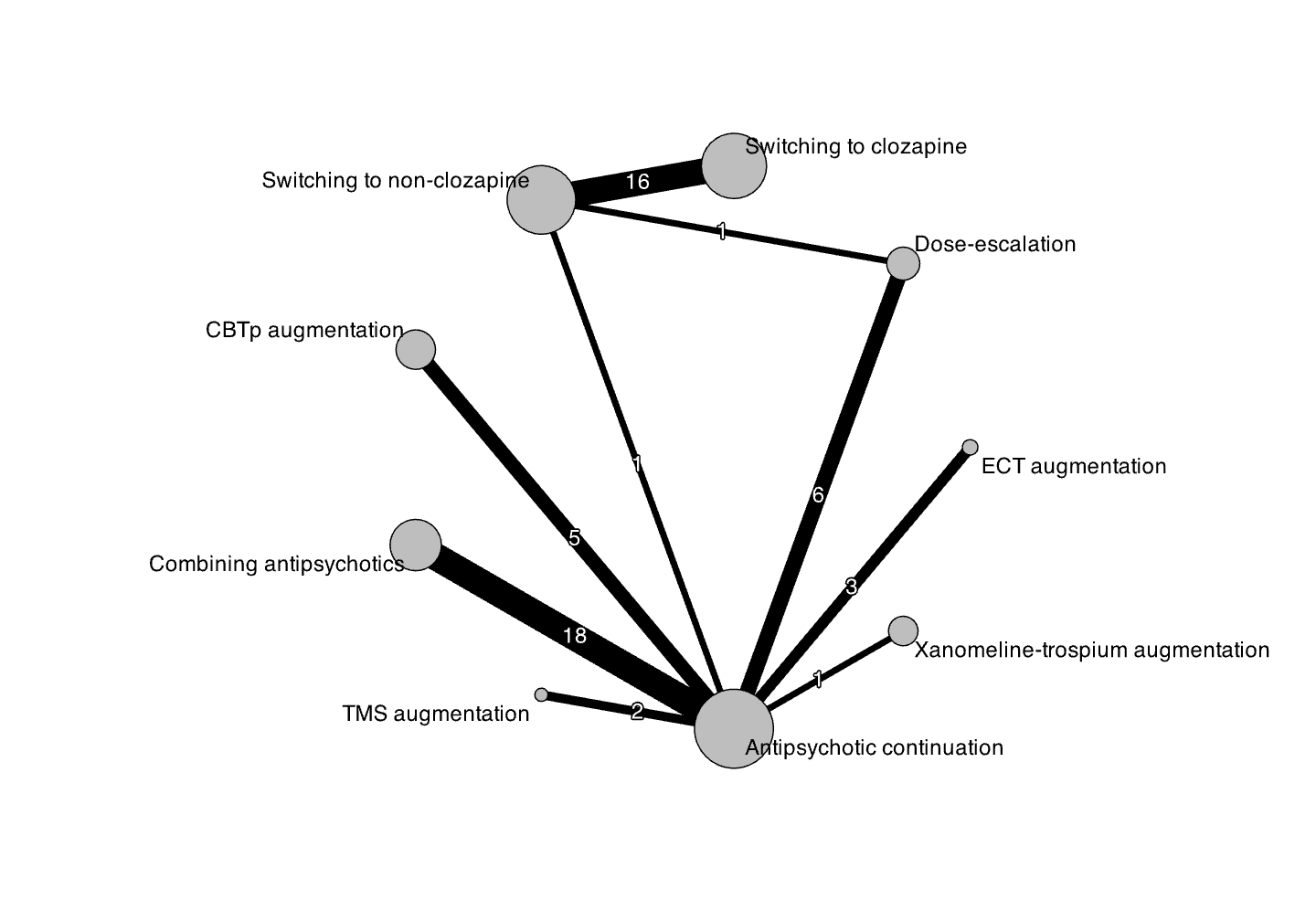

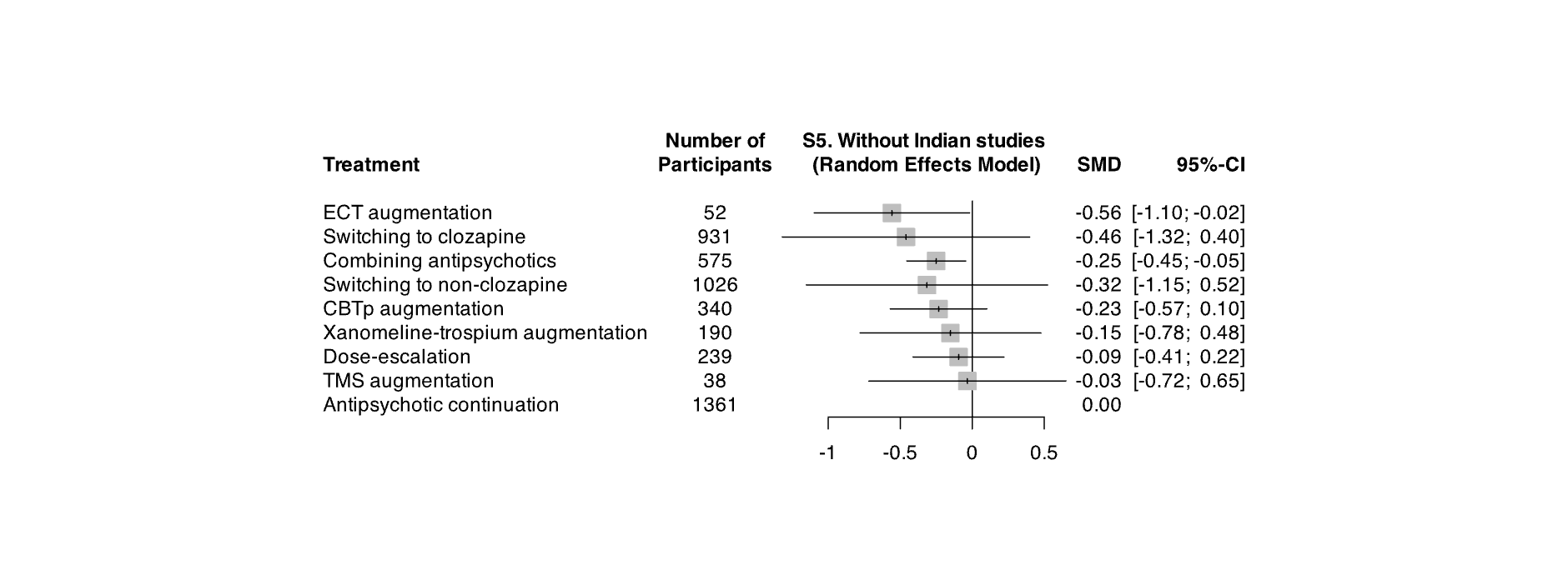


## S6. Excluding baseline severity outlier (Mishra 2022)


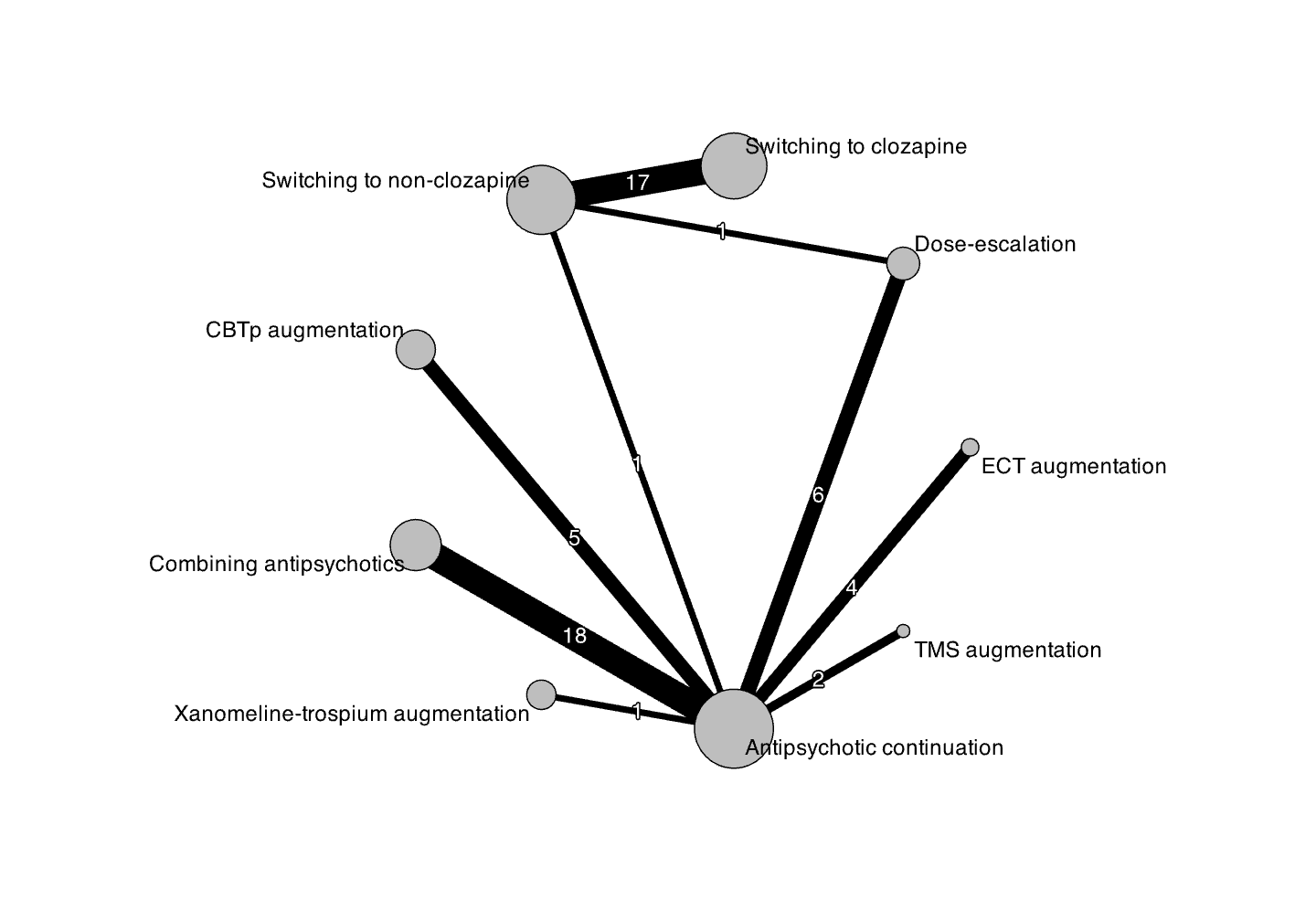

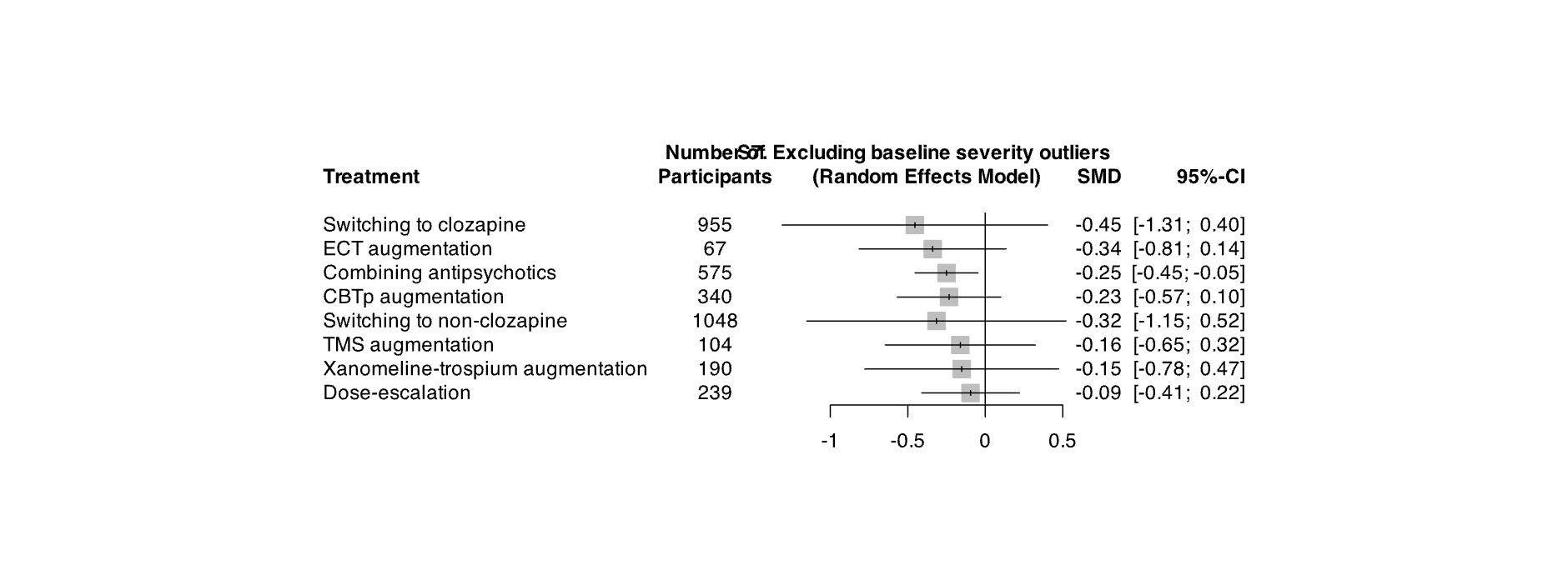


## S7. Subgroup analyses of combination trials (post hoc)

**
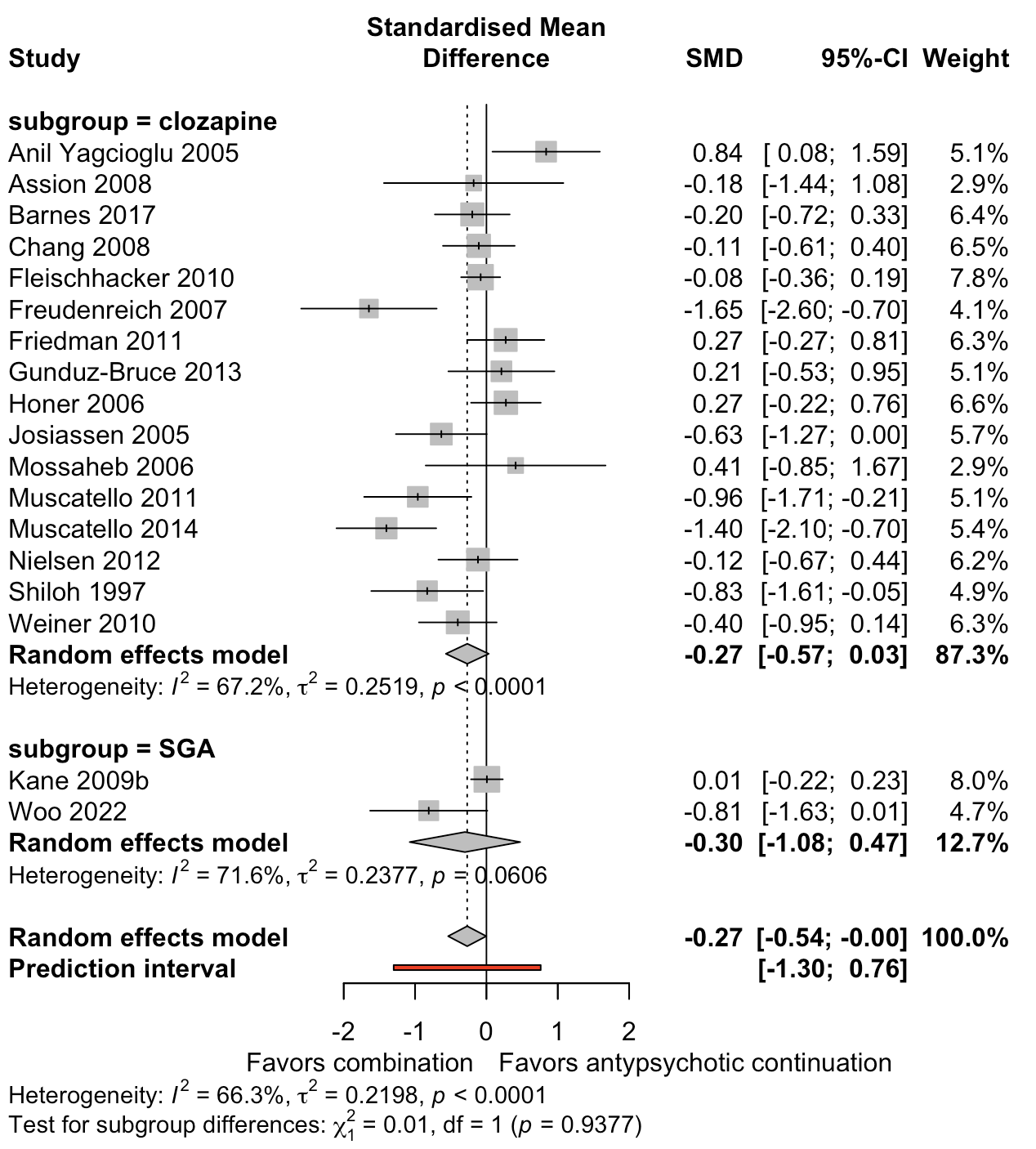
**

**
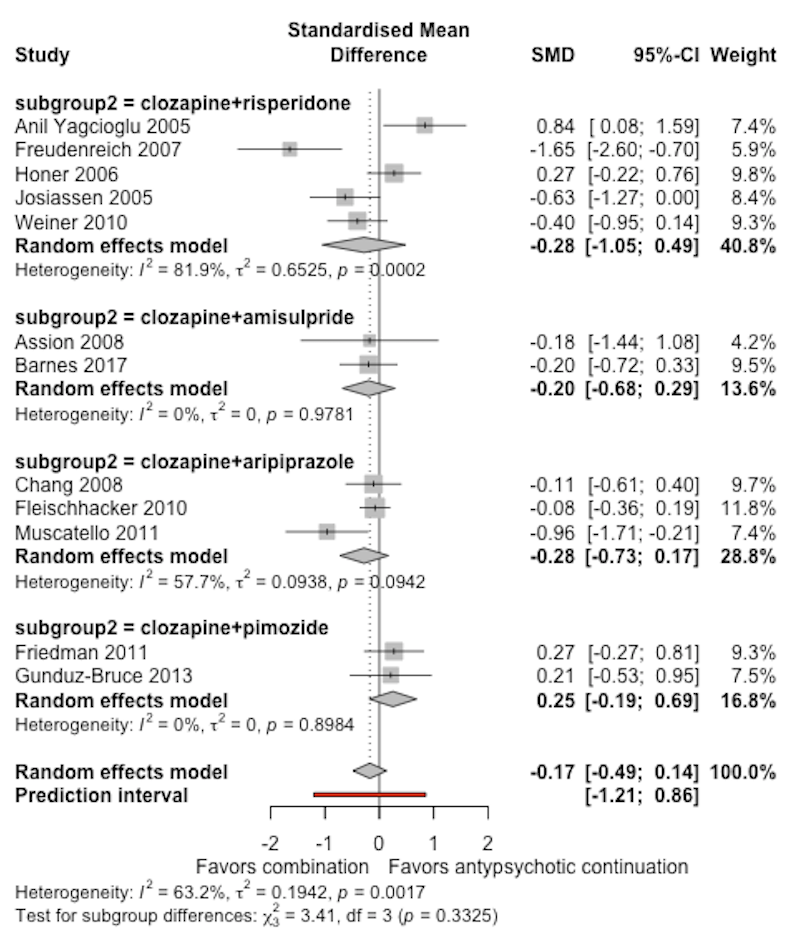
**

**
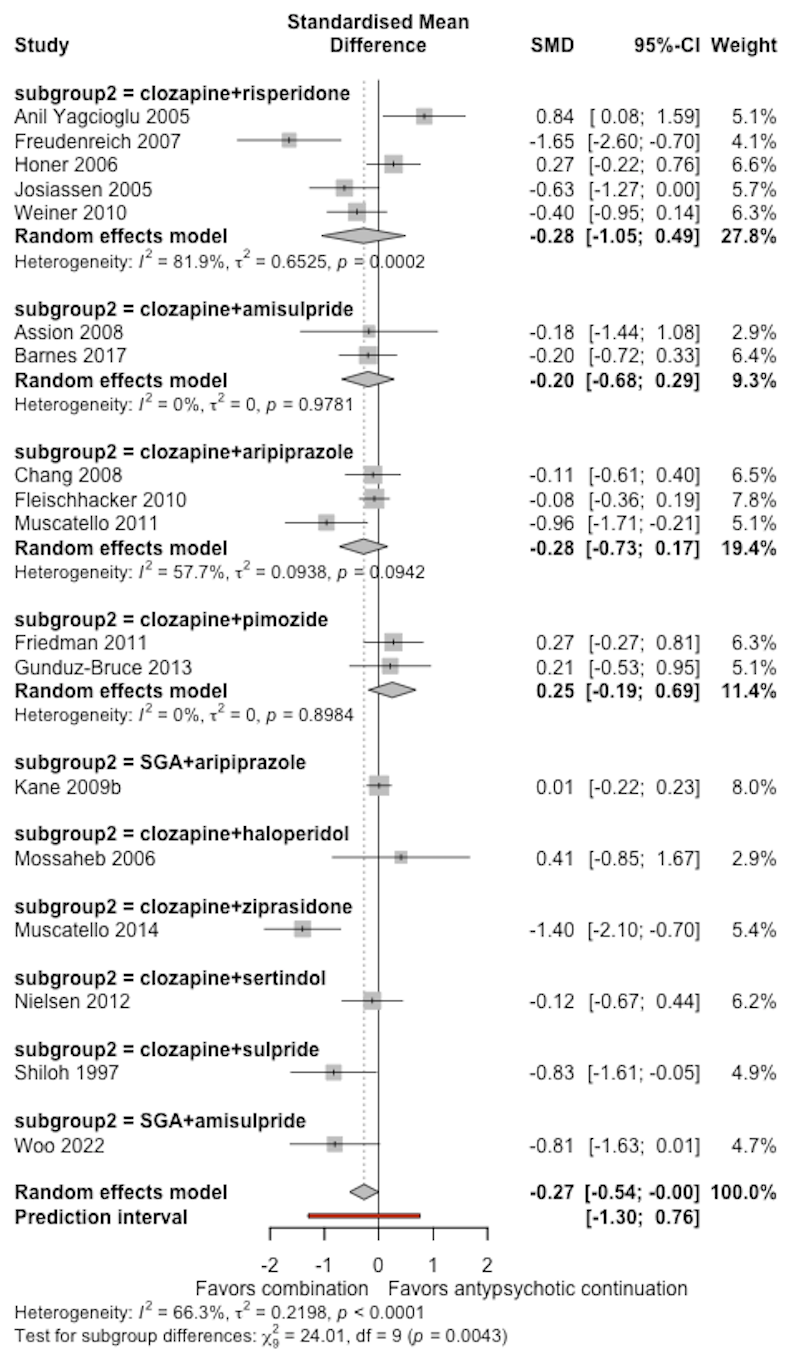
**

## S8. Excluding Salganik 1998 (only triak on the elder population) (post hoc)

**
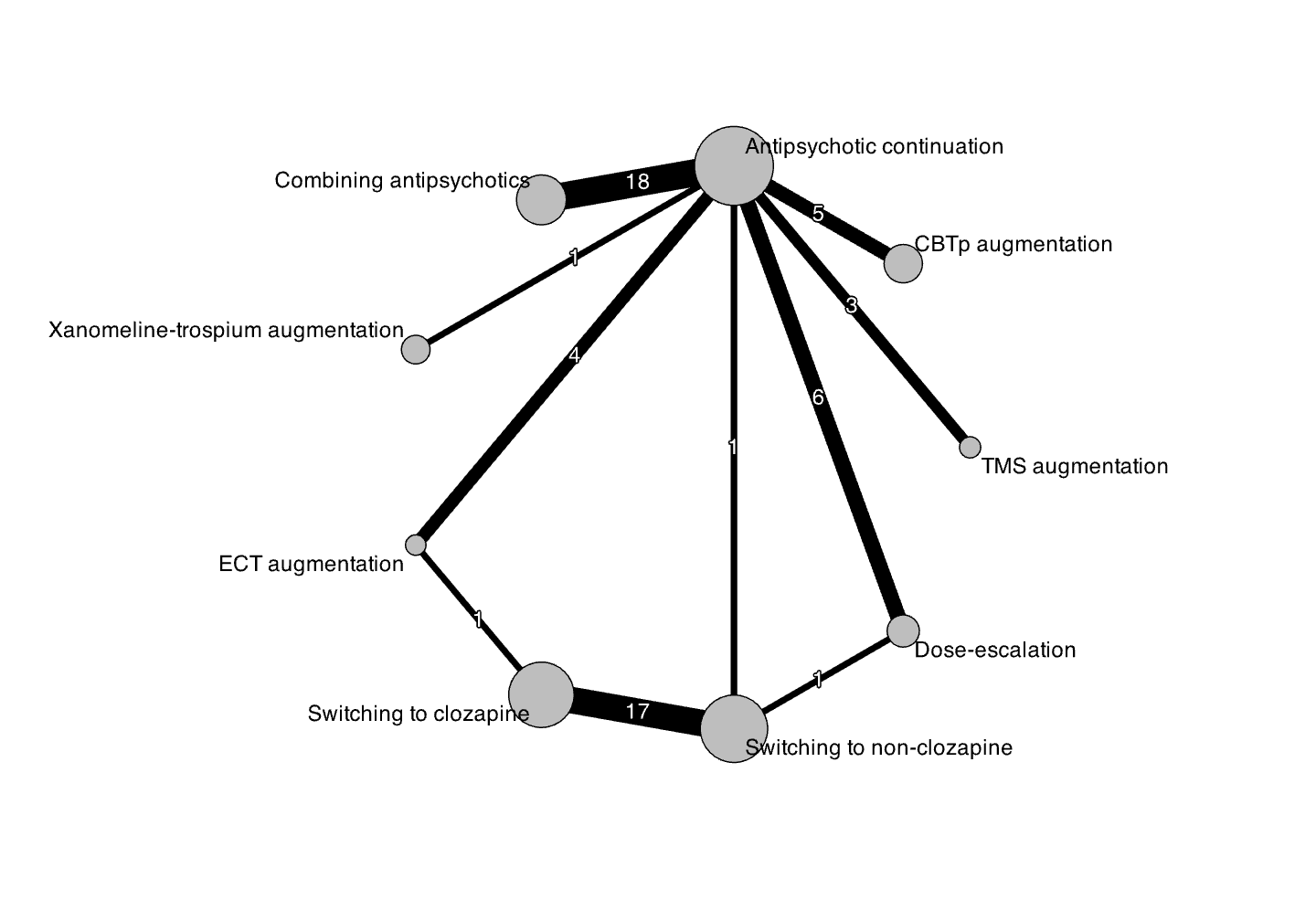

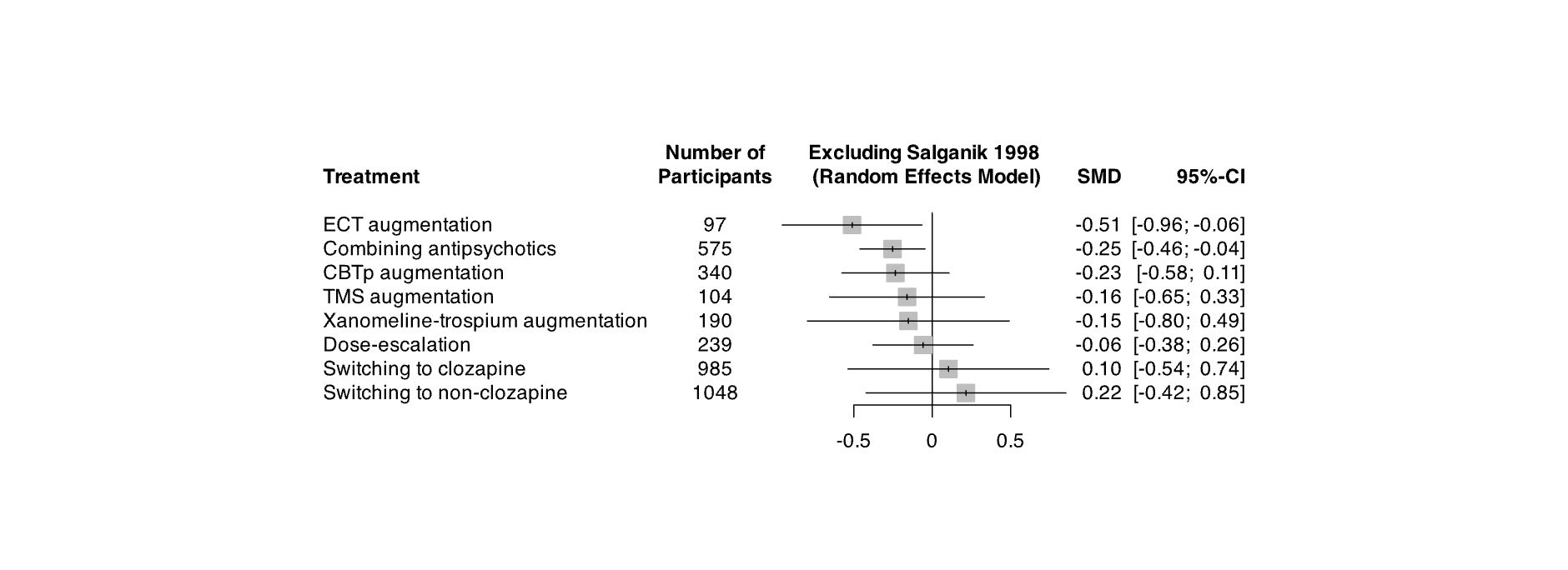
**

## S9. Component Network Meta-Analysis (*post hoc*)


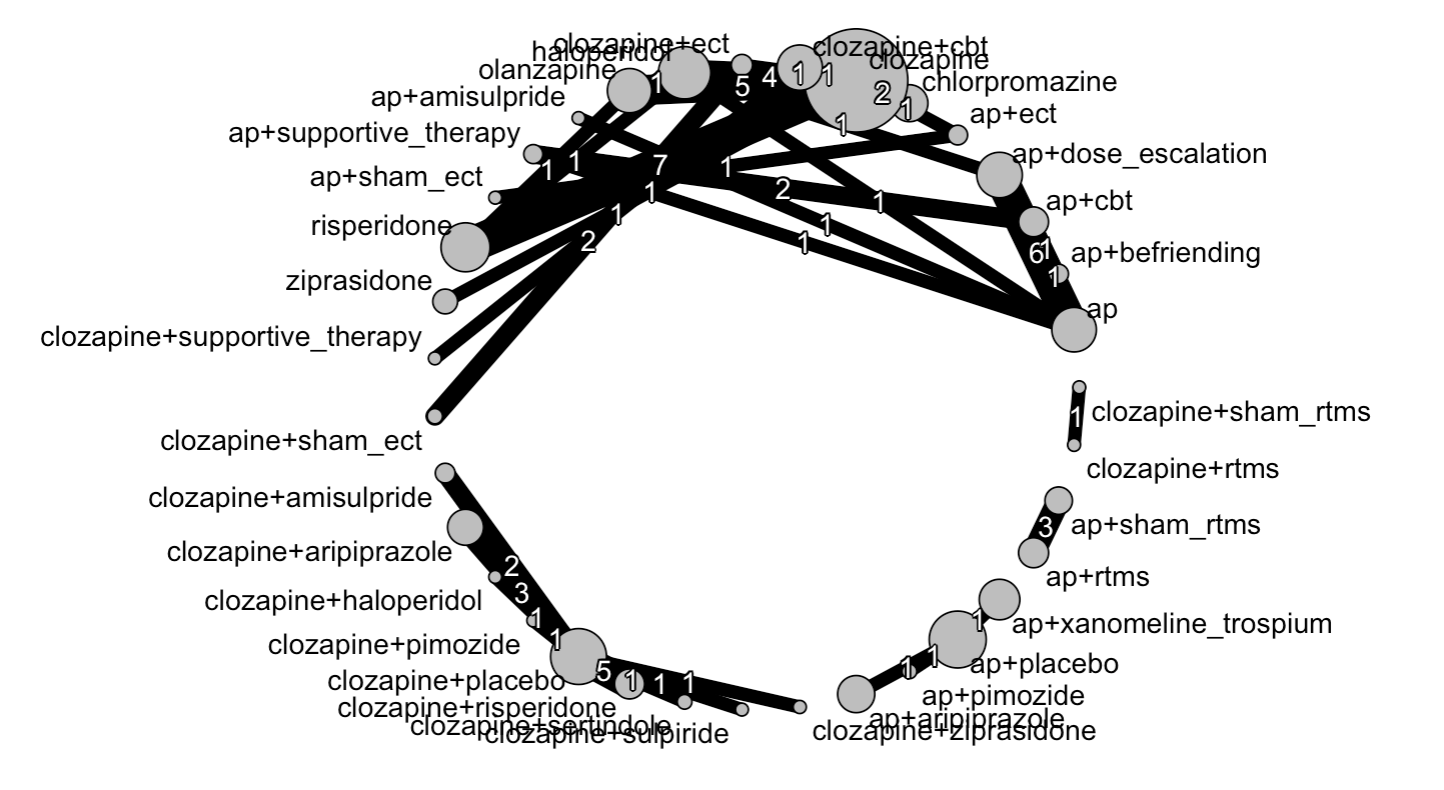


Number of studies: k = 56

Number of pairwise comparisons: m = 65

Number of treatments: n = 35

Number of active components: c = 22

Number of designs: d = 31

Number of subnetworks: s = 5

Quantifying heterogeneity / inconsistency:

tau^2 = 0.0451; tau = 0.2123; I^2 = 41.4% [14.4%; 59.9%]

Heterogeneity statistic:

Q df p-value

66.54 39 0.0039

Details of network meta-analysis methods:

- Frequentist graph-theoretical approach

- Component network meta-analysis

- DerSimonian-Laird estimator for tau^2

- Calculation of I^2 based on Q

Incremental effect for components:

iSMD 95%-CI z p-value

amisulpride -0.8361 [-1.7540; 0.0818] -1.79 0.0742

ap -0.6059 [-1.9196; 0.7077] -0.90 0.3660

aripiprazole -0.7319 [-1.8643; 0.4005] -1.27 0.2052

befriending 0.0697 [-0.6298; 0.7691] 0.20 0.8452

cbt -0.1807 [-0.5601; 0.1986] -0.93 0.3504

chlorpromazine -0.1093 [-1.3364; 1.1178] -0.17 0.8614

clozapine -0.9545 [-2.1108; 0.2018] -1.62 0.1057

dose_escalation -0.0784 [-0.3389; 0.1822] -0.59 0.5555

ect -1.3318 [-1.9662; -0.6974] -4.11 < 0.0001

haloperidol -0.7666 [-1.9454; 0.4121] -1.27 0.2024

olanzapine -1.0471 [-2.2299; 0.1357] -1.74 0.0827

pimozide -0.3252 [-1.5436; 0.8933] -0.52 0.6009

placebo -0.5766 [-1.6723; 0.5191] -1.03 0.3023

risperidone -0.8675 [-2.0075; 0.2725] -1.49 0.1358

rtms NA . . .

sertindole -0.6970 [-1.9937; 0.5998] -1.05 0.2922

sham_ect -1.3130 [-2.1211; -0.5050] -3.18 0.0014

sham_rtms NA . . .

sulpiride -1.4318 [-2.8404; -0.0232] -1.99 0.0463

supportive_therapy 0.0669 [-0.4413; 0.5751] 0.26 0.7964

xanomeline_trospium -0.7286 [-1.9176; 0.4604] -1.20 0.2298

ziprasidone -1.2853 [-2.4951; -0.0754] -2.08 0.0373

“Incremental effect” refers to the effect of the presence of a component compared the absence of the component. The incremental effect of rTMS and sham rTMS was not calculable.


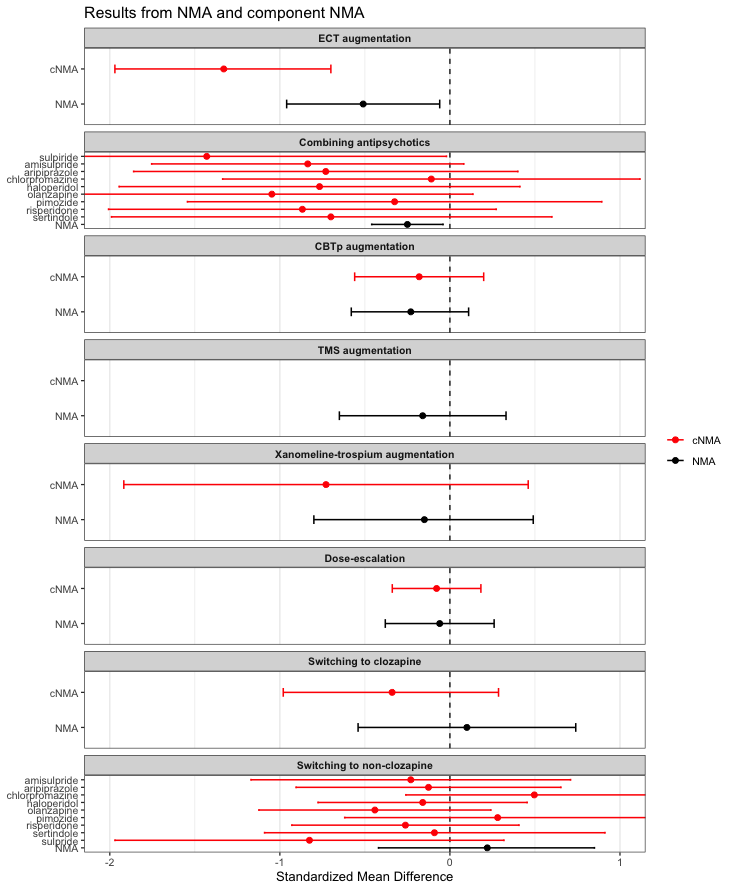


The reference for cNMA results were continuation of the previous antipsychotic alone
